# Sequence of the supernumerary B chromosome of maize provides insight into its drive mechanism and evolution

**DOI:** 10.1073/pnas.2104254118

**Published:** 2021-06-04

**Authors:** Nicolas Blavet, Hua Yang, Handong Su, Pavel Solanský, Ryan N. Douglas, Miroslava Karafiátová, Lucie Šimková, Jing Zhang, Yalin Liu, Jie Hou, Xiaowen Shi, Chen Chen, Mohamed El-Walid, Morgan E. McCaw, Patrice S. Albert, Zhi Gao, Changzeng Zhao, Gil Ben-Zvi, Lior Glick, Guy Kol, Jinghua Shi, Jan Vrána, Hana Šimková, Jonathan C. Lamb, Kathleen Newton, R. Kelly Dawe, Jaroslav Doležel, Tieming Ji, Kobi Baruch, Jianlin Cheng, Fangpu Han, James A. Birchler, Jan Bartoš

**Affiliations:** ^a^Centre of the Region Haná for Biotechnological and Agricultural Research, Institute of Experimental Botany of the Czech Academy of Sciences, Olomouc 77900, Czech Republic;; ^b^Division of Biological Sciences, University of Missouri, Columbia, MO 65211;; ^c^State Key Laboratory of Plant Cell and Chromosome Engineering, Institute of Genetics and Developmental Biology, Innovation Academy for Seed Design, Chinese Academy of Sciences, 100101 Beijing, China;; ^d^Department of Electrical Engineering and Computer Science, University of Missouri, Columbia, MO 65211;; ^e^NRGene, Ness-Ziona 7403649, Israel;; ^f^Bionano Genomics, San Diego, CA 92121;; ^g^Bayer Crop Science, Chesterfield, MO 63017;; ^h^Department of Plant Biology, University of Georgia, Athens, GA 30602;; ^i^Department of Statistics, University of Missouri, Columbia, MO 65211;; ^j^College of Advanced Agricultural Science, University of Chinese Academy of Sciences, 100049 Beijing, China

**Keywords:** B chromosome, genetic drive, nondisjunction, preferential fertilization

## Abstract

B chromosomes are enigmatic elements in thousands of plant and animal genomes that persist in populations despite being nonessential. They circumvent the laws of Mendelian inheritance but the molecular mechanisms underlying this behavior remain unknown. Here we present the sequence, annotation, and analysis of the maize B chromosome providing insight into its drive mechanism. The sequence assembly reveals detailed locations of the elements involved with the *cis* and *trans* functions of its drive mechanism, consisting of nondisjunction at the second pollen mitosis and preferential fertilization of the egg by the B-containing sperm. We identified 758 protein-coding genes in 125.9 Mb of B chromosome sequence, of which at least 88 are expressed. Our results demonstrate that transposable elements in the B chromosome are shared with the standard A chromosome set but multiple lines of evidence fail to detect a syntenic genic region in the A chromosomes, suggesting a distant origin. The current gene content is a result of continuous transfer from the A chromosomal complement over an extended evolutionary time with subsequent degradation but with selection for maintenance of this nonvital chromosome.

## Significance

B chromosomes are nonvital chromosomes found in thousands of plants and animals that persist through various drive mechanisms. The drive mechanism of the maize B chromosome consists of mitotic nondisjunction at the second pollen division to produce two unequal sperm and then the sperm with the B chromosomes preferentially fertilizes the egg in double fertilization. A high-quality sequence of the maize B chromosome together with genetic analysis reveals the *cis* factor for nondisjunction is a B chromosome-specific repeat interspersed in and around the centromere. The gene and transposable element content of the B chromosome and relaxed purifying selection of transposed protein-encoding genes suggest that the chromosome has been present in the evolutionary lineage for millions of years.

Supernumerary chromosomes were first discovered in the leaf-footed plant bug *Metapodius* more than a century ago (1). Since then, they have been reported in numerous plant, animal, and fungal species (2). A common feature of these so-called B chromosomes is that they are nonessential and are present only in some individuals in the population of a particular species. Through their evolution, they have developed various modes of behavior, e.g., tissue-specific elimination in *Aegilops* (3), preferential fertilization in *Zea* (4), or sex manipulation in *Nasonia* (5). In many plant species, they undergo controlled nondisjunction—unequal allocation to daughter nuclei during postmeiotic divisions (6). Their effect on frequency and distribution of meiotic crossovers along the standard A chromosomes has also been described (7, 8). Despite the peculiar behavior and unclear origins, no high-quality B chromosome reference sequence has been previously obtained in any organism.

**Fig. 1.**
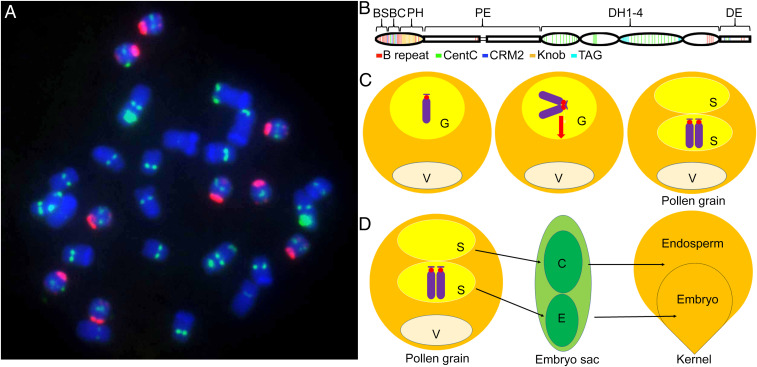
The maize B chromosome. (*A*) Root tip metaphase spread of a line possessing nine B chromosomes (red signal). The red signal identifies the ZmBs B chromosome repeat in and around the centromere with a minor representative at the distal tip of the B long arm. Green signal identifies several chromosomal features, namely, the CentC centromeric satellite, 45S ribosomal DNA repeats, and TAG microsatellite clusters. DAPI stains the chromosomes (blue). (*B*) Schematic view of the acrocentric maize B chromosome at pachynema of meiosis. The chromosome is divided into the B short arm (BS), B centromere (BC), proximal heterochromatin (PH), proximal euchromatin (PE), four blocks of distal heterochromatin (DH1-4), and the distal euchromatin (DE). The B-specific repeat ZmBs, CentC satellite, CRM2 retrotransposon, knob heterochromatin, and TAG microsatellite cluster are color coded along the length of the chromosome. (*C*) Depiction of nondisjunction of the B chromosome. The B chromosome (blue with a red centromere) is shown in the generative nucleus (G) after the first microspore division. After replication, the two chromatids proceed to the same pole at the second microspore mitosis in the vast majority of divisions. Thus, most mature pollen grains contain two sperm (S) with only one containing the B chromosomes. V: vegetative cell. (*D*) Depiction of preferential fertilization. For most lines of maize, the sperm with the two B chromosomes will preferentially fertilize the egg (E) as compared with the central cell (C) in the process of double fertilization. The fertilized egg develops into the next generation embryo and the fertilized central cell develops into the endosperm. The combination of nondisjunction at the second pollen mitosis and preferential fertilization comprise the drive mechanism of the B chromosome.

The B chromosome of maize is one of the most thoroughly studied supernumerary chromosomes (9–11) (Fig. 1). It can be found in numerous landraces and also in populations of Mexican teosinte, the maize wild relative (12). Despite being dispensable, it is maintained in populations by two properties: nondisjunction at the second pollen mitosis giving rise to unequal sperm and then preferential fertilization of the egg by the B chromosome-containing sperm (4, 13) (Fig. 1). This acrocentric chromosome is smaller than standard A chromosomes. Its long arm comprises proximal (PE) and distal euchromatin (DE), proximal heterochromatin (PH), and four large distal blocks of heterochromatin (DH1-4) (Fig. 1). Its short arm is minute. In a majority of genetic backgrounds, the presence of B chromosomes is not detrimental unless at high copy number (10), but in some others will cause breakage at the second pollen mitosis of some A chromosomes that contain heterochromatic knobs (14). This effect is thought to be an extension of the B chromosome drive mechanism involved with nondisjunction at this particular mitosis. The B chromosome has also evolved the property of increasing recombination in heterochromatic regions in general, probably to ensure its own chiasmata for proper distribution in meiosis (7, 15–17). Further, it has acquired a mechanism for its faithful transmission as a univalent (18, 19), which would also enhance its transmission, given that it can sometimes be present in odd numbers. Thus, classical cytogenetic studies established multiple properties of this unusual chromosome that act to maintain it in populations, but the molecular basis of its non-Mendelian inheritance remained obscure. To date, the efforts to generate B-specific sequences in maize have been limited to DNA marker development, general characterization of repetitive sequences, and low-pass sequencing (20–23). While genomes of several maize lines were sequenced to reference quality (24–26), comparable information for the supernumerary chromosome has not yet been available.

## Results and Discussion

### Sequencing and Assembly of the Maize B Chromosome.

We sequenced the maize B chromosome introgressed into the B73 inbred line using a combination of chromosome flow sorting, Illumina sequencing, Bionano optical mapping, and high-throughput chromatin conformation capture (Hi-C). In total, 597 Gb of DNA sequence were generated (*SI Appendix*, Table S1). Initial assembly with De-NovoMAGIC software resulted in nearly 67,000 scaffolds representing 2.3 Gb (*SI Appendix*, Table S2), which exceeded the size of the assembled fraction of the B73 genome (without B) (24) by about 200 Mb. Hybrid scaffolding utilizing Bionano optical mapping and Hi-C analysis increased the N50 from 6.2-fold to 23.8 Mb producing a final assembly. Subsequently, 328 B chromosome-specific scaffolds longer than 10 kb were selected. The cumulative length (125.9 Mb) represents 89% of the predicted 141-Mb size of the B chromosome from flow cytometry (*SI Appendix*, Fig. S1 and Table S3).

### Verification of the B Assembly and Pseudomolecule Construction.

In the absence of genetic landmarks as guidance for the construction of the B chromosome pseudomolecule, we used lines of maize carrying only a portion of the chromosome in the form of B-A translocations (27), B centromere misdivisions (28, 29), and B chromosome breakage products (30–32) (*SI Appendix*, Figs. S2 and S3). We sequenced genomic DNA of those lines using Illumina technology to a low coverage, mapped reads to B chromosome-specific scaffolds, and determined the presence or absence of each scaffold in the segment of the B chromosome carried by a particular line (*SI Appendix*, Fig. S4). Using this approach, we successfully combined 21 scaffolds into a pseudomolecule of the B chromosome (chrB v1.0) with a total length of 106.6 Mb (Dataset S1). The pseudomolecule begins at the centromere and spans the bulk of the long arm of the chromosome. Thirteen scaffolds were assigned to the short arm of the chromosome by the deficiency mapping approach, of which 11 comprise only variants of the ZmBs repeat sequence, one contains both ZmBs and the centromeric retrotransoposon CRM1, and one additional scaffold with no predicted genes. The final B chromosome sequence is available at MaizeGDB (https://www.maizegdb.org/genome/assembly/Zm-B73_B_CHROMOSOME-MBSC-1.0).

### Annotation of Transposable Elements and Genes.

**Fig. 2.**
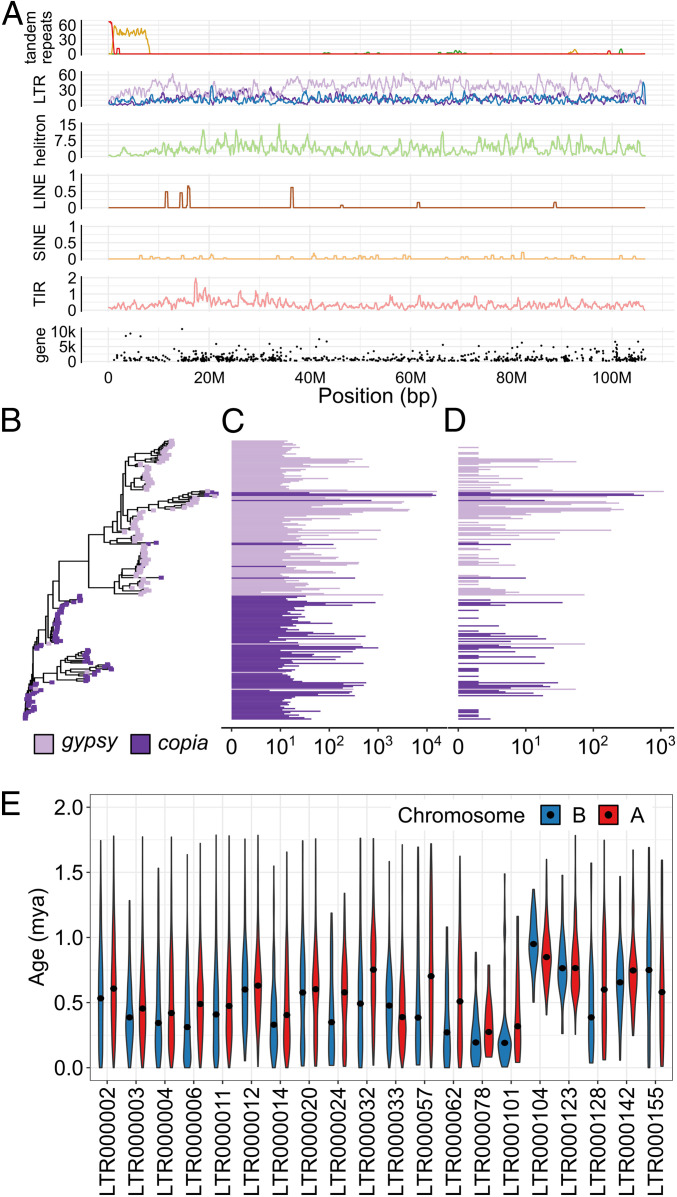
Repetitive sequences in the B chromosome. (*A*) Distribution of repetitive elements and genes along the maize B pseudomolecule. Tandem repeats are clustered mainly at the 5′ pseudomolecule end, i.e., the region close to the centromere: red, ZmBs; gold, 180-bp knob repeat; and dark green, CentC satellite. Transposable elements are evenly distributed along the B chromosome with the exception of the region occupied by tandem repeats. LTR-TEs: light violet, gypsy; dark purple, copia; and blue, unassigned. Occupancy by each class of elements (in percentage of sequence) is given per 500-kb window. For genes, the *y* axis represents the length of coding sequence. (*B*) Phylogenetic analysis based on the sequence of the reverse transcriptase domain of elements in both B and A chromosome(s). (*C*) Abundance analysis of elements of distinct families of LTR-TEs in A chromosomes and (*D*) the B chromosome sequence of maize (log10 scale). Only families with at least 10 elements were analyzed. Light violet, gypsy superfamily; dark purple, copia superfamily. (*E*) Estimated time since insertion of elements in the B (blue) and A (red) chromosome(s) in the 20 most abundant families. The age estimation is based on the sequence similarity of 5′ and 3′ LTRs of the respective element. Median values are marked by dots. Note similar age distribution for most families for A and B copies, indicating a shared history. Only elements of age below the 99th age percentile are visualized.

We performed comprehensive annotation to reveal the molecular organization of the B chromosome. We used multiple tools to assess the abundance of transposable elements (TEs) in the B chromosome yielding 15,145 distinct elements (Dataset S2). All TE types were equally dispersed along the B chromosome (Fig. 2) with the exception of the region close to the centromere (i.e., 5′ pseudomolecule end; see below for centromere definition), where the TEs are underrepresented and tandem repeats predominate. Most abundant are long terminal repeat (LTR) retrotransposons from the gypsy superfamily (Fig. 2). In total, TEs occupy 60% of the B chromosome length (*SI Appendix*, Table S4), which is comparable to their abundance in the maize A chromosomes (24). In order to determine the contribution of organellar sequences inserted into the B chromosome, we aligned our assembly against sequences of both organelles. This analysis identified a total of 712 kb organellar DNA in the assembled chrB v1.0 pseudomolecule, which represents about 0.6% of the B chromosome assembly. While the contribution of chloroplast sequences was small (67 kb), several insertions of mitochondrial DNA were recognized unambiguously. Mitochondrial sequences were located predominantly in the proximal euchromatin (PE), distal heterochromatin 3 (DH3), and distal euchromatin (DE) of the B chromosome (*SI Appendix*, Fig. S5). We confirmed these results by cytogenetic mapping using fluorescence in situ hybridization (FISH) (*SI Appendix*, Fig. S5).

For decades, B chromosomes were believed to be composed of repetitive DNA only. Recently, actively transcribed genes were reported in several studies (33–35). We annotated genes of the B chromosome using a modified Maker P pipeline and obtained a working set of 1,781 genes and 3,504 transcripts. After depletion of TE protein-encoding genes, we identified 758 genic sequences, each of them represented by the longest transcript in a filtered gene set (Fig. 2). There might be additional genes that encode long noncoding and microRNAs. To investigate the level of expression of the predicted genes on the B chromosome, we analyzed leaf transcriptomes of maize B73 inbred lines without B (0B), B73 + 1B (1B), and B73 + 6B (6B) (36). The reads from RNA sequencing (RNA-seq) from plants with 1B and 6B chromosomes mapped to 174 and 236 genes on the B chromosome with an average expression (reads per million [RPM]) of 0.47 and 2.84, respectively (Dataset S3). For comparison, the average expression of 15,574 genes localized on the standard A chromosomes is 12.68 RPM and is similar in all three samples because the A chromosomes are present in the same dose (Dataset S4). We found significant Log2-fold change (*P* value 0.05) for 26, 76, and 88 B chromosome genes between the transcriptomes from plants with 0B and 1B, 1B and 6B, and 0B and 6B chromosomes, respectively (Dataset S3). Further, we performed Gene Ontology (GO) analysis to investigate whether the B chromosome gene repertoire is enriched for genes linked to its peculiar behavior. At least one GO term was assigned to 568 genes in the filtered set (Dataset S5). Blast2GO enrichment analysis with B chromosome genes revealed 81 overrepresented GO terms in comparison to the maize A chromosomal complement at false discovery rate (FDR) threshold of 0.05. Among them, 28 GO terms were preserved as the most specific (*SI Appendix*, Table S5 and Fig. S6). Several GO terms overrepresented on the B that we have identified could be related to its drive. The most promising include “condensed nuclear chromosome, centromeric region” (GO:0000780); “spindle microtubule” (GO:0005876); “regulation of cytokinesis” (GO: 0032465); “histone-serine phosphorylation” (GO:0035404); and “chromosome segregation” (GO:0007059). As described below, the gene content of the B consists of randomly transposed genes from the A chromosomes, followed by generalized degradation for most genes, which suggests that the GO enrichment represents selection of genes involved in B chromosome maintenance.

### B Chromosome Centromere.

**Fig. 3.**
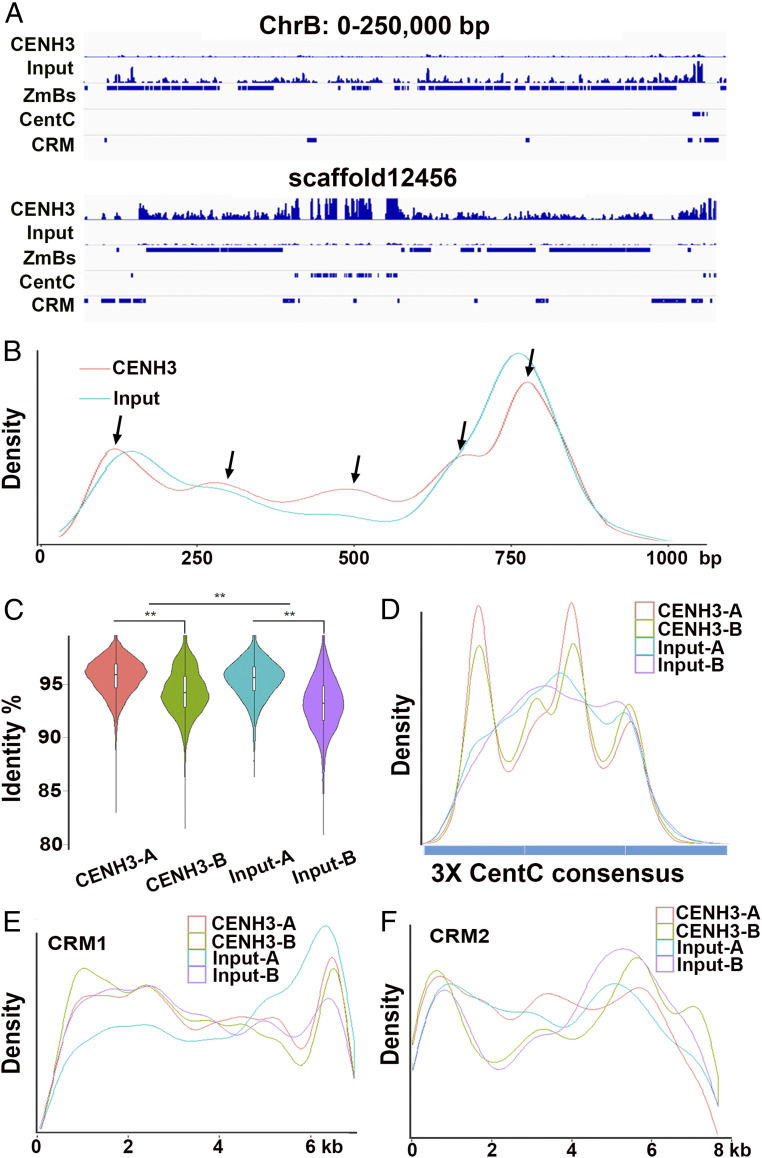
Characterization of the B chromosome centromere. (*A*) Snapshot of the distribution of centromere-specific repeats and CENH3 nucleosomes in the B chromosome pseudomolecule (chrB: 0 to 250,000 bp) and scaffold12456, the largest in the centromeric region. This scaffold maps between the breakpoints of the centromere misdivisions of miniB876 and isoTB-6Lc (Dataset S9). The horizontal scale marks the centromere locus in kilobases. The tracks from the *Top* to the *Bottom* are: CENH3-ChIP-seq; input; B-specific repeat ZmBs; centromeric satellite CentC; centromeric retrotransposon CRM. (*B*) Midpoint positions of CENH3-ChIP-seq (red) and input (blue) reads along the ZmB sequences. Two major and three minor CENH3 nucleosome positions (arrows) are indicated by alignment of the CENH3-ChIP-seq reads. Only two major CENH3 nucleosome positions are observed in the input reads. (*C*) Characterization of CentC satellite repeats in maize. Violin plot of the sequence identity to the CentC consensus sequence as sampled in the maize A and B chromosomes by the CENH3-ChIP-seq and input reads. **P < 0.001 (two-tailed Student’s *t* test). The greater difference between the violin plots of CENH3-B versus input-B compared to CENH3-A versus input-A indicates that the CentC in the B centromere is more similar to those in the A centromeres than the dispersed CentC copies in the B long arm. (*D*) Distribution of the midpoint positions of CENH3-ChIP-seq and input reads from the maize A and B chromosomes along the trimer of CentC satellite consensus sequence. (*E* and *F*) Distribution of the midpoint positions of CENH3-ChIP-seq and input reads from maize A and B chromosomes along the CRM1 (*E*) and CRM2 (*F*) sequence.

The pseudomolecule starts at the centromere of the B chromosome; its tiny short arm remains fragmented in scaffolds not in the pseudomolecule. The centromere was identified using a combination of deficiency mapping, presence of centromeric repeats, and association with the centromere-specific histone CENH3, a component of functional centromeric chromatin. In total, these sequences represent ∼574 kb, which is close to the previous cytological estimate (29). A major fraction of this sequence is comprised of the CentC satellite and the Centromere Retrotransposons of Maize (CRM), which are also typical of the centromeres of A chromosomes. We identified functional components of the B chromosome centromere using CENH3-Chromatin Immunoprecipitation Sequencing (CENH3-ChIP-seq) data (37). The analysis showed that midpoint positions of CENH3 nucleosomes on CentC repeats in the B chromosome did not differ from those on A chromosomes (Fig. 3), indicating that there is no difference in the loading of CENH3. Similarly, no significant differences were observed in the positions of CENH3 nucleosomes on CRM elements between A and B chromosomes (Fig. 3). In agreement with previous cytogenetic observations (38), clusters of CentC units are also interspersed on the long arm of the B, but do not associate with CENH3. Further, the CentC copies in the centromere are similar to those in the A centromeres but those in the long arm are more divergent (Fig. 3). In addition, we identified two major and three minor peaks of CENH3 nucleosome positions in the monomer of ZmBs. Altogether, these observations indicate that the B chromosome centromere has a structure and function similar to that of the A chromosomes.

### Elements of B Chromosome Drive.

**Fig. 4.**
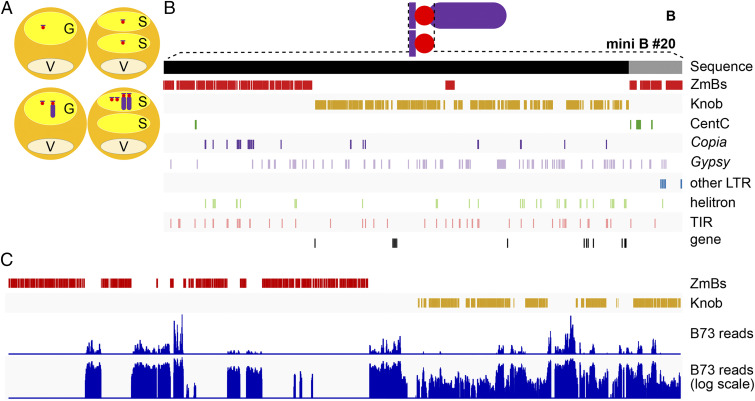
Region of the *cis* factor for nondisjunction. (*A*) Mini B#20 disjoins regularly itself during the second pollen mitosis. However, in the presence of complete B chromosomes, it undergoes nondisjunction as well, indicating the presence of the *cis* factor in the sequence of mini B#20 (41). (*B*) Analysis of the sequence composition of mini B#20 revealed 10 genes and a prevalence of repetitive elements in and around the B centromere. The black line corresponds to the beginning of B chromosome pseudomolecule (chrB v1.0); the gray part represents additional unordered scaffolds belonging to the sequence of mini B#20. The major components of mini B#20 are tandem arrays of ZmBs (red) and 180-bp knob repeats (gold) in the pericentromeric region, which are respectively displayed in the second and third tracks. The following tracks present the CentC satellite (dark green); LTR-TE, copia (dark purple), gypsy (light violet), unassigned (blue); helitron (light green); and TIR (pink). The final track shows the position of the genes. (*C*) Enlargement of the B chromosome pseudomolecule region (at position 900 to 1,300 kb) containing the border between the segment covered by ZmBs (red) and the 180-bp knob units (gold). The coverage of the region by reads originating from the B73 A chromosomal complement indicates homology of all the sequences but the ZmBs repetitive arrays, illustrating its specificity to the B chromosome. The *Upper* blue track shows a histogram of mapping density for reads representing 20x coverage of the B73 genome mapped to mini B#20 (with maximum of coverage 61,707). The *Lower* blue track shows the same data in a log scale.

One of the components of the drive mechanism is that the B chromosome regularly undergoes nondisjunction at the second pollen mitosis. During this division, sister chromatids do not separate at the centromeric region. We refer to the factor required for the adhesion of sister chromatids as a *cis* factor for nondisjunction. The adhesion is further dependent on *trans*-acting factors on the B chromosome long arm; one of them at the very distal tip (*trans* factor #1) (39, 40) and another in the proximal euchromatin region (41). Among the B-deficiency-carrying lines we used, one comprises a mini B chromosome #20. Because mini B#20 undergoes nondisjunction when supplied with the *trans*-acting factors in a full-sized B chromosome (31, 42) (Fig. 4), it must contain the *cis* factor.

We identified 4 Mb of sequence representing mini B#20 and showed that it is composed mainly of the centromere region and the ZmBs repeats, a repetitive unit unique to the B chromosome and the heterochromatin 180-bp knob repeat (Fig. 4). These two repeat types are related on the sequence level (23). Further, 12 genes were found in this particular region.

Evidence from previous genetic studies of nondisjunction in centromeric aberrations and their molecular analysis, coupled with the sequence data presented here, provide insight into the *cis* factor. W. R. Carlson recovered a pseudoisochromosome from TB-9Sb (43) that contains one arm similar to the original B long arm but the other arm joined a break in the centromere with a region in the proximal euchromatin. This chromosome was capable of nondisjunction and preferential fertilization. From the pseudoisochromosome, he further recovered telocentric chromosomes from centromere misdivision that consisted of either arm of the original. The telocentric with the normal B long arm containing the proximal heterochromatin had near normal levels of nondisjunction. However, the telocentric chromosome from the other arm that is missing the proximal heterochromatin, including the knob region, had extremely low levels of nondisjunction. Nevertheless, the frequency of nondisjunction of this chromosome could be increased in some genetic configurations, indicating a retention of the ability to undergo nondisjunction (44). Indeed, when an isochromosome from an additional centromere misdivision was recovered from the latter telocentric chromosome, the frequency of nondisjunction increased substantially but did not achieve normal levels. Thus, the knob region can be eliminated as the sole determinant of nondisjunction because chromosomes missing it altogether are still capable of nondisjunction to some degree. This conclusion is further supported by the finding that an inactive B centromere, which is incapable of organizing a kinetochore and is translocated to the tip of chromosome arm 9S, has very little knob repeat remaining (*SI Appendix*, Figs. S2 and S4), but is capable of attempting or achieving nondisjunction of chromosome 9 in the presence of normal B chromosomes that supply the *trans*-acting factors (45). The fact that centromere misdivision derivatives, or their further derivatives that eliminate one side or the other region adjacent to the centromere, are capable of nondisjunction, makes the involvement of single copy genes highly unlikely.

Furthermore, the copy number of the B-specific repeat in and around the centromere was quantified in all of the mentioned misdivision chromosomes (28). The quantity of the B repeat number is related to the frequency of nondisjunction of the respective chromosomes. Collectively, the results indicate that the *cis* factor for nondisjunction is divisible and therefore repetitive as well as dependent on the copy number of the B repeat.

As mentioned above, apart from the functional centromere, the mini B#20 consists of tandem repeats of ZmBs interspersed in and around the active centromere together with a distal block of 180-bp knob repeats (Fig. 4). We further show that the ZmB repetitive arrays are the only sequences unique to the mini B#20 chromosome (Fig. 4) and that the 180-bp knob repeats are common on the A chromosomes, which do not undergo nondisjunction. Together with the previous findings noted above that the chromosomes lacking the knob are still capable of nondisjunction (28, 43, 44), those results suggest that the B chromosome-specific ZmB repeats act as the *cis* factor mediating nondisjunction. The collective data suggest that the B repeat unit has become concentrated in and around the B centromere and confers upon it in a quantitative manner the *cis* component of the chromosomal drive mechanism.

### *Trans*-Acting Factor for Nondisjunction.

The very tip of the long arm of the B chromosome has been shown to be required to be in the same nucleus as the centromere in order for nondisjunction to occur (13, 17, 46). It need not be present on the same chromosome and thus acts as a *trans* factor. Previous analysis showed that the B-3Sb chromosome, which lacks only a tiny distal segment of the B chromosome, fails to nondisjoin itself at the second pollen mitosis (46). Using deficiency mapping, we identified the missing part of B-3Sb with a size of about 2.7 Mb. We identified 34 predicted protein-coding genes in this region as candidates for *trans* factor #1 (*SI Appendix*, Table S6). It is also possible that the *trans*-acting effect in this region is not due to a protein-encoding gene.

### Preferential Fertilization.

In addition to nondisjunction, preferential fertilization, in which the sperm with B chromosomes preferentially fertilizes the egg at a higher frequency than the polar nuclei in the process of double fertilization, is the second major component of the maize B chromosome drive mechanism. Together these two processes are necessary for the enhanced frequency of B chromosome transmission from one generation to the next. Various translocations between the B chromosome and one of the A chromosomes (27) retain the property of preferential fertilization and can be used to delimit the factor underlying it. With these translocations, the B-A chromosome, which has the B centromere, is the chromosome that is different between the two sperm following nondisjunction, while the A-B chromosome is present in both sperm. Thus, the centromere proximal region of the chromosome must contain the region responsible for preferential fertilization. We showed that the B-8Lc chromosome (*SI Appendix*, Fig. S2) has the smallest segment of B chromosome among them consisting essentially of the (peri)centromeric region (*SI Appendix*, Fig. S4). It is represented by 8.7 Mb in our assembly and 29 predicted genes were identified in the region (Zm00044a000001 to Zm00044a000029; see Dataset S5 for additional information about genes). Preferential fertilization can be affected by the female parent (47), so it is more difficult to assign a responsible region than for nondisjunction. Nevertheless, it is clear that an involvement of a region in close proximity to the *cis* factor for nondisjunction, if not identical to it, ensures that the two components of the drive mechanism will seldom, if ever, be separated by recombination in the B chromosome, which is known to occur (48).

### Evolutionary History of the B Chromosome.

To uncover the B chromosome evolutionary history, we first performed comparative analysis of transposable elements in the B chromosome and A chromosomal complement. We clustered LTR-TEs based on 5′ LTR similarity and identified 42 distinct families that have at least 10 members on the B chromosome (the largest of them containing 1,104 members). While 16 families are overrepresented on the B chromosome compared to the A chromosomes (Dataset S6), only two families were found exclusively on the B chromosome having 25 and 11 members. LTR-TE phylogeny analysis based on sequence similarity of the reverse transcriptase region revealed two distinct populations of Gypsy and Copia superfamilies (Fig. 2). The estimated age of LTR-TEs based on LTR divergence follows a Poisson distribution with a median value of 0.46 million years ago and with 95% of the elements younger than 1.42 million years. While we found a significant variation among the age of LTR-TE families suggesting different activation histories, little difference in insertion time was found among B and A chromosomal copies within individual families (Fig. 2). We conclude that insertions of TEs in the genome with B chromosomes are randomly distributed within the chromatin of A and B chromosomes and the current TEs of the B chromosome are coincident with the A chromosomal complement.

**Fig. 5.**
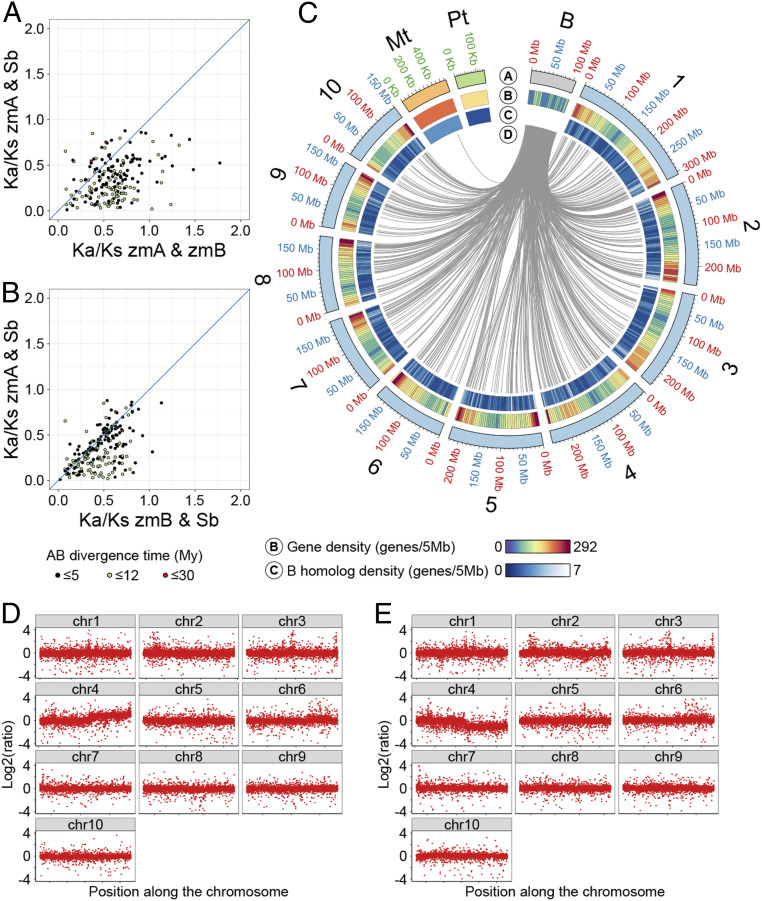
Evolution of the B chromosome sequence. (*A* and *B*) Comparison of genes with homologs in the maize and *S. bicolor* genomes. Comparison of *K*_a_/*K*_s_ for homologs in maize A chromosomal complement and *S. bicolor* (*y* axis) vs. *K*_a_/*K*_s_ for homologs in the maize B chromosome and maize A chromosomal complement (*A*), and vs. *K*_a_/*K*_s_ for homologs in the maize B chromosome and *S. bicolor* genome (*B*). Color corresponds to the estimated divergence time of the B gene and the A counterpart. Note the global increase of *K*_a_/*K*_s_ when the B chromosome genes are involved (*x* axis), which indicates a relaxation of purifying selection. (*C*) Comparison of the maize B chromosome and the B73 A chromosomal complement. Track A: chromosomes and organellar DNA; magnification of the mitochondrial and chloroplastic DNA by 200x and 500x, respectively. Track B: gene density in 5-Mb windows. Track C: density of paralogs of B chromosome genes in 5-Mb windows. Track D: link between B and A paralogs with mitochondrial gene links colored in orange. (*D* and *E*) Copy number analysis in disomy or monosomy of TB-4Lb, a translocation between the B chromosome and the long arm of chromosome 4, and haploid controls using the read counts from gene regions. For chromosome 4, TB-4Lb disomy contains two copies of the long arm of chromosome 4 (4, B-4Lb) in an otherwise haploid plant and most of the B chromosome. TB-4Lb monosomy has the chromosome constitution of one copy each of 4Lb-B and chromosome 4 together with the addition of the distal tip of the B long arm. The disomy and monosomy of TB-4Lb show a two-fold higher and lower copy number variation (CNV) on 4L, respectively. No large region with CNV was found on other chromosomes that might result from homology to the portions of the B chromosome present.

We also performed phylogenetic analysis of B chromosome genes with their homologs in the A chromosome complement and related species. We compared transcripts of the filtered B chromosome gene set to *Brachypodium distachyon*, *Oryza sativa*, *Sorghum bicolor*, and *Zea mays* coding sequences (CDSs). A total of 213 B chromosome genes with homologs in all related genomes were investigated in more detail (Dataset S7). We estimated the divergence time between A and B chromosome gene copies in a range from 0.24 to 12.5 million years (Dataset S8). The results indicate a continuous and gradual introduction of new genes into the B chromosome. Comparison of *K*_a_/*K*_s_ among homologs in the maize B chromosome, the maize A chromosomes, and sorghum revealed that genes on the maize B chromosome are accumulating more nonsynonymous mutations in general than genes in the sorghum genome and maize A chromosomal complement (Fig. 5), suggesting a relaxed purifying selection for B-encoded genes. Further, we show that homologs of B chromosome genes are widely dispersed on all A chromosomes (Fig. 5).

In addition, an analysis of copy number reads for disomic haploid and monosomic diploid B-A translocations, similar in principle to the deficiency mapping but instead documenting regions of increased sequencing reads, indicates that no region of the maize genome has a recognizable syntenic region with high homology to the B chromosome (Fig. 5) The sequence read copy number for the translocated A chromosomal regions illustrates that two-fold changes with the copy number of regions present can be recognized but the additional B chromosomal regions in the genotypes show no similar increase on any A chromosome or segment. It should also be noted that whole chromosome paints for each of the 10 maize chromosomes fail to detect any homology on the B chromosome (49). Together, these data indicate that the gene sequences present in any potential progenitor genomic region that gave rise initially to the B chromosome have degenerated beyond recognition. Because preservation of synteny has been observed over millions of years in the grass family for core genes (50), this result indicates that in the absence of purifying selection, the original gene repertoire has deteriorated. The diversity of the divergence times we estimated for paralogs on A and B chromosomes and the inability to recognize a progenitor synteny suggest that the current gene content of the B is a result of gradual transposition to this chromosome over time, indicating that the drive mechanism has propelled this degenerate chromosome through millions of years of evolution.

## Concluding Remarks

The sequence of the supernumerary B chromosome of maize has narrowed the site for nondisjunction to the centromere and strongly suggests that the *cis* factor for nondisjunction is the B-specific repeat that is interspersed in and around the centromere. It is related to heterochromatic knob repeats (23) that have delayed replication in the cell cycle (51–53), which could potentially explain the failure of the sister chromatids of the B chromosome to separate at the second pollen mitosis. The localization of the breakpoints of B-A translocations on the B sequence also allowed the delineation of a region involved with preferential fertilization that is in close proximity to the centromere, if not coincident with it.

The GO term analysis suggests an overrepresentation of gene functions that could function in the maintenance of the B chromosome. As noted above, genetic experiments have defined at least two regions of the chromosome that are needed for nondisjunction of the centromeric region to occur and these operate in *trans*, suggesting a diffusible product. The most distally broken B-A translocation defines the region of the sequence that is implicated in nondisjunction. There are 34 predicted protein-encoding genes in this region but it is also possible that an RNA encoded therein is responsible.

From multiple lines of evidence, we failed to identify a region in the A chromosomes that has a syntenic region of homologous genes that might indicate a chromosome or chromosomal fragment that could have been a progenitor to the B chromosome. In contrast, transposable elements between the B and the A chromosomes show a similar spectrum. Together these two results suggest that the B chromosome predates the most ancient expansion of particular transposable elements that can be recognized but there is a lack of any identifiable syntenic gene sequences due to the absence of selection, given that the chromosome is dispensable. A survey sequence of the rye B chromosome was able to identify blocks of synteny in the barley genome (54), indicating that the maize B is much older, which is consistent with the fact that detrimental effects of the maize B emerge only at much higher copy number than such effects of the rye B (10, 55).

The lesser detrimental effects of the maize B suggests that it is generally devoid of dosage-sensitive genes. In vertebrates, three different homologous chromosome pairs have evolved into heteromorphic sex chromosomes with the loss of many genes (56). However, dosage-sensitive genes are retained between the chromosome pair, illustrating selection against a two-fold change in dosage. In the plant kingdom, genome fractionation following whole genome duplication shows a similar retention of dosage-sensitive genes (57). The nonvital nature of the B chromosome and the tolerance of many copies (Fig. 1) suggest that the B is generally lacking dosage-sensitive genes and that the transposition of them to the B is selected against. Thus, only genes that foster the perpetuation of the B itself would be retained with the degeneration and loss of others.

The genes that are detected on the B chromosome have a wide range of sequence divergences with their paralogs on the A chromosomes, which are dispersed across the genome. A comparison of these gene pairs indicates that the copies on the B chromosome have relaxed purifying selection as a group. While the majority of genes transposed to the B chromosome go there to die, the others have the opportunity to evolve specific new functions as duplicate genes to foster the perpetuation of the B chromosome with regard to nondisjunction (13), preferential fertilization (4), stabilizing the B chromosome transmission as a univalent (18, 19), increasing recombination in meiosis across the genome in heterochromatic regions to foster its own transmission (7, 16, 40), and modulating gene expression on other chromosomes (36), which are all properties to ensure the transmission of this chromosome despite being dispensable. The B sequence reported here provides a reference to investigate these properties.

## Materials and Methods

### Plant Material.

Plants of *Z. mays* (L.) inbred line B73 containing various numbers of B chromosomes were used for the majority of the experiments. Plants with two B chromosomes were used for chromosome sorting, with 6, 8, 10, and 12 chromosomes for estimation of B chromosome size, and with 10 to 15 B chromosomes, were further used for whole genome sequencing and Bionano optical mapping and Hi-C. Various lines with B chromosome deficiencies and translocations (*SI Appendix*, Fig. S2) were used for deficiency mapping to support construction of the B chromosome pseudomolecule and misassembly corrections. The 9-B inactivated centromere-1 (9-Bic-1) chromosome was produced when an inactive B centromere from TB-9Sb was translocated there in material undergoing the breakage-fusion-bridge (BFB) cycle for that B-A translocation (30, 58). Mini B9 and B20 were derived from a BFB experiment involving TB-9Sb with a foldback duplication (31, 32, 42). Mini B140, 533, 599, 876, 881, and 1,120 were produced from B chromosome breakage in material of 9-Bic-1 that was monitored for the generation of minichromosomes in the presence of normal B’s. Isochromosome B long arm was generated from a B chromosome centromere misdivision (28). Isochromosome TB-6Lc was found upon identifying TB-6Lc disomy (A, BA). The B-A translocation lines were generated and characterized by multiple laboratories (27). The tertiary trisomies (A, A, BA) of TB-5Sc and TB-10L18 were from a cross between female hyperploid heterozygotes (A, AB, BA, and BA) and their respective tester lines. When TB-9Sb, TB-6Lc, and TB-4Lb hyperploid heterozygotes were crossed as males with genetic marker tester lines, the monosomic (A, AB) kernels will show colorless embryo and colored endosperm. For disomy (A, BA), the hyperploid heterozygotes were crossed as female by the haploid inducer line RWSK (*R1-navajo* [*R1-nj*]; *C1-I*) or RWS (GFP; *R1-nj*) (59). Haploid kernels will show colored embryo and colorless endosperm when crossed by RWSK (*R1-nj*; *C-I*). If crossed by RWS (GFP; *R1-nj*), roots of haploid seedlings will show an absence of GFP signal. FISH identification confirmed the chromosome constitution of each B chromosome deficiency (*SI Appendix*, Fig. S3).

### De-NovoMAGIC Assembly, Hybrid Bionano Assembly, and Hi-C Scaffolding.

Genomic DNA was isolated from leaves of inbred line B73 plus 10 to 15 B chromosomes using Qiagen DNeasy Plant Mini Kit (Qiagen). Five size-selected genomic DNA libraries ranging from 470 bp to 10 kb were constructed and sequenced. Two shotgun libraries were prepared using fragments of ∼470 bp and 800 bp and the TruSeq DNA Library Preparation Kit v2 with no PCR amplification (PCR-free) according to the manufacturer’s protocol (Illumina). To increase sequence diversity and genome coverage, three separate mate-pair (MP) libraries were constructed with 2- to 5-kb, 5- to 7-kb, and 7- to 10-kb jumps using the Illumina Nextera Mate-Pair Sample Preparation Kit (Illumina). The fragment size 470 bp was designed to produce a sequencing overlap of the fragments after sequencing on the Hiseq2500 in rapid mode as 2 × 265 bp, thus creating an opportunity to produce “stitched” reads up to 520 bp in length. The 800-bp shotgun library and the MP libraries were sequenced on an Illumina HiSeq2500 as 2 × 160 bp reads (using the v4 Illumina chemistry). Raw data were submitted to the National Center for Biotechnology Information (NCBI)-Sequence Read Archive (SRA) as BioProject PRJNA633287.

Genome assembly was conducted using De-NovoMAGIC software v2.0 platform (NRGene), a De Bruijn graph-based assembler, designed to efficiently extract the underlying information in the raw reads to solve the complexity of the De Bruijn graph due to genome polyploidy, heterozygosity, and repetitiveness. This task is accomplished using accurate-reads-based traveling in the graph that iteratively connected consecutive phased contigs over local repeats to generate long phased scaffolds (60–65). In brief, the algorithm is composed of the following steps: 1) Preprocessing of reads; PCR duplicates, Illumina adaptor AGATCGGAAGAGC, and Nextera linkers (for mate-pair libraries) were removed. The pair-end (2 × 265 bp) overlapping reads of the 450-bp library were merged with minimal required overlap of 10 bp to create the stitched reads. 2) Error correction; following preprocessing, merged pair-end reads were scanned to detect and filter reads with putative sequencing error (containing a subsequence that does not reappear at least four times in other reads). 3) Contigs assembly; the first step of the assembly consisted of building a De Bruijn graph (kmer = 127 bp) of contigs from the all pair-end and mate-pair reads. Next, pair-end reads were used to find reliable paths in the graph between contigs for repeat resolving and contigs extension. 4) Scaffold assembly; contigs were linked into scaffolds with pair-end and mate-pair information, estimating gaps between the contigs according to the distance of pair-end and mate-pair links. 5) Gap filling; a final gap-filling step used pair-end and mate-pair links and De Bruijn graph information to detect a unique path connecting the gap edges.

High molecular weight DNA was prepared with the Bionano IrysPrep Plant Tissue DNA Isolation Kit (RE-014-05; Bionano) from a pool of seven seedlings from a B73 derivative with 10 to 15 B chromosomes. The DNA was labeled with Nt.BspQI using the IrysPrep Nick, Label, Repair, and Stain labeling kit (RE-012-10; Bionano). A total of 186 Gb filtered data (>150 kb) were collected on a single Bionano IrysChip with an average molecule length of 259 kb. Molecules that aligned to the B73 genome were first removed. The remaining 11 Gb (∼37.3 × coverage of the B chromosome) were assembled with IrysView software (version 2.5.1) set to “optArgument_human.” Bionano Solve software was then used to create hybrid scaffolds using the assembled cmaps and NRgene sequence assembly.

For the final assembly improvement, Hi-C technology was used (66). Nuclei from young leaves of a B73+10B plant were isolated and proximity ligation was performed using the Dovetail Hi-C Kit (Dovetail Genomics). A sequencing library was subsequently prepared with Dovetail Library Module for Illumina (Dovetail Genomics). Scaffolding was performed using HiRise (Dovetail Genomics) with Hi-C linked reads and Bionano-hybrid scaffolds as input.

### Comparison of Assembly to the Maize B73 Genome and Identification of B-Specific Scaffolds.

Assembled final scaffolds were assigned to the A chromosomal complement and B chromosome as follows: 1) Scaffolds with length ≥10 kb were aligned to pseudomolecules of the 10 chromosomes of the B73 inbred line (24) using nucmer (67) (*SI Appendix*, Fig. S7 and Table S7). 2) Scaffolds not mapping to any A chromosome were checked for a B-specific signature using a Kmer profiling. Suffix array was created with suffixerator, a part of genome tools v1.5.1 (68) for sorted B chromosome reads equivalent to 20× and B73 genome (with parameters -tis -suf -lcp -des -ssp sds dna). A 49-mer index for each suffix array was then created using tallymer mkdindex (with parameter -minocc 1 -counts -pl). Finally, each scaffold was profiled using tallymer indexes with tallymer search (with parameters -output qseqnum qpos counts). Profile with B73 index was used to identify repetitive regions along the scaffolds and profile with 20× B chromosome data was used to assign scaffolds to the B chromosome when nonrepetitive regions were covered (*SI Appendix*, Fig. S8).

### Deficiency Mapping and Pseudomolecule Construction.

Genomic DNA extraction of various lines with B chromosome deficiencies and translocations was performed using Qiagen DNeasy Plant Mini Kit (Qiagen). DNA libraries were prepared with a TruSeq genomic DNA library preparation kit with no PCR amplification and Illumina single end sequencing was performed with 75-bp length reads on an Illumina NextSEq 500 (service was provided by the DNA Core at University of Missouri; raw data were submitted to NCBI-SRA as BioProject PRJNA634743). The DNA sequencing data for each of the B chromosome deficiencies shown in *SI Appendix*, Fig. S2 were trimmed at the 3′ end of the sequences for ambiguous nucleotides (Ns) and for artificial poly Gs using cutadapt (69). Roughly, 6× coverage trimmed reads were aligned to the maize W22 reference genome (Zm-W22-REFERENCE-NRGENE-2.0) (25) or B73 reference genome plus mitochondria and chloroplast genomes (24) using Bowtie2 (70). Specifically, the B73, 9-Bic-1, and mini chromosomes that were produced from B chromosome breakage during the identification of 9-Bic-1 derivatives were mapped to B73 plus organellar genomes. The remaining lines were aligned to the W22 genome. The threshold of maximum number of distinct alignments was set to 10. MULTICOM-MAP (71) was used to remove the reads mapped to a unique location on the maize W22 or B73 sequences with at most two mismatches. The remaining reads were aligned to the B chromosome assembly using Bowtie2. Only reads that mapped to a unique location with no mismatch were kept for calculation of scaffold coverage. The reads were counted in the regions of 1 kb along each sequence and the results were plotted by ggplot2 in R (72). Scaffolds were oriented and combined into a pseudomolecule based on the location of the translocation breakpoints. Short scaffolds, which cannot be placed and oriented unambiguously, were not used for pseudomolecule construction; however, they were assigned to a particular chromosomal segment defined by two adjacent breakpoints (Dataset S9). Finally, the pseudomolecule was profiled with the reads in the same way as for the scaffolds to verify its assembly (*SI Appendix*, Fig. S4). The B chromosome sequence is available at MaizeGDB under the name Zm-B73_B-CHROMOSOME-MBSC-1.0 with the identifier Zm00044a.

### Gene Annotation.

Annotation of the B chromosome was performed using the MAKER-P pipeline as described previously (24). The annotation was split into distinct steps. First, RepeatMasker v4.0.7 (73) was used to mask repetitive sequences using maize transposable elements (74). Afterward, genes were ab initio predicted using AUGUSTUS v2.5.5 (75) and FGENESH v8.0.0a (SoftBerry) with maize and monocot matrices, respectively. To support gene identification, the pipeline utilized results of a similarity search between repeat-masked sequence and protein sequences annotated in related genomes and available information for the maize transcriptome. Proteins of *Arabidopsis thaliana*, *B. distachyon*, *O. sativa*, *S. bicolor*, and *Setaria italica* were retrieved from the Ensembl Plants release 43 (76). Maize full-length cDNA (77), isoSEq (78) and maize RNA-seq reads (79) that have been assembled with Trinity (80) were downloaded from GenBankMaizeCode_annotation_evidence_data_2017 (Cold Spring Harbor Laboratory, 2017). In addition to the databases used by Jiao et al. (24), Trinity v2.6.6 was used to assembly RNA-seq data from leaves of B73 and B chromosome(s)-possessing lines (B73+1B and B73+6B) (36) and data were implemented in the pipeline. Further, RNA-seq data from additional lines were generated, assembled, and used for gene prediction (*SI Appendix*, Table S8). In order to keep the annotation process as similar as possible to the annotation of the maize B73 sequence v4, protein and cDNA from the maize B73 v3 were also used as input for gene annotation. Finally, predicted transcripts were then filtered for transposable elements using BLAST and the Plant Genome and Systems Biology (PGSB) TE database 9.3 for Poaceae (81). All transcripts with hits that have identity percentage superior or equal to 70% and a coverage percentage superior or equal to 50% of the sequence length were removed from the annotation. Finally, the longest transcript for each gene was retained as a representative gene model. In order to check the quality of our annotation, we have reannotated chromosome 1 from the maize B73 v4 with our pipeline, revealing comparable results (*SI Appendix*, Fig. S9).

### Functional Gene Annotation and GO Enrichment Analysis.

To determine potential homologs of identified genes, a BLAST search (v2.9.0) (82) of translated protein sequences was performed against the NCBI nonredundant protein database restricted for Viridiplantae accessions (txid 33090). InterProScan v5.36-75.055 was used to identify protein domains and families. GO terms for each gene were retrieved from the BLAST search and InterProScan results, merged, and validated using Blast2GO v5.2.556. Protein descriptions were retrieved from BLAST search results using Blast2GO module Blast Description Annotator (BDA). An identical pipeline was repeated for the B73 RefGen_v4 maize reference annotation. GO enrichment analysis was performed with Blast2GO using annotated genes on the B chromosome as a test set and genes on the A chromosomal complement as a reference set and tested using the one-sided Fisher’s exact test with correction via FDR and *P* value threshold of 0.05.

### Phylogenetic Analysis and Estimation of Gene Duplication Time.

Gene model transcripts were compared to *B. distachyon*, *O. sativa*, *S. bicolor*, and *Z. mays* (Bd, Os, Sb, and Zm, respectively) CDSs retrieved from the Ensembl Plants release 43 (76) by reciprocal best BLAST hits (82). Only genes having orthologs in each species were considered in the following analysis (Dataset S7). Each set of five genes (copies in the sequence of the maize B, Bd, Os, Sb, and Zm) was multiple aligned using MUSCLE v3.8.1551 (83). The alignments were converted in SeaView v4 (84) to PHYLIP format required by the subsequent tools. The alignments served afterward as input to PartitionFinder v2.1.1 (85), which defined the best evolution model for each gene set using a Bayesian information criterion (BIC) model with optimization for BEAST software as model choice parameters in addition to the greedy algorithm. Phylogenetic trees and divergence times were calculated in BEAST v1.8.4 (86, 87). The input files were generated using the BEASTGen package (https://beast.community/beastgen) providing alignment from MUSCLE and the model from PartitionFinder as source data. Further, additional taxa were constructed to guide the tree topology: 1) Poaceae, comprising genomes of all five species selected for phylogenetic analysis; 2) BED clade, comprising *B. distachyon* and *O. sativa* (set as monophyletic); and 3) Andropogoneae, comprising *Z. mays* and *S. bicolor*. The B chromosome gene copy was allowed to cluster freely in the tree. Divergence times were set to 55 ± 5 MYA for Poaceae, 46 ± 1 MYA for BED clade, and 12 ± 1 MYA for Andropogoneae, following a normal distribution (88). A consensus tree was produced using TreeAnnotator with the output trees from BEAST after burning the hundred first trees. The final tree was then produced using R and the packages treeio, ggplot2, ggtree, ape, and tidytree (72, 89–92). Resulting trees were used to retrieve information about the time of divergence between homologs in the B chromosome and maize A chromosomal complement sequence for each gene set. In order to compute *K*_a_/*K*_s_ ratios between B-specific and A chromosomal homologs, B-specific and *S. bicolor* homologs, and between maize A chromosomal and *S. bicolor* homologs, protein-guided nucleic alignments were generated using the R package seqinr (93). Subsequently the *K*_a_/*K*_s_ was calculated for each pair of genes with KaKs_Calculator v2.0 and the Model Averaging method (94). *K*_a_/*K*_s_ analysis was performed for gene sets with an estimate of divergence time between B chromosomal and A chromosomal homologs with the 95% highest posterior density interval ≤10 million years, only.

### Identification and Characterization of Centromeric Satellite Repeats and Retrotransposons.

All individual units of B-specific repetitive sequence ZmBs (23), CentC satellite sequence (95), and centromeric retrotransposons CRM1 and CRM2 (96) (accession nos. AC116034.3 and AY129008.1) and the respective sequence identities to consensus were retrieved via BLAST homology search (v2.8.1+) (82). The positions of identified repeats were converted to bed format and displayed and evaluated in Integrative Genome Viewer v2.3.69 (IGV) (97).

The CENH3-ChIP-seq datasets derived from TB-9Sb materials generated by Liu et al. (37) were downloaded from Gene Expression Omnibus (GEO) (98) database (accession no. GSE59124). The paired-end reads were mapped to the B73 v4 genome (24) supplemented with B chromosome assembly using bwa v0.7.13 with mem algorithm and default parameters (99). The mapped reads with quality over 20 were treated as unique reads and used for further analysis (100). The alignment results were converted to bed format and CENH3-ChIP-seq enrichment was calculated with bedtools v2.25.0 (101). The ChIP-seq results were displayed and evaluated in Integrative Genome Viewer software (97).

To further characterize nucleosome positions in the centromere sequences, the paired-end reads from CENH3-ChIP-seq datasets (see above) and Input-seq were merged using SeqPrep software with default parameters (https://github.com/jstjohn/SeqPrep). The Input-seq was generated from purified nuclei of young leaves of the B73 line possessing two B chromosomes digested with MNase as described previously (102). The MNase-digested DNA sample was sequenced using the Illumina platform to generate pair-ended 150-bp sequence reads (data were submitted to NCBI-Gene Expression Omnibus [GEO] under accession GSE152074). The joined reads were aligned to ZmBs, trimer of CentC satellite sequence, and CRM1 and CRM2 centromeric retrotransposons using bwa v0.7.13 with mem algorithm and default parameters (99), and the nucleosome midpoint position plots were generated according to Su et al. (103). Data processing and analysis were performed using Perl, and the figures were plotted with R (104).

Reads containing CentC were divided into A and B chromosome groups according to the mapping results to genome sequence. The sequence identity to CentC consensus was determined using BLAST. The full-length centromeric retrotransposons CRM1 and CRM2 (96) on A and B chromosomes were retrieved from repeat annotation and their divergence and insertion times were analyzed.

Details of FISH, B chromosome size estimation, B chromosome sorting, annotation of repetitive sequences, TE family expansions, organellar DNA insertions, expression of B chromosomal genes, and tests of homology to A chromosomes are provided in *SI Appendix*.

## Supplementary Material

Supplementary File

Supplementary File

Supplementary File

Supplementary File

Supplementary File

Supplementary File

Supplementary File

Supplementary File

Supplementary File

Supplementary File

## Data Availability

Raw data used for sequence assembly of B73 line possessing B chromosome(s) and additional RNA-seq data used for B chromosome annotation are available in NCBI-SRA as BioProject PRJNA633287. Sequence reads for B-deficiency-carrying lines of maize are available in NCBI-SRA as BioProject PRJNA634743. Input-seq data are available in NCBI-GEO under accession GSE152074. The final B chromosome sequence and its annotation are available at MaizeGDB (https://www.maizegdb.org/) under the name Zm-B73_B-CHROMOSOME-MBSC-1.0 with the identifier Zm00044a.
