# Supplementary material for: Sequence of the supernumerary B chromosome of maize provides insight into its drive mechanism and evolution
Source: Proc Natl Acad Sci U S A. 2021 Jun 4;118(23):e2104254118. doi: 10.1073/pnas.2104254118 (PMC8201846; doi:10.1073/pnas.2104254118)
Supplement: Supplementary File [file pnas.2104254118.sapp.pdf]

PNAS
www.pnas.org

Supplementary Information for

Sequence of the supernumerary B chromosome of maize provides insight into its drive mechanism and evolution

Nicolas Blavet, Hua Yang, Handong Su, Pavel Solanský, Ryan N. Douglas, Miroslava Karafiátová, Lucie Šimková, Jing Zhang, Yalin Liu, Jie Hou, Xiaowen Shi, Chen Chen, Mohamed El-Walid, Morgan E. McCaw, Patrice S. Albert, Zhi Gao, Changzeng Zhao, Gil Ben-Zvi, Lior Glick, Guy Kol, Jinghua Shi, Jan Vrána, Hana Šimková, Jonathan C. Lamb, Kathleen Newton, R. Kelly Dawe, Jaroslav Doležel, Tieming Ji, Kobi Baruch, Jianlin Cheng, Fangpu Han, James A. Birchler, Jan Bartoš

Corresponding authors: Jan Bartos, James A. Birchler, Fangpu Han

Email: bartos@ueb.cas.cz; birchlerj@missouri.edu; fphan@genetics.ac.cn

This PDF file includes:

Supporting Information Methods

Figures S1 to S9

Tables S1 to S8

Legends for Datasets S1 to S9

SI References

Other supplementary materials for this manuscript include the following:

Datasets S1 to S9

Supporting Information Methods

Preparation of Chromosome Spreads and FISH

Kernels were germinated at 28°C for 2-3 days in moist vermiculite; the primary root tip was excised and then the seedlings were transplanted to the greenhouse. Probe preparation, somatic chromosome spreading, FISH, image capture, and processing were performed as described previously with minor modification (1, 2). Generally, excised root tips were treated with gaseous nitrous oxide (3) for 2-3 h. Treated root tips were fixed in ice-cold acetic acid-ethanol (9:1 dilution) for 10 min and stored in 70% ethanol at -20 °C until use. After washing in water on ice for 10 min, the root tip containing dividing cells was dissected and digested in 1% pectolyase and 3% cellulase solution for 30-50 min at 37 °C depending on the excised root tip size. After digestion, the root sections were briefly twice washed in 100% ethanol. The root sections were carefully broken using a needle in acetic acid methanol (9:1 dilution) solution. The cell suspension was dropped onto glass slides in a box lined with wet paper towels and dried slowly. After the cell spreads were dried on slides, they were UV-crosslinked for 1 min (total energy, 120 mJ/cm^2). At the center of the cell spreads, 10 µl of 2× SSC, 1× TE solution containing probe mixture (36 ng CentC; 10 ng TAG microsatellite; 6 ng ZmBs repeat or 10 ng Telomere, which cross-hybridizes with the ZmBs; and 50 ng NOR if applicable) was dropped. After application of a plastic coverslip, the slide preparation was denatured by placing onto a wet paper towel in an aluminium tray floating in boiling water (100°C) for 5 min. The slides were transferred immediately to a 55°C preheated humidity chamber containing water-soaked paper towels and incubated at 55°C oven overnight. Slides were submerged vertically into a Coplin jar filled with room temperature 2× SSC to remove coverslips and then washed in 2× SSC for 15 min at 55°C. Slides were removed from 2× SSC and shaken dry by hand. A drop of Vectashield (with DAPI) was added to the center of the probed cells

and a 22 mm × 50 mm glass coverslip was placed on the slide. After at least 20 min, spreads were screened using a fluorescence Olympus microscope. Images were processed with Photoshop.

B chromosome size estimation

The size of the B chromosome was estimated using flow cytometry in plants with six, eight, ten and twelve B chromosomes. Maize inbred line B73 was the background genome of all plants possessing B chromosomes. Nuclei were simultaneously isolated from young leaves of standard (i.e. B73 without Bs) and a plant with a defined number of B chromosomes. Briefly, equal amounts of tissue (approx. 0.5 cm^2) from the two plants were chopped using a razor blade in 0.5 ml OTTO I solution (4). Crude suspension was filtered through 20 µm mesh and mixed with 1ml OTTO II solution supplemented with 50 µg/ml propidium iodide and 50 µg/ml RNase. Fluorescence of stained nuclei was measured using a Partec PAS flow cytometer (Partec, Münster, Germany). Measurement was repeated three times on different dates for each line to avoid bias caused by instrument setup. Genome size of B-chromosomes-possessing lines was calculated from the ratio of peak positions for the two populations of nuclei (standard and B-positive) based on the assumption that the size of the haploid reference of B73 inbred line is 2,135 Mb (5). B chromosome size was determined by subtracting the reference genome size from the genome size of B-chromosomes-possessing lines and dividing the remainder by the respective number of B chromosomes.

B chromosome sorting and survey sequencing

Flow cytometry was used for chromosome isolation (6) of maize B chromosomes. Roots of young seedlings were treated with 3 mM hydroxyurea for 18 hours in order to synchronize the cell cycle. Accumulation of cells in metaphase was achieved using

nitrous oxide at 5 atmospheres for 2 hours. To prepare the chromosome suspension, roots were cut and transferred into 3% formaldehyde fixative for 25 min at 4°C. After washing in Tris buffer (3x5 min), root-tip meristems were excised and collected in 1 ml of LB01 lysis buffer and homogenized. The homogenate was filtered through 25 μm nylon mesh into a sample tube. Chromosomal DNA was stained with 2 μg/ml of DAPI and chromosome analysis and sorting were achieved using FACSAria II SORP flow cytometer (Becton Dickinson, Franklin Lakes, NJ, USA). Purity check was performed via FISH using the B-specific probe (7). Total of 235,000 flow-sorted chromosomes were treated by proteinase K and column-purified as described previously (8), yielding 24 ng DNA, which was then amplified in three independent reactions using Illustra GenomiPhi V2 DNA Amplification kit (GE Healthcare, Chalfont St. Giles, United Kingdom). Resulting 2.5 micrograms amplified DNA were pooled and used to prepare two paired-end libraries: the first one with ≈500 bp insert using DNA Library Preparation Kit v2 (Illumina, San Diego, CA, USA) and of the second one with ≈800 bp insert using Nextera XT DNA Library Preparation Kit (Illumina). The sequencing of both libraries was then performed on an Illumina HiSeq2000 sequencer (Illumina) at the Institute of Applied Genomics (Udine, Italy).

Annotation of repetitive sequences

Tandem repeat ZmBs (7) (accession number - S67586.1), 180-bp knob repeat (9) (accession number - M32530.1) and CentC satellite sequence (10) (accession number - AF078922.1) were identified via BLAST homology search (11).

The identification of LTR retrotransposons (LTR-TE), LINEs, SINEs, TIRs and helitrons was performed as described in Jiao et al. (5) using provided scripts with minor modifications. In brief, ZmBs, 180-bp knob repeat and CentC elements were masked prior analysis and LTR-TEs were identified using LTRharvest v1.5.10 (12) and

LTRdigest v1.5.10 (13). Seventy-six iterations were performed to discover the nested elements. Identified elements longer than 100 kb were excluded from further analyses because they are likely to be false positives. Retrieved LTR-TEs were clustered into families by the 5' LTR similarity based on comparison with vsearch v2.13.6 (14) and SiLiX v1.2.9 (15). Superfamily membership was assigned to each element based on the order of the genes for integrase, reverse transcriptase (RT) and RNaseH (if found in a particular element). Identification of those genes was performed using homology search (vsearch) against the provided consensus and exemplar transposable elements (TE) from the Maize TE Consortium (MTEC) (16, 17).

Regions annotated as a reverse transcriptase by LTRdigest were extracted from each LTR-TE in both A and B genomes. In some elements, the regions were composed of several highly significant matches with the Hidden Markov model (HMM) RT model database partly overlapping each other. In such cases, a whole region was considered relevant sequence for further analysis. Elements containing LTR-TE regions longer than twice the average length of the RT consensuses were excluded from further analysis. The RT regions of elements in each family were aligned using Clustal Omega v1.2.4 (18) and the family consensus was generated with EMBOSS cons v6.6.0.0 (19). After the alignment of the consensus sequences of all families, the maximum likelihood tree was generated with FastTree v2.1.11 (20).

SINE-finder v1.0.1 (21) was used to search for SINEs and TARGeT v2.10 (22), mTEA pipeline v1.0 (https://github.com/stajichlab/mTEA) and detectMITE v20170425 (23) to recover LINE and TIR elements. Helitron candidates were identified using HelitronScanner v1.0 (24) and assigned to families with SiLiX and vsearch in a similar manner as performed for LTR-TEs.

TE family expansion

Sequences of 5' and 3' LTRs were extracted from each LTR-TE in both the A and B genomes. Pairwise alignment of each pair was performed with Clustal Omega and nucleotide distances were counted with EMBOSS distmat employing Kimura correction. Mutation rate 3.3 x 10^{-9} proposed by Clark et al. (25) was used for the insertion date estimation. The identical approach was applied for elements originating in sequence of the B chromosome and A chromosomal complement. The number of all members of each family on the B chromosome was used as a test set for one-sided Fisher's exact test. The sum of members in A chromosomal complement served as a reference set for testing. The p-value was adjusted via FDR correction (26).

Identification of insertions of organellar DNA

The maize chloroplast genome (NC_001666.2) and 10 mitochondrial genomes (NA: DQ490952, NB: NC_007982, CMS-C: DQ645536, CMS-S: DQ490951, CMS-T: DQ490953, Zmp: DQ645539, Zp: DQ645538, Zl: DQ645537, Td: DQ984517, Sb: NC_008360) were aligned to the B chromosome sequence. The selected mitochondrial genomes were from: the “normal” maize cytotypes NB (27) and NA; the maize cytoplasmic male steriles C, T and S (28), the most closely related teosinte *Zea mays* ssp. *parviglumis* (Zmp); the most distantly related teosintes, *Zea perennis* (Zp) and *Zea luxurians* (Zl), as well as other grass relatives, *Tripsacum dactyloides* (Td) and *Sorghum bicolor* (Sb). The alignments were performed using NCBI MegaBlast with the default algorithm parameters. The resulting hit-tables and the B chromosome sequence were provided to an in-house program that generated a one-dimensional Boolean array representation of the B chromosome. The hit-tables for each of the 10 mitochondrial and one chloroplast genome were then parsed one at a time, extracting the coordinates of homology on the B chromosome. This approach generated an array representation of the B chromosome with the locations of homology depicted across

the sequence. The sequence was then traversed in 300-kb windows and the number of true values within the window was counted and printed to a file. This in-house program was run independently for chloroplast and for total mitochondrial homologies. FISH analysis of the B chromosome using probes to mitochondrial and chloroplastic DNA sequences was performed as described previously (29).

Expression analysis of B-linked genes

RNA-seq reads generated from leaves of maize B73 containing no, one and six B chromosomes (30) were used for the expression analysis. Data were retrieved from Sequence Read Archive (SRA, http://www.ncbi.nlm.nih.gov/sra, accession number SRP072810). Reads were trimmed with Trimmomatic v0.36 (31) with the parameters LEADING:20, TRAILING:20, SLIDINGWINDOW:4:20 and MINLEN:80. Trimmed reads were mapped using Hisat2 v2.0.5 (32) to the B73 v4 genome (5) supplemented with B chromosome sequence. Output bam files were sorted via Samtools v1.3.1 (33). In further analysis, only genes with expression higher than one read per million (RPM >1) in at least one sample were considered. Seqmonk v1.45.4 (http://www.bioinformatics.babraham.ac.uk/projects/seqmonk) was used to perform RNA-seq quantitation pipeline with CDSs as transcript features. Further, Seqmonk was used to run a differential expression analysis with DESeq2 (34) and with p-value set to < 0.05.

Test of homology of the B chromosome to the A chromosomes

The copy number variation analysis was performed as described previously (35). Generally, single-end reads after filtering low quality reads were aligned to the maize W22 reference genome (36) using Bowtie2 (37). Only uniquely mapped reads with no more than 2 mismatches were kept. To examine the copy number change with a single

gene window, the reads from per gene windows were counted along each chromosome and the results were plotted by ggplot2 in R (38).

References to Supporting Information

1. A. Kato, J. C. Lamb, J. A. Birchler, Chromosome painting using repetitive DNA sequences as probes for somatic chromosome identification in maize. *Proc Natl Acad Sci U S A* **101**, 13554–13559 (2004).

2. J. C. Lamb, N. C. Riddle, Y.-M. Cheng, J. Theuri, J. A. Birchler, Localization and transcription of a retrotransposon-derived element on the maize B chromosome. *Chromosome Res.* **15**, 383–398 (2007).

3. A. Kato, Air drying method using nitrous oxide for chromosome counting in maize. *Biotech Histochem* **74**, 160–166 (1999).

4. J. Doležel, J. Bartoš, Plant DNA flow cytometry and estimation of nuclear genome size. *Ann. Bot.* **95**, 99–110 (2005).

5. Y. Jiao, *et al.*, Improved maize reference genome with single-molecule technologies. *Nature* **546**, 524-527 (2017).

6. J. Vrána, H. Simková, M. Kubaláková, J. Cíhalíková, J. Doležel, Flow cytometric chromosome sorting in plants: the next generation. *Methods* **57**, 331–337 (2012).

7. M. R. Alfenito, J. A. Birchler, Molecular characterization of a maize B chromosome centric sequence. *Genetics* **135**, 589–597 (1993).

8. H. Šimková, *et al.*, Coupling amplified DNA from flow-sorted chromosomes to high-density SNP mapping in barley. *BMC Genomics* **9**, 294 (2008).

9. W. J. Peacock, E. S. Dennis, M. M. Rhoades, A. J. Pryor, Highly repeated DNA sequence limited to knob heterochromatin in maize. *Proc Natl Acad Sci U S A* **78**, 4490–4494 (1981).

10. E. V. Ananiev, R. L. Phillips, H. W. Rines, Chromosome-specific molecular organization of maize (*Zea mays* L.) centromeric regions. *Proc. Natl. Acad. Sci. U.S.A.* **95**, 13073–13078 (1998).

11. S. F. Altschul, *et al.*, Gapped BLAST and PSI-BLAST: a new generation of protein database search programs. *Nucleic Acids Research* **25**, 3389–3402 (1997).

12. D. Ellinghaus, S. Kurtz, U. Willhoeft, LTRharvest, an efficient and flexible software for de novo detection of LTR retrotransposons. *BMC Bioinformatics* **9**, 18 (2008).

13. S. Steinbiss, U. Willhoeft, G. Gremme, S. Kurtz, Fine-grained annotation and classification of de novo predicted LTR retrotransposons. *Nucleic Acids Res* **37**, 7002–7013 (2009).

14. T. Rognes, T. Flouri, B. Nichols, C. Quince, F. Mahé, VSEARCH: a versatile open source tool for metagenomics. *PeerJ* **4**, e2584 (2016).

15. V. Miele, S. Penel, L. Duret, Ultra-fast sequence clustering from similarity networks with SiLiX. *BMC Bioinformatics* **12**, 116 (2011).

16. P. S. Schnable, *et al.*, The B73 maize genome: complexity, diversity, and dynamics. *Science* **326**, 1112–1115 (2009).

17. R. S. Baucom, *et al.*, Exceptional diversity, non-random distribution, and rapid evolution of retroelements in the B73 maize genome. *PLoS Genet.* **5**, e1000732 (2009).

18. F. Sievers, *et al.*, Fast, scalable generation of high-quality protein multiple sequence alignments using Clustal Omega. *Molecular Systems Biology* **7**, 539 (2011).

19. P. Rice, I. Longden, A. Bleasby, EMBOSS: the European Molecular Biology Open Software Suite. *Trends Genet.* **16**, 276–277 (2000).

20. M. N. Price, P. S. Dehal, A. P. Arkin, FastTree 2--approximately maximum-likelihood trees for large alignments. *PLoS ONE* **5**, e9490 (2010).

21. T. Wenke, *et al.*, Targeted Identification of Short Interspersed Nuclear Element Families Shows Their Widespread Existence and Extreme Heterogeneity in Plant Genomes. *The Plant Cell* **23**, 3117–3128 (2011).

22. Y. Han, J. M. Burnette, S. R. Wessler, TARGeT: a web-based pipeline for retrieving and characterizing gene and transposable element families from genomic sequences. *Nucleic Acids Res* **37**, e78–e78 (2009).

23. C. Ye, G. Ji, C. Liang, *detectMITE*: A novel approach to detect miniature inverted repeat transposable elements in genomes. *Scientific Reports* **6**, 19688 (2016).

24. W. Xiong, L. He, J. Lai, H. K. Dooner, C. Du, HelitronScanner uncovers a large overlooked cache of Helitron transposons in many plant genomes. *Proc Natl Acad Sci U S A* **111**, 10263–10268 (2014).

25. R. M. Clark, S. Tavaré, J. Doebley, Estimating a Nucleotide Substitution Rate for Maize from Polymorphism at a Major Domestication Locus. *Mol Biol Evol* **22**, 2304–2312 (2005).

26. Y. Benjamini, Y. Hochberg, Controlling the False Discovery Rate: A Practical and Powerful Approach to Multiple Testing. *Journal of the Royal Statistical Society: Series B (Methodological)* **57**, 289–300 (1995).

27. S. W. Clifton, *et al.*, Sequence and Comparative Analysis of the Maize NB Mitochondrial Genome. *Plant Physiology* **136**, 3486–3503 (2004).

28. J. O. Allen, *et al.*, Comparisons Among Two Fertile and Three Male-Sterile Mitochondrial Genomes of Maize. *Genetics* **177**, 1173–1192 (2007).

29. A. N. Lough, *et al.*, Mitochondrial DNA Transfer to the Nucleus Generates Extensive Insertion Site Variation in Maize. *Genetics* **178**, 47–55 (2008).

30. W. Huang, Y. Du, X. Zhao, W. Jin, B chromosome contains active genes and impacts the transcription of A chromosomes in maize (*Zea mays* L.). *BMC Plant Biology* **16**, 88 (2016).

31. A. M. Bolger, M. Lohse, B. Usadel, Trimmomatic: a flexible trimmer for Illumina sequence data. *Bioinformatics* **30**, 2114–2120 (2014).

32. D. Kim, B. Langmead, S. L. Salzberg, HISAT: a fast spliced aligner with low memory requirements. *Nat Methods* **12**, 357–360 (2015).

33. H. Li, *et al.*, The Sequence Alignment/Map format and SAMtools. *Bioinformatics* **25**, 2078–2079 (2009).

34. M. I. Love, W. Huber, S. Anders, Moderated estimation of fold change and dispersion for RNA-seq data with DESeq2. *Genome Biol.* **15**, 550 (2014).

35. H. Yang, *et al.*, Predominantly inverse modulation of gene expression in genomically unbalanced disomic haploid maize. *The Plant Cell* (2021) https:/doi.org/10.1093/plcell/koab029 (February 9, 2021).

36. N. M. Springer, *et al.*, The maize W22 genome provides a foundation for functional genomics and transposon biology. *Nature Genetics* **50**, 1282 (2018).

37. B. Langmead, S. L. Salzberg, Fast gapped-read alignment with Bowtie 2. *Nat. Methods* **9**, 357–359 (2012).

38. H. Wickham, *ggplot2: Elegant Graphics for Data Analysis* (Springer-Verlag, 2016) (March 26, 2019).

39. J. B. Beckett, “Cytogenetic, Genetic and Plant Breeding Applications of B–A Translocations in Maize” in *Developments in Plant Genetics and Breeding*, P.K. Gupta and T. Tsuchiya, Ed. (Elsevier, 1991), pp. 493–529.

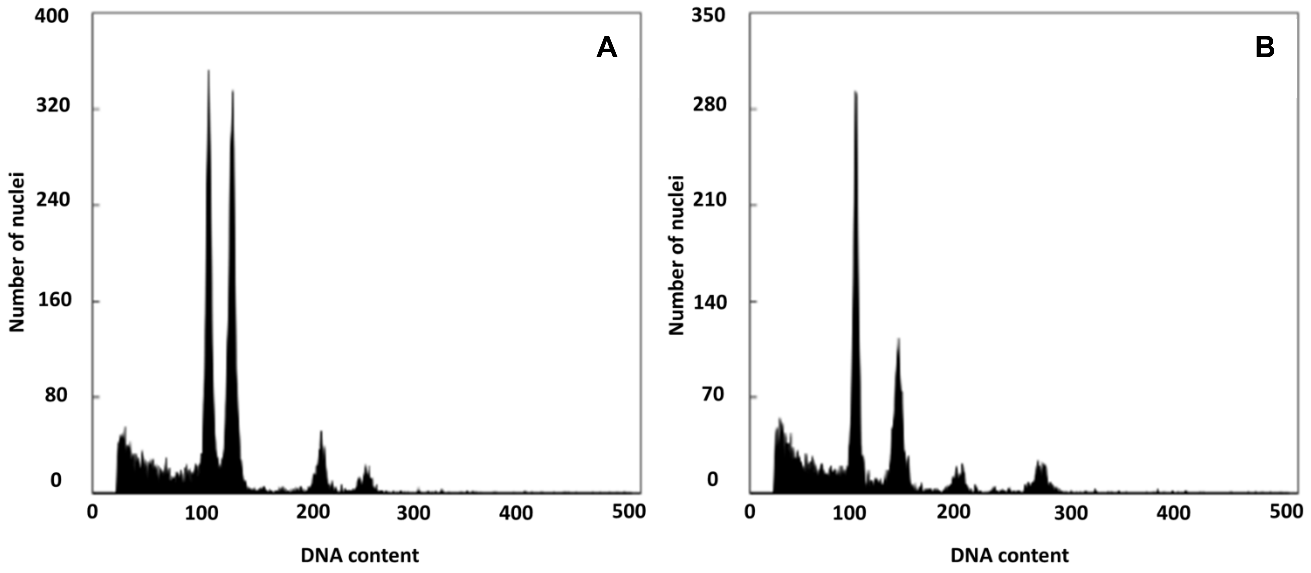

Fig. S1.

Estimation of genome size in lines possessing B chromosomes. Histograms of nuclear DNA content were obtained using flow cytometry of propidium iodide-stained nuclei isolated simultaneously from inbred line B73 and line B73 possessing six (A) and ten (B) B chromosomes. Left peak - B73; right peak - B73 plus six or ten Bs.

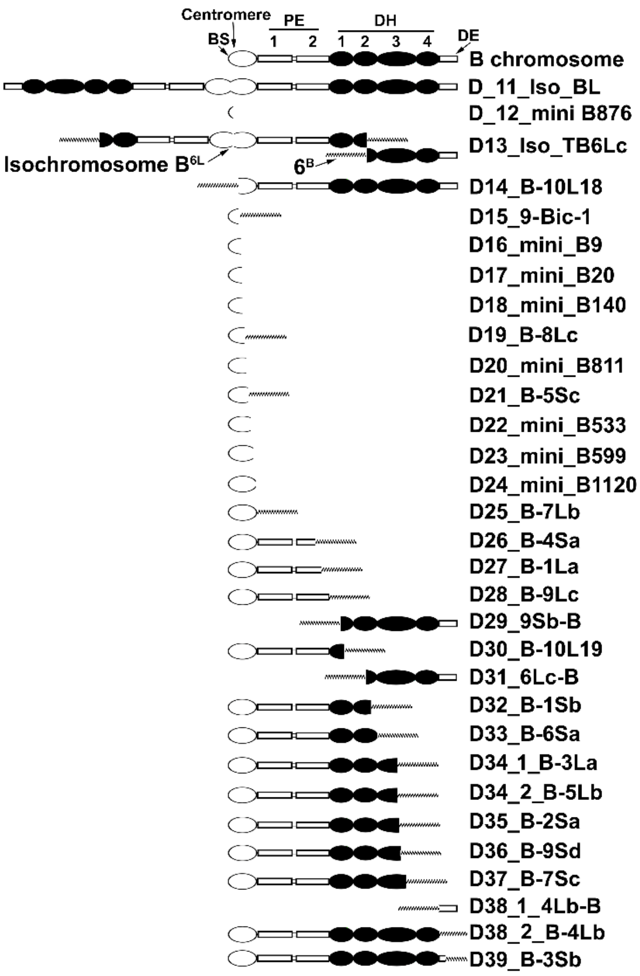

Fig. S2.

Diagram of B-deficiency materials. The B chromosome consists of a short arm (BS), a centromere, and a long arm composed of a small heterochromatic knob (PH), an euchromatic region (PE), a long heterochromatic region consisting of four blocks (DH), and finally a short distal euchromatic region (DE) (39). B-A translocations are interchanges that involve a B-chromosome and members of the basic (A) set of chromosomes. B-A translocated chromosomes are usually designated in the form BA or AB. The superscripts denote the translocated arm to an arm with a centromere. The A chromosome arms are shown as wavy lines and are not drawn to scale. D11, Isochromosome B long arm (Iso_BL); D12, mini B876; D13, Isochromosome TB-6Lc euploid (Iso-B-6Lc + 6Lc-B); D14, B-10L18 (TB-10L18 tertiary trisomy); D15, 9-B inactivated centromere-1 (9-Bic-1); D16, miniB9; D17, mini B20; D18, mini B140; D19, B-8Lc (TB-8Lc disomy); D20, mini B881; D21, B-5Sc (TB-5Sc tertiary trisomy); D22, mini B533; D23, mini B599; D24, mini B1120; D25, B-7Lb (TB-7Lb disomy); D26, B-4Sa (TB-4Sa disomy); D27, B-1La (TB-1La disomy); D28, B-9Lc (TB-9Lc disomy); D29, 9Sb-B (TB-9Sb monosomy); D30, B-10L19 (TB-10L19 disomy); D31, 6Lc-B (TB-6Lc monosomy); D32, B-1Sb (TB-1Sb disomy); D33, B-6Sa (TB-6Sa tertiary trisomy); D34_1, B-3La (TB-3La disomy); D34_2, B-5Lb (TB-5Lb disomy); D35, B-2Sa (TB-2Sa disomy); D36, B-9Sd (TB-9Sd disomy); D37, B-7Sc (TB-7Sc disomy); D38_1, 4Lb-B (TB-4Lb monosomy); D38_2, B-4Lb (TB-4Lb disomy); D39, B-3Sb (TB-3Sb disomy). The delineation of the *cis* region for nondisjunction is described in the text. The region for preferential fertilization resides between the breakpoints of TB-10L18 and TB-8Lc. The region for *trans*-acting factor #1 resides distal to the breakpoint in TB-3Sb.

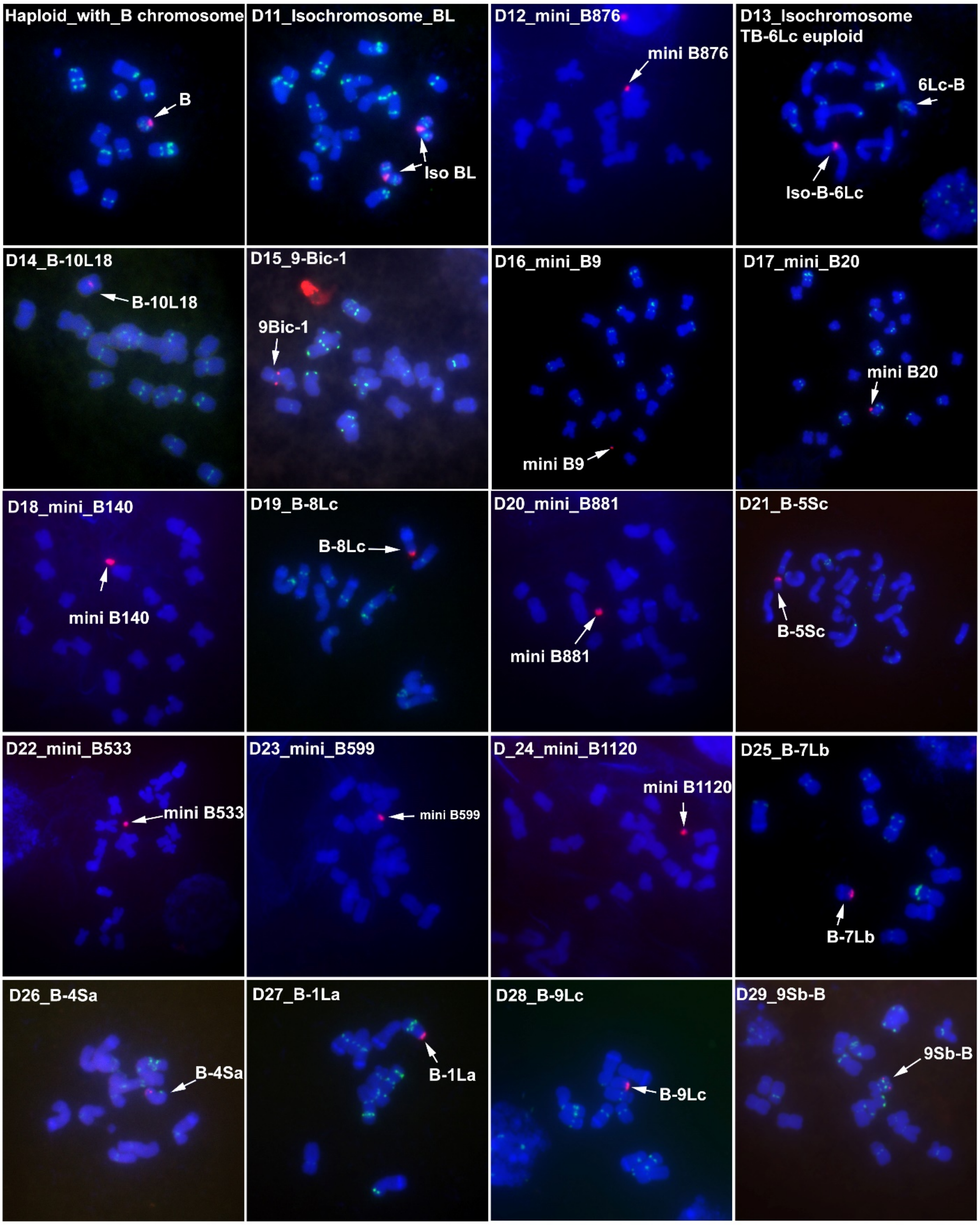
Haploid_with_B chromosome
B
D11_Isochromosome_BL
Iso BL
D12_mini_B876
mini B876
D13_Isochromosome TB-6Lc euploid
6Lc-B
Iso-B-6Lc
D14_B-10L18
B-10L18
D15_9-Bic-1
9Bic-1
D16_mini_B9
mini B9
D17_mini_B20
mini B20
D18_mini_B140
mini B140
D19_B-8Lc
B-8Lc
D20_mini_B881
mini B881
D21_B-5Sc
B-5Sc
D22_mini_B533
mini B533
D23_mini_B599
mini B599
D_24_mini_B1120
mini B1120
D25_B-7Lb
B-7Lb
D26_B-4Sa
B-4Sa
D27_B-1La
B-1La
D28_B-9Lc
B-9Lc
D29_9Sb-B
9Sb-B

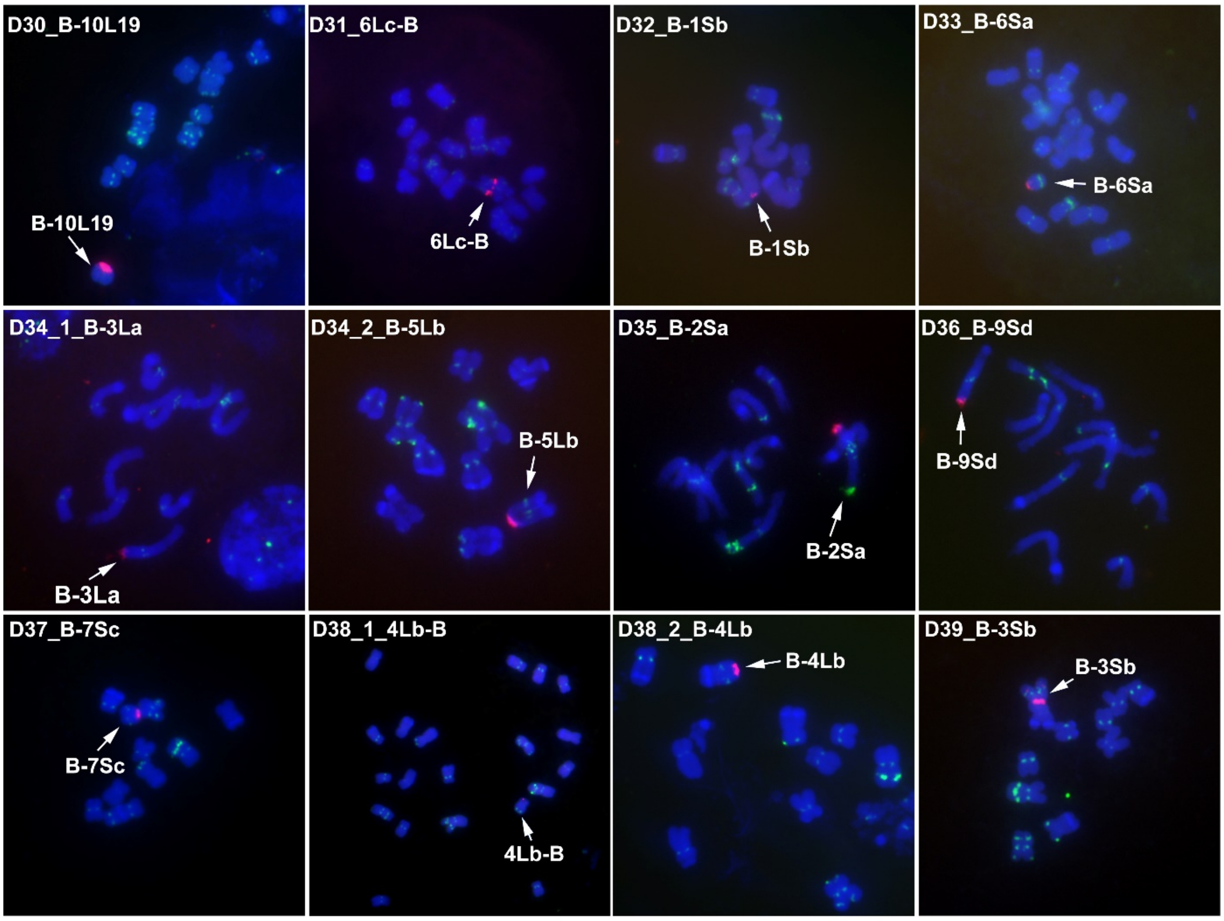

Fig. S3.

Identification of deficient B chromosomes by FISH. Mitotic metaphase spread of deficiency material used for the mapping. The probes for FISH included CentC (green), TAG microsatellite (green), B-specific repeat, ZmBs (red), nucleolus organizer region sequences (NOR, green) and telomere sequences (Telo, red) counterstained with 4', 6-diamidino-2-phenylindole (blue). CentC was enriched in centromeric regions of both A and B chromosomes. TAG is found in the long arm of chromosome 1, long arm and short arm of chromosome 2, short arm of chromosome 4 and the long arm of the B chromosome. NOR is only in the short arm of chromosome 6. ZmBs showed an intense signal in the B centromere region and one minor site in the B distal heterochromatin and another minor site in the B distal euchromatin (long arm tip). Knob, shown in intense blue, can be detected in the long arm of chromosomes 4, 5, 7, 8, and the short arm of chromosome 9. Telomere was enriched at the tip of each chromosome arm. In mini B140, 533, 599, 876, 881 and 1120, only ZmBs was used. For the remainder of the chromosomes, except TB-6Sa tertiary trisomy, CentC, TAG and ZmBs were applied for FISH. For TB-6Sa tertiary trisomy, NORs and Telomere were used in addition to CentC, TAG and ZmBs. In D16, D17, D21 and D38_1_4Lb_B, bar indicates 3.33 μm, while the bar in other FISH images is 5 μm.

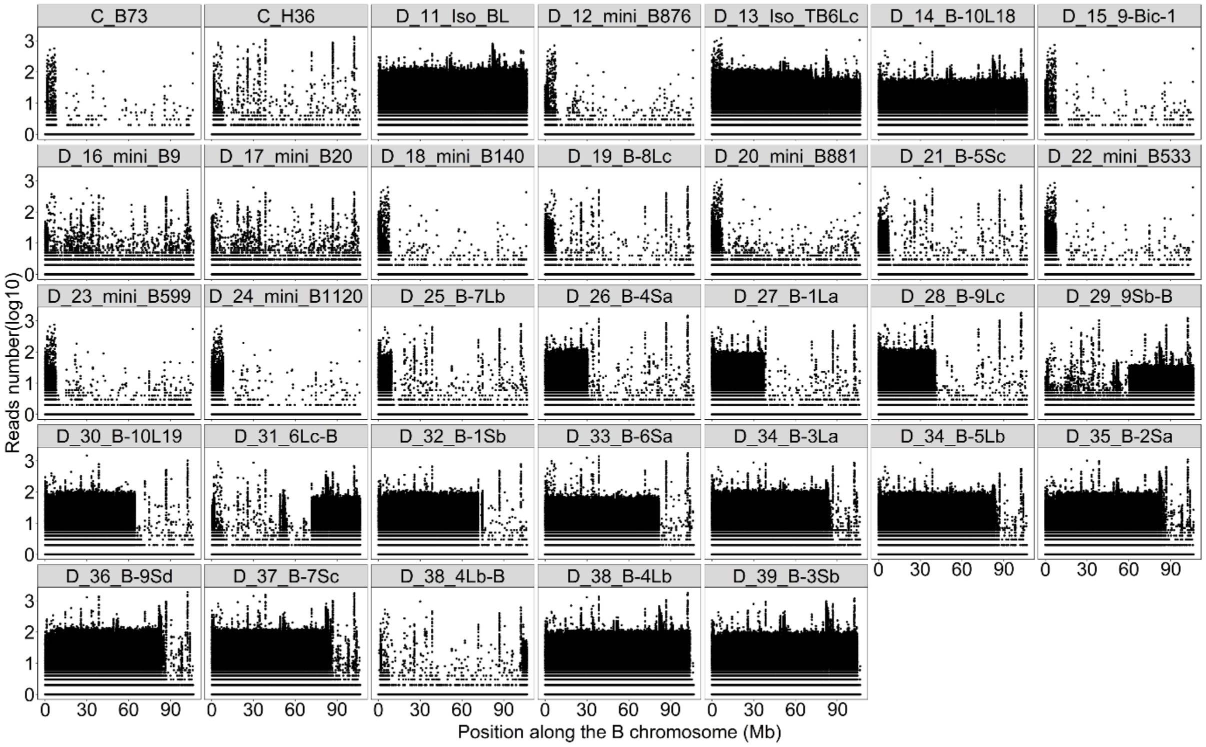

Fig. S4.

Deficiency mapping. Sequence reads of all B chromosome deficiency data and controls were mapped to the B genome assembly and pseudomolecule. Each dot represents the read count in 1 Kb windows along the pseudomolecule. C - control; D - deficiency. C_B73 and C_H36 contain no B chromosome and were used as negative controls. The background results from homology to the 180 knob repeats, long arm CentC repeats, and organellar DNA insertions, which are all present on the A chromosomes and thus will show homology to the B sequence.

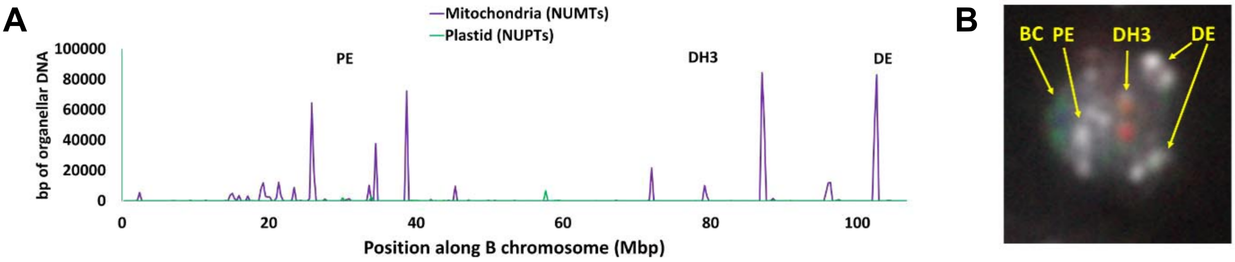

Fig. S5.

Distribution of organellar sequences on the B chromosome. (A) Mitochondrial and plastid sequences in the chrB v1.0 pseudomolecule. (B) Hybridization of mitochondrial DNA to the B chromosome. A set of probes consisting of mtDNA cloned from the NB mitogenome hybridized strongly (white) to specific parts of the B chromosome (root-tip mitotic metaphase): the proximal euchromatin (PE), distal heterochromatin (DH3), and distal euchromatin (DE). CentC (green) is a marker for the B centromere (BC), and the TAG microsatellite probe (red) marks the 3rd block of distal heterochromatin (DH3).

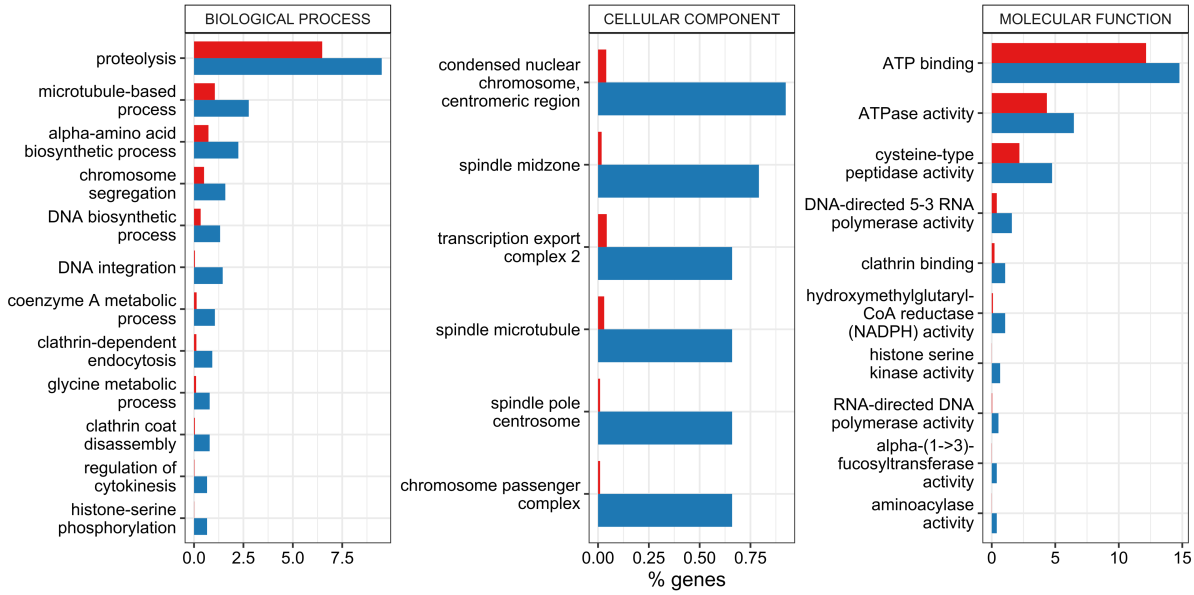

Fig. S6.

GO term enrichment of the B chromosome genes. GO terms enriched among genes encoded in the B chromosome sequence (red) compared to frequency of genes in the same GO term in A chromosomal complement (blue). The significance was tested using one-sided Fisher's exact test with correction via FDR and p-value threshold of 0.05.

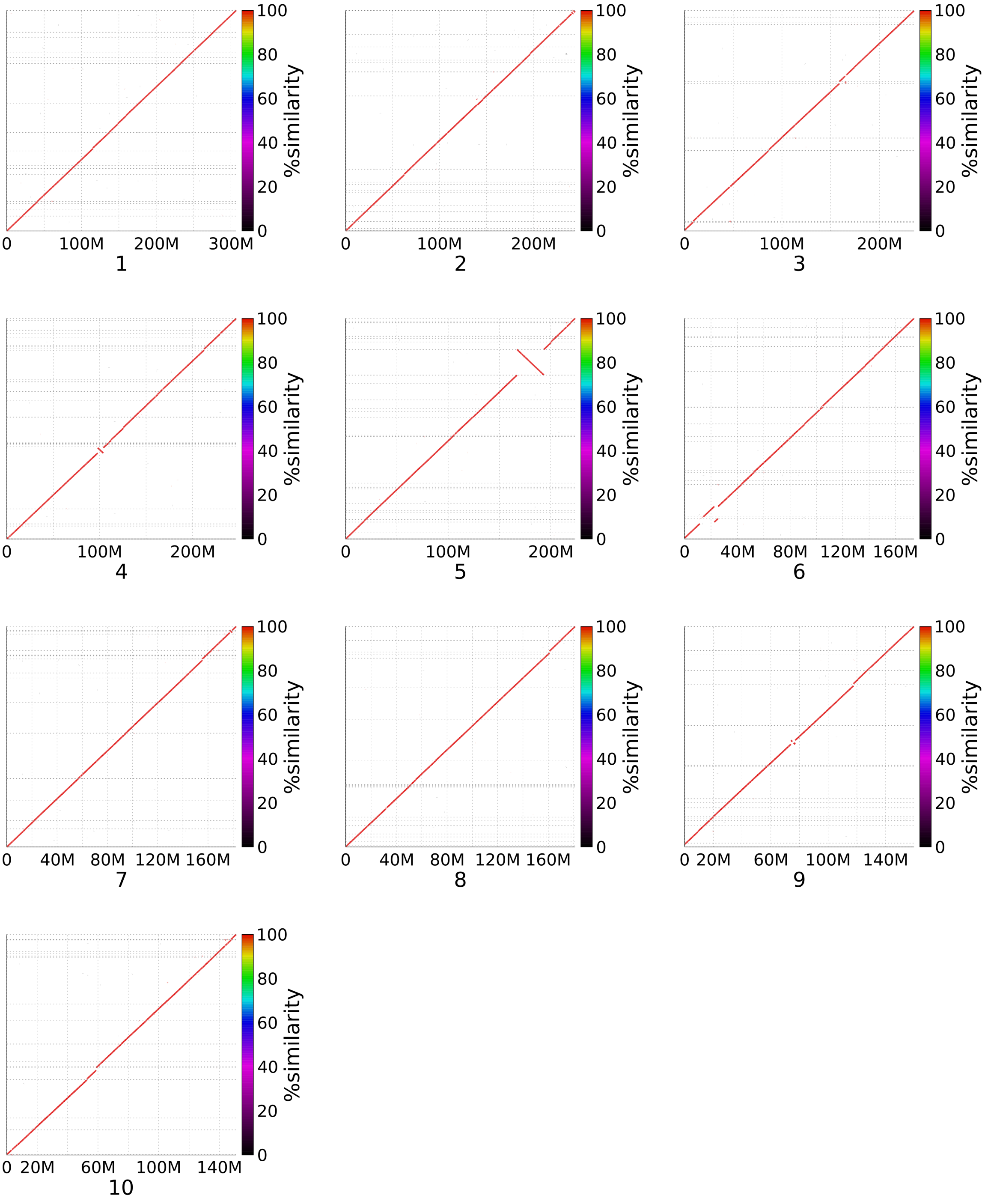

Fig. S7.

Alignment of final scaffolds (Y-axis) to pseudomolecules of ten chromosomes of B73 genome (X-axis) (5). Note nearly perfect collinearity and high similarity of the sequence.

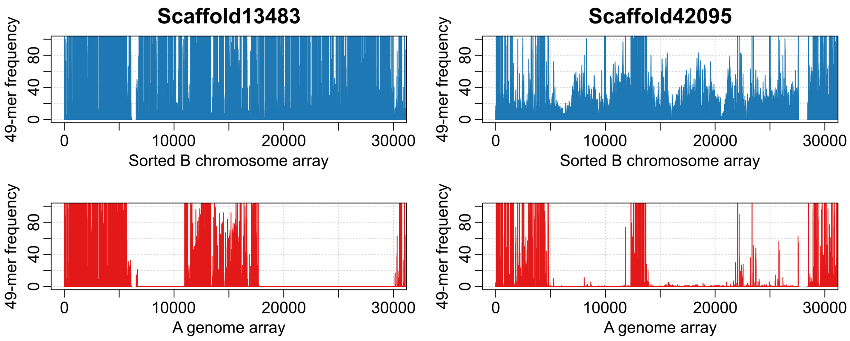

Fig. S8.

K-mer profile of two scaffolds assigned to the B chromosome sequence. Scaffold sequences were profiled with 49-mer frequencies in 20x coverage of flow-sorted B chromosome sequence (blue) and B73 genome (red). Repetitive sequences were deduced from the profile with the B73 genome. Scaffolds were assigned to the B chromosome based on an increased coverage of regions not abundant in the B73 genome in the B chromosomal profile.

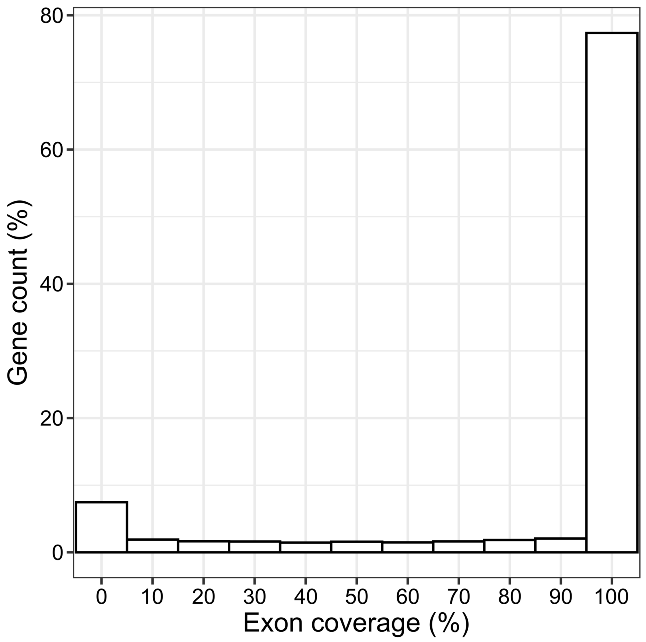

Fig. S9.

Evaluation of gene annotation pipeline. Comparison of results of annotation of chromosome 1 of B73v4 with the pipeline used for the B chromosome and the one used for the A chromosomal complement (5). X-axis corresponds to coverage of exons in B73v4 by gene models retrieved by the pipeline used in this study. Y-axis shows the number of exons with a particular match.

Table S1. Characteristics of libraries used for sequencing and subsequent assembly.

id	source	type*	insert size	read length	# reads	cumulative size [bp]	genome/chr coverage
1C	chromosome	PE	500 bp	2 x 100	113,076,072	11,307,607,200	80x
2C	chromosome	PE	800 bp	2 x 100	130,691,634	13,069,163,400	93x
3G	genome	PE	470 bp	2 x 260	565,002,342	146,900,608,920	65x
4G	genome	PE	800 bp	2 x 160	378,397,716	60,543,634,560	27x
5G	genome	MP	2-5 kb	2 x 160	529,645,376	84,743,260,160	37x
6G	genome	MP	5-7 kb	2 x 160	481,303,170	77,008,507,200	34x
7G	genome	MP	7-10 kb	2 x 160	512,273,014	81,963,682,240	36x
8H	genome	Hi-C	N/A	2 x 150	812,481,012	121,872,151,800	54x

* PE – pair-end; MP – mate-pair
Acc. numbers in NCBI-SRA
1C – SRR11802371
2C – SRR11802370
3G – SRR11802369
4G – SRR11802368
5G – SRR11802367
6G – SRR11802366
7G – SRR11802365
8H – SRR11802364

Table S2. Assembly statistics of the maize B73 genome with B chromosomes.

	DeNovoMAGIC B73+B assembly	DeNovoMAGIC assembly of B*	Final B73+B assembly	Final assembly of B*
Total [Mbp]	2,341.8	120.0	2,369.4	125.9
# scaffolds	66,975	361	66,269	328
max. length [bp]	22,782,047	16,642,982	81,955,313	71,674,295
N50 [bp]	3,841,203	2,992,294	23,788,118	71,674,295
# N50 scaffolds	178	11	31	1
# Ns	34,580,123	3,714,006	62,121,592	9,068,090
# Gaps	53,082	5,523	57,589	6,286

* extracted from the assembly of B73 line possessing B chromosome(s). Only scaffolds with length ≥ 10kb were selected.

Table S3. Estimation of B-chromosome size in lines possessing different number of B chromosomes.

Line	standard [peak]	sample [peak]	peak ratio	genome size sample [2C/Mbp]	B size [Mbp]	mean		SD
B73 + 6B	110.35	131.91	1.1954	5,104.3	139.0	139.1	±	1.7
	99.95	119.26	1.1932	5,094.9	137.5			
	107.84	129.18	1.1979	5,115.0	140.8			
B73 + 8B	109.13	137.91	1.2637	5,396.1	140.8	138.9	±	1.9
	102.54	128.86	1.2567	5,366.0	137.0			
	100.35	126.47	1.2603	5,381.4	138.9			
B73 + 10B	110.40	146.15	1.3238	5,652.7	138.3	141.6	±	3.0
	99.93	133.68	1.3377	5,712.1	144.2			
	110.00	146.65	1.3332	5,692.7	142.3			
B73 + 12B	107.96	151.57	1.4039	5,994.8	143.7	143.4	±	0.8
	98.18	137.50	1.4005	5,980.1	142.5			
	111.67	156.83	1.4044	5,996.8	143.9			
**Average B chromosome size**						**140.7**	**±**	**2.6**

The estimates are based on the assumption that the size of the haploid reference of B73 inbred line is 2,135 Mb (5).

Table S4. Cumulative length of TEs identified in the sequence of the B chromosome.

Feature	Length (kbp)	Length (%)
LTR-TE gypsy	39,614.6	32.69
LTR-TE unassigned	14,327.3	11.82
LTR-TE copia	13,620.5	11.24
Helitron	4,319.2	3.56
TIR	401.2	0.33
LINE	20.1	0.02
SINE	19.3	0.02

Table S5. GO terms enriched on the B chromosome in comparison to the maize A chromosomal complement.

GO ID	GO Name	GO category	FDR	# in B	# in A
GO:0015074	DNA integration	BP	4.6e-09	11	18
GO:0051233	spindle midzone	CC	6.3e-05	6	7
GO:0000780	condensed nuclear chromosome, centromeric region	CC	1.0e-04	7	16
GO:0035404	histone-serine phosphorylation	BP	1.3e-04	5	4
GO:0035174	histone serine kinase activity	MF	1.3e-04	5	4
GO:0031616	spindle pole centrosome	CC	1.3e-04	5	4
GO:0032133	chromosome passenger complex	CC	1.3e-04	5	4
GO:0004420	hydroxymethylglutaryl-CoA reductase (NADPH) activity	MF	4.1e-04	8	37
GO:0008234	cysteine-type peptidase activity	MF	5.2e-04	36	862
GO:0032465	regulation of cytokinesis	BP	6.1e-04	5	8
GO:0072318	clathrin coat disassembly	BP	6.1e-04	6	17
GO:0015936	coenzyme A metabolic process	BP	1.6e-03	8	52
GO:0005876	spindle microtubule	CC	1.6e-03	5	12
GO:0007017	microtubule-based process	BP	3.4e-03	21	417
GO:1901607	alpha-amino acid biosynthetic process	BP	3.4e-03	17	289
GO:0006508	proteolysis	BP	3.4e-03	72	2,568
GO:0003899	DNA-directed 5'-3' RNA polymerase activity	MF	5.1e-03	12	157
GO:0070390	transcription export complex 2	CC	5.1e-03	5	17
GO:0072583	clathrin-dependent endocytosis	BP	5.6e-03	7	47
GO:0006544	glycine metabolic process	BP	1.9e-02	6	41
GO:0071897	DNA biosynthetic process	BP	2.1e-02	10	133
GO:0005524	ATP binding	MF	2.4e-02	112	4,810
GO:0030276	clathrin binding	MF	2.4e-02	8	86
GO:0007059	chromosome segregation	BP	2.9e-02	12	201
GO:0004046	aminoacylase activity	MF	3.0e-02	3	5
GO:0016887	ATPase activity	MF	3.1e-02	49	1,718
GO:0003964	RNA-directed DNA polymerase activity	MF	3.3e-02	4	16
GO:0046920	alpha-(1->3)-fucosyltransferase activity	MF	3.8e-02	3	6

MF - molecular function; BP - biological process; CC - cellular component. FDR – false discovery rate. Significance was tested using one-sided Fisher's exact test with correction via FDR and p-value threshold of 0.05.

Table S6. Genes in the region of *trans*-factor #1

gene_ID	sequence name	start	end	CDS_length	description
Zm00044a000678	chrB	104 544 832	104 545 490	201	cystinosin homolog isoform X1
Zm00044a000677	chrB	104 579 383	104 579 609	93	---NA---
Zm00044a000676	chrB	104 595 237	104 595 880	117	---NA---
Zm00044a000675	chrB	104 607 429	104 608 071	117	---NA---
Zm00044a000674	chrB	104 863 213	104 866 079	774	putative clathrin assembly protein
Zm00044a000673	chrB	105 154 547	105 165 072	6 639	retrotransposon protein, putative, unclassified
Zm00044a000672	chrB	105 166 736	105 176 430	1 749	RNA polymerase2
Zm00044a000671	chrB	105 212 501	105 213 824	1 074	RMUA
Zm00044a000670	chrB	105 219 378	105 221 069	540	serine/threonine-protein kinase EDR1-like isoform X1
Zm00044a000669	chrB	105 253 605	105 255 251	1 305	Bzip transcription factor-like
Zm00044a000668	chrB	105 303 867	105 310 450	1 197	Aspartyl protease family protein 1
Zm00044a000667	chrB	105 793 779	105 806 007	3 066	putative gag-pol polyprotein
Zm00044a000666	chrB	105 834 730	105 836 278	1 107	F-box protein At5g49610
Zm00044a000665	chrB	105 844 042	105 849 639	1 770	uncharacterized protein LOC103645298 isoform X2
Zm00044a000664	chrB	105 868 343	105 869 262	570	transposase-associated domain protein
Zm00044a000663	chrB	106 149 488	106 158 111	3 969	probable helicase MAGATAMA 3
Zm00044a000685	chrB	106 401 599	106 407 347	594	60S ribosomal protein L11
Zm00044a000686	chrB	106 586 773	106 591 885	987	Chaperone protein ClpB1
Zm00044a000691	scaffold49456	15 350	21 179	1 539	putative ubiquitin-like-specific protease 1B
Zm00044a000692	scaffold49456	65 742	77 149	1 407	WD-40 repeat-containing protein MSI4
Zm00044a000693	scaffold49456	99 292	101 818	1 266	hAT transposon superfamily protein
Zm00044a000706	scaffold42216	30 435	34 069	825	Sas10/Utp3/C1D family
Zm00044a000707	scaffold42216	123 180	127 973	495	3-hydroxy-3-methylglutaryl-coenzyme A reductase 2
Zm00044a000708	scaffold42216	128 277	129 618	342	Sas10/Utp3/C1D family
Zm00044a000709	scaffold21734	20 478	21 161	216	Serine/threonine-protein kinase Aurora-3
Zm00044a000710	scaffold21734	26 313	27 868	540	Peptidase/ serine-type peptidase
Zm00044a000711	scaffold21734	51 831	53 402	282	peptidase/ serine-type peptidase
Zm00044a000718	scaffold60435	1 219	8 820	912	oligomeric Golgi complex component-related protein
Zm00044a000719	scaffold60435	10 450	20 569	387	Protein SPIRRIG
Zm00044a000720	scaffold60435	29 602	31 746	627	AT-rich interactive domain-containing protein 1A-like isoform X3
Zm00044a000724	scaffold33271	7 948	14 333	2 136	DUF724 domain-containing protein 3 isoform X2
Zm00044a000725	scaffold33271	30 743	32 855	735	E3 ubiquitin-protein ligase At4g11680
Zm00044a000746	scaffold60536	73 628	75 610	1 407	Serine/threonine-protein kinase Aurora-3
Zm00044a000751	scaffold2414	9 526	10 029	162	nuclear pore complex protein NUP54 isoform X1

Table S7. Representation of ten pseudomolecules of the B73 genome (5) in the assembly of B73+B line.

Chromosome	Coverage (%)	Identity (%)	# of scaffolds
chr01	97.27	99.71	45
chr02	96.66	99.75	43
chr03	97.57	99.78	35
chr04	96.84	99.76	44
chr05	96.95	99.72	43
chr06	95.64	99.76	35
chr07	97.29	99.77	40
chr08	97.12	99.77	34
chr09	96.92	99.78	39
chr10	97.15	99.8	35

Table S8. Accession numbers of RNA-seq data used for B chromosome annotation.

Accession	Sample Name	Design description	Description
SRR12052977	MBSC_RNA_B73_B_1	RNA extracted from the 3rd leaf of a maize B73 +B plant, rRNA was depleted using EpiBio RiboZero kit, single-end Illumina library was prepared using ScriptSeq	RNA-Seq biological replicate 1
SRR12052976	MBSC_RNA_B73_B_2	RNA extracted from the 3rd leaf of a maize B73 +B plant, rRNA was depleted using EpiBio RiboZero kit, single-end Illumina library was prepared using ScriptSeq	RNA-Seq biological replicate 2
SRR12052975	MBSC_RNA_B73_B_3	RNA extracted from the 3rd leaf of a maize B73 +B plant, rRNA was depleted using EpiBio RiboZero kit, single-end Illumina library was prepared using ScriptSeq	RNA-Seq biological replicate 3
SRR12052974	MBSC_small_RNA_B73_B_1	RNA extracted from the 3rd leaf of a maize B73 +B plant, small RNA was extracted using Invitrogen miRvana kit, single-end Illumina library was prepared using TruSeq SmallRNA	miRNA-Seq biological replicate 1
SRR12052973	MBSC_small_RNA_B73_B_2	RNA extracted from the 3rd leaf of a maize B73 +B plant, small RNA was extracted using Invitrogen miRvana kit, single-end Illumina library was prepared using TruSeq SmallRNA	miRNA-Seq biological replicate 2
SRR12052972	MBSC_small_RNA_B73_B_3	RNA extracted from the 3rd leaf of a maize B73 +B plant, small RNA was extracted using Invitrogen miRvana kit, single-end Illumina library was prepared using TruSeq SmallRNA	miRNA-Seq biological replicate 3

Dataset S1. (separate file)
Coordinates of the scaffolds building the B pseudomolecule.

Dataset S2. (separate file)
Transposable elements identified in the sequence of the maize B chromosome.

Dataset S3. (separate file)
Expression of B chromosome genes in B73 lines without B chromosome and possessing one and six B chromosomes.

Dataset S4. (separate file)
Expression of A chromosomal genes in B73 lines without B chromosome and possessing one and six B chromosomes.

Dataset S5. (separate file)
Gene Ontology (GO) terms assigned to genes in the B chromosome sequence.

Dataset S6. (separate file)
Families of LTR retrotransposons and their abundance in the sequence of the B chromosome and the A chromosomal complement.

Dataset S7. (separate file)
Best reciprocal hits of B chromosome genes in related genomes.

Dataset S8. (separate file)
Phylogenetic trees of 198 gene sets comprising homologs in all analysed sequences; i.e. genomes of *Brachypodium distachyon*, *Oryza sativa*, *Sorghum bicolor* and *Zea mays* and the maize B chromosome sequence.

Dataset S9. (separate file)
Localization of scaffolds along the B chromosome.
