# Supplementary material for: Sequence of the supernumerary B chromosome of maize provides insight into its drive mechanism and evolution
Source: Proc Natl Acad Sci U S A. 2021 Jun 4;118(23):e2104254118. doi: 10.1073/pnas.2104254118 (PMC8201846; doi:10.1073/pnas.2104254118)
Supplement: Supplementary File [file pnas.2104254118.sd08.pdf]

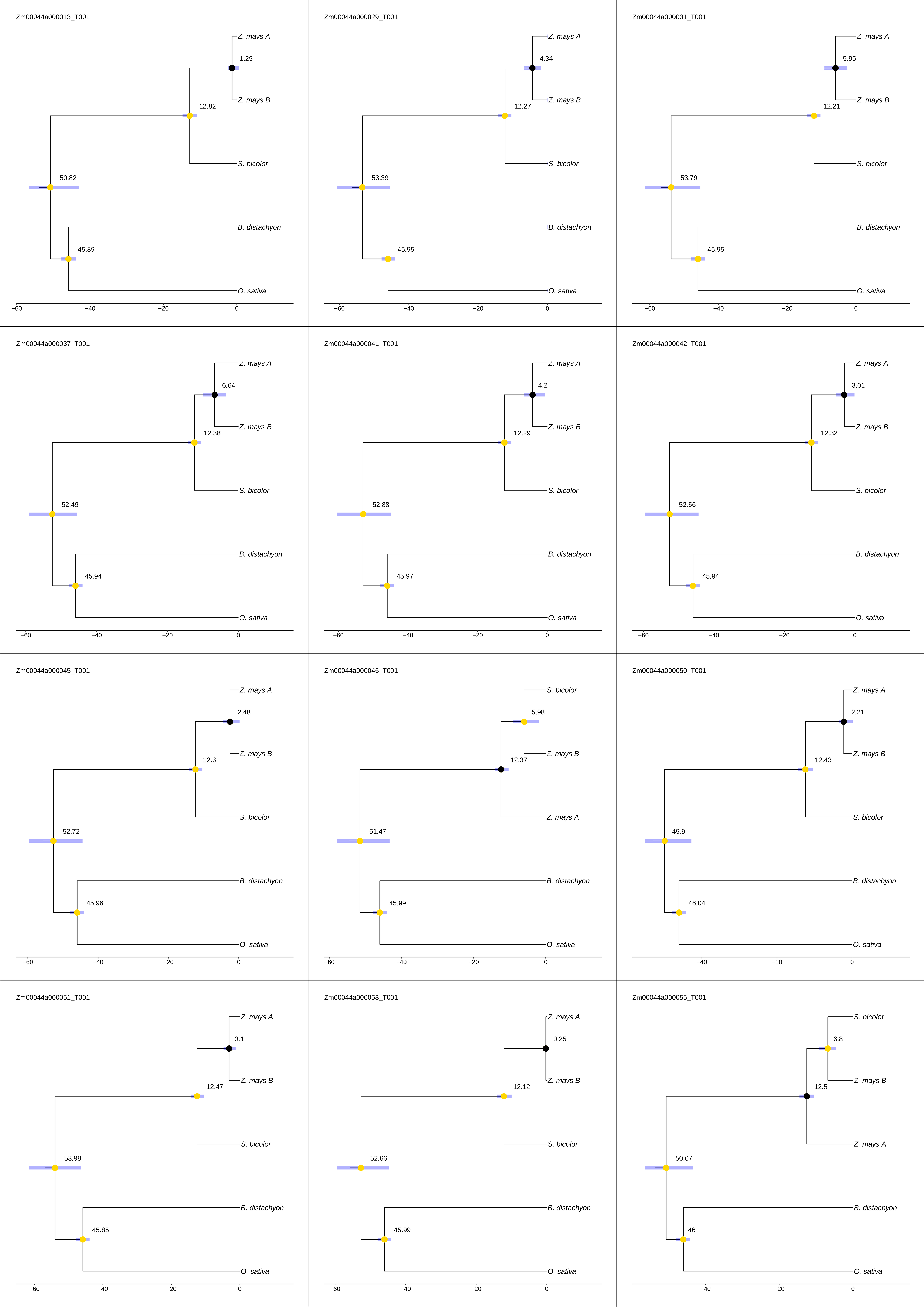

Zm00044a000013_T001
Z. mays A
1.29
Z. mays B
12.82
S. bicolor
50.82
B. distachyon
45.89
O. sativa
−60 −40 −20 0
Zm00044a000029_T001
Z. mays A
4.34
Z. mays B
12.27
S. bicolor
53.39
B. distachyon
45.95
O. sativa
−60 −40 −20 0
Zm00044a000031_T001
Z. mays A
5.95
Z. mays B
12.21
S. bicolor
53.79
B. distachyon
45.95
O. sativa
−60 −40 −20 0
Zm00044a000037_T001
Z. mays A
6.64
Z. mays B
12.38
S. bicolor
52.49
B. distachyon
45.94
O. sativa
−60 −40 −20 0
Zm00044a000041_T001
Z. mays A
4.2
Z. mays B
12.29
S. bicolor
52.88
B. distachyon
45.97
O. sativa
−60 −40 −20 0
Zm00044a000042_T001
Z. mays A
3.01
Z. mays B
12.32
S. bicolor
52.56
B. distachyon
45.94
O. sativa
−60 −40 −20 0
Zm00044a000045_T001
Z. mays A
2.48
Z. mays B
12.3
S. bicolor
52.72
B. distachyon
45.96
O. sativa
−60 −40 −20 0
Zm00044a000046_T001
S. bicolor
5.98
Z. mays B
12.37
Z. mays A
51.47
B. distachyon
45.99
O. sativa
−60 −40 −20 0
Zm00044a000050_T001
Z. mays A
2.21
Z. mays B
12.43
S. bicolor
49.9
B. distachyon
46.04
O. sativa
−40 −20 0
Zm00044a000051_T001
Z. mays A
3.1
Z. mays B
12.47
S. bicolor
53.98
B. distachyon
45.85
O. sativa
−60 −40 −20 0
Zm00044a000053_T001
Z. mays A
0.25
Z. mays B
12.12
S. bicolor
52.66
B. distachyon
45.99
O. sativa
−60 −40 −20 0
Zm00044a000055_T001
S. bicolor
6.8
Z. mays B
12.5
Z. mays A
50.67
B. distachyon
46
O. sativa
−40 −20 0

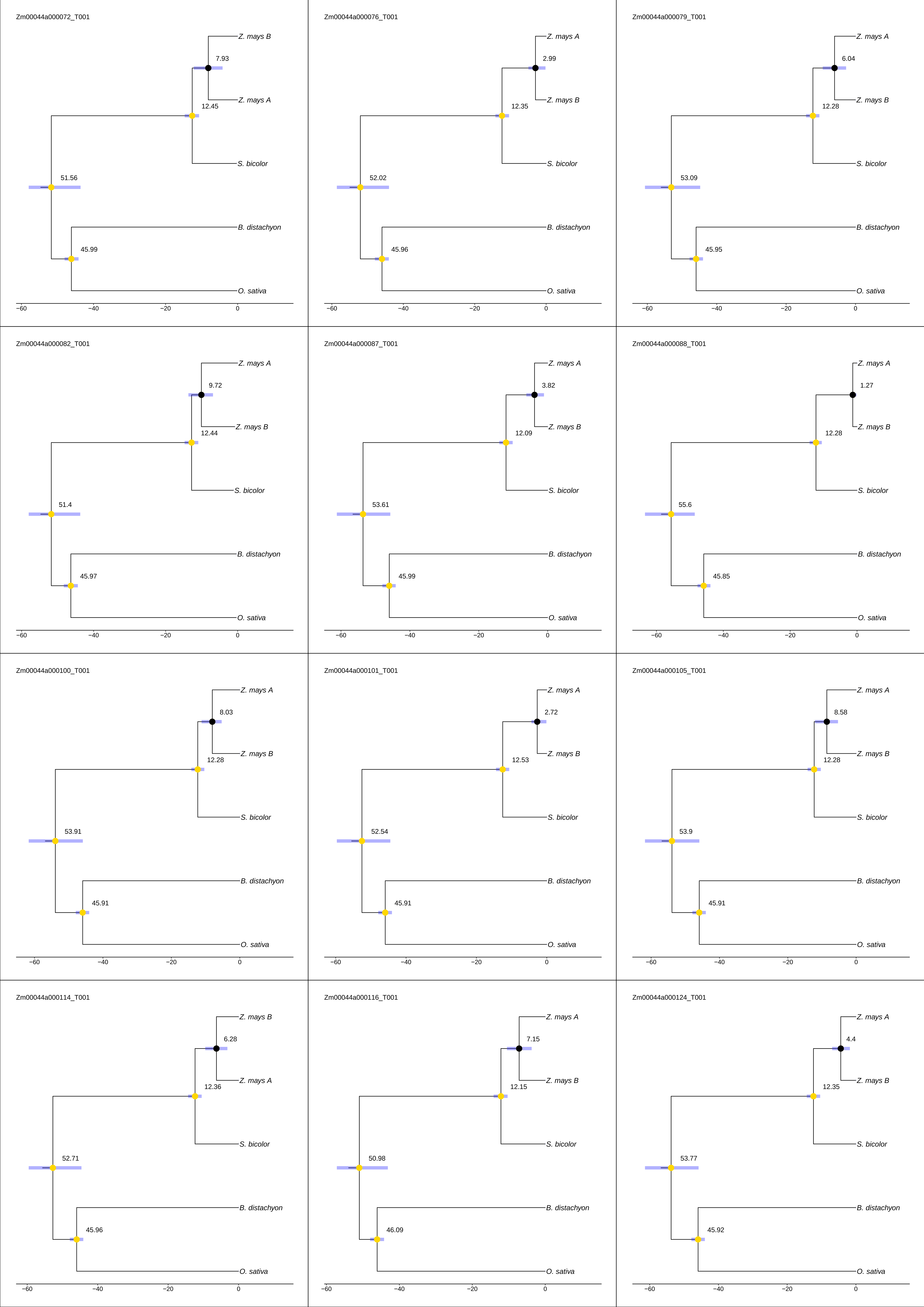

Zm00044a000072_T001
Z. mays B
7.93
Z. mays A
12.45
S. bicolor
51.56
B. distachyon
45.99
O. sativa
−60 −40 −20 0
Zm00044a000076_T001
Z. mays A
2.99
Z. mays B
12.35
S. bicolor
52.02
B. distachyon
45.96
O. sativa
−60 −40 −20 0
Zm00044a000079_T001
Z. mays A
6.04
Z. mays B
12.28
S. bicolor
53.09
B. distachyon
45.95
O. sativa
−60 −40 −20 0
Zm00044a000082_T001
Z. mays A
9.72
Z. mays B
12.44
S. bicolor
51.4
B. distachyon
45.97
O. sativa
−60 −40 −20 0
Zm00044a000087_T001
Z. mays A
3.82
Z. mays B
12.09
S. bicolor
53.61
B. distachyon
45.99
O. sativa
−60 −40 −20 0
Zm00044a000088_T001
Z. mays A
1.27
Z. mays B
12.28
S. bicolor
55.6
B. distachyon
45.85
O. sativa
−60 −40 −20 0
Zm00044a000100_T001
Z. mays A
8.03
Z. mays B
12.28
S. bicolor
53.91
B. distachyon
45.91
O. sativa
−60 −40 −20 0
Zm00044a000101_T001
Z. mays A
2.72
Z. mays B
12.53
S. bicolor
52.54
B. distachyon
45.91
O. sativa
−60 −40 −20 0
Zm00044a000105_T001
Z. mays A
8.58
Z. mays B
12.28
S. bicolor
53.9
B. distachyon
45.91
O. sativa
−60 −40 −20 0
Zm00044a000114_T001
Z. mays B
6.28
Z. mays A
12.36
S. bicolor
52.71
B. distachyon
45.96
O. sativa
−60 −40 −20 0
Zm00044a000116_T001
Z. mays A
7.15
Z. mays B
12.15
S. bicolor
50.98
B. distachyon
46.09
O. sativa
−60 −40 −20 0
Zm00044a000124_T001
Z. mays A
4.4
Z. mays B
12.35
S. bicolor
53.77
B. distachyon
45.92
O. sativa
−60 −40 −20 0

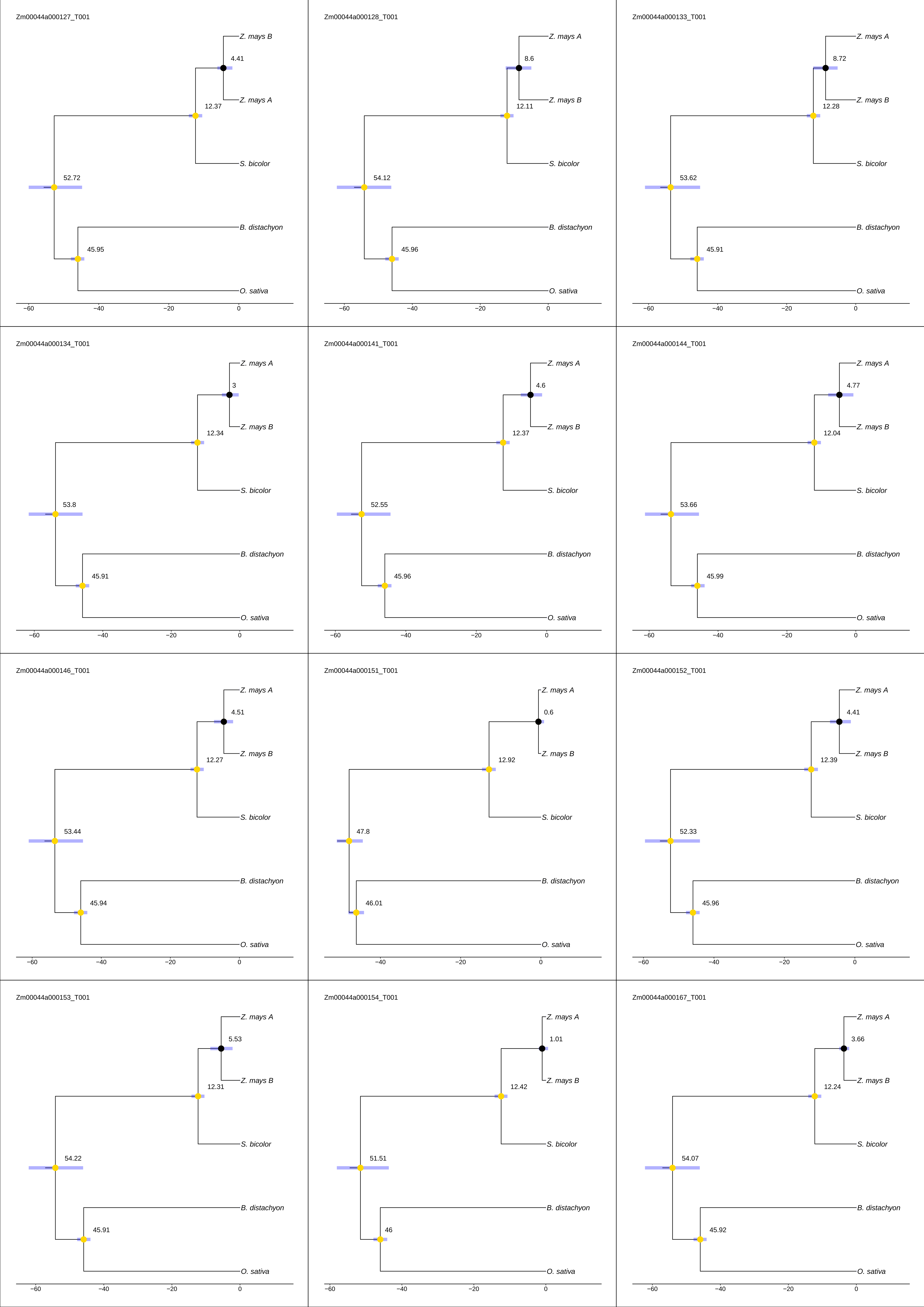
Zm00044a000127_T001
Z. mays B
4.41
Z. mays A
12.37
S. bicolor
52.72
B. distachyon
45.95
O. sativa
−60 −40 −20 0
Zm00044a000128_T001
Z. mays A
8.6
Z. mays B
12.11
S. bicolor
54.12
B. distachyon
45.96
O. sativa
−60 −40 −20 0
Zm00044a000133_T001
Z. mays A
8.72
Z. mays B
12.28
S. bicolor
53.62
B. distachyon
45.91
O. sativa
−60 −40 −20 0
Zm00044a000134_T001
Z. mays A
3
Z. mays B
12.34
S. bicolor
53.8
B. distachyon
45.91
O. sativa
−60 −40 −20 0
Zm00044a000141_T001
Z. mays A
4.6
Z. mays B
12.37
S. bicolor
52.55
B. distachyon
45.96
O. sativa
−60 −40 −20 0
Zm00044a000144_T001
Z. mays A
4.77
Z. mays B
12.04
S. bicolor
53.66
B. distachyon
45.99
O. sativa
−60 −40 −20 0
Zm00044a000146_T001
Z. mays A
4.51
Z. mays B
12.27
S. bicolor
53.44
B. distachyon
45.94
O. sativa
−60 −40 −20 0
Zm00044a000151_T001
Z. mays A
0.6
Z. mays B
12.92
S. bicolor
47.8
B. distachyon
46.01
O. sativa
−40 −20 0
Zm00044a000152_T001
Z. mays A
4.41
Z. mays B
12.39
S. bicolor
52.33
B. distachyon
45.96
O. sativa
−60 −40 −20 0
Zm00044a000153_T001
Z. mays A
5.53
Z. mays B
12.31
S. bicolor
54.22
B. distachyon
45.91
O. sativa
−60 −40 −20 0
Zm00044a000154_T001
Z. mays A
1.01
Z. mays B
12.42
S. bicolor
51.51
B. distachyon
46
O. sativa
−60 −40 −20 0
Zm00044a000167_T001
Z. mays A
3.66
Z. mays B
12.24
S. bicolor
54.07
B. distachyon
45.92
O. sativa
−60 −40 −20 0

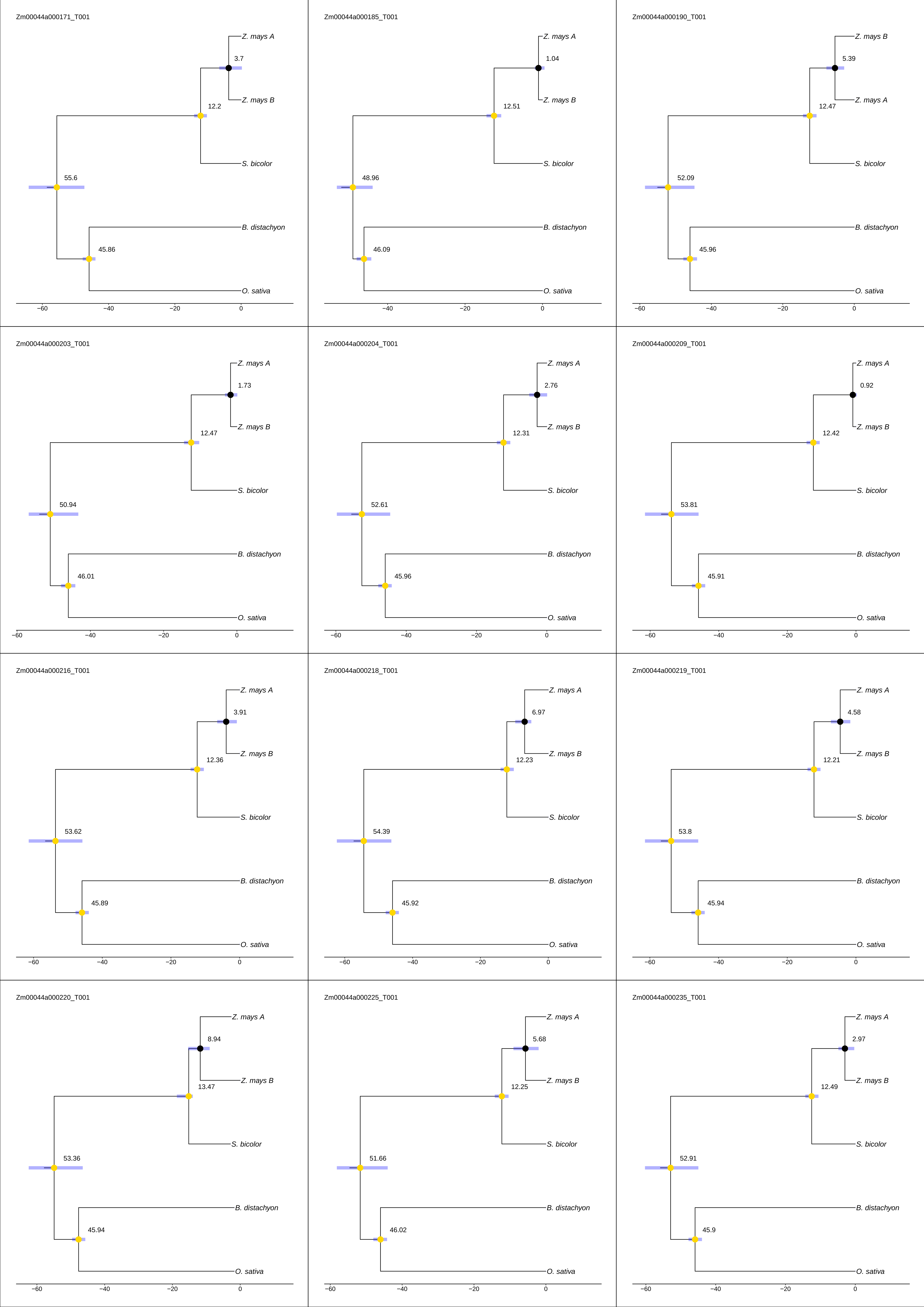
Zm00044a000171_T001
Z. mays A
3.7
Z. mays B
12.2
S. bicolor
55.6
B. distachyon
45.86
O. sativa
−60
−40
−20
0
Zm00044a000185_T001
Z. mays A
1.04
Z. mays B
12.51
S. bicolor
48.96
B. distachyon
46.09
O. sativa
−40
−20
0
Zm00044a000190_T001
Z. mays B
5.39
Z. mays A
12.47
S. bicolor
52.09
B. distachyon
45.96
O. sativa
−60
−40
−20
0
Zm00044a000203_T001
Z. mays A
1.73
Z. mays B
12.47
S. bicolor
50.94
B. distachyon
46.01
O. sativa
−60
−40
−20
0
Zm00044a000204_T001
Z. mays A
2.76
Z. mays B
12.31
S. bicolor
52.61
B. distachyon
45.96
O. sativa
−60
−40
−20
0
Zm00044a000209_T001
Z. mays A
0.92
Z. mays B
12.42
S. bicolor
53.81
B. distachyon
45.91
O. sativa
−60
−40
−20
0
Zm00044a000216_T001
Z. mays A
3.91
Z. mays B
12.36
S. bicolor
53.62
B. distachyon
45.89
O. sativa
−60
−40
−20
0
Zm00044a000218_T001
Z. mays A
6.97
Z. mays B
12.23
S. bicolor
54.39
B. distachyon
45.92
O. sativa
−60
−40
−20
0
Zm00044a000219_T001
Z. mays A
4.58
Z. mays B
12.21
S. bicolor
53.8
B. distachyon
45.94
O. sativa
−60
−40
−20
0
Zm00044a000220_T001
Z. mays A
8.94
Z. mays B
13.47
S. bicolor
53.36
B. distachyon
45.94
O. sativa
−60
−40
−20
0
Zm00044a000225_T001
Z. mays A
5.68
Z. mays B
12.25
S. bicolor
51.66
B. distachyon
46.02
O. sativa
−60
−40
−20
0
Zm00044a000235_T001
Z. mays A
2.97
Z. mays B
12.49
S. bicolor
52.91
B. distachyon
45.9
O. sativa
−60
−40
−20
0

Zm00044a000237_T001

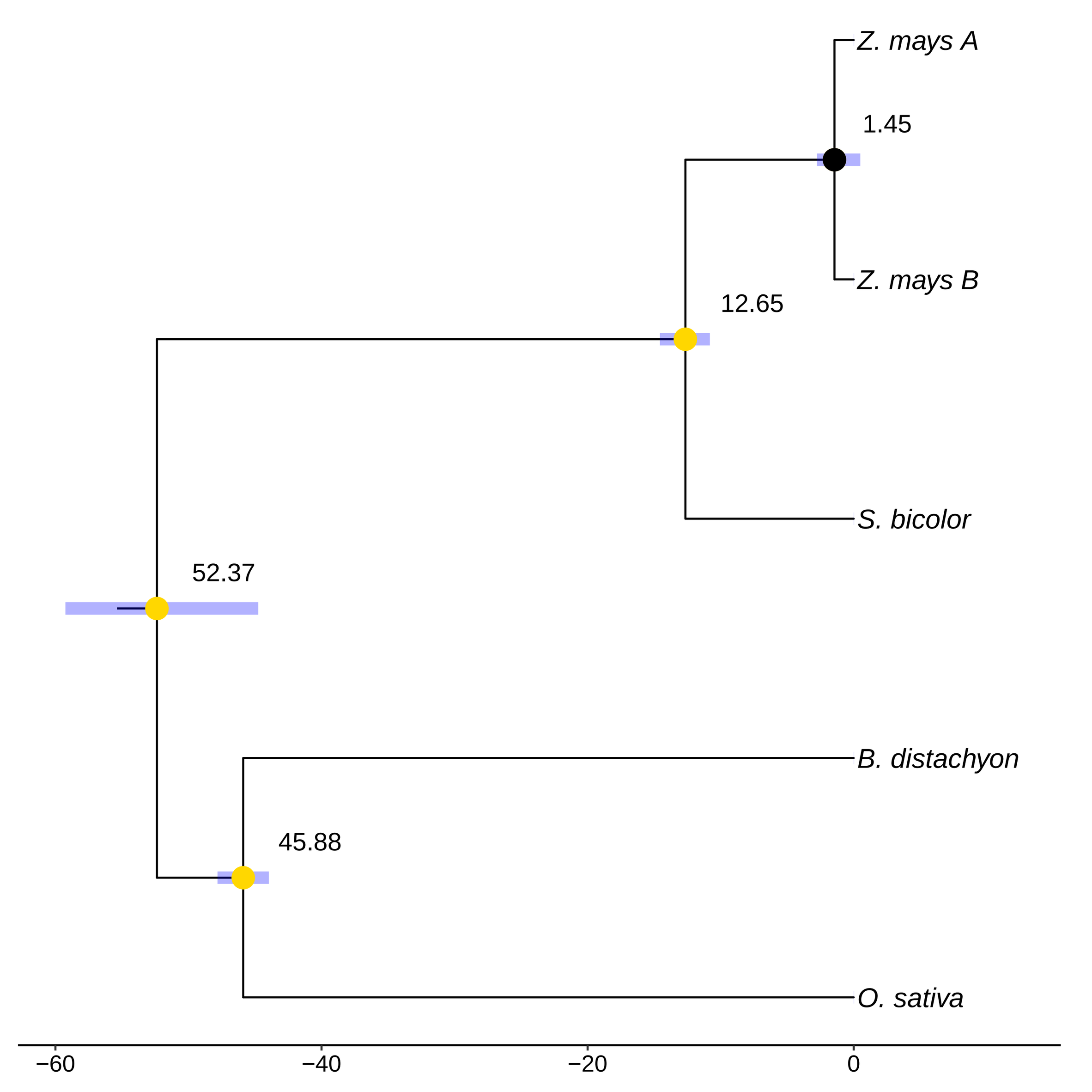

Zm00044a000239_T001

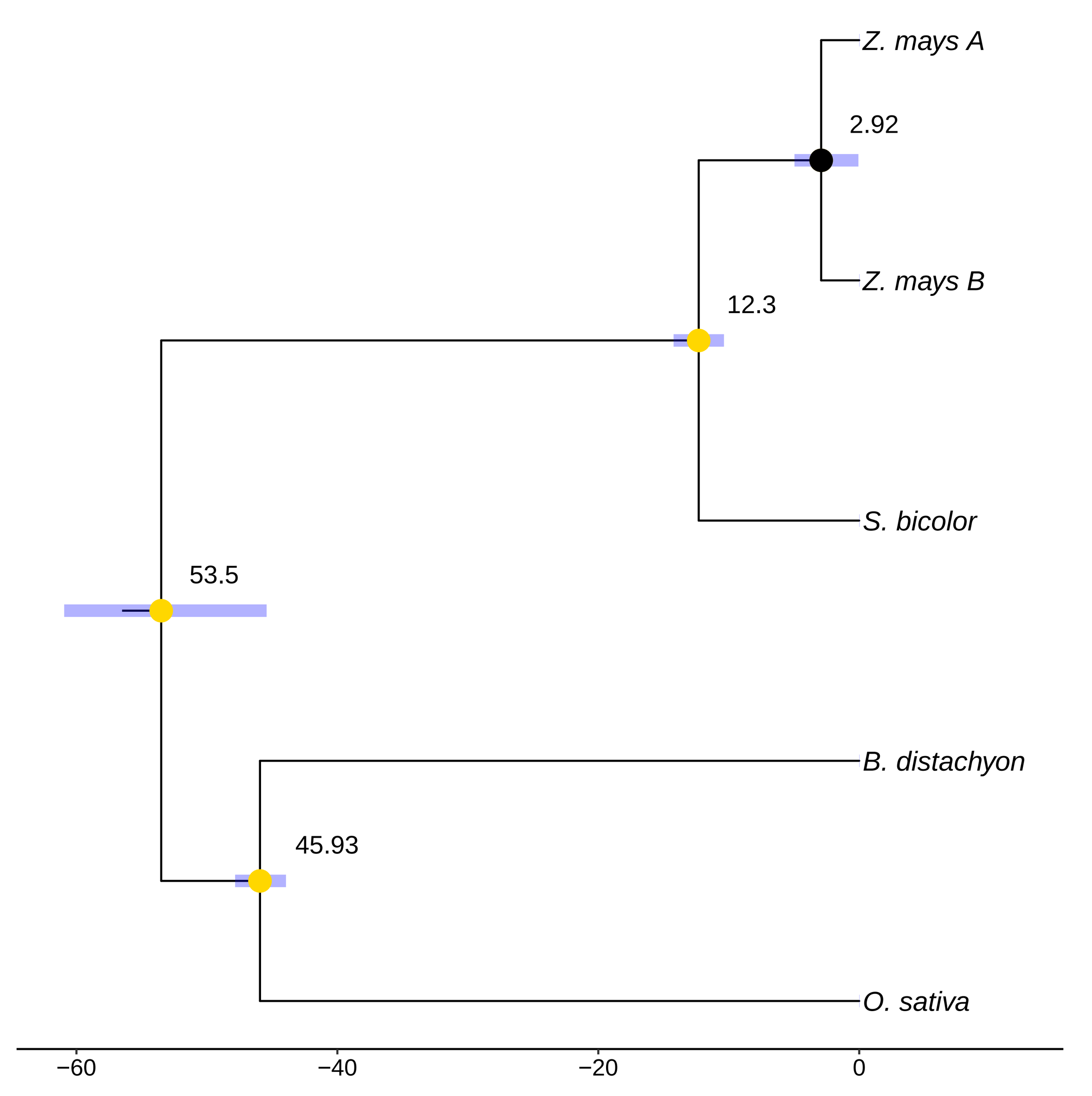

Zm00044a000243_T001

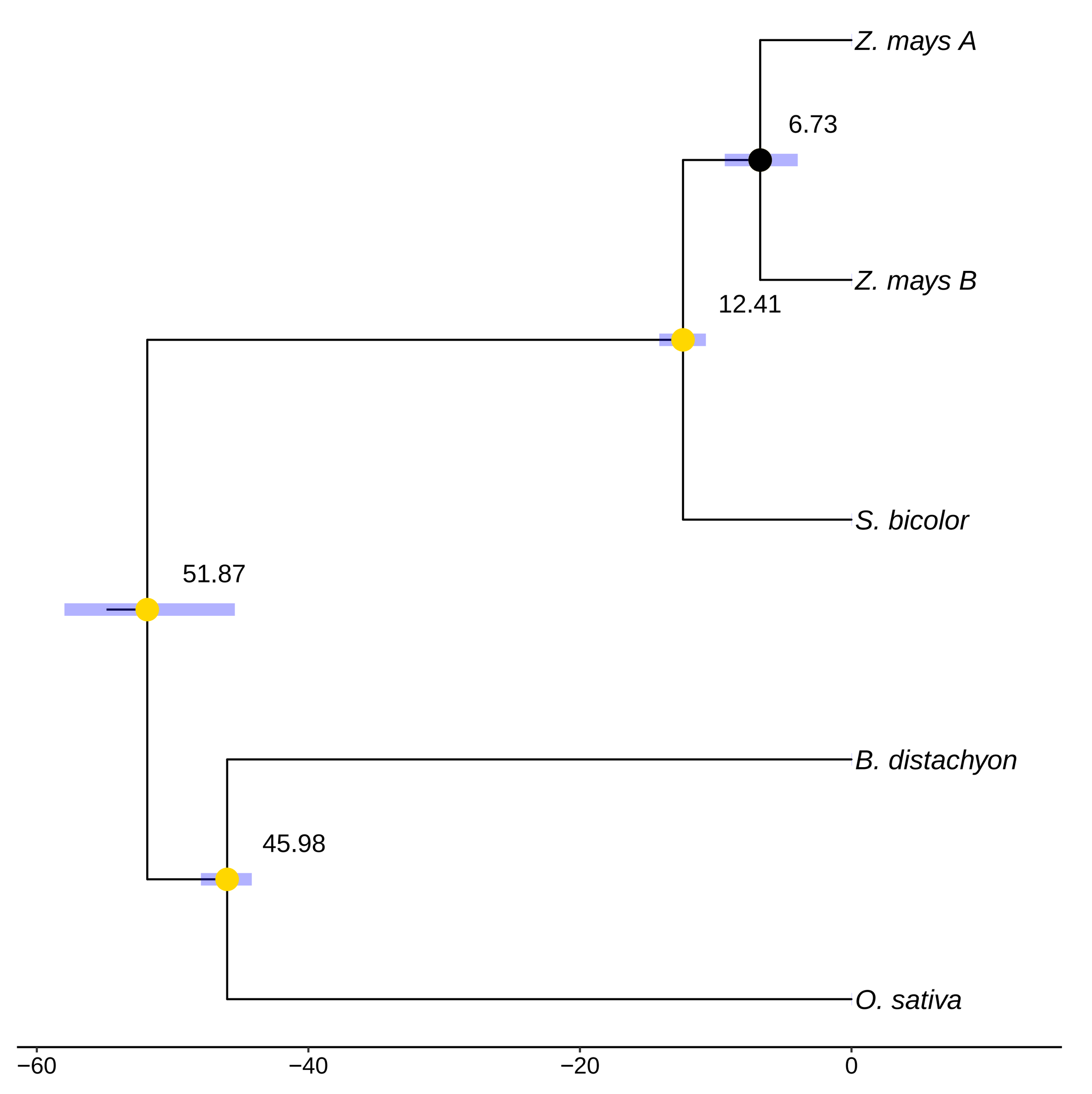

Zm00044a000247_T001

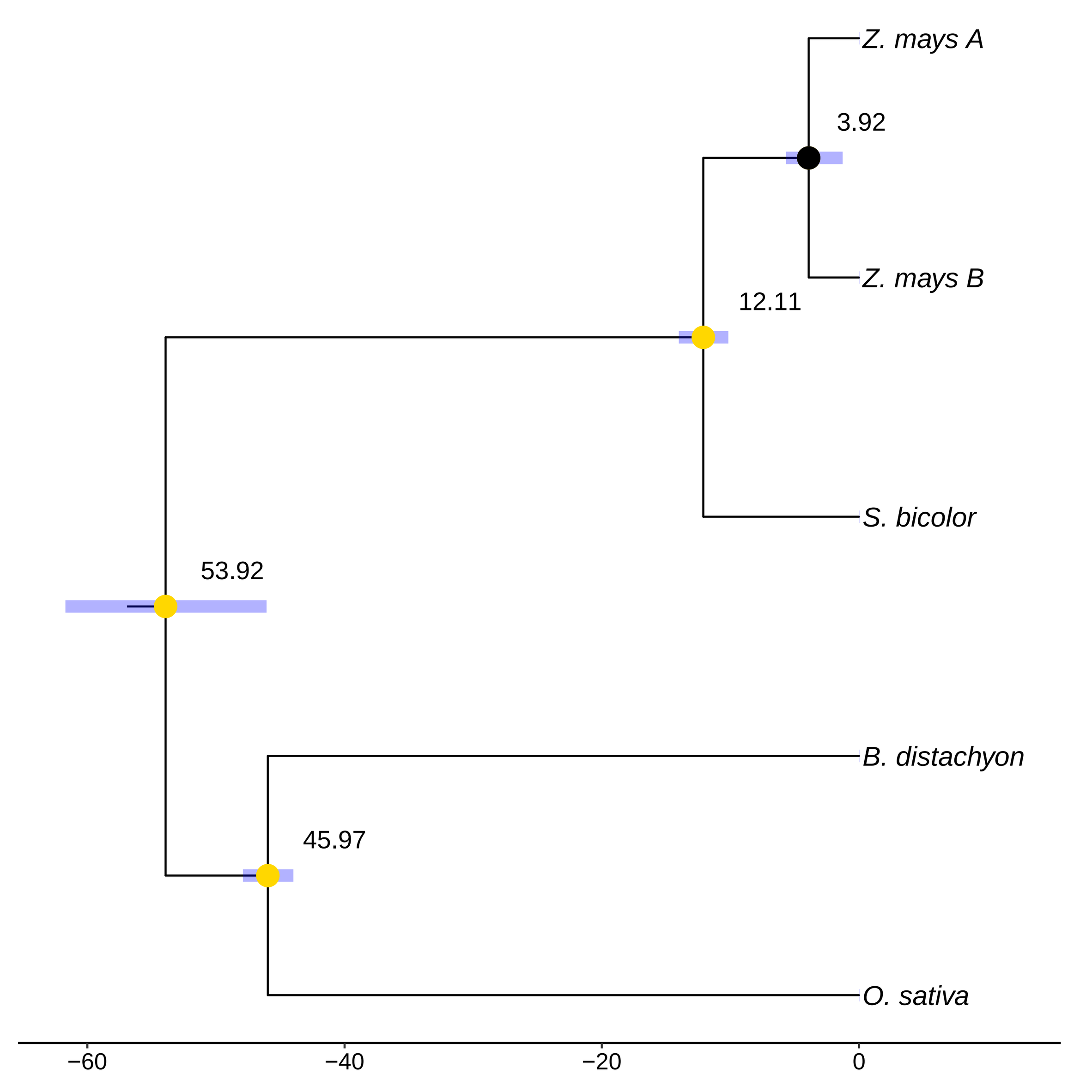

Zm00044a000259_T001

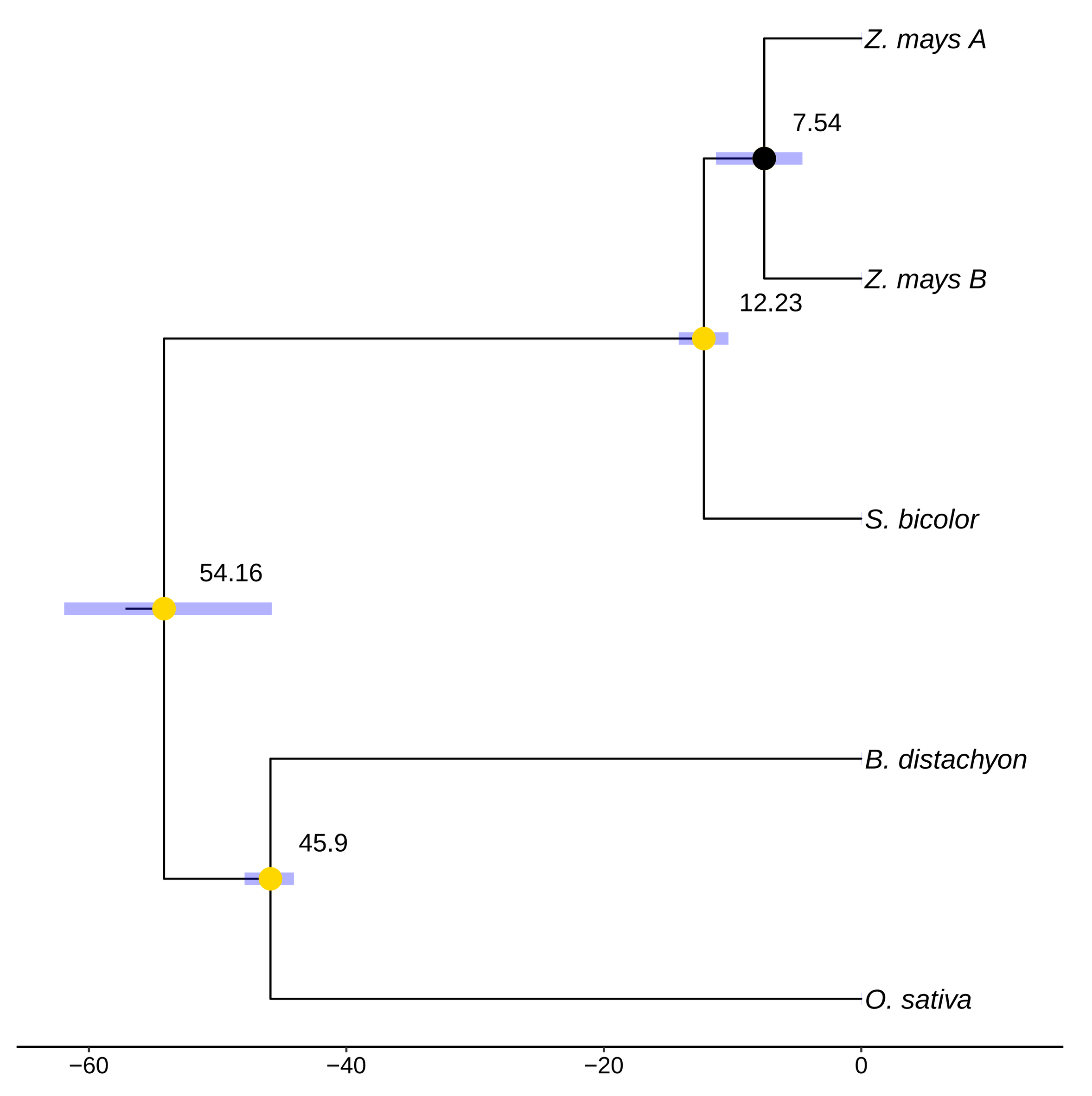

Zm00044a000261_T001

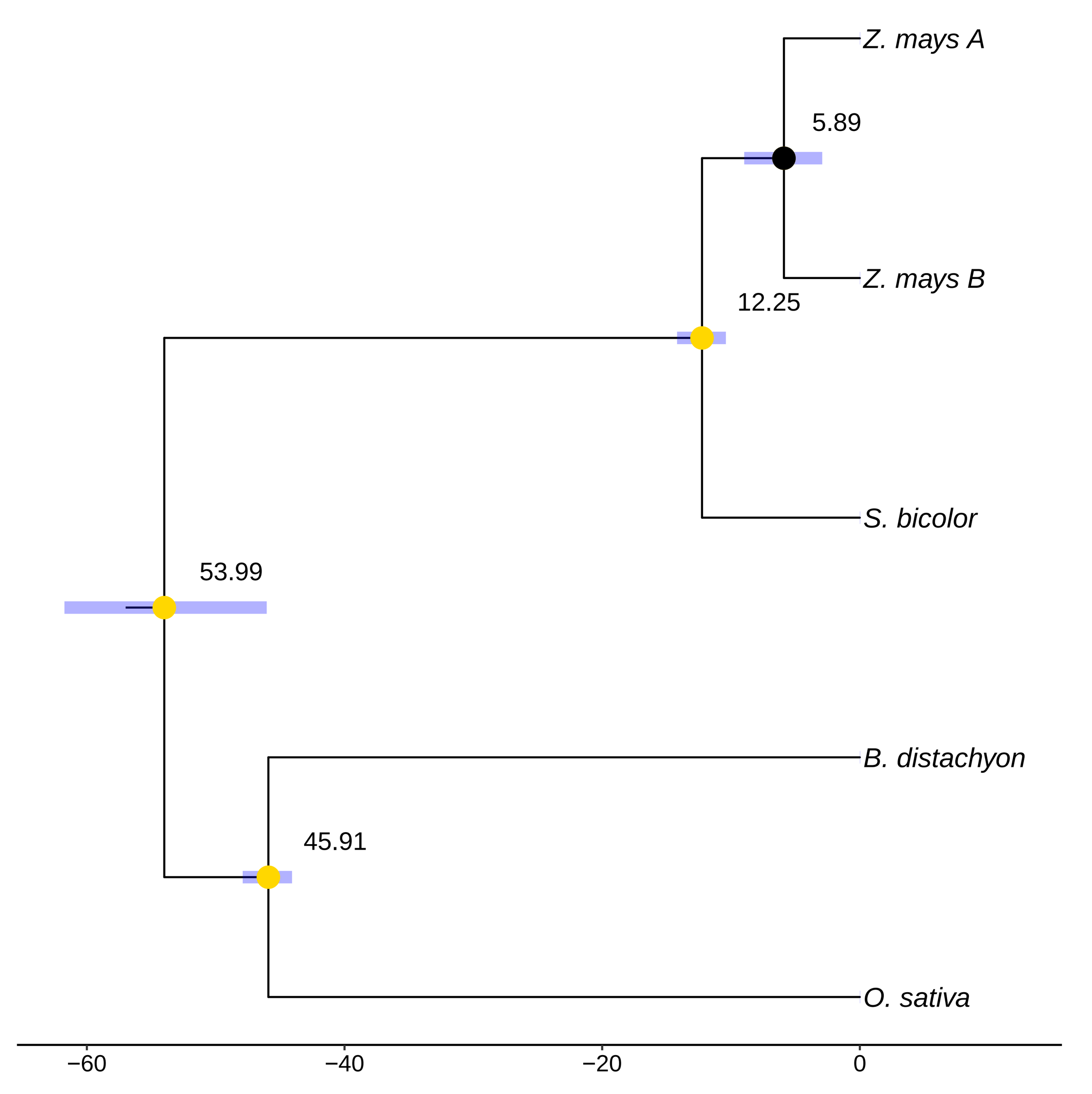

Zm00044a000265_T001

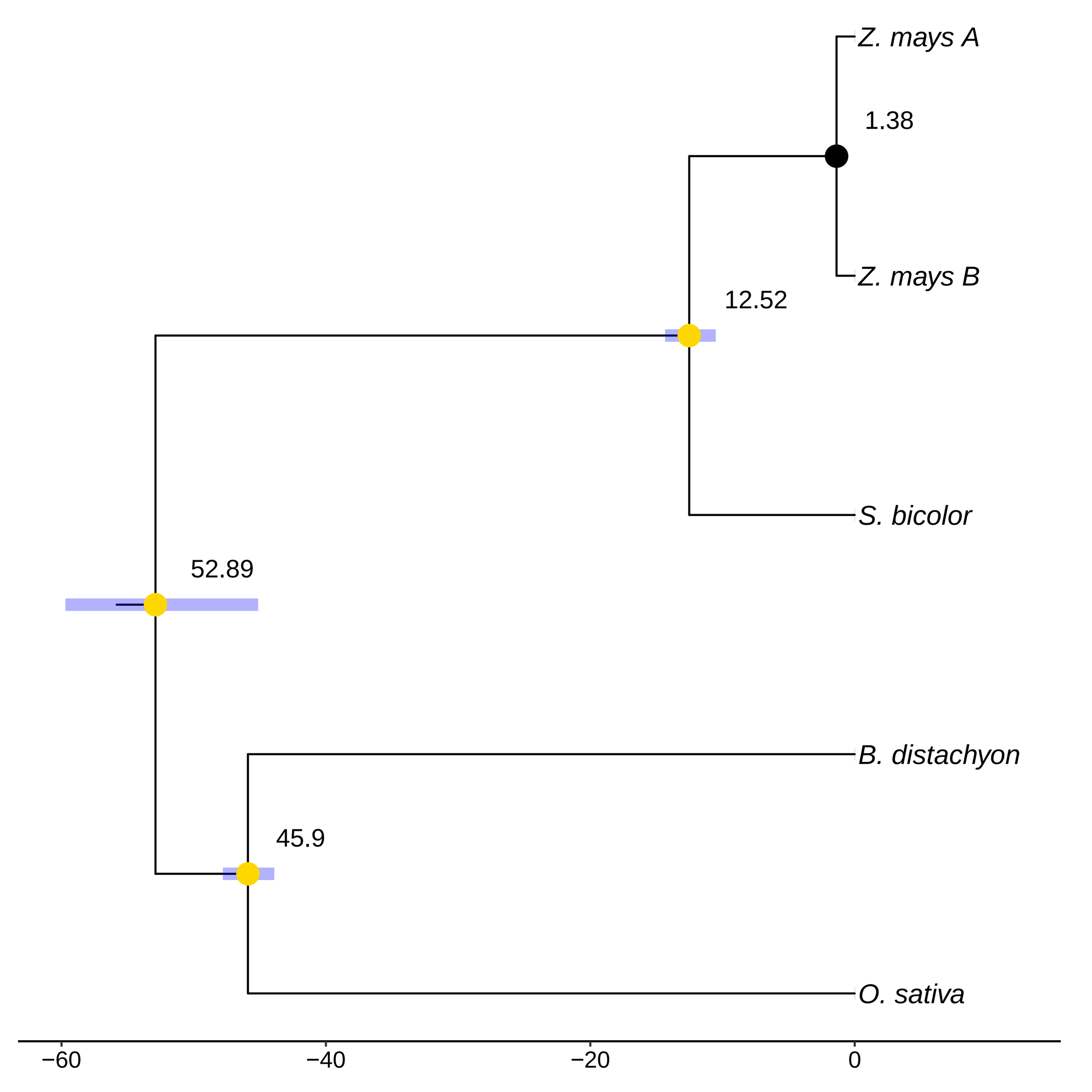

Zm00044a000266_T001

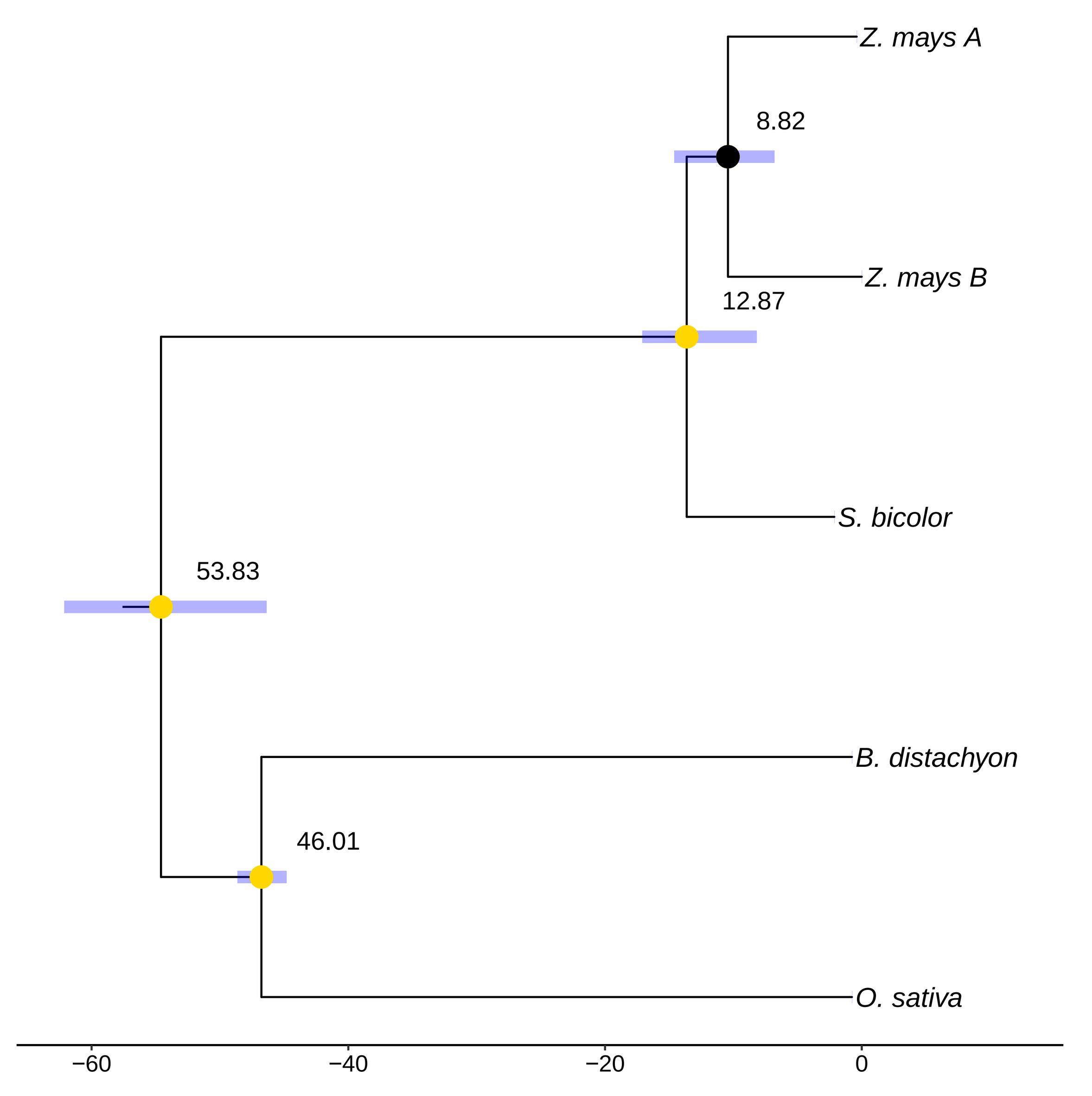

Zm00044a000271_T001

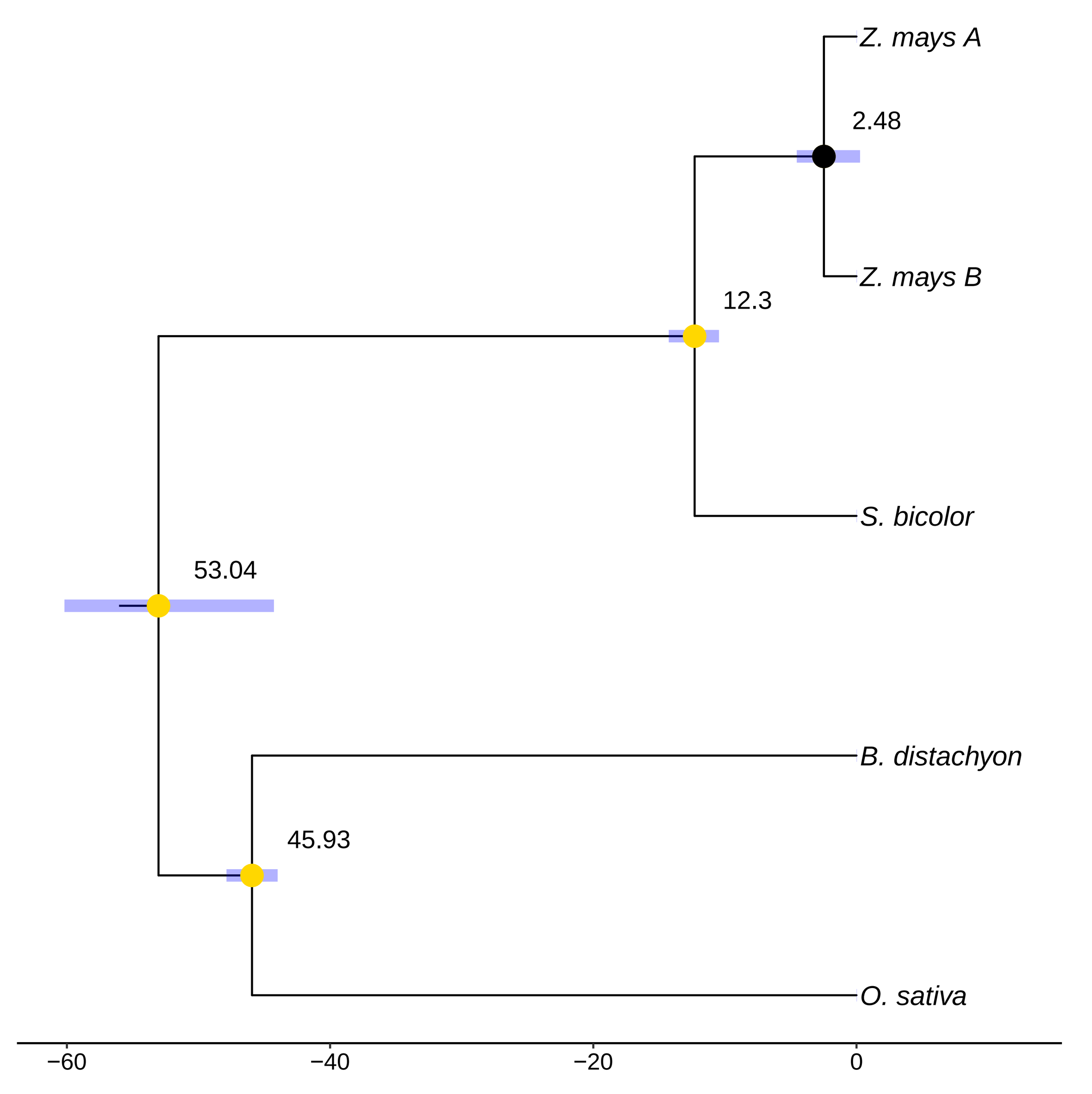

Zm00044a000273_T001

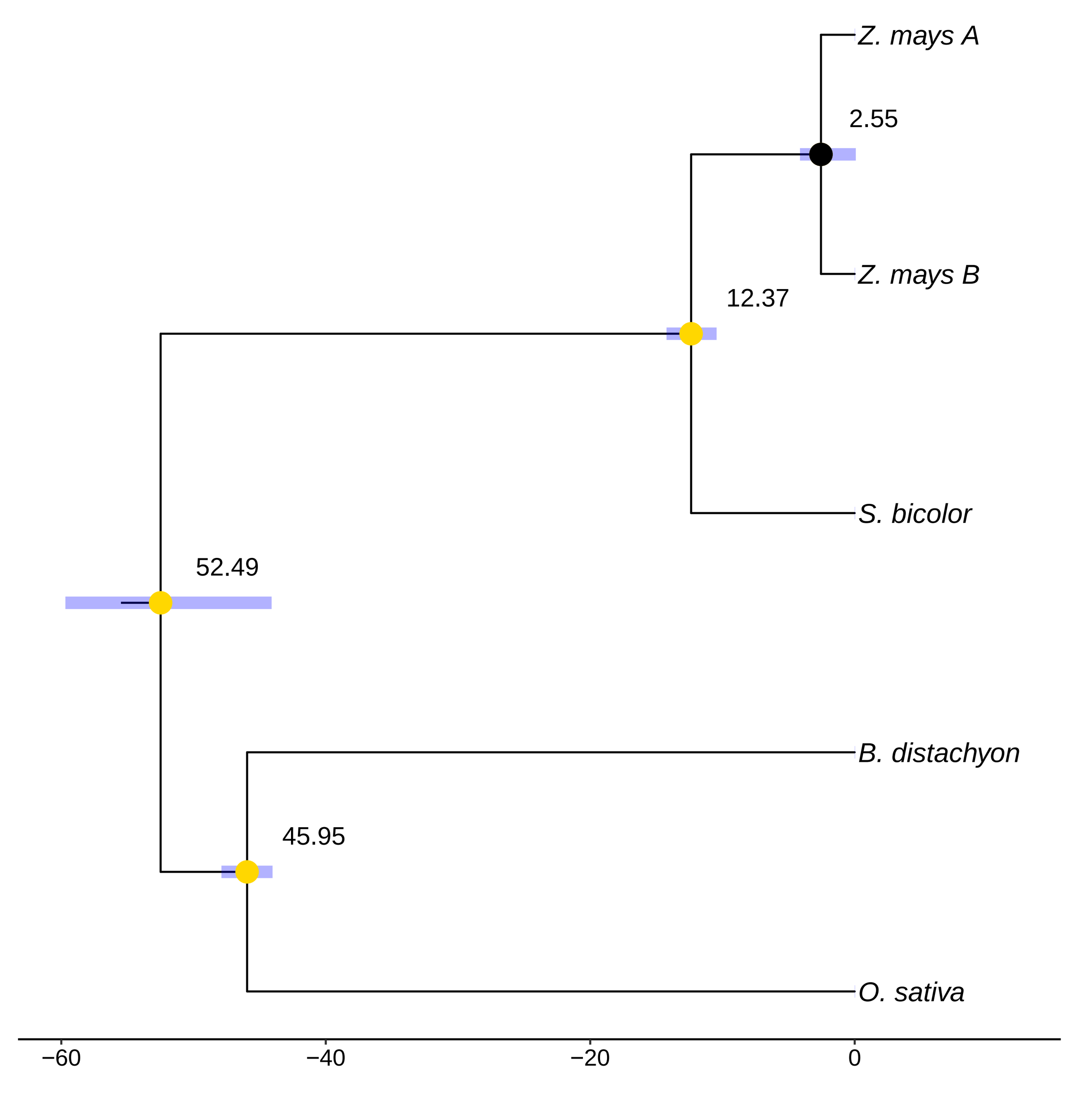

Zm00044a000276_T001

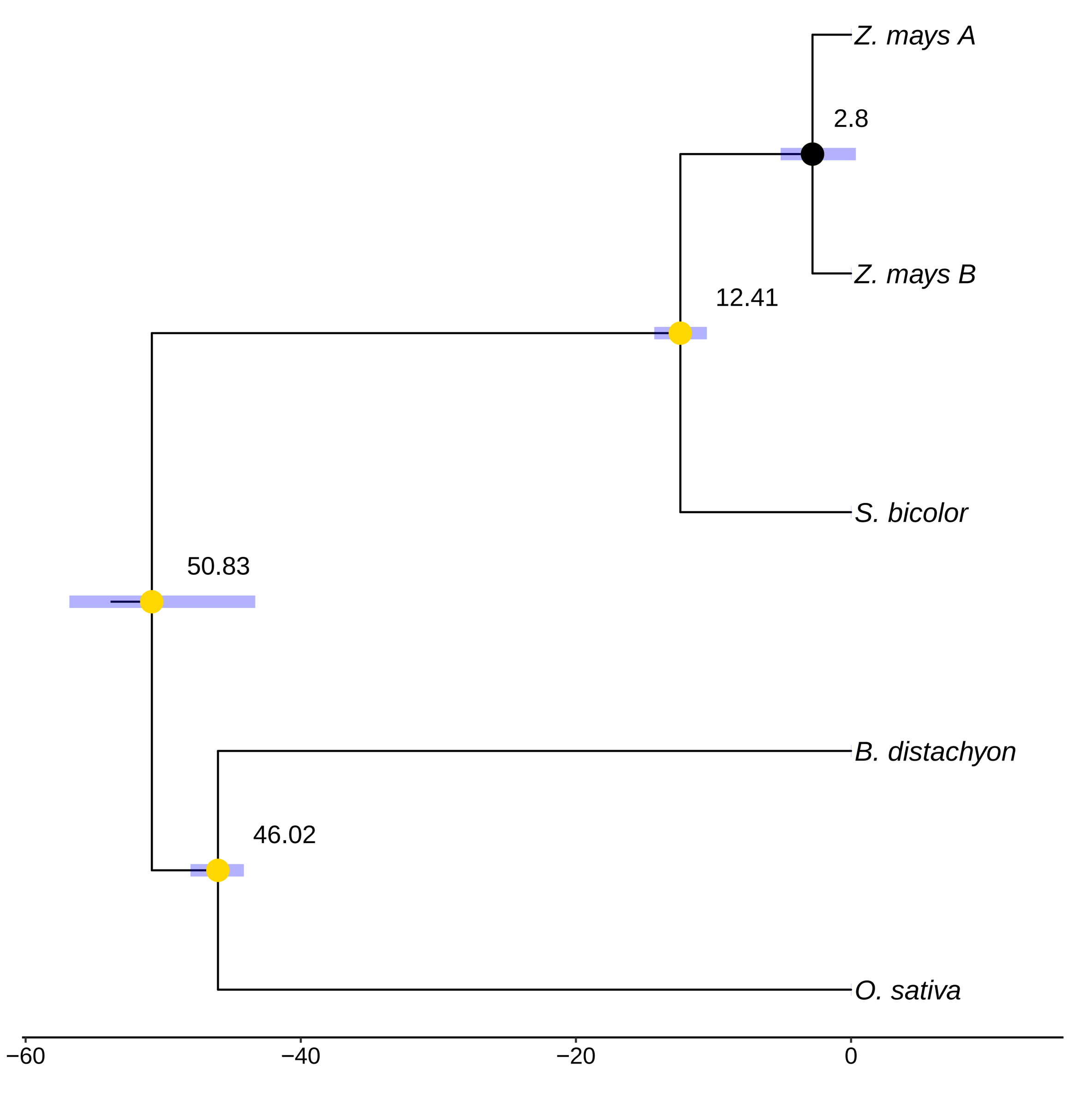

Zm00044a000277_T001

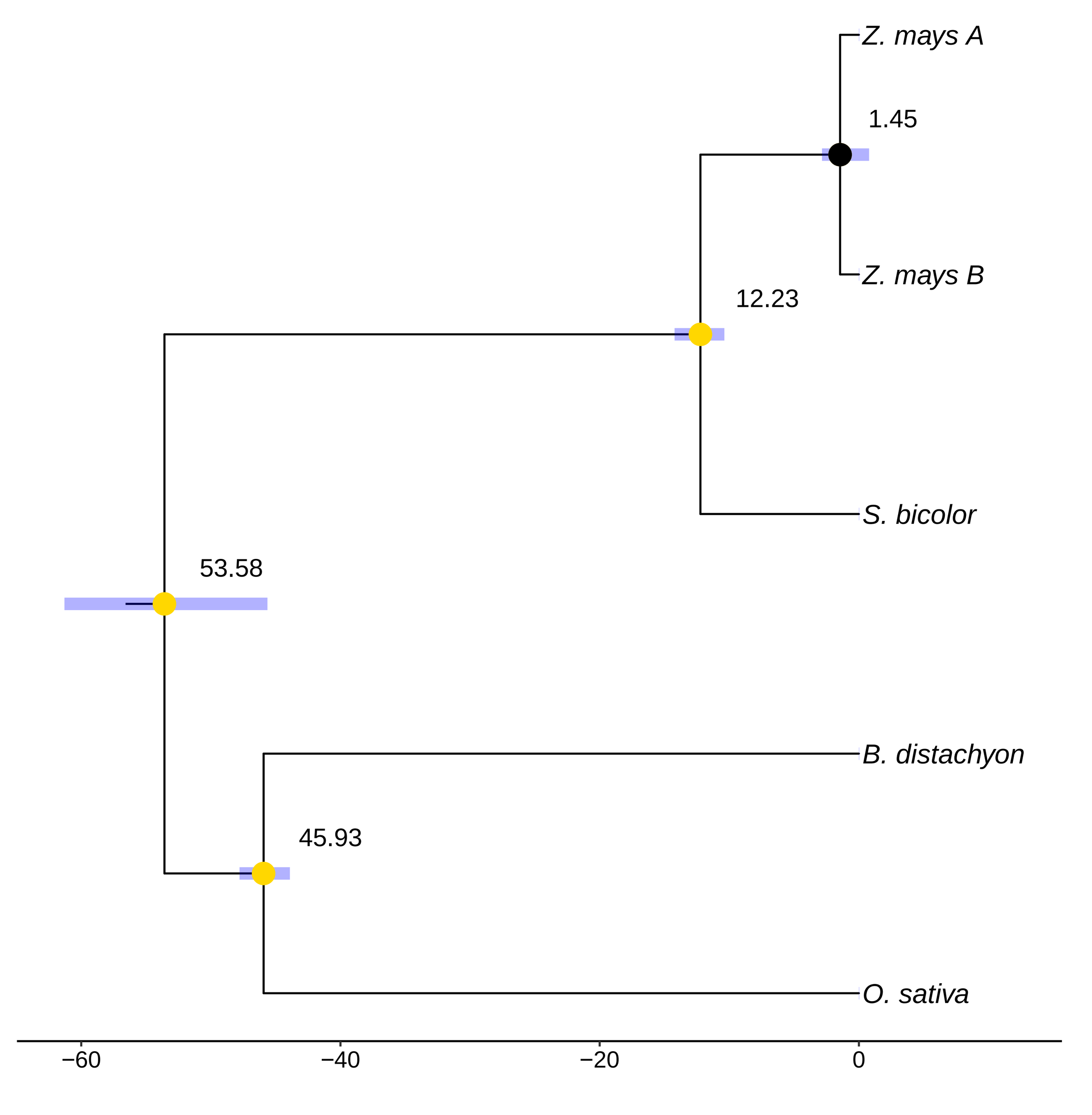

Zm00044a000279_T001

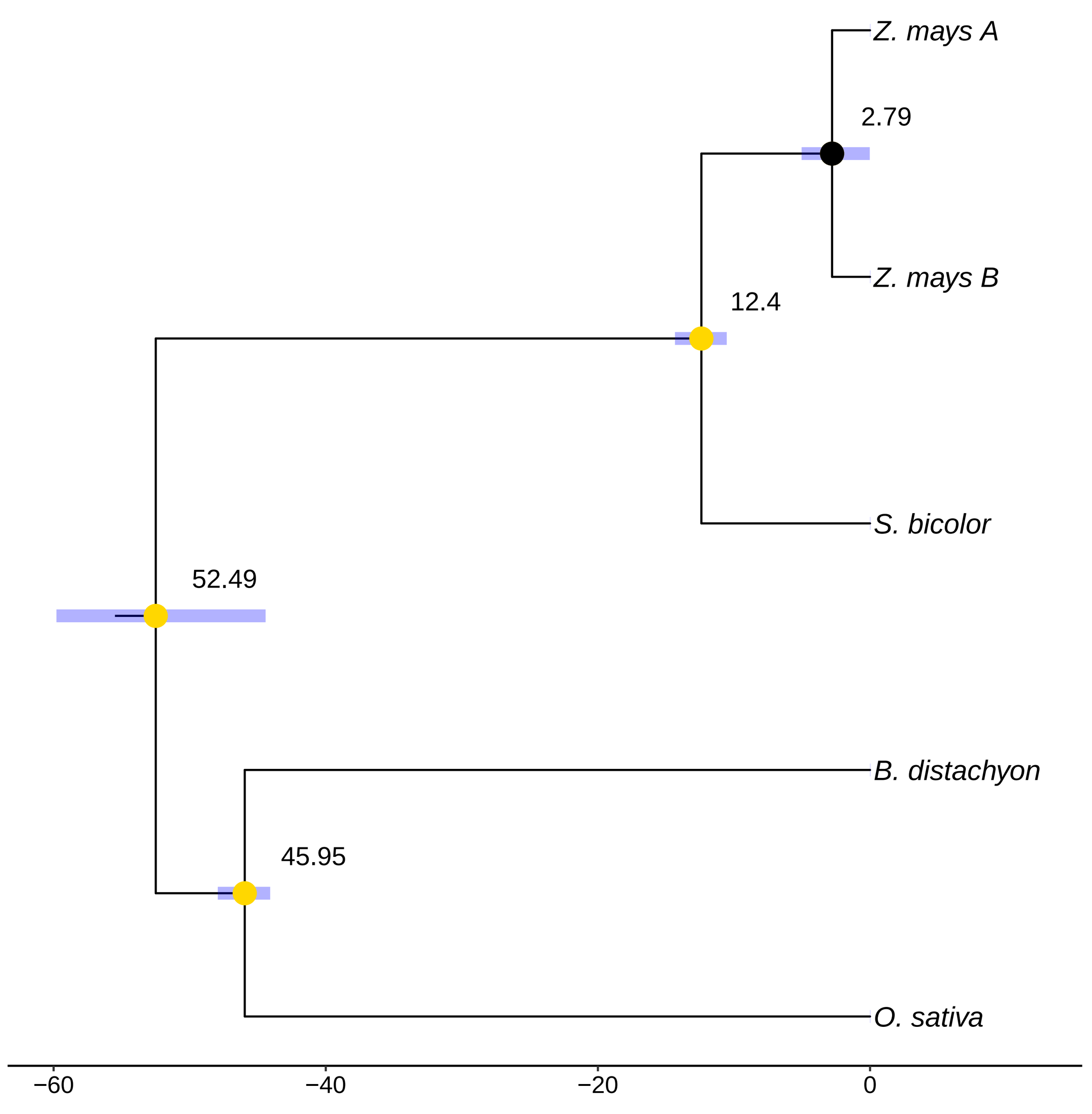

Zm00044a000281_T001

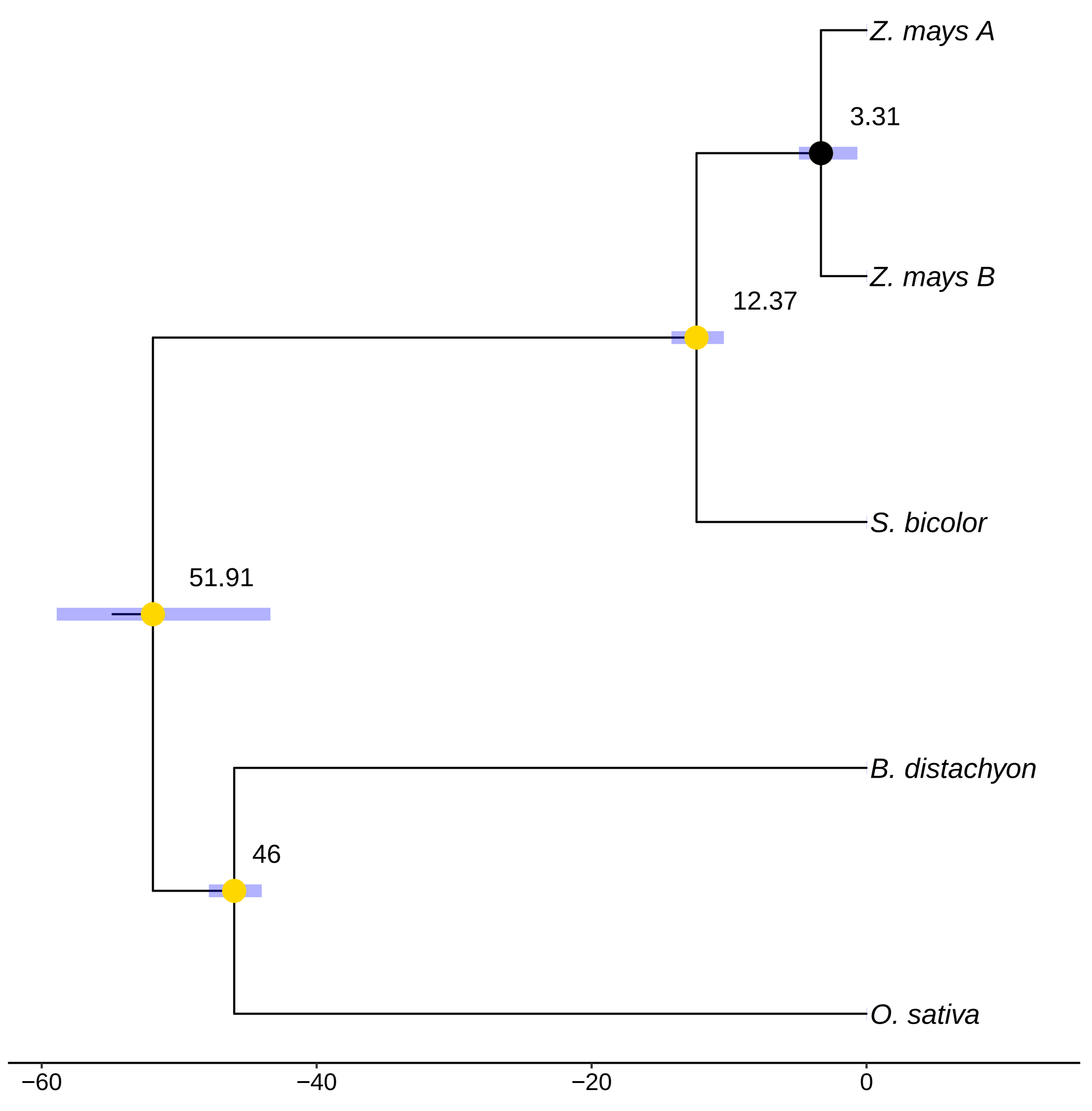

Zm00044a000283_T001

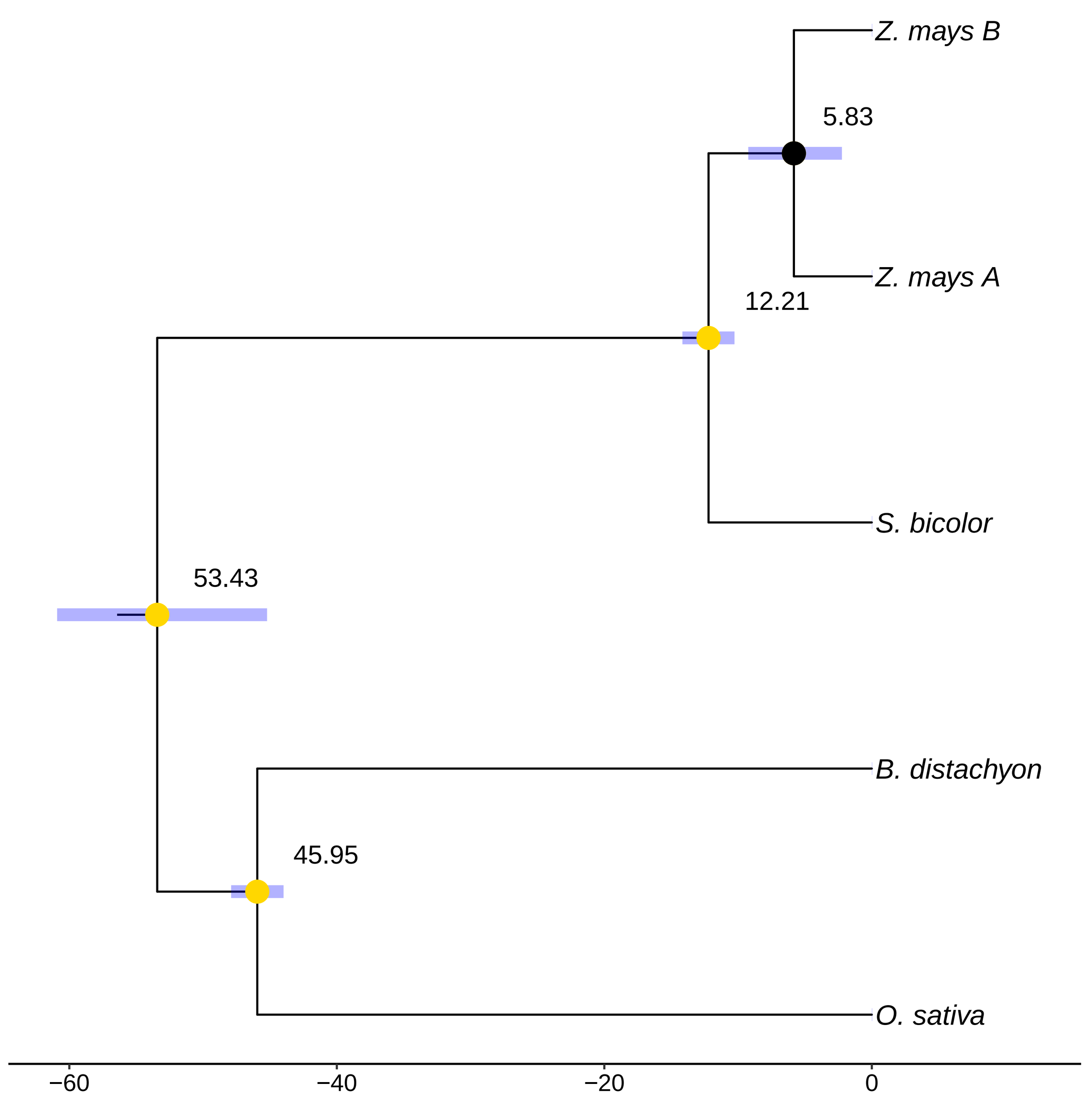

Zm00044a000288_T001

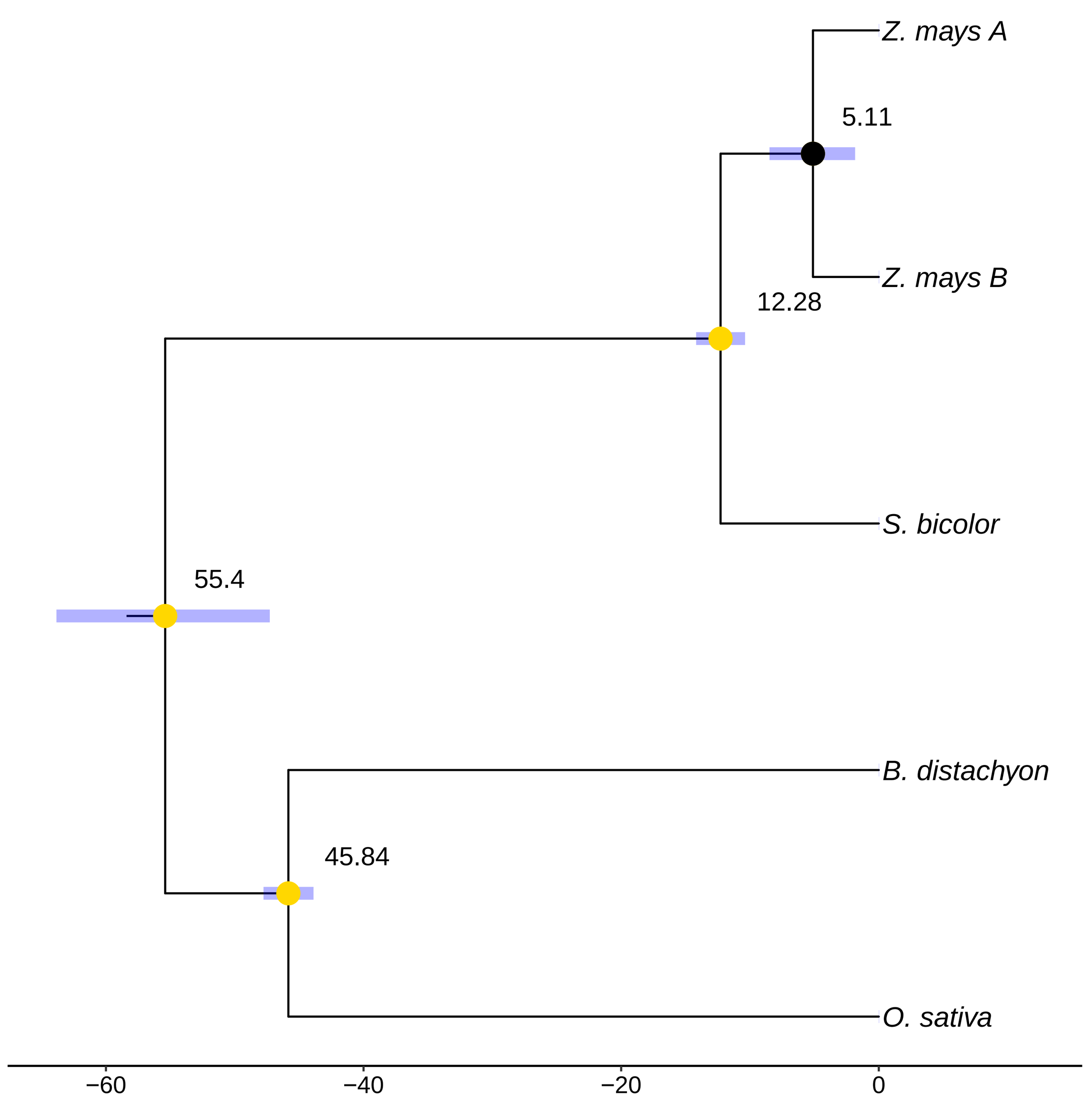

Zm00044a000289_T001

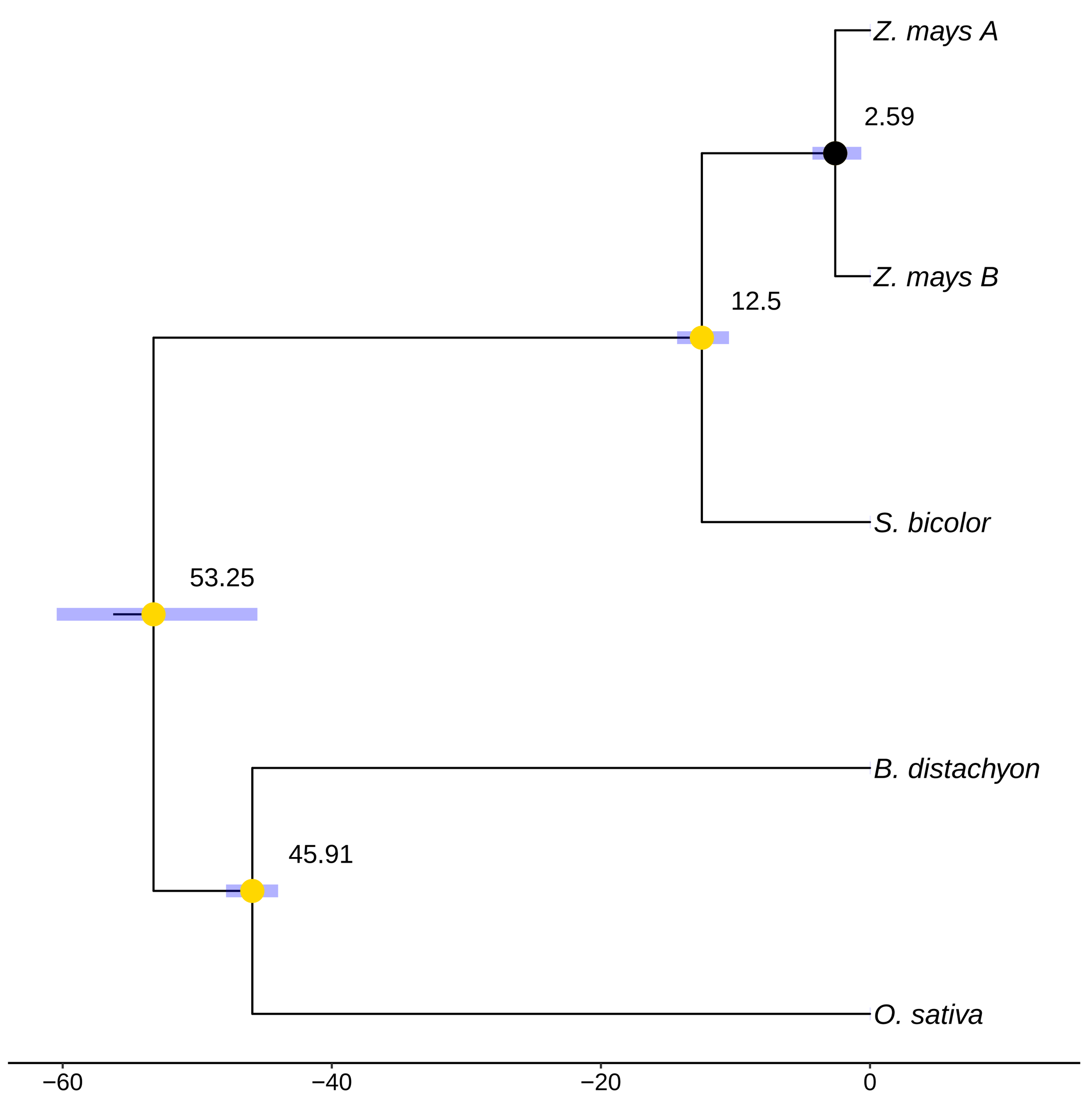

Zm00044a000293_T001

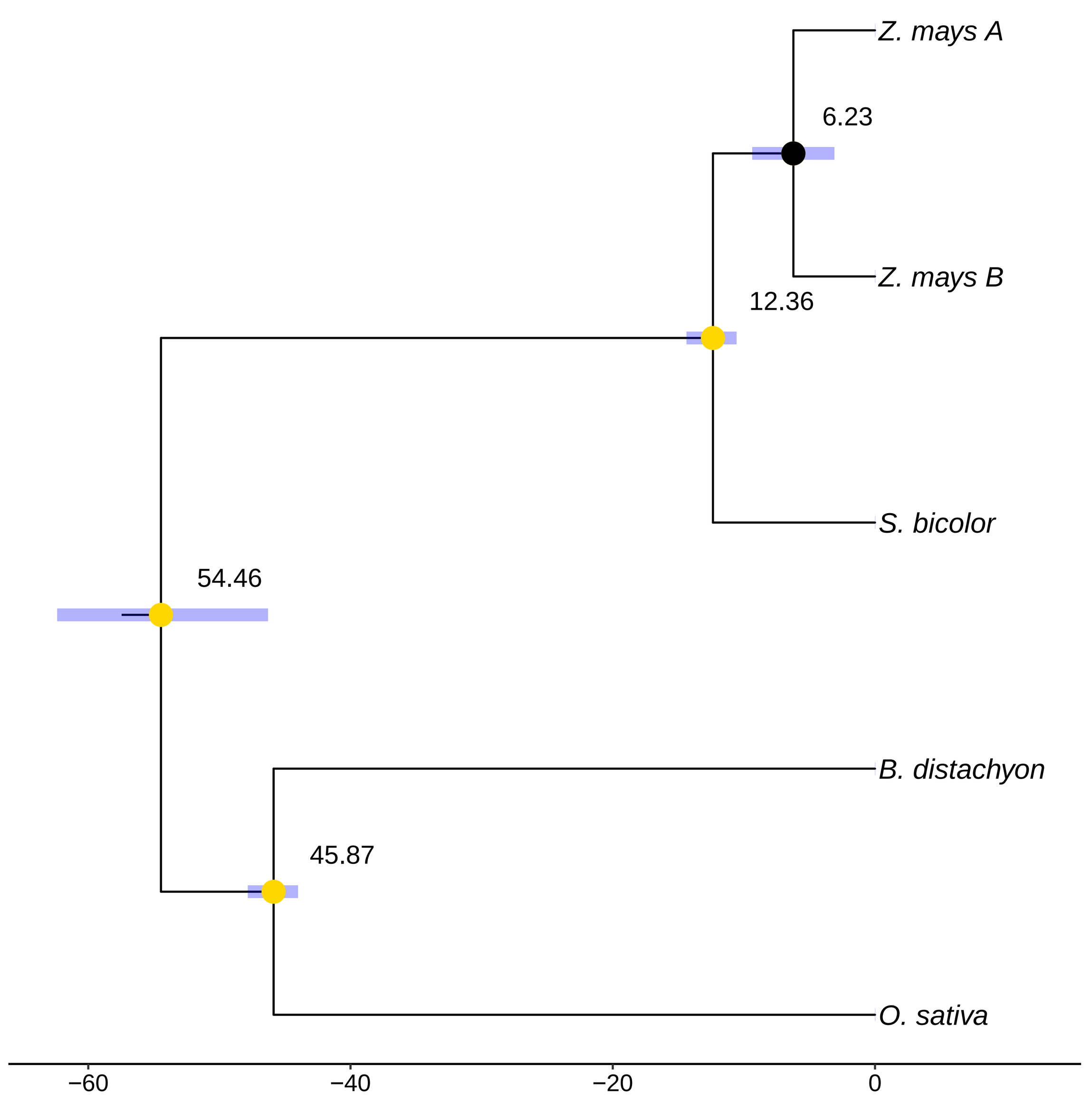

Zm00044a000297_T001

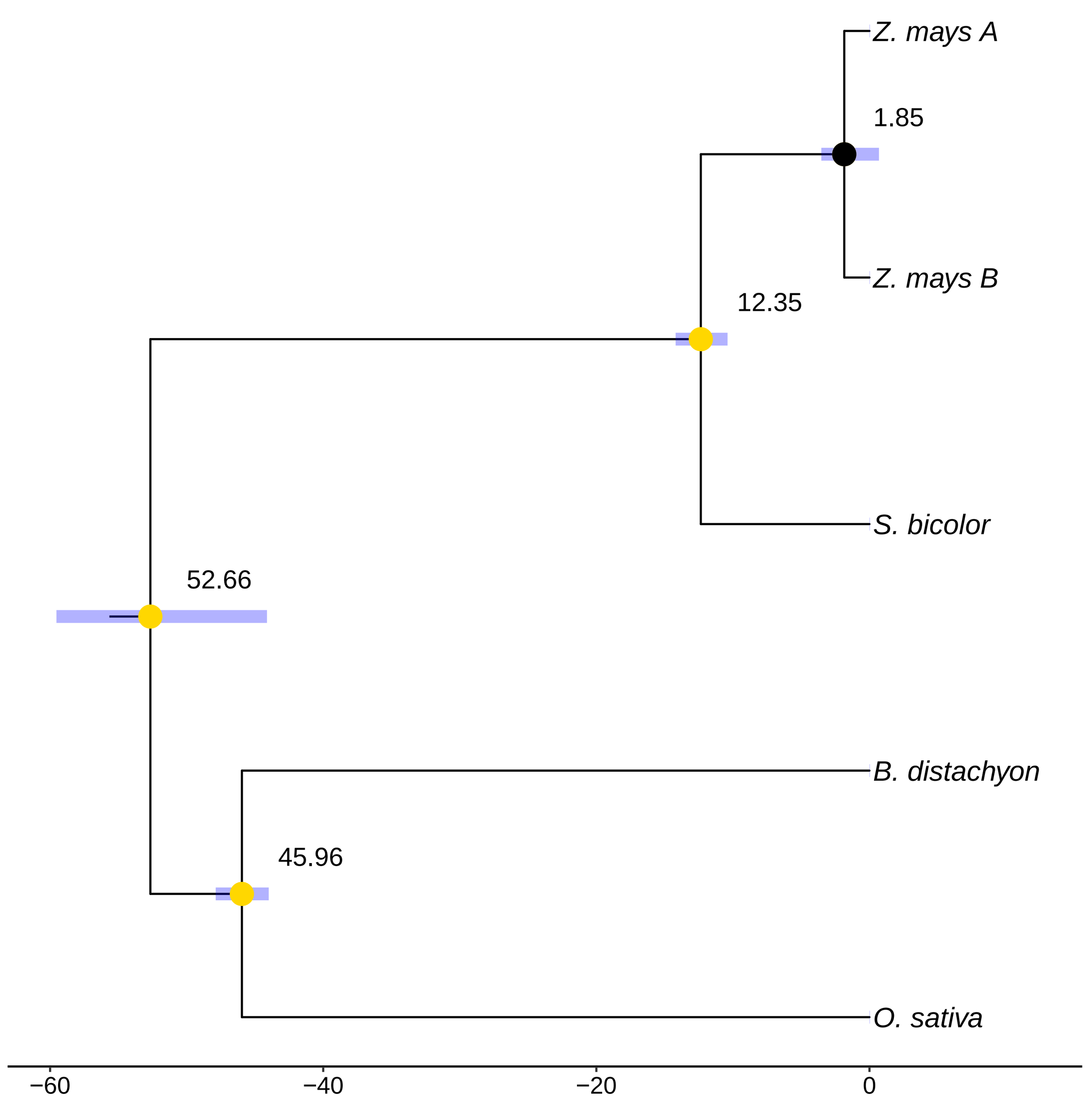

Zm00044a000306_T001

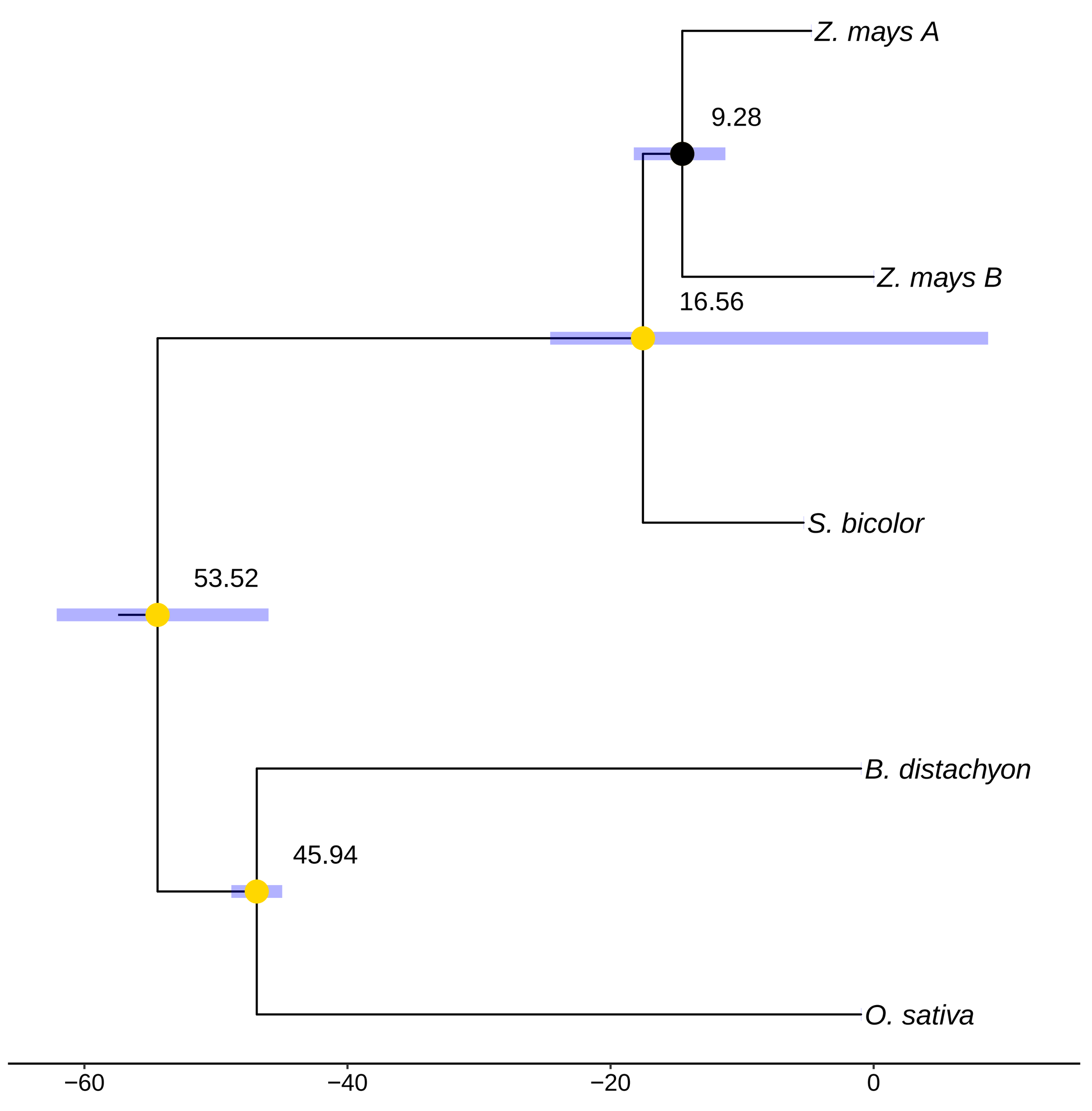

Zm00044a000307_T001

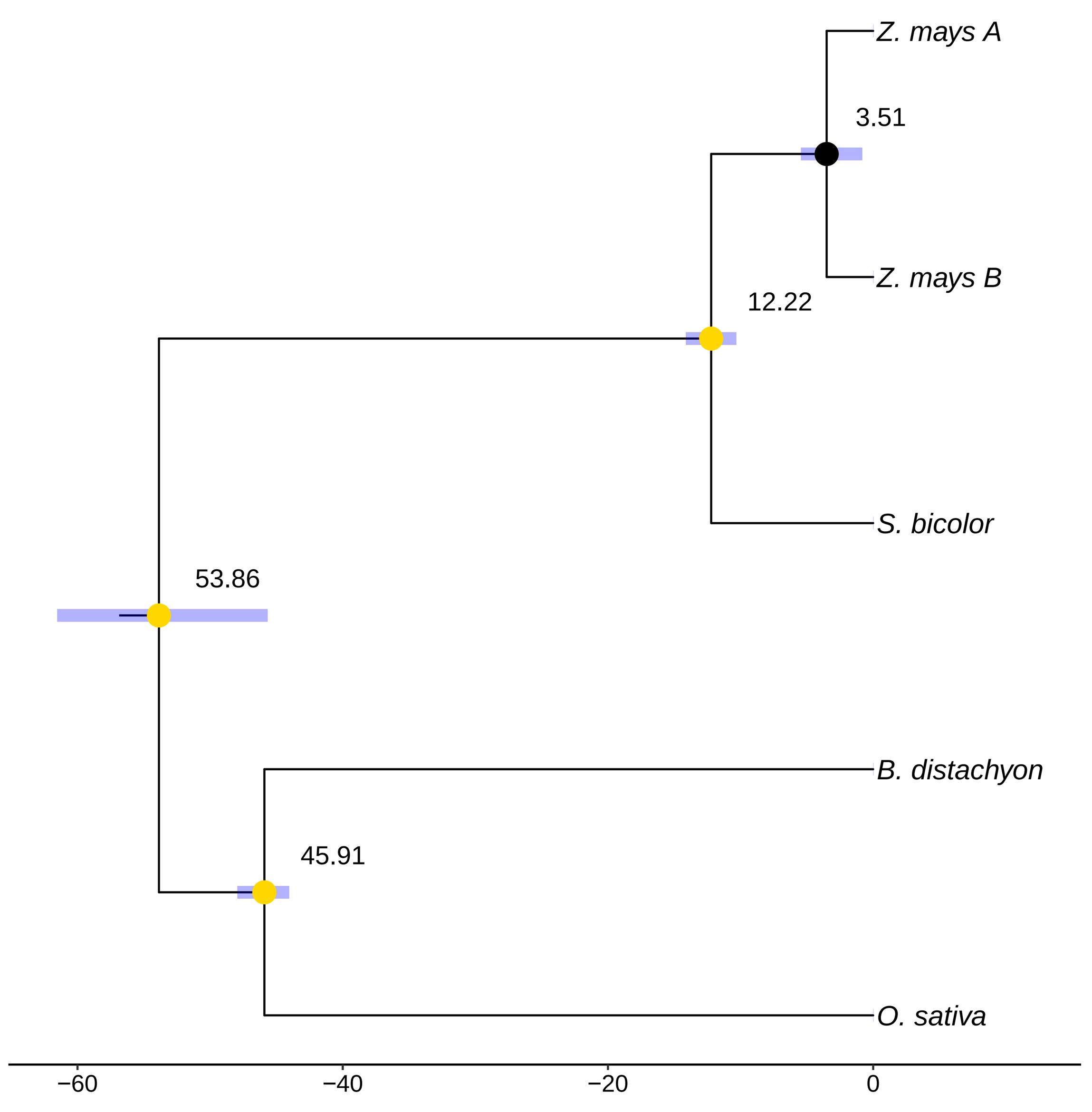

Zm00044a000312_T001

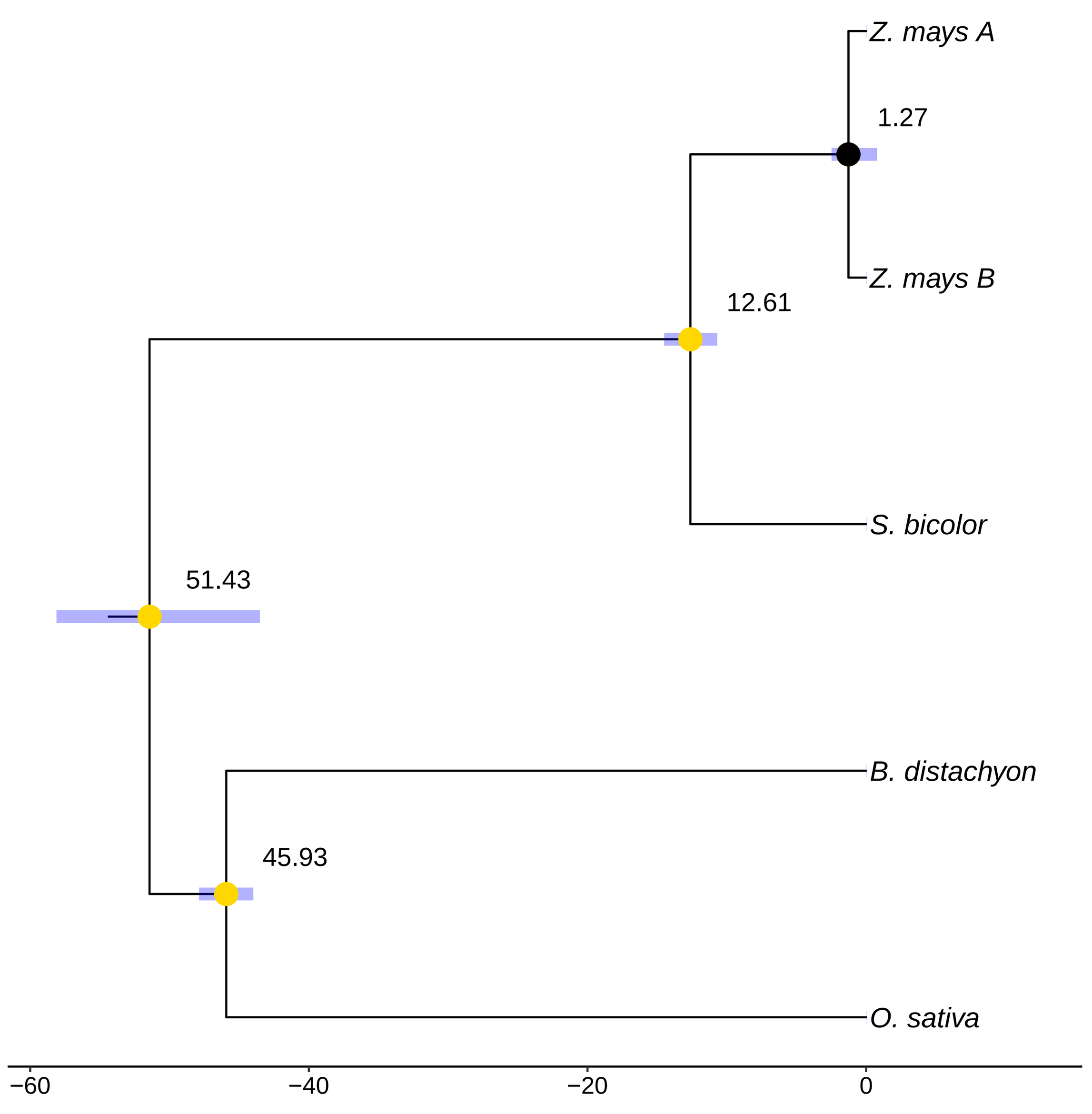

Zm00044a000320_T001

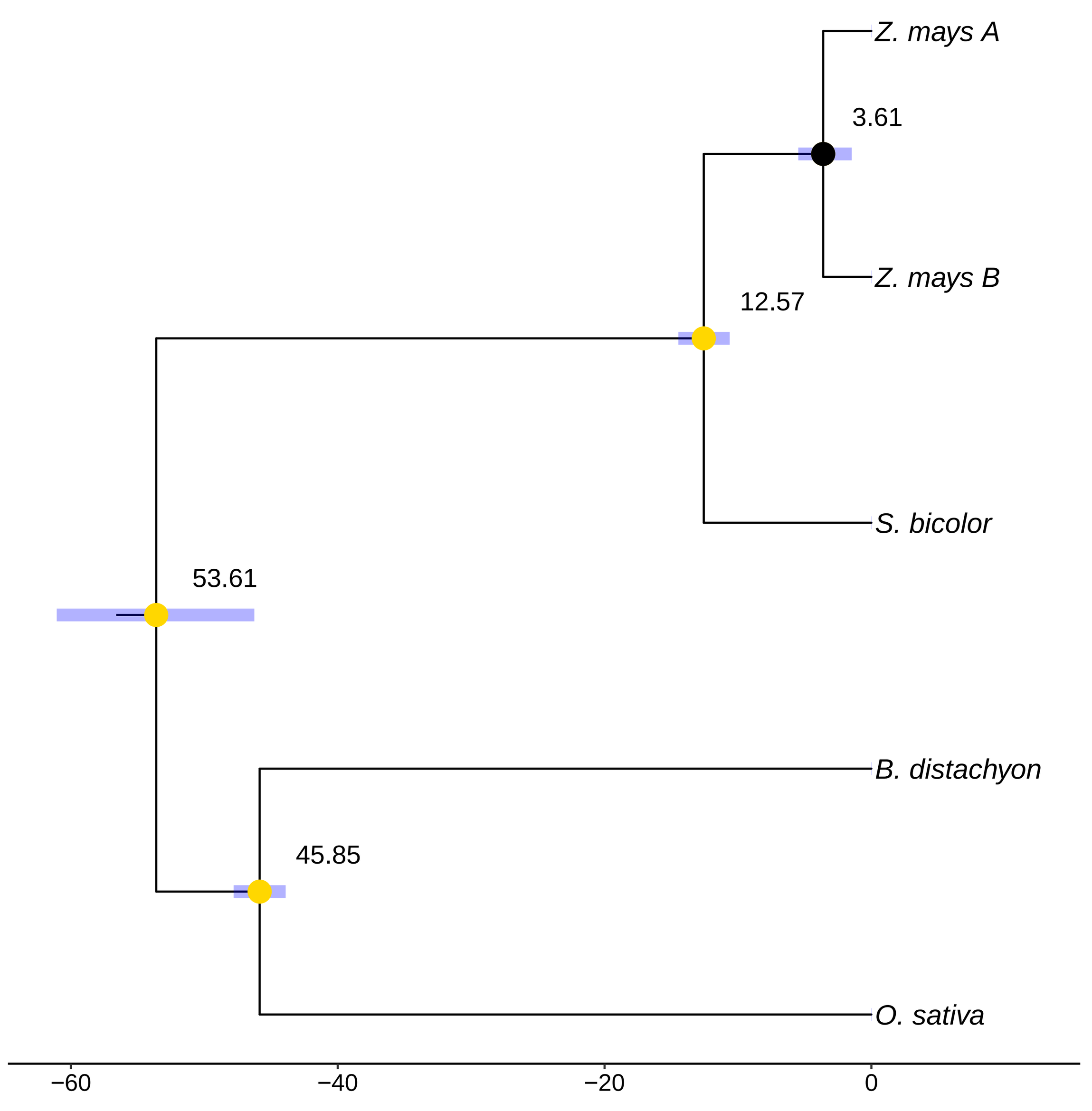

Zm00044a000325_T001

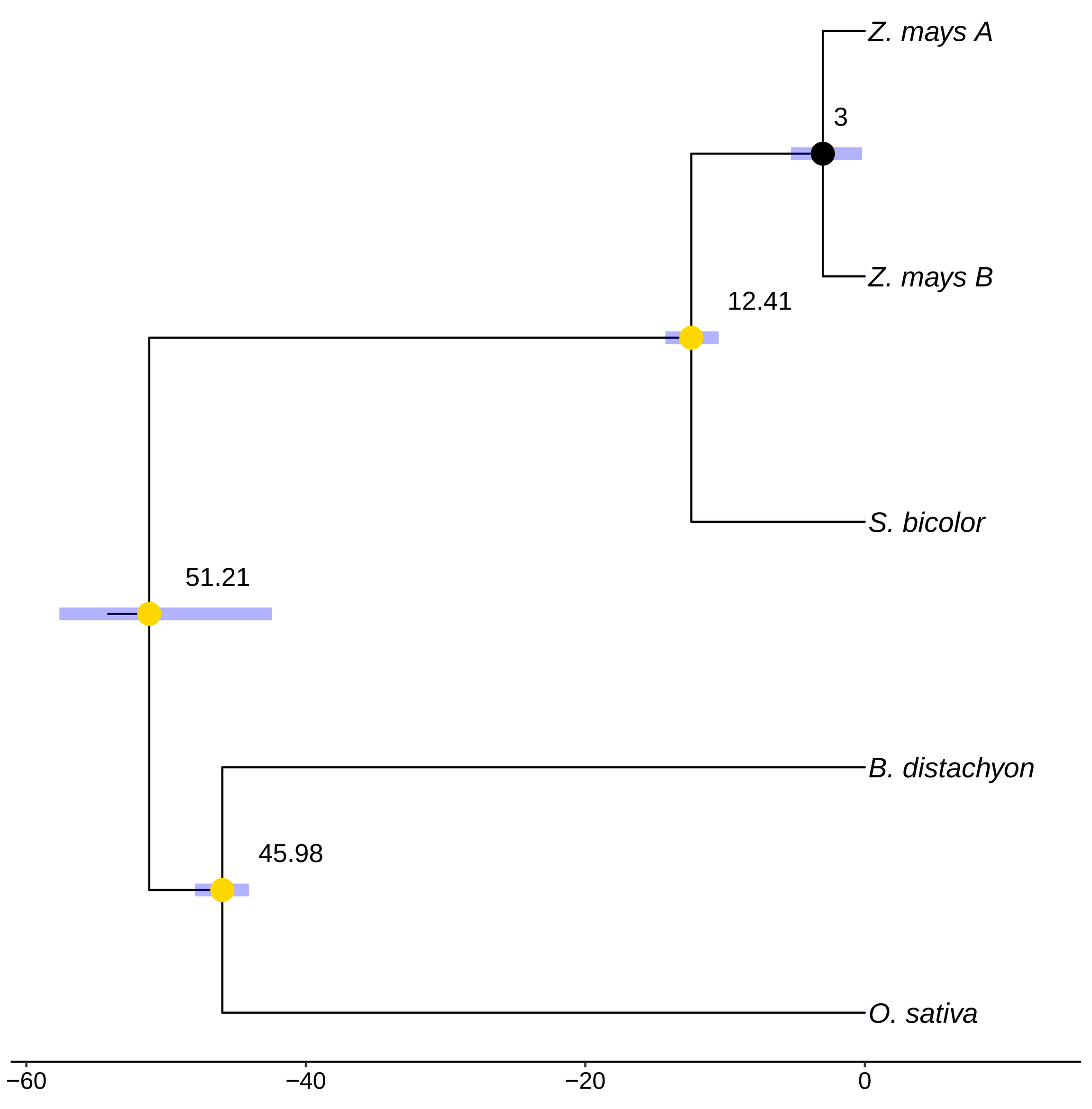

Zm00044a000331_T001

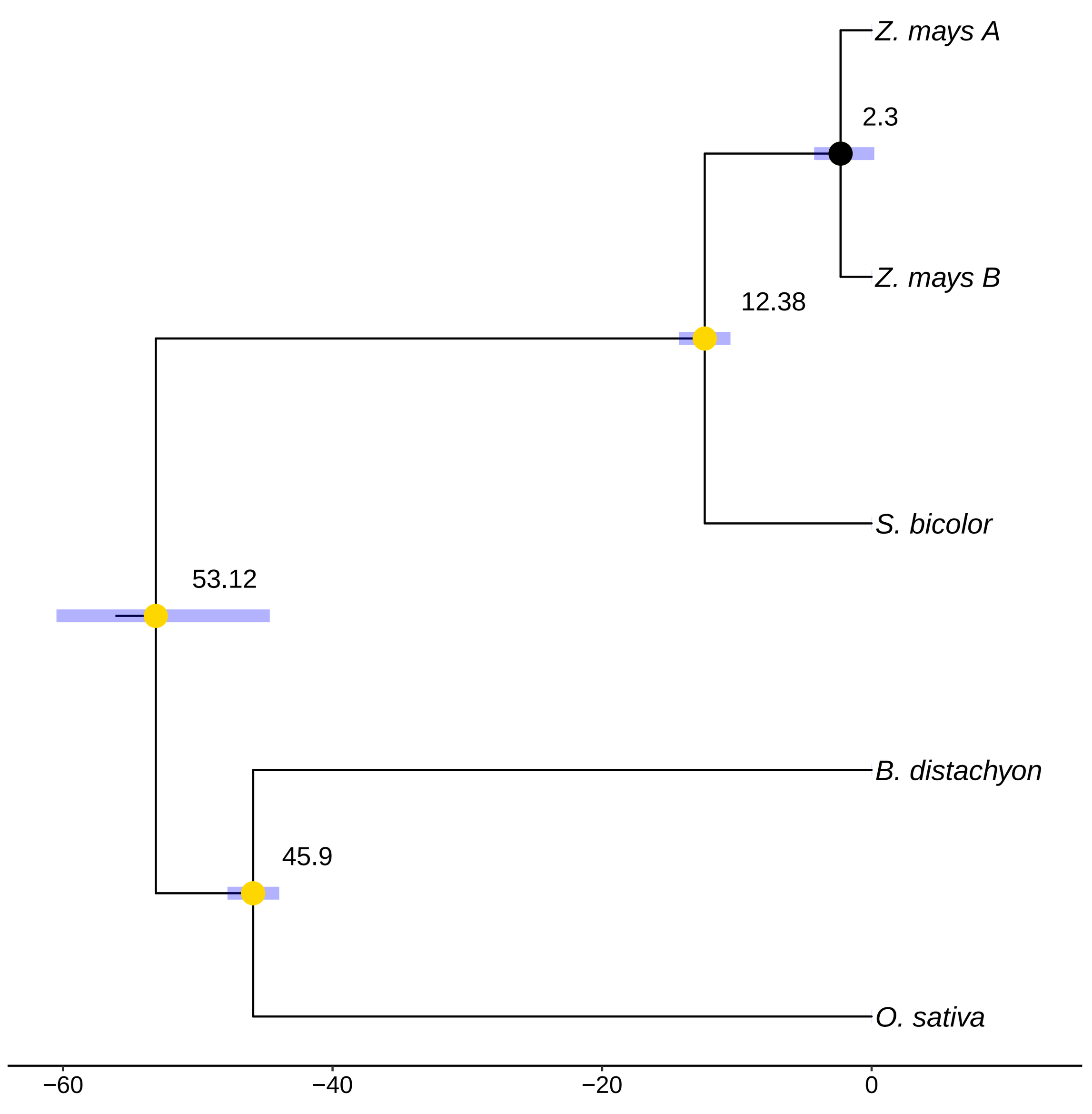

Zm00044a000334_T001

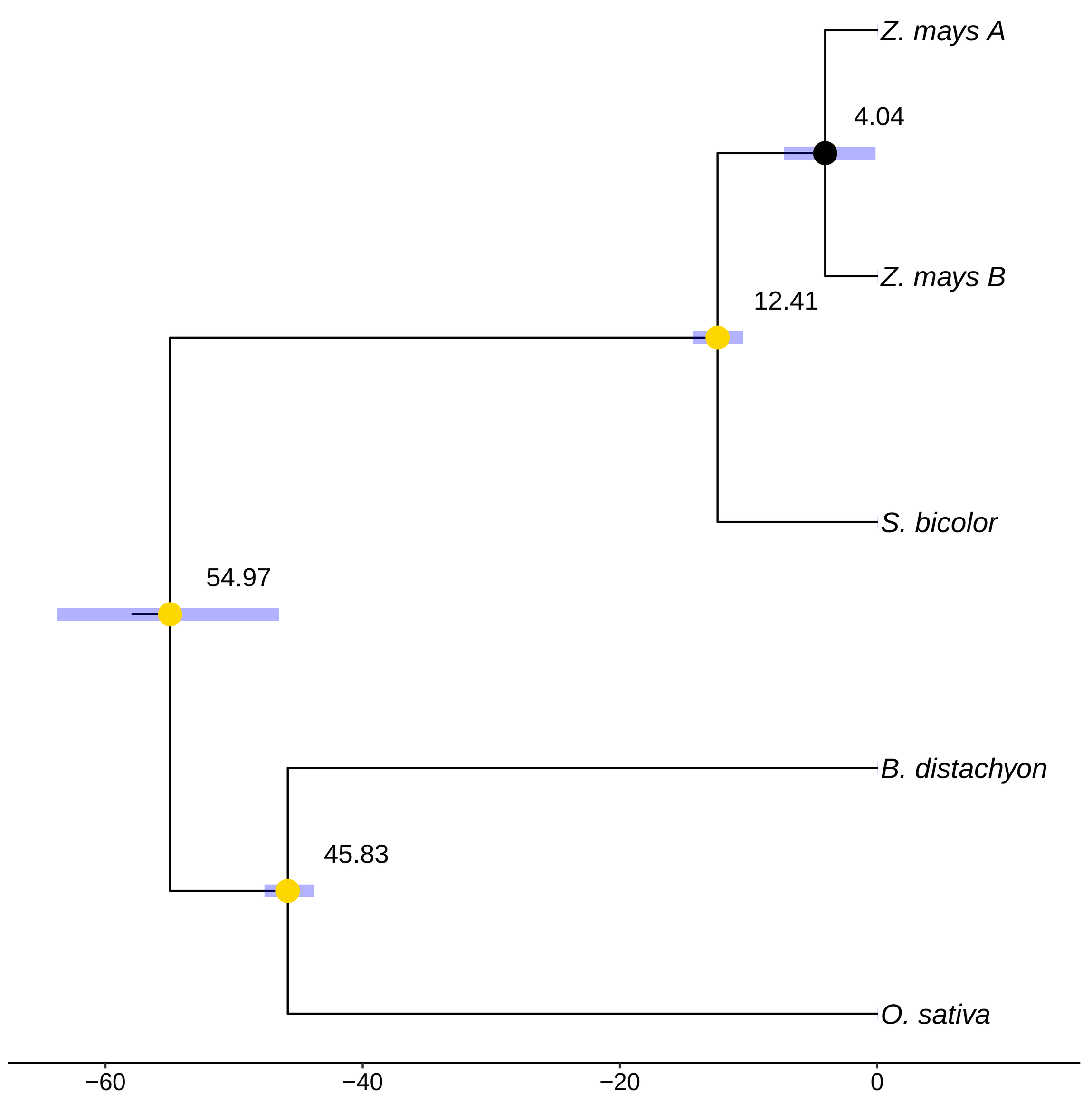

Zm00044a000338_T001

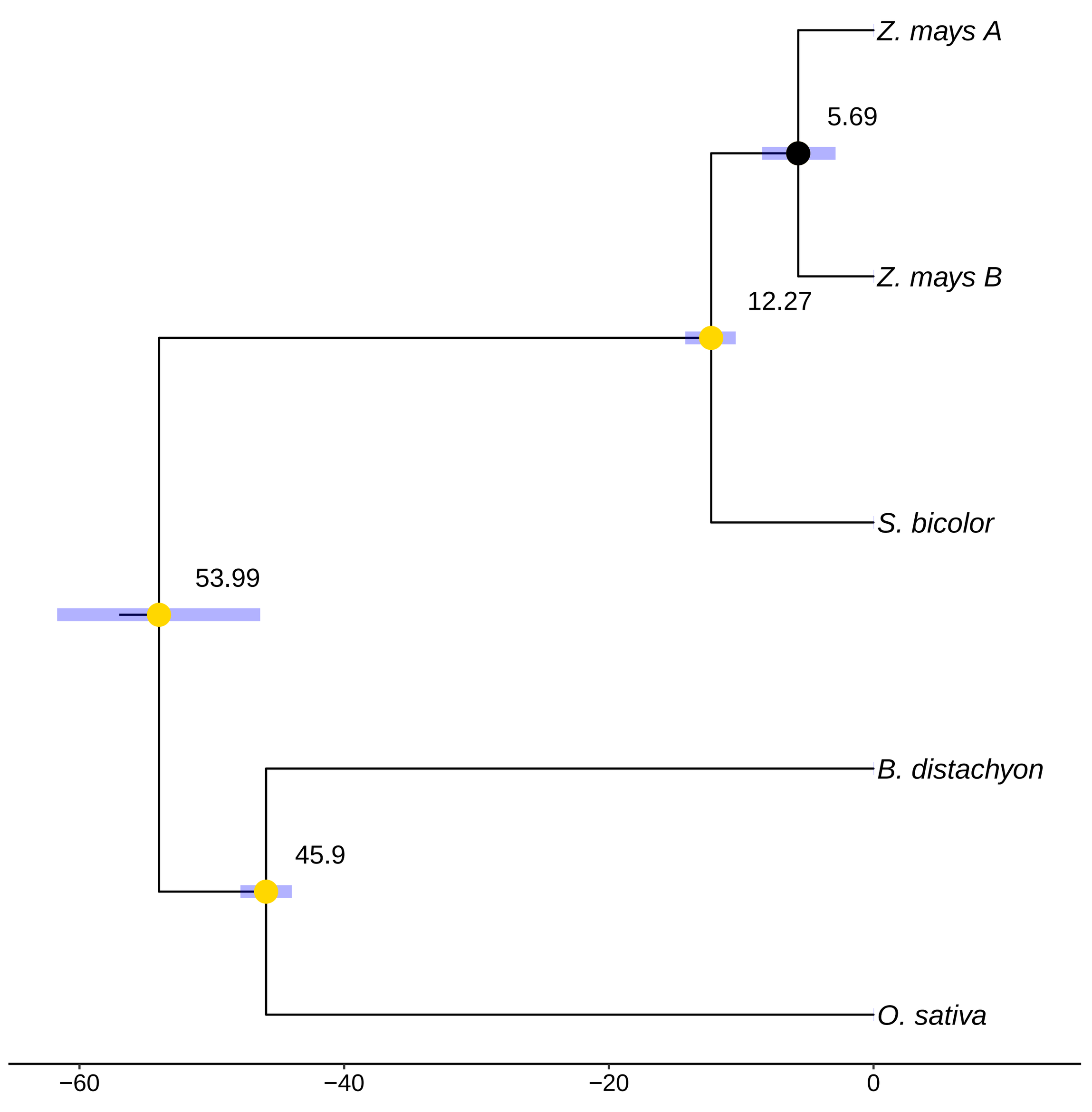

Zm00044a000341_T001

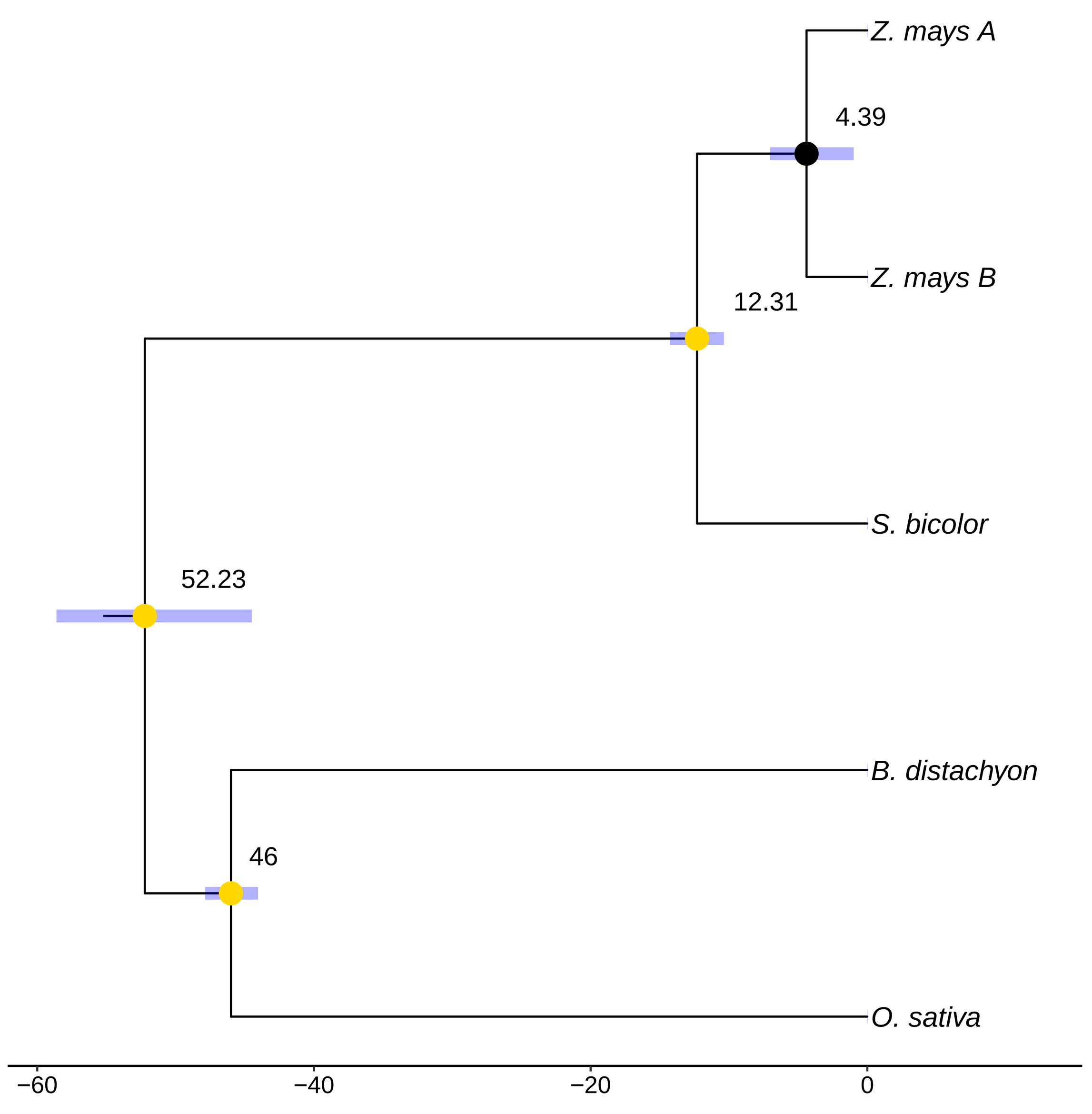

Zm00044a000342_T001

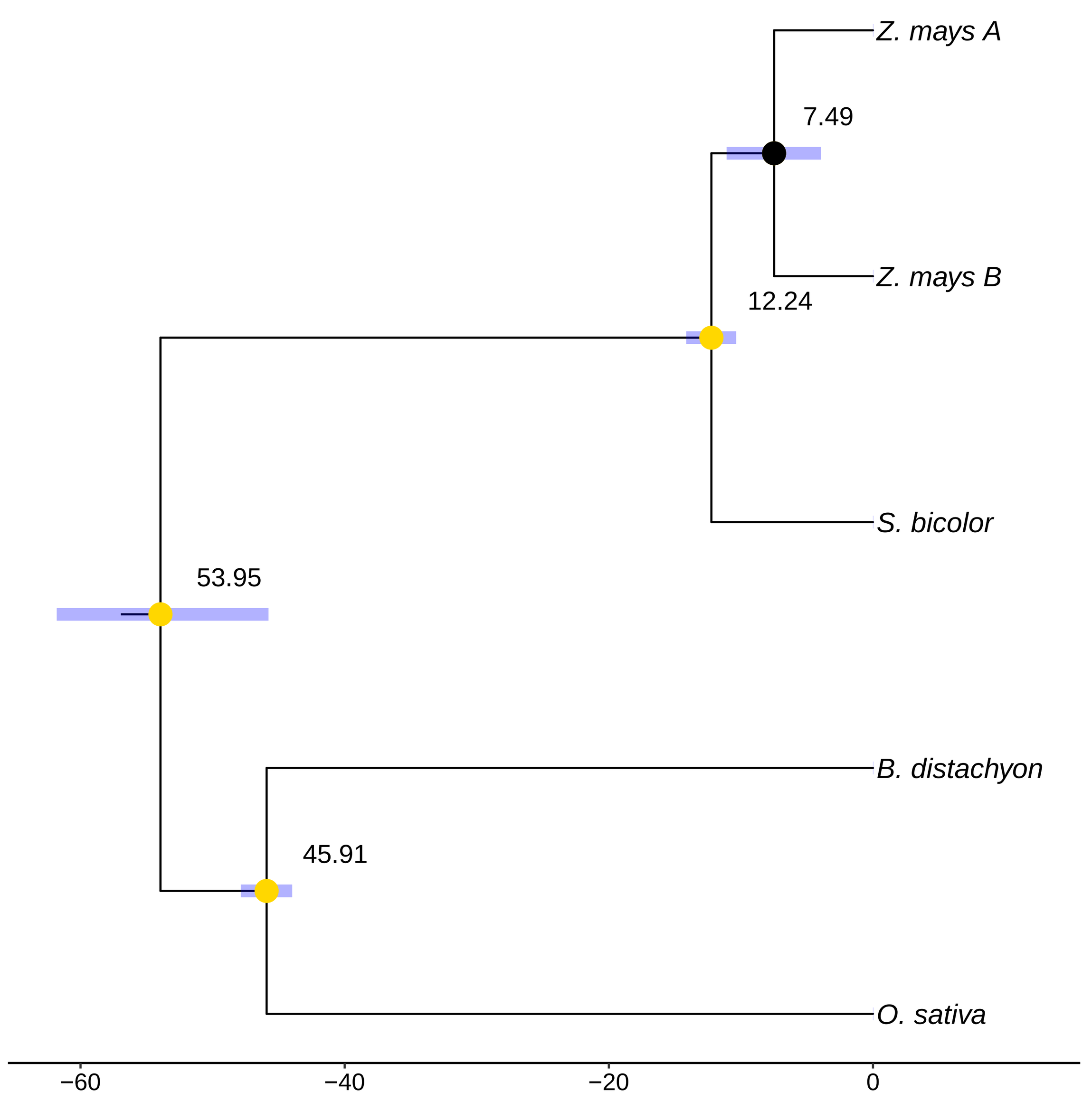

Zm00044a000343_T001

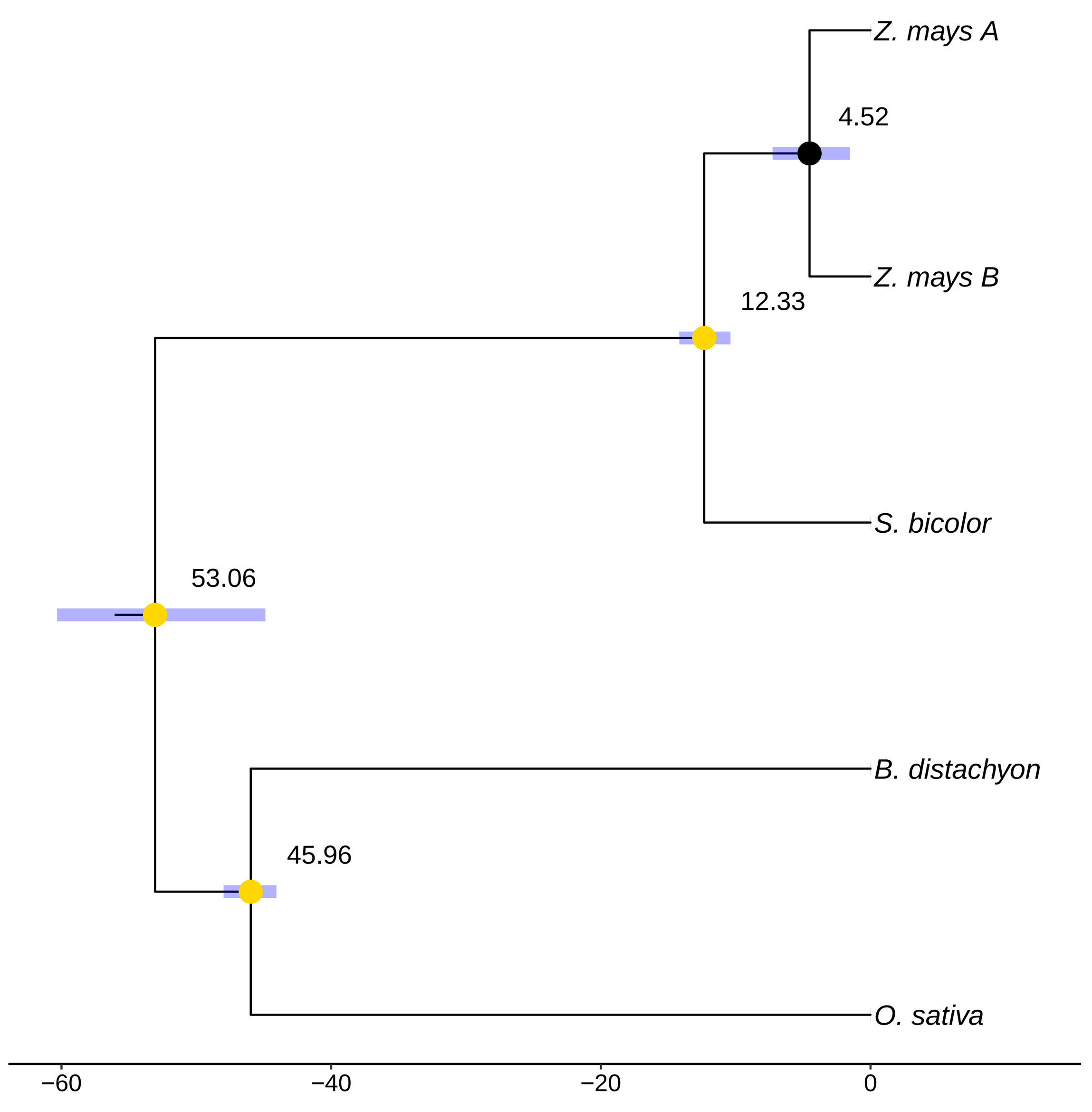

Zm00044a000347_T001

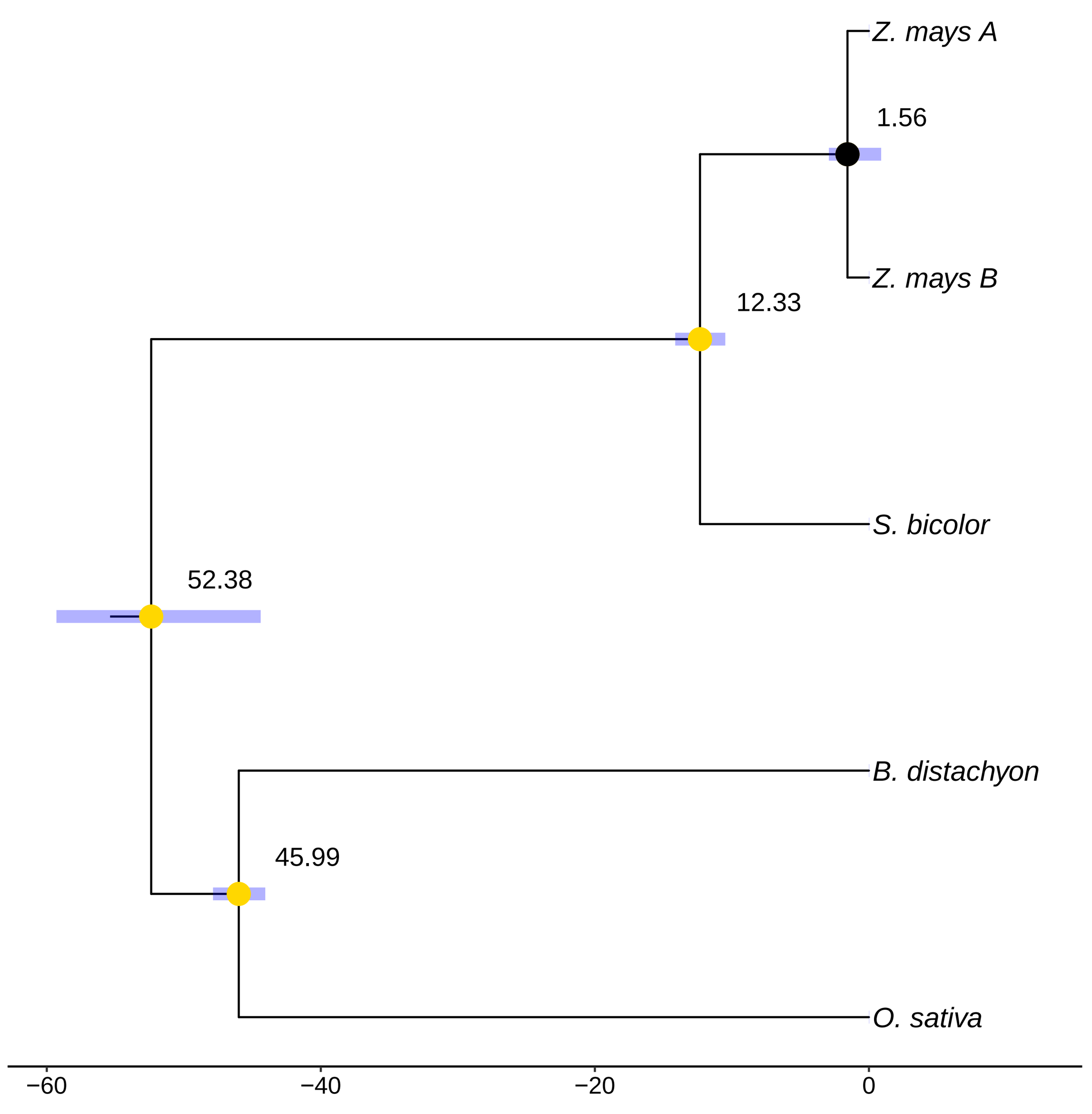

Zm00044a000351_T001

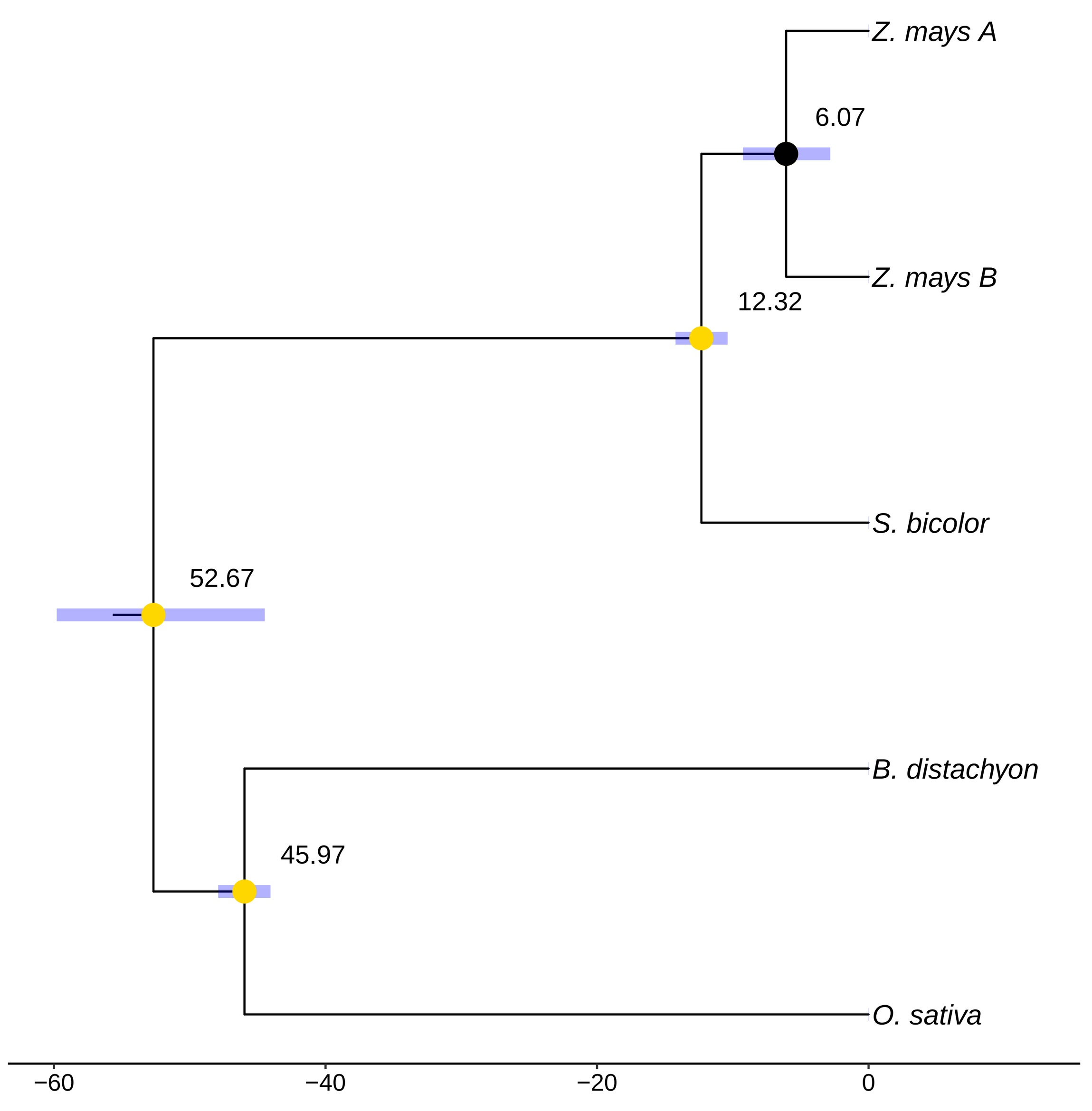

Zm00044a000355_T001

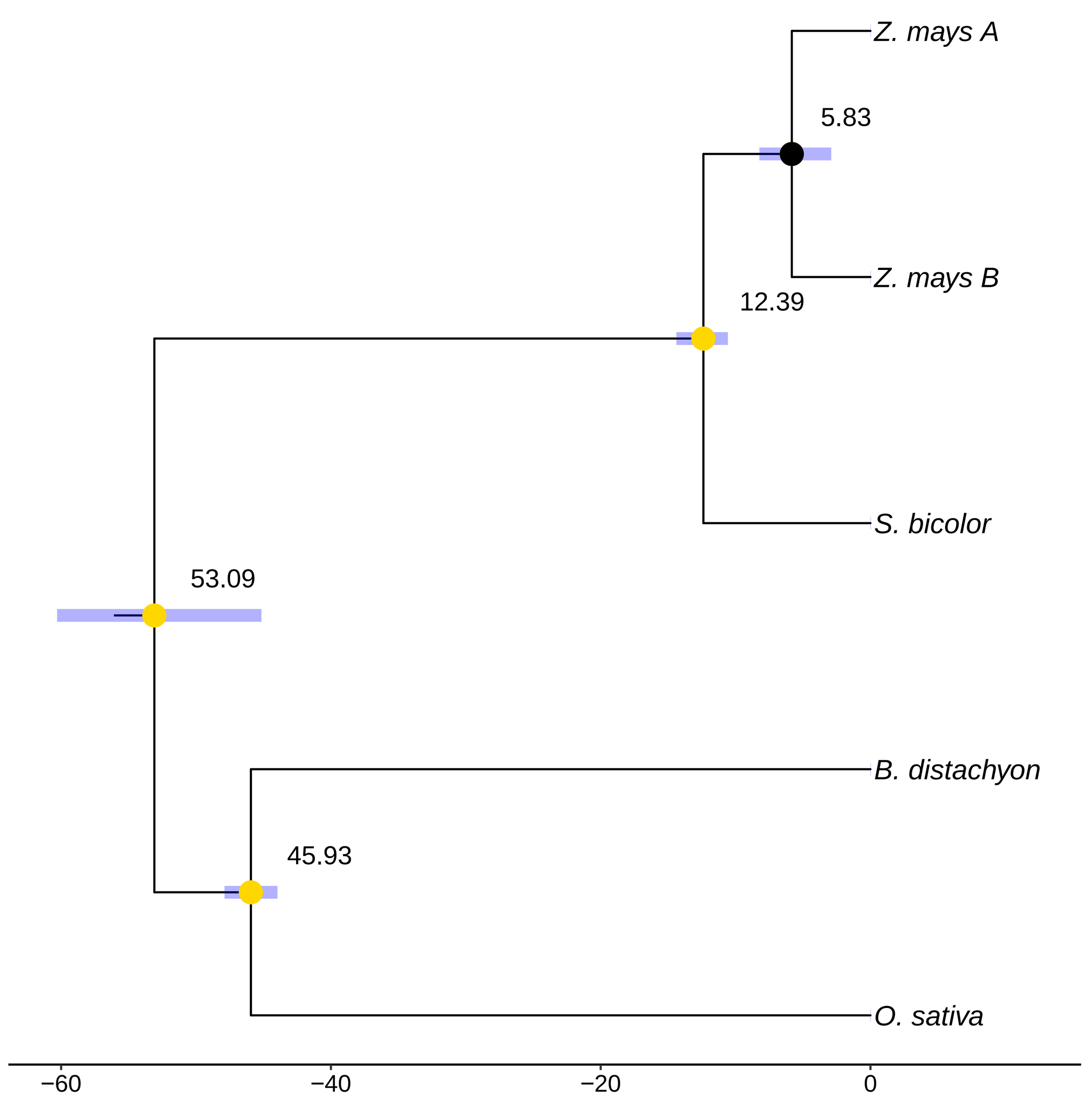

Zm00044a000362_T001

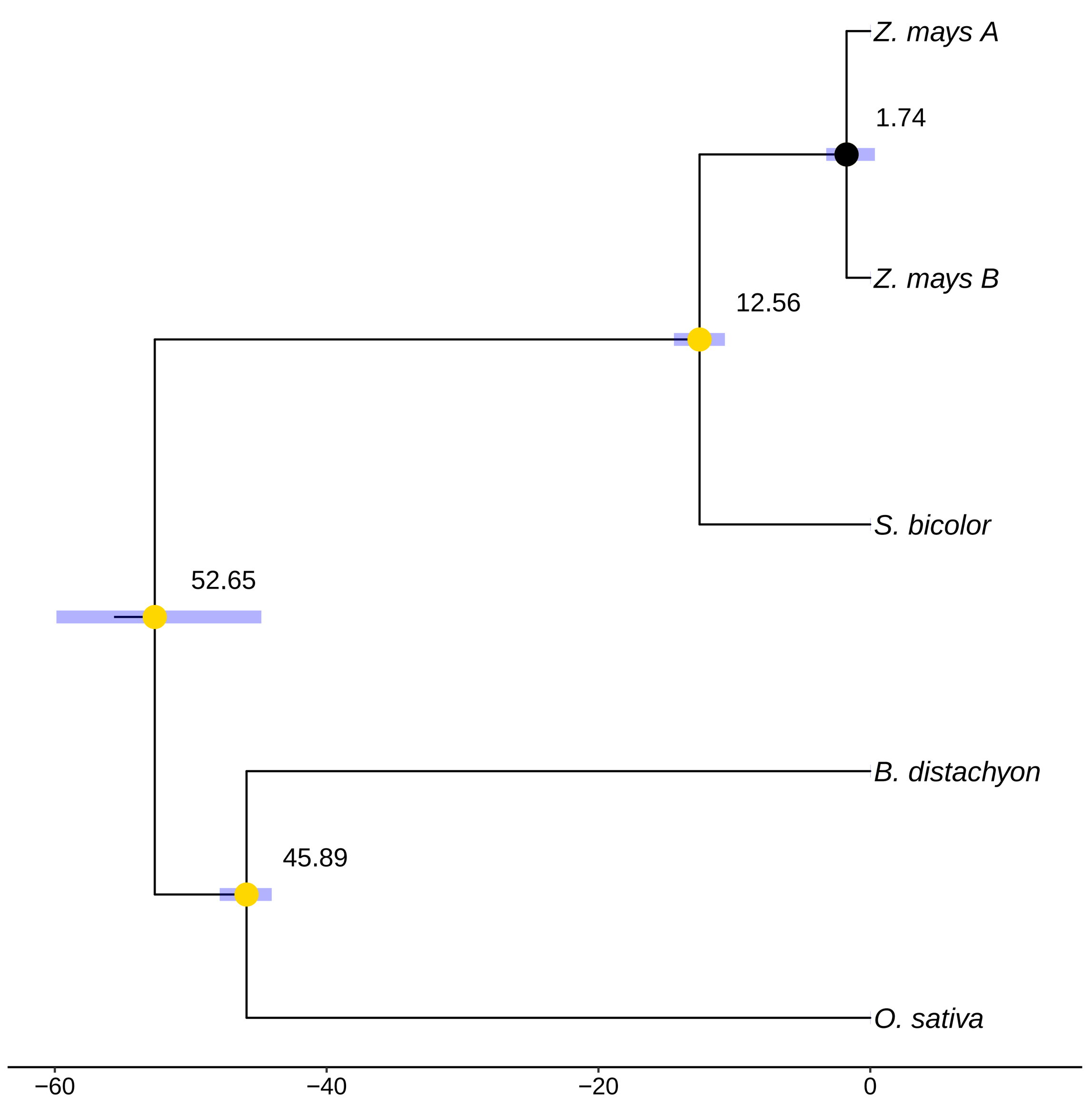

Zm00044a000363_T001

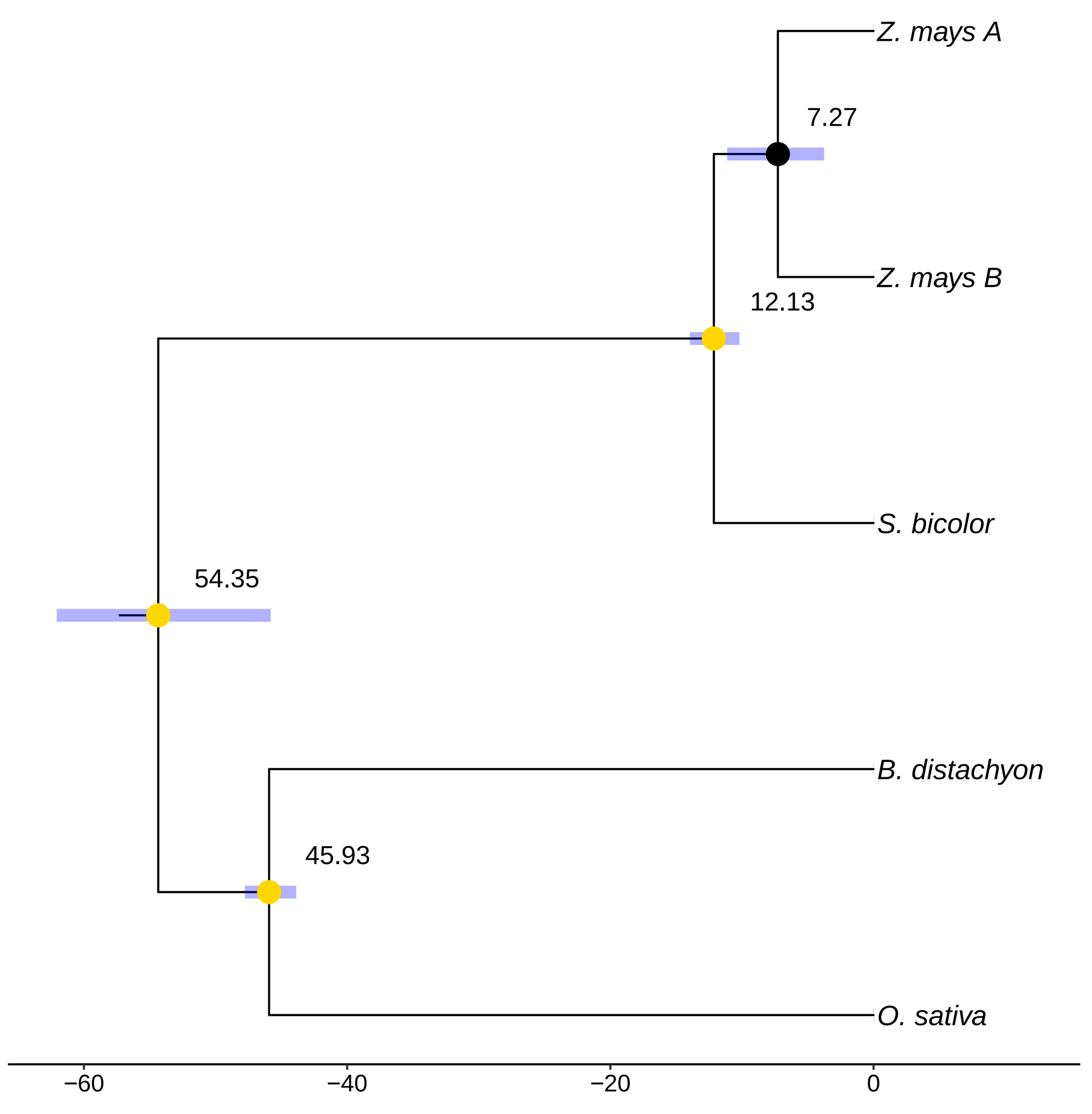

Zm00044a000365_T001

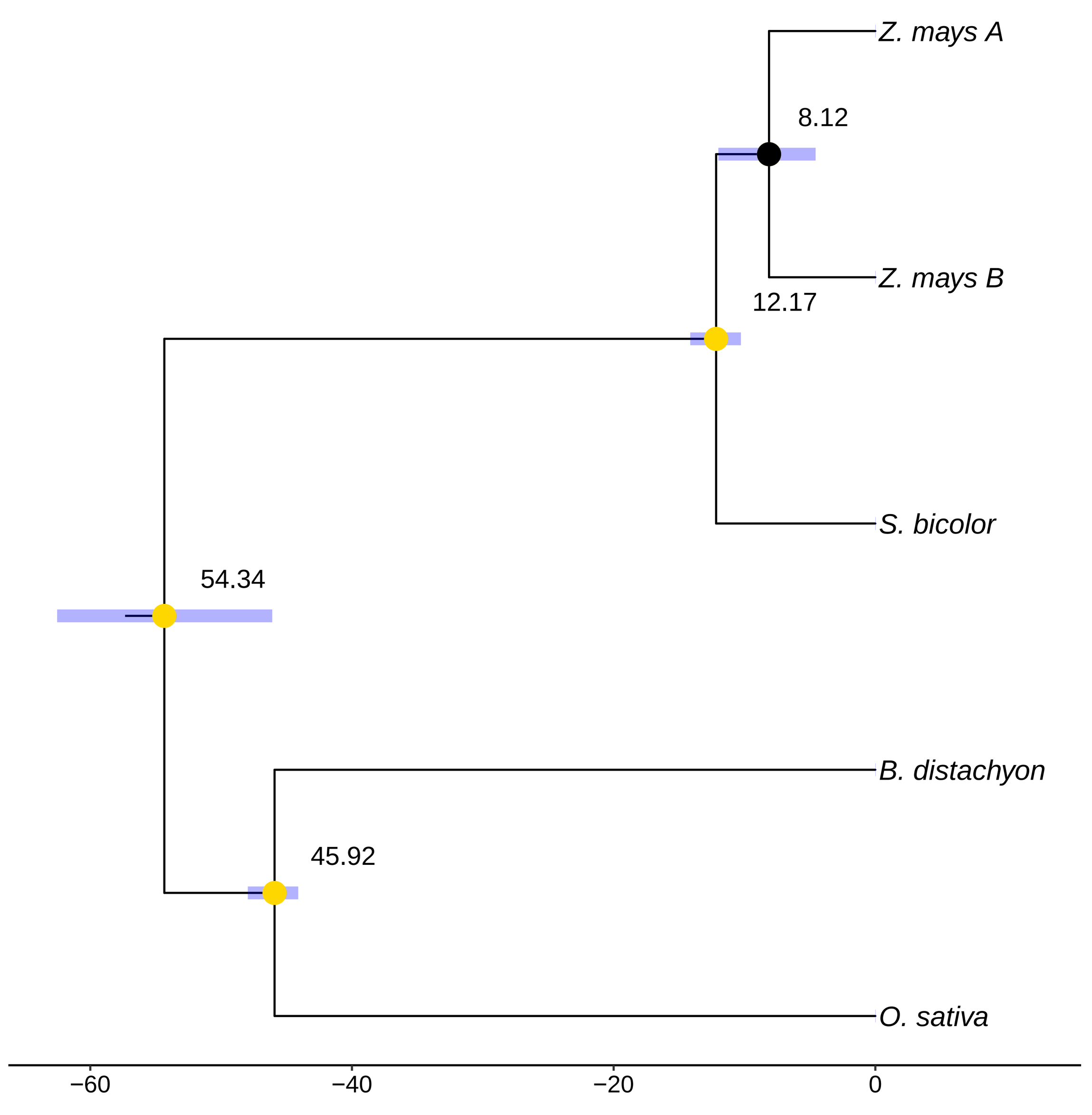

Zm00044a000368_T001

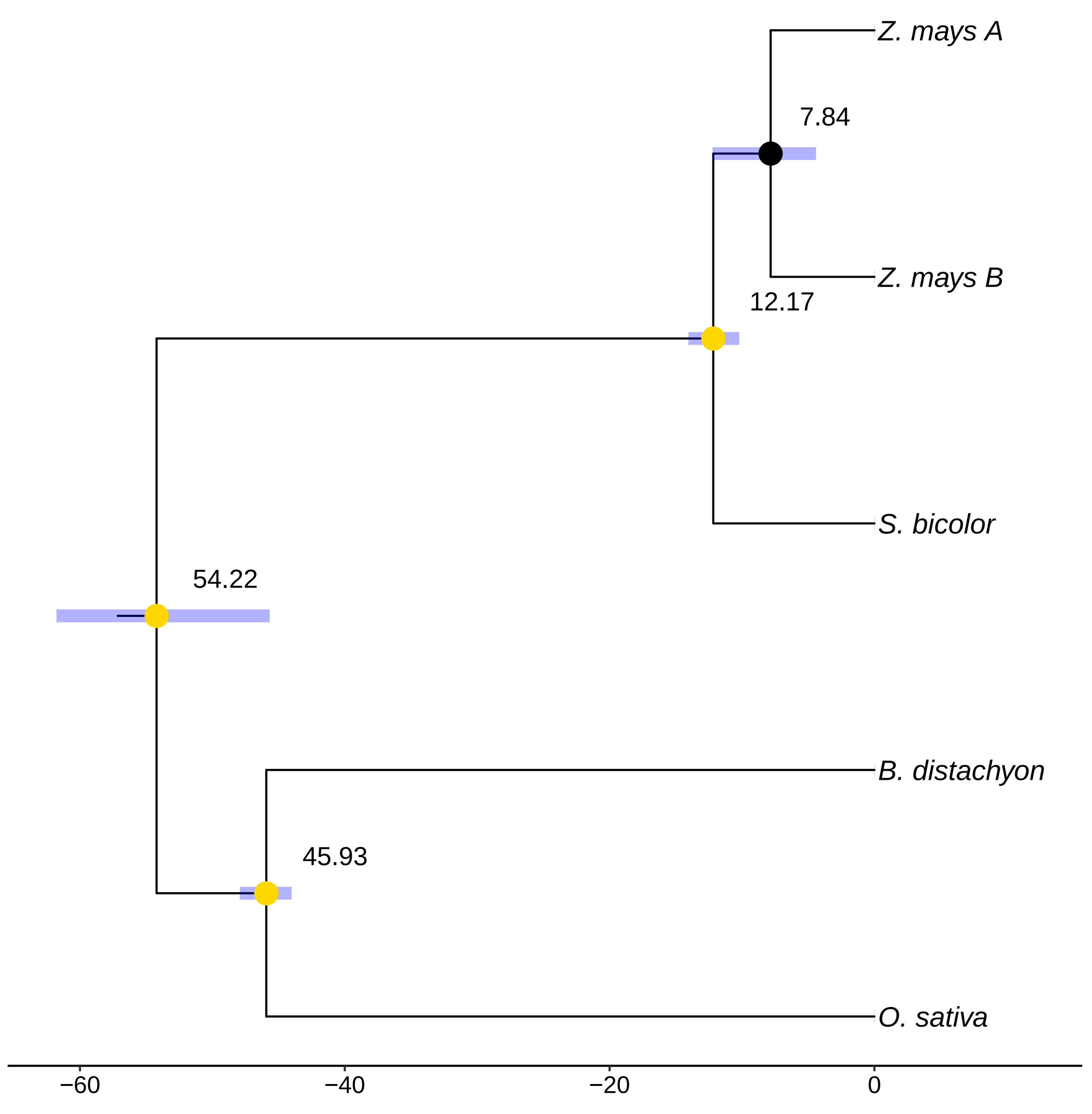

Zm00044a000369_T001

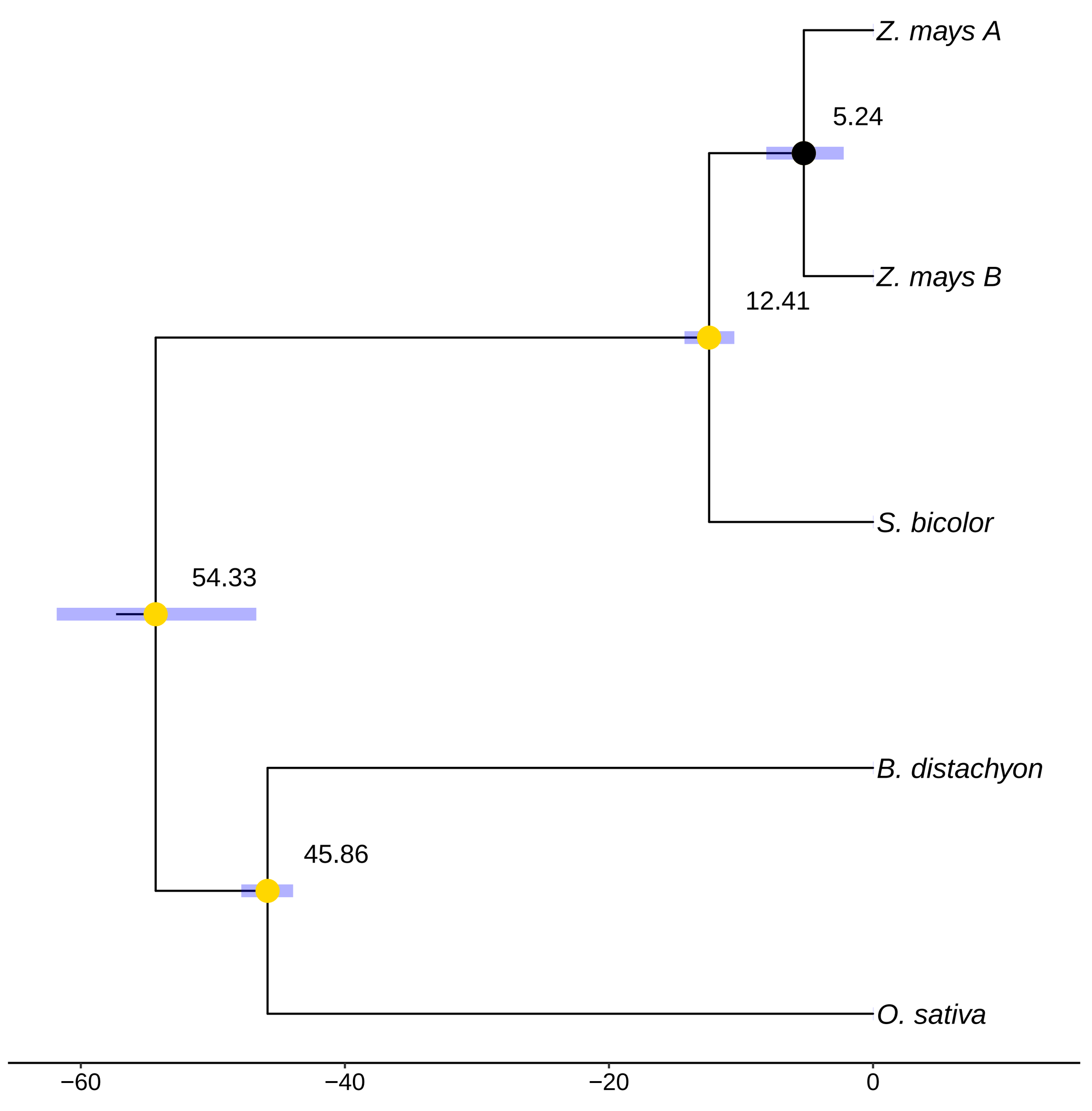

Zm00044a000376_T001

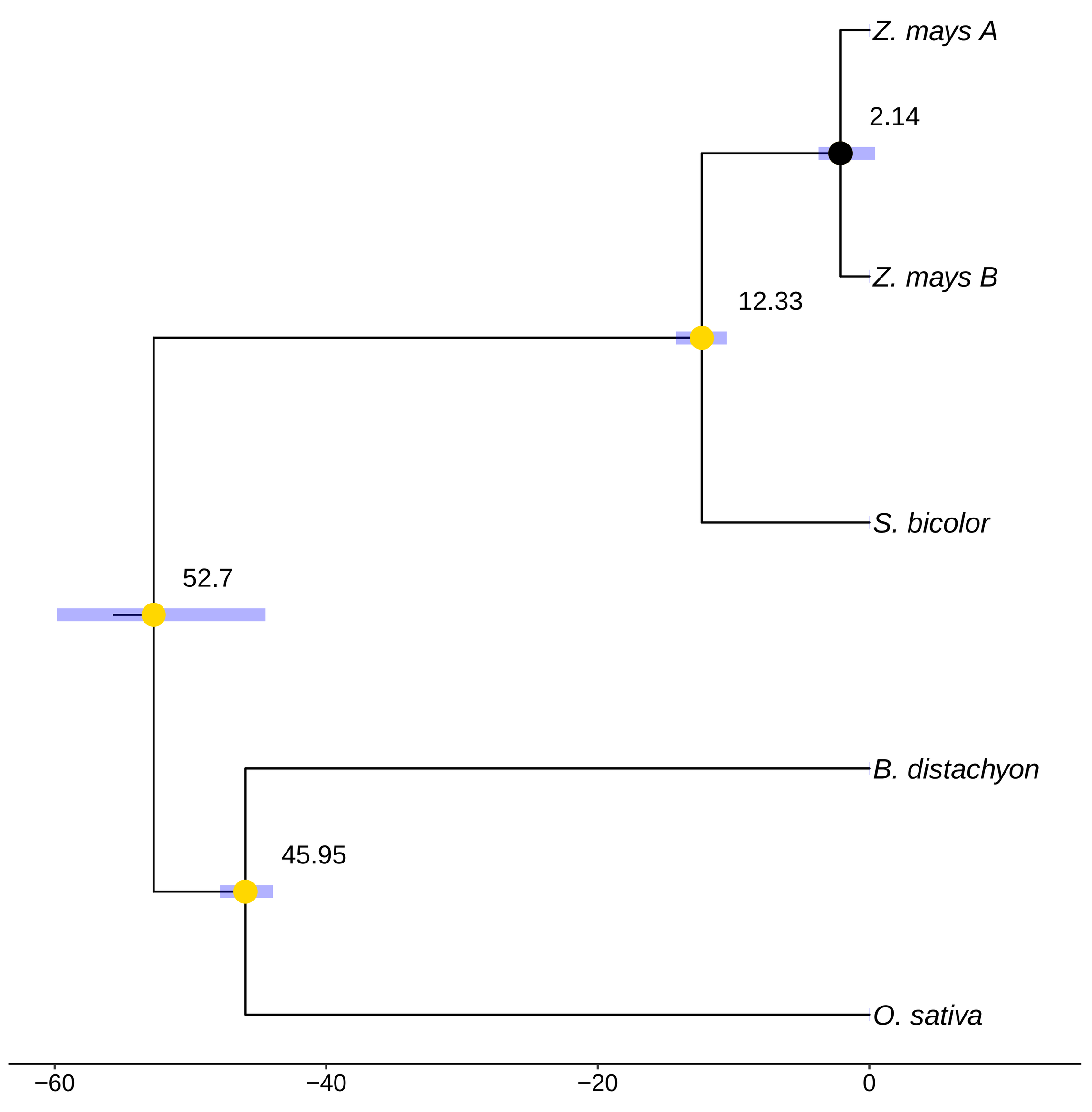

Zm00044a000377_T001

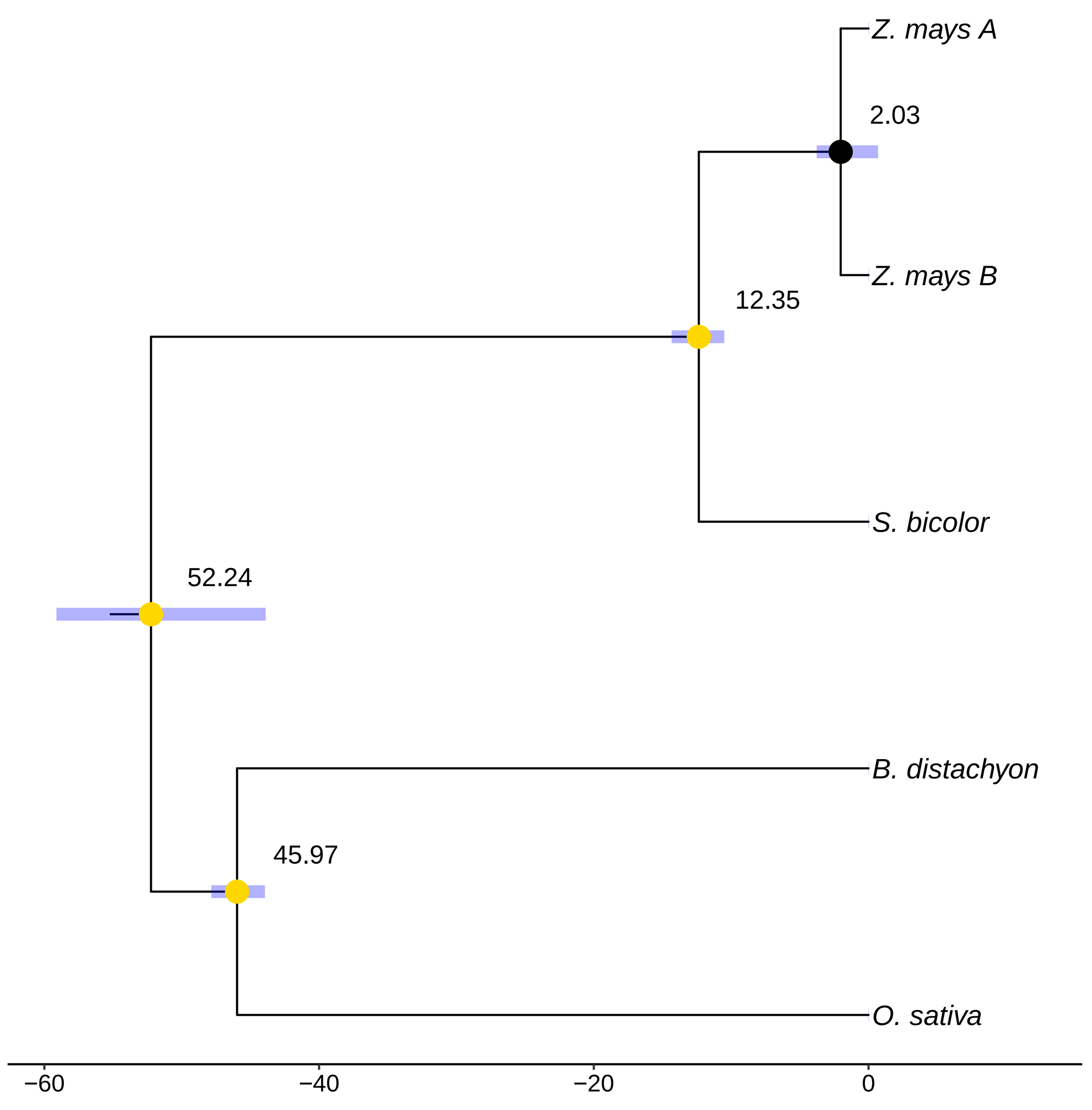

Zm00044a000383_T001

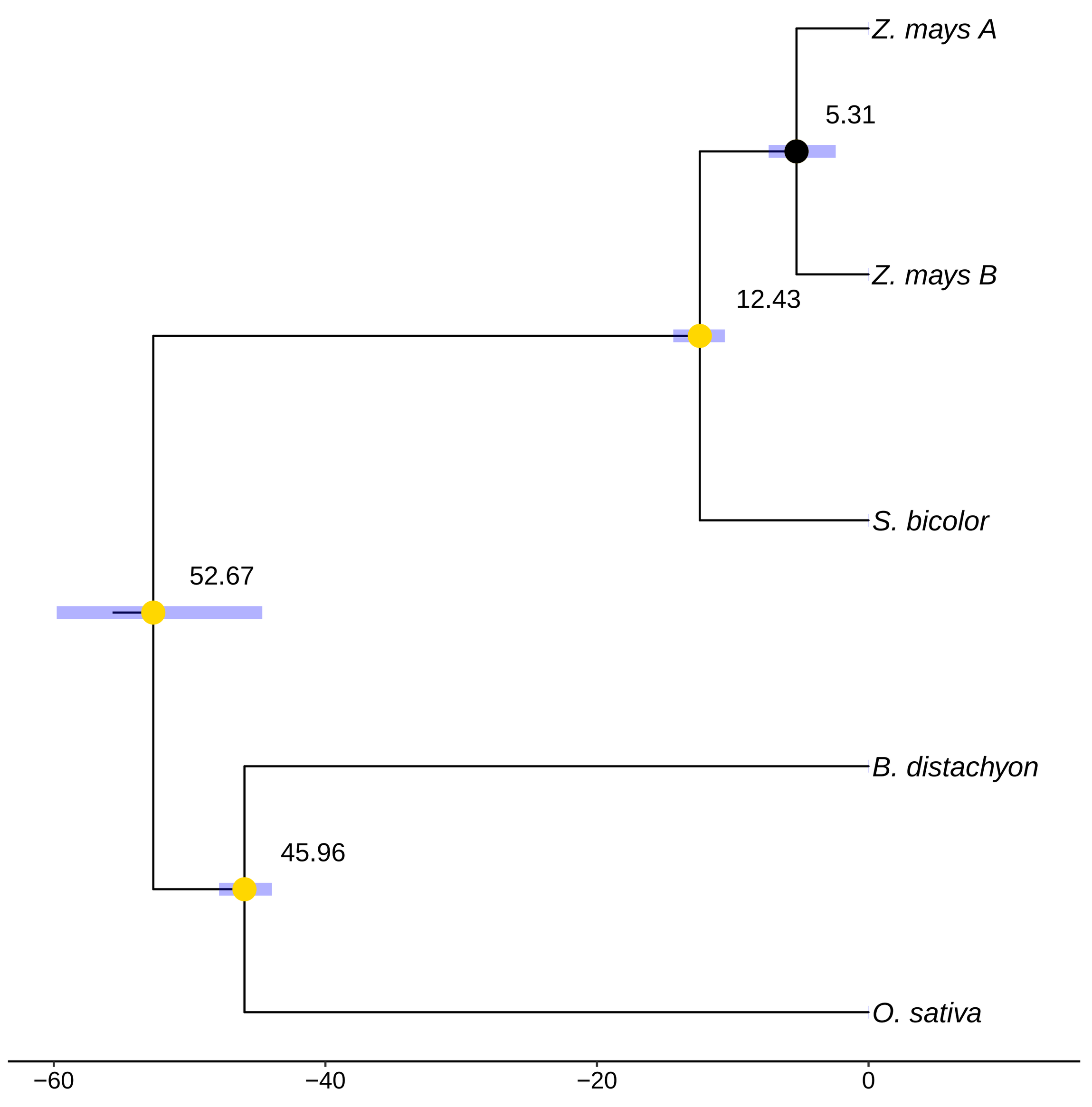

Zm00044a000384_T001

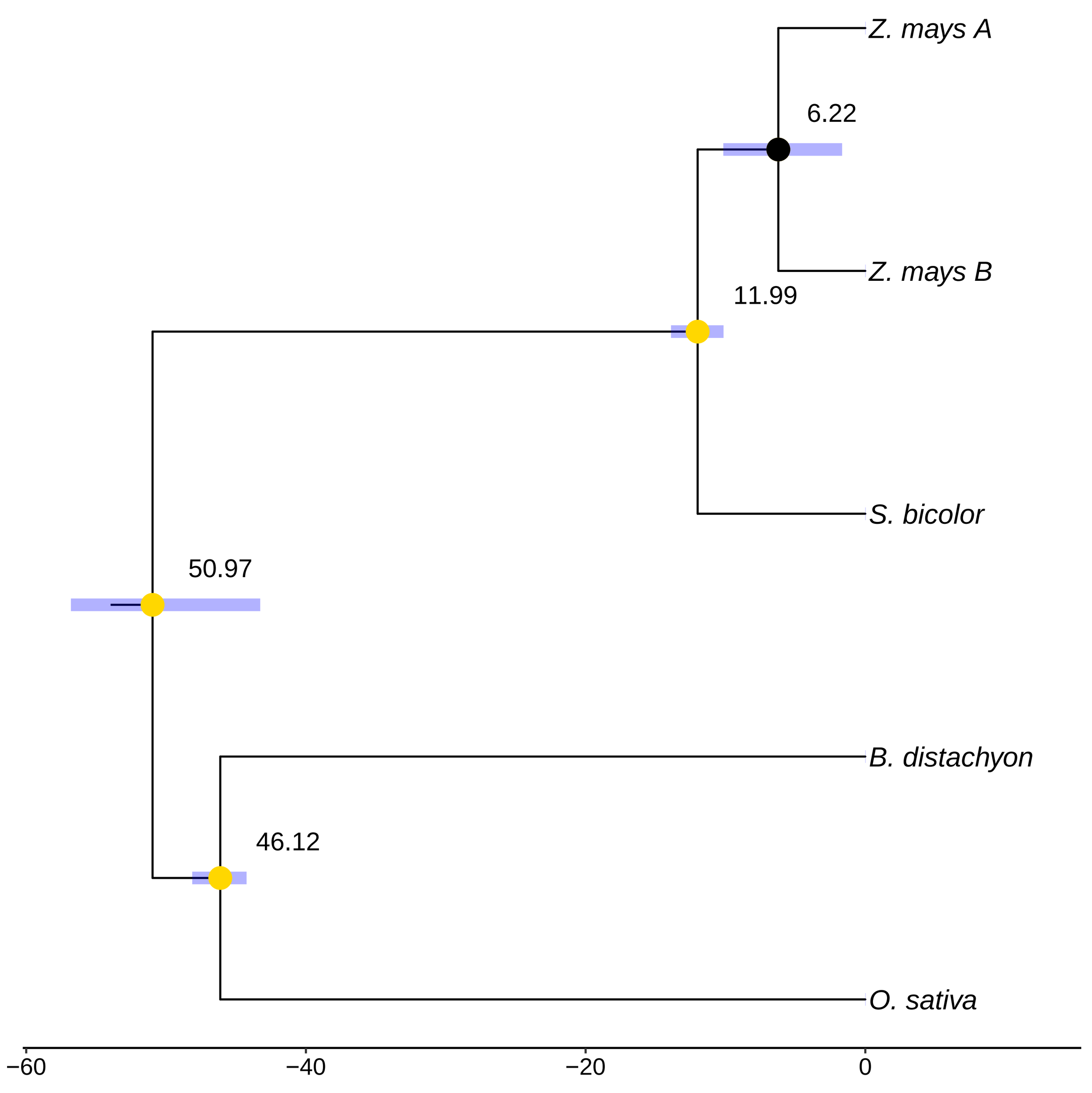

Zm00044a000385_T001

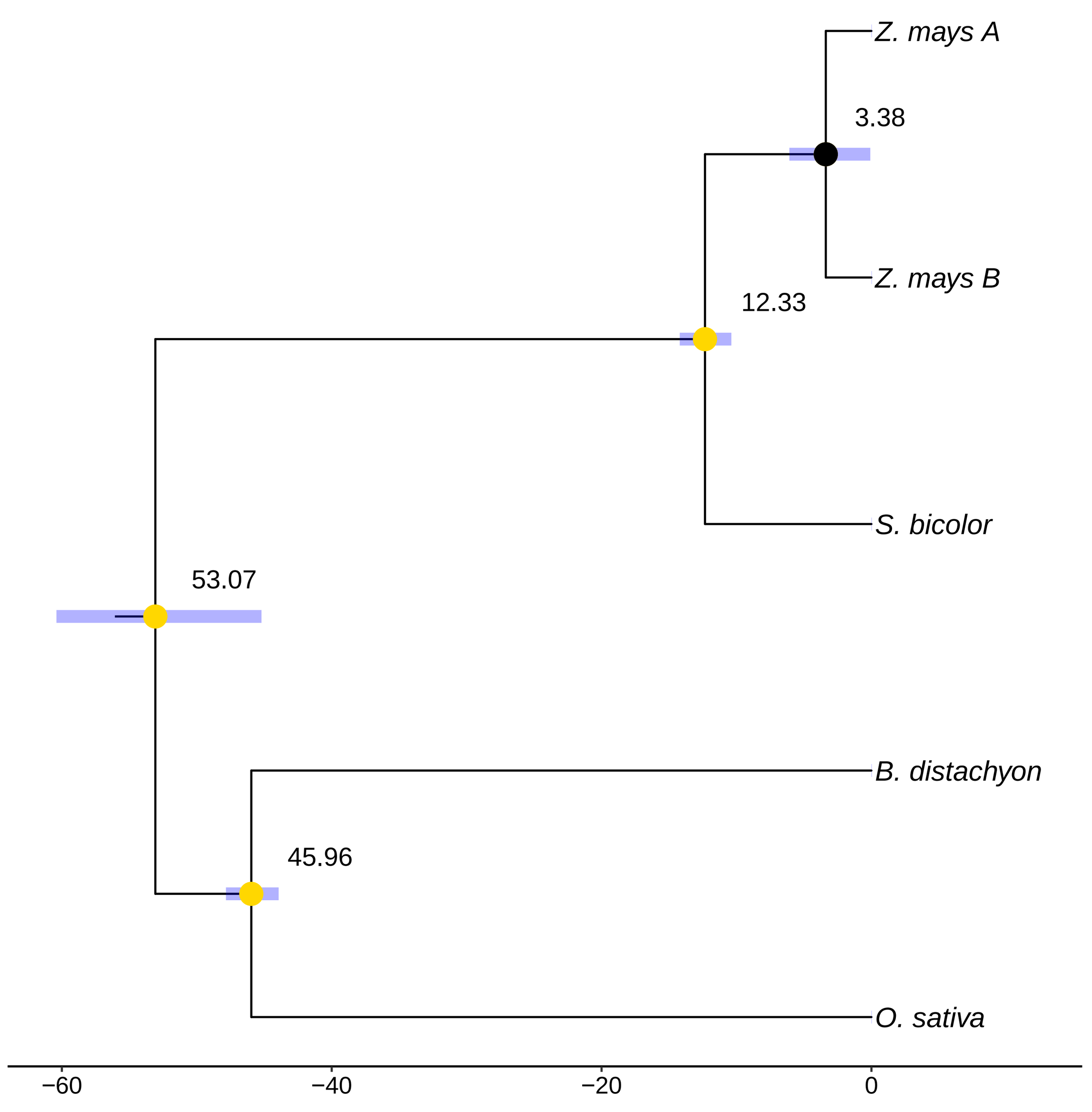

Zm00044a000386_T001

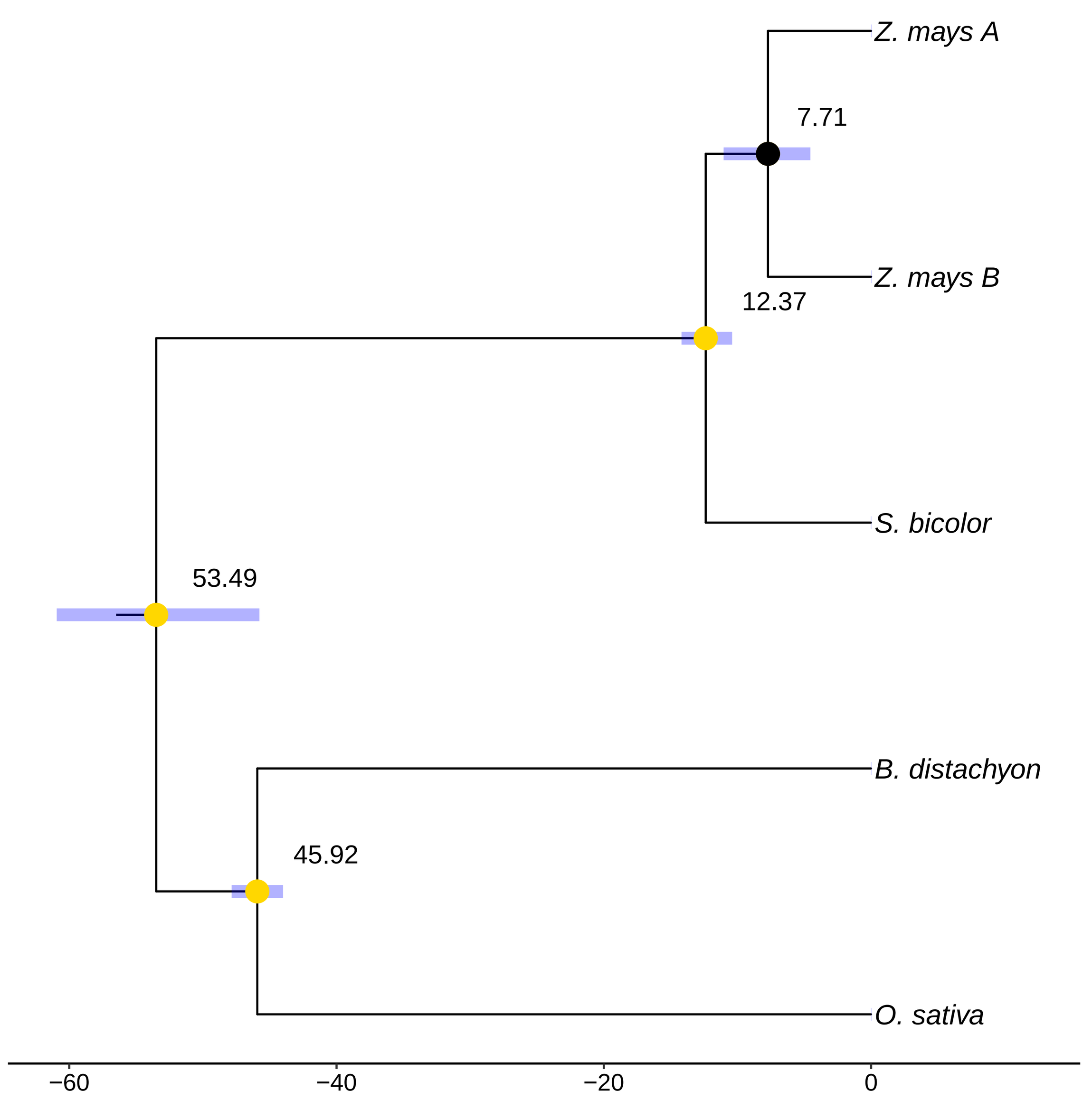

Zm00044a000389_T001

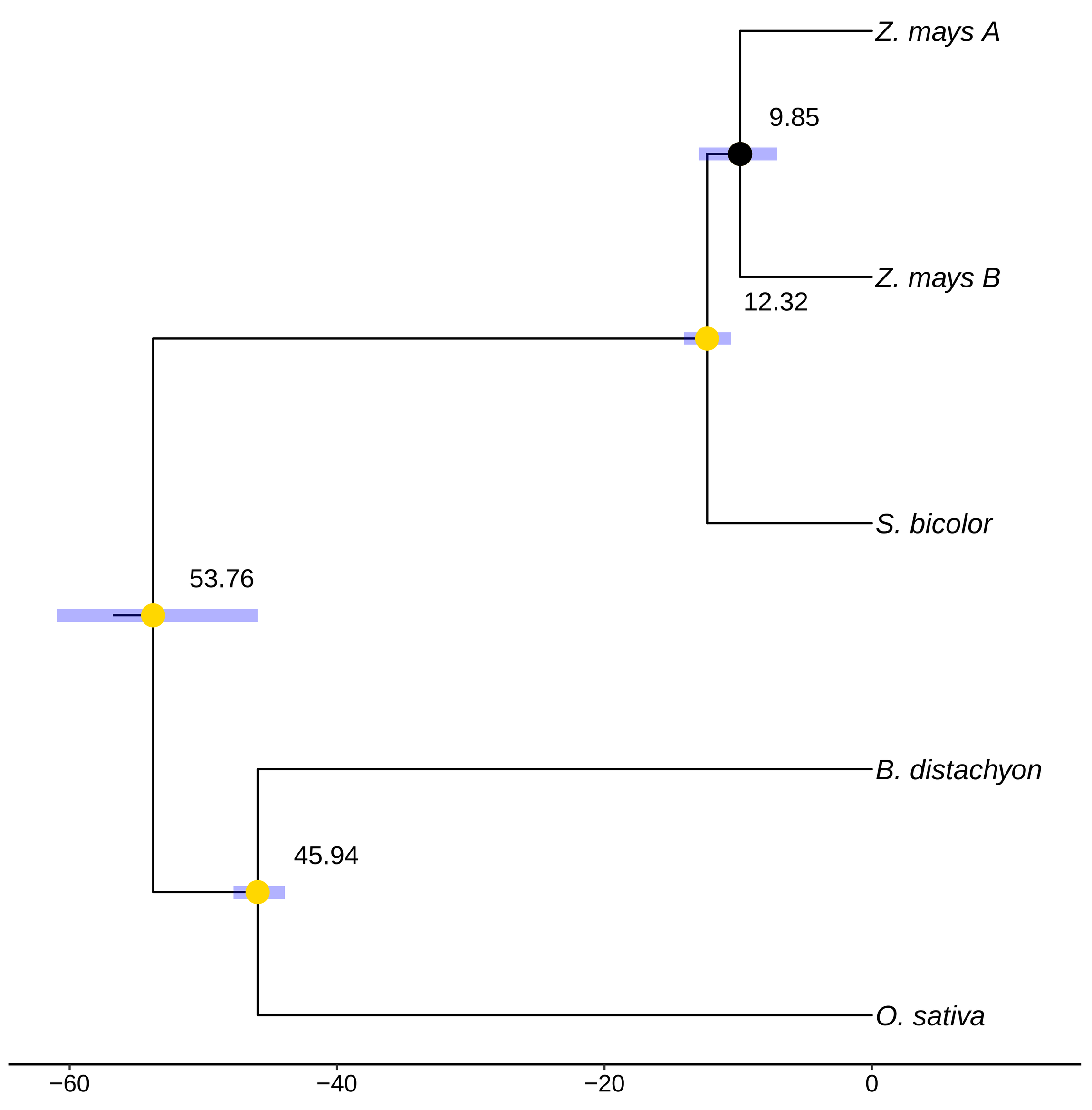

Zm00044a000393_T001

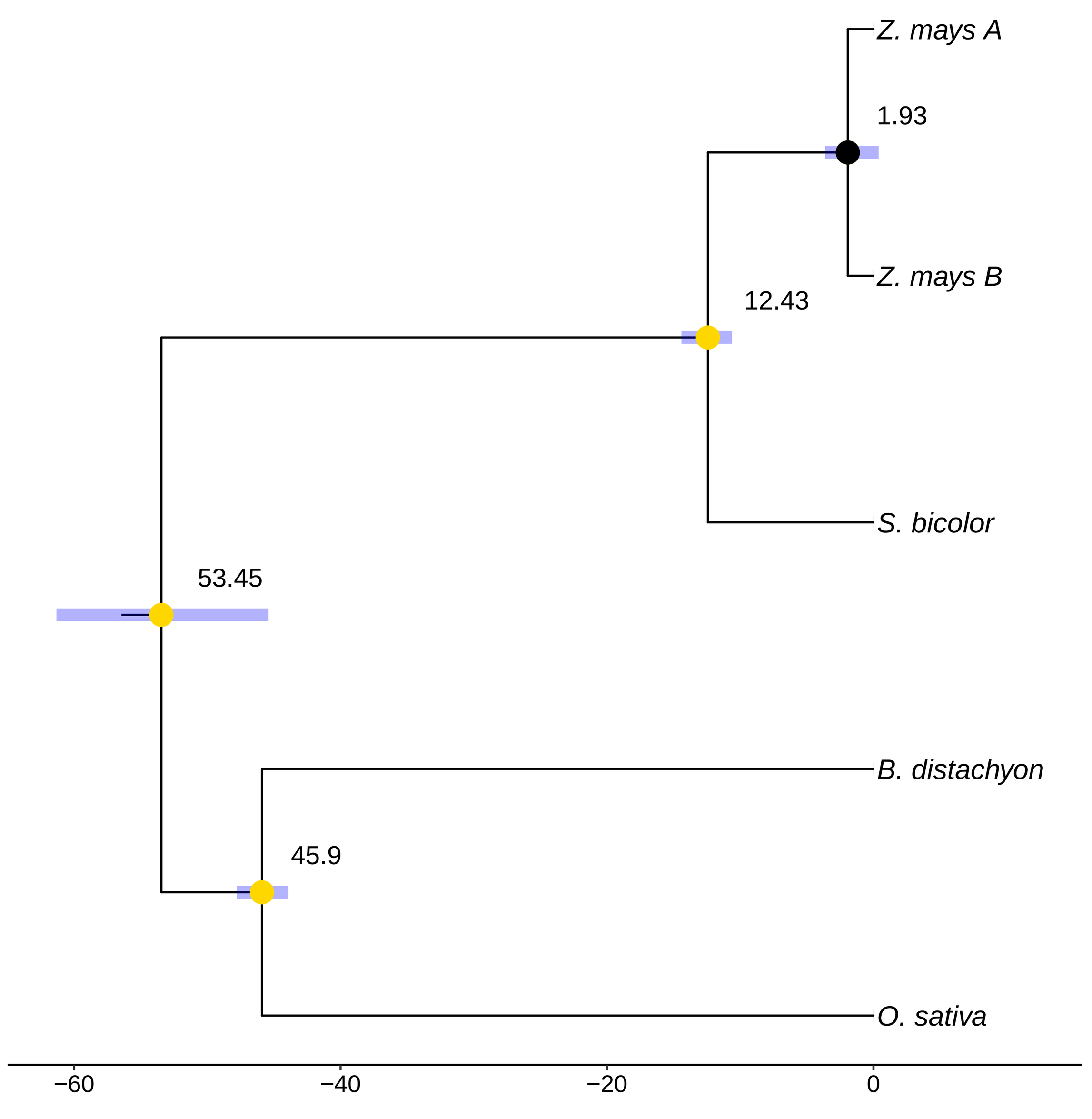

Zm00044a000400_T001

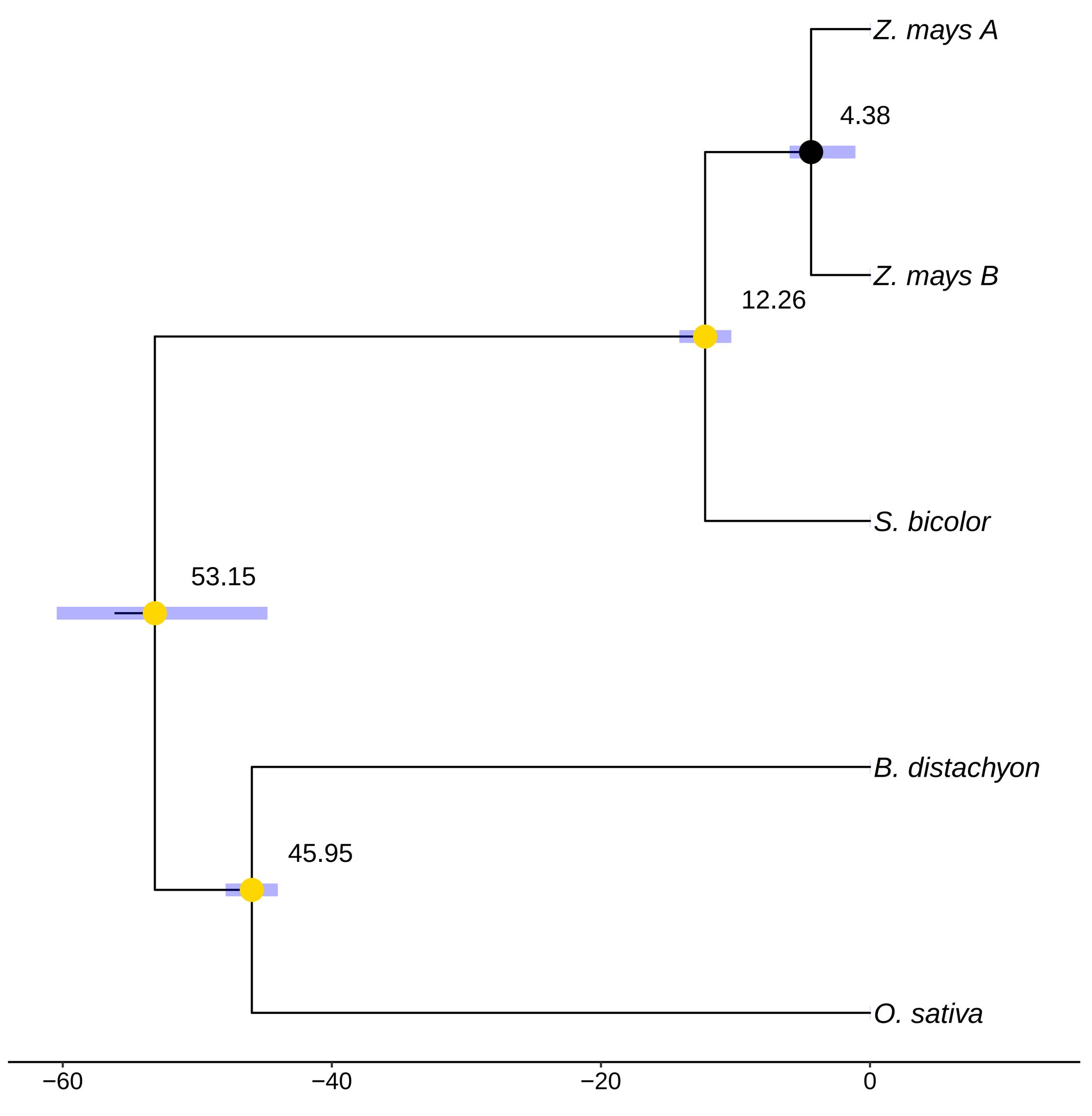

Zm00044a000402_T001

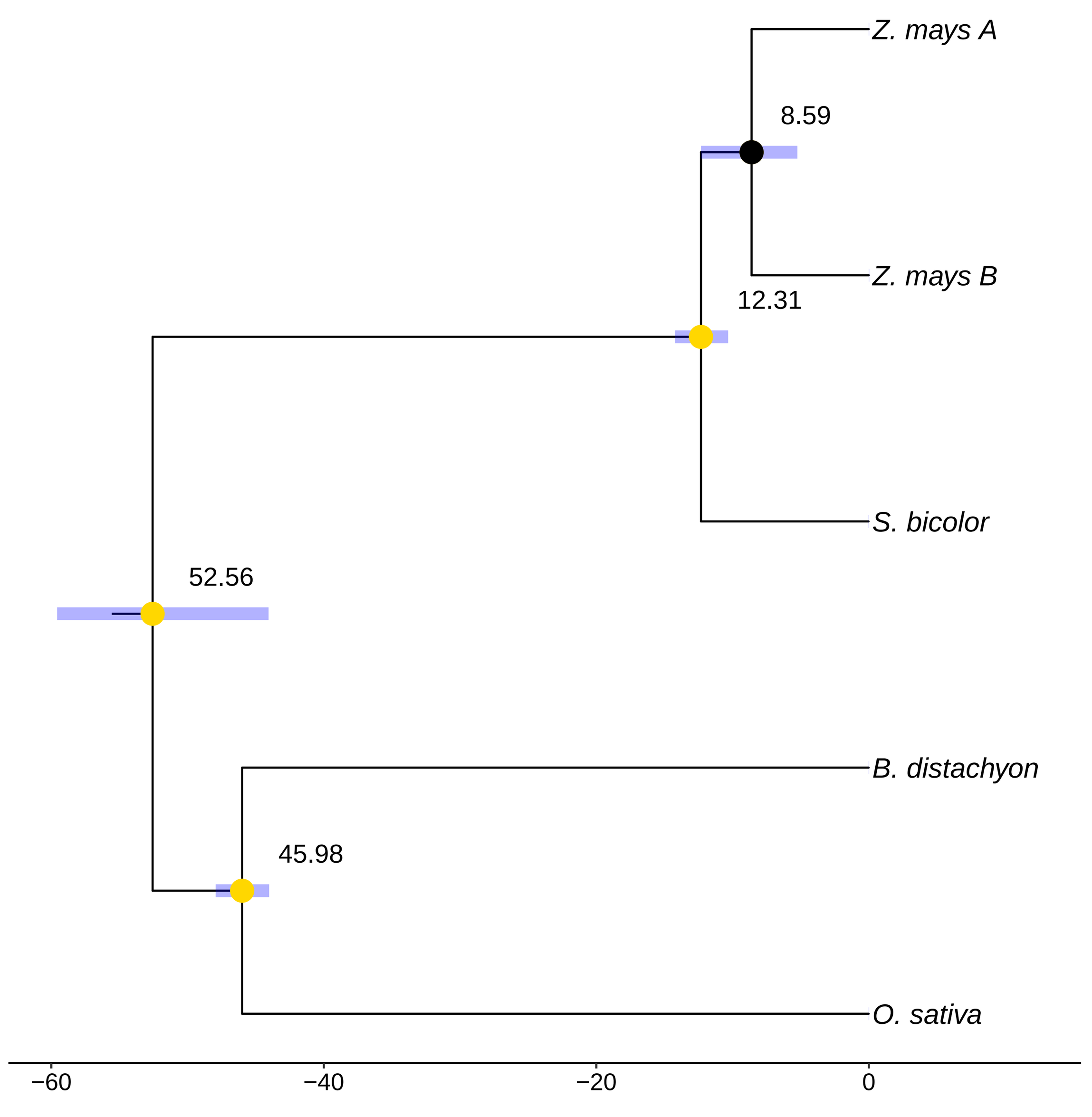

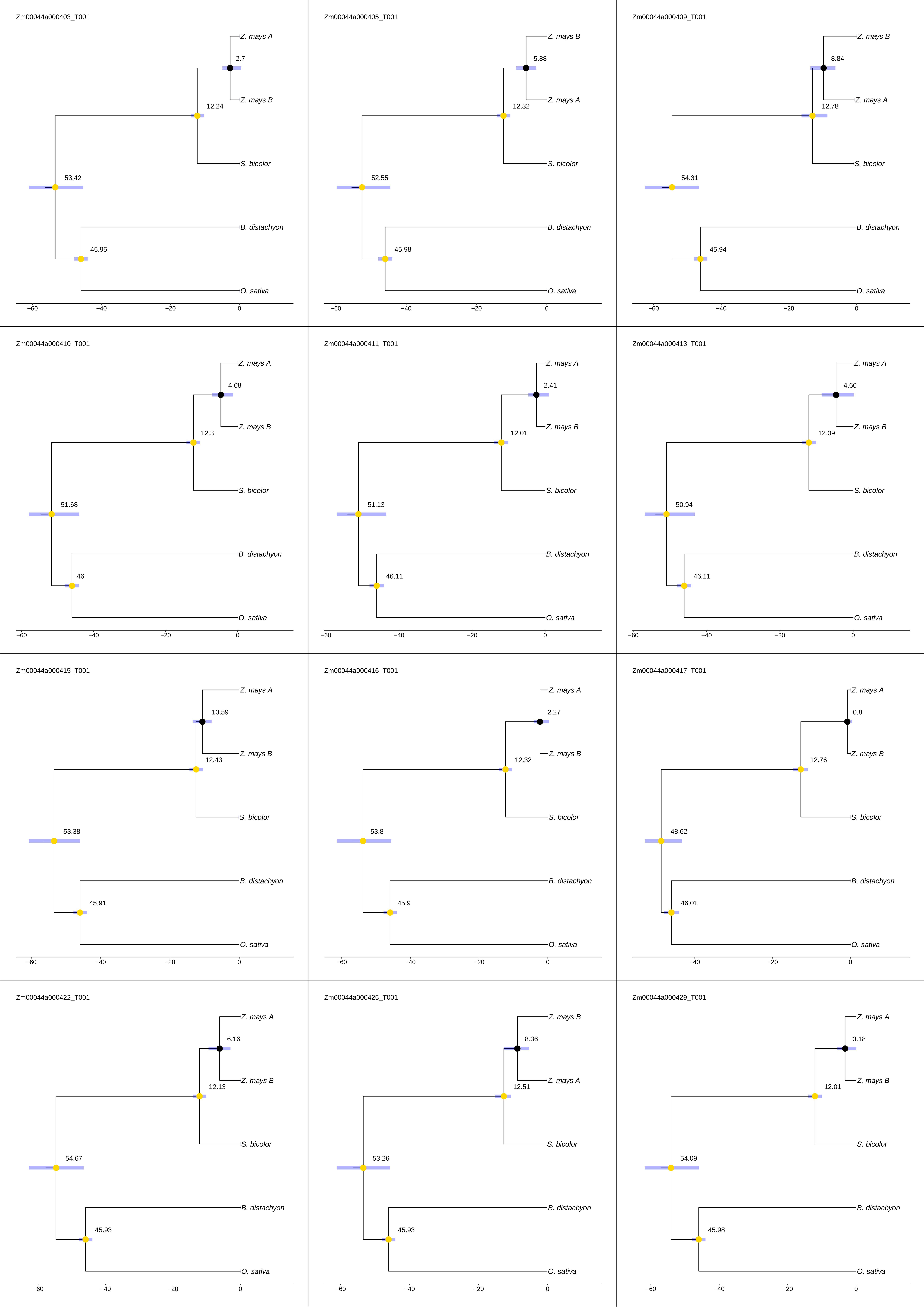
Zm00044a000403_T001
Z. mays A
2.7
Z. mays B
12.24
S. bicolor
53.42
B. distachyon
45.95
O. sativa
−60 −40 −20 0
Zm00044a000405_T001
Z. mays B
5.88
Z. mays A
12.32
S. bicolor
52.55
B. distachyon
45.98
O. sativa
−60 −40 −20 0
Zm00044a000409_T001
Z. mays B
8.84
Z. mays A
12.78
S. bicolor
54.31
B. distachyon
45.94
O. sativa
−60 −40 −20 0
Zm00044a000410_T001
Z. mays A
4.68
Z. mays B
12.3
S. bicolor
51.68
B. distachyon
46
O. sativa
−60 −40 −20 0
Zm00044a000411_T001
Z. mays A
2.41
Z. mays B
12.01
S. bicolor
51.13
B. distachyon
46.11
O. sativa
−60 −40 −20 0
Zm00044a000413_T001
Z. mays A
4.66
Z. mays B
12.09
S. bicolor
50.94
B. distachyon
46.11
O. sativa
−60 −40 −20 0
Zm00044a000415_T001
Z. mays A
10.59
Z. mays B
12.43
S. bicolor
53.38
B. distachyon
45.91
O. sativa
−60 −40 −20 0
Zm00044a000416_T001
Z. mays A
2.27
Z. mays B
12.32
S. bicolor
53.8
B. distachyon
45.9
O. sativa
−60 −40 −20 0
Zm00044a000417_T001
Z. mays A
0.8
Z. mays B
12.76
S. bicolor
48.62
B. distachyon
46.01
O. sativa
−40 −20 0
Zm00044a000422_T001
Z. mays A
6.16
Z. mays B
12.13
S. bicolor
54.67
B. distachyon
45.93
O. sativa
−60 −40 −20 0
Zm00044a000425_T001
Z. mays B
8.36
Z. mays A
12.51
S. bicolor
53.26
B. distachyon
45.93
O. sativa
−60 −40 −20 0
Zm00044a000429_T001
Z. mays A
3.18
Z. mays B
12.01
S. bicolor
54.09
B. distachyon
45.98
O. sativa
−60 −40 −20 0

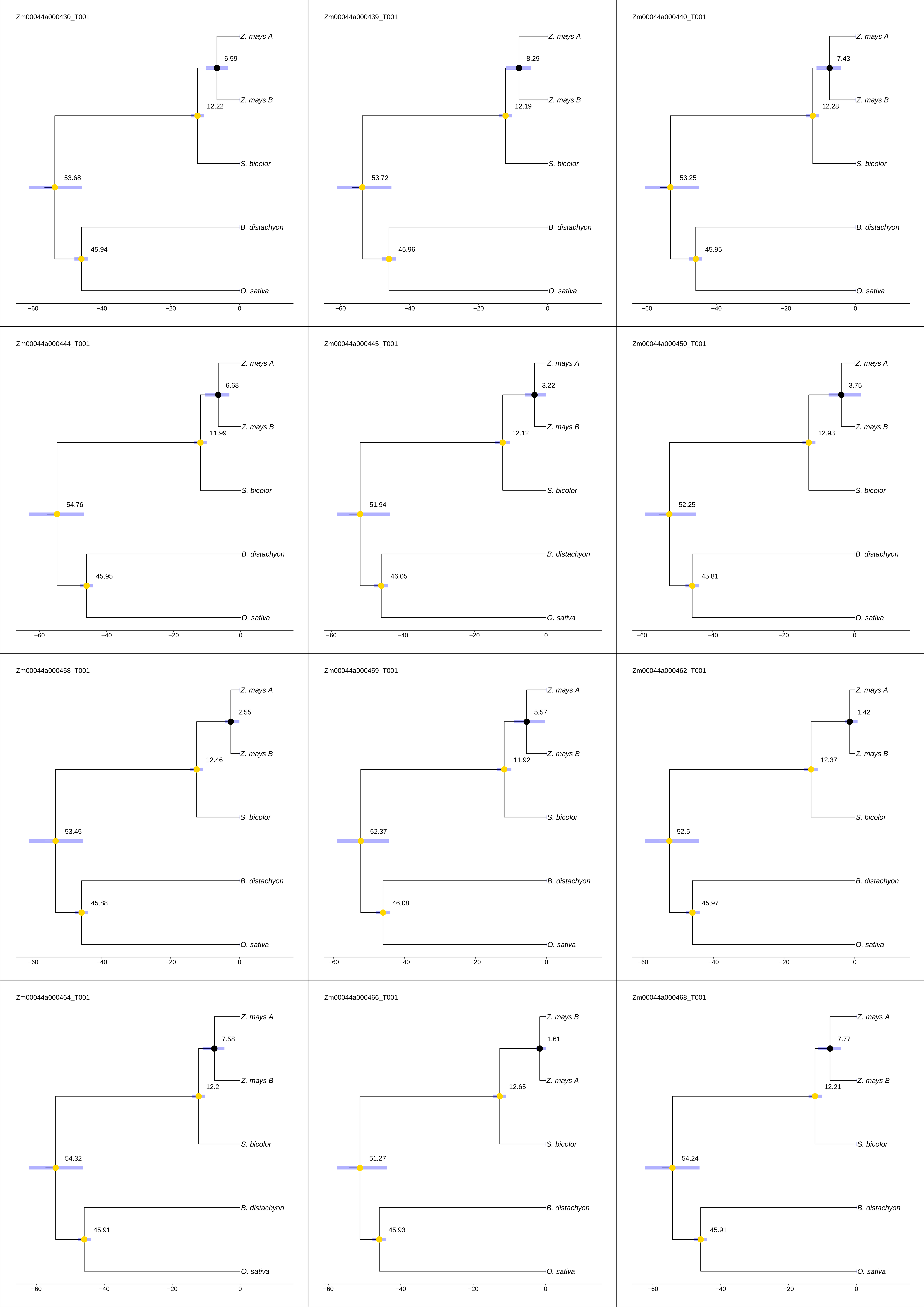
Zm00044a000430_T001
Z. mays A
6.59
Z. mays B
12.22
S. bicolor
53.68
B. distachyon
45.94
O. sativa
−60
−40
−20
0
Zm00044a000439_T001
Z. mays A
8.29
Z. mays B
12.19
S. bicolor
53.72
B. distachyon
45.96
O. sativa
−60
−40
−20
0
Zm00044a000440_T001
Z. mays A
7.43
Z. mays B
12.28
S. bicolor
53.25
B. distachyon
45.95
O. sativa
−60
−40
−20
0
Zm00044a000444_T001
Z. mays A
6.68
Z. mays B
11.99
S. bicolor
54.76
B. distachyon
45.95
O. sativa
−60
−40
−20
0
Zm00044a000445_T001
Z. mays A
3.22
Z. mays B
12.12
S. bicolor
51.94
B. distachyon
46.05
O. sativa
−60
−40
−20
0
Zm00044a000450_T001
Z. mays A
3.75
Z. mays B
12.93
S. bicolor
52.25
B. distachyon
45.81
O. sativa
−60
−40
−20
0
Zm00044a000458_T001
Z. mays A
2.55
Z. mays B
12.46
S. bicolor
53.45
B. distachyon
45.88
O. sativa
−60
−40
−20
0
Zm00044a000459_T001
Z. mays A
5.57
Z. mays B
11.92
S. bicolor
52.37
B. distachyon
46.08
O. sativa
−60
−40
−20
0
Zm00044a000462_T001
Z. mays A
1.42
Z. mays B
12.37
S. bicolor
52.5
B. distachyon
45.97
O. sativa
−60
−40
−20
0
Zm00044a000464_T001
Z. mays A
7.58
Z. mays B
12.2
S. bicolor
54.32
B. distachyon
45.91
O. sativa
−60
−40
−20
0
Zm00044a000466_T001
Z. mays B
1.61
Z. mays A
12.65
S. bicolor
51.27
B. distachyon
45.93
O. sativa
−60
−40
−20
0
Zm00044a000468_T001
Z. mays A
7.77
Z. mays B
12.21
S. bicolor
54.24
B. distachyon
45.91
O. sativa
−60
−40
−20
0

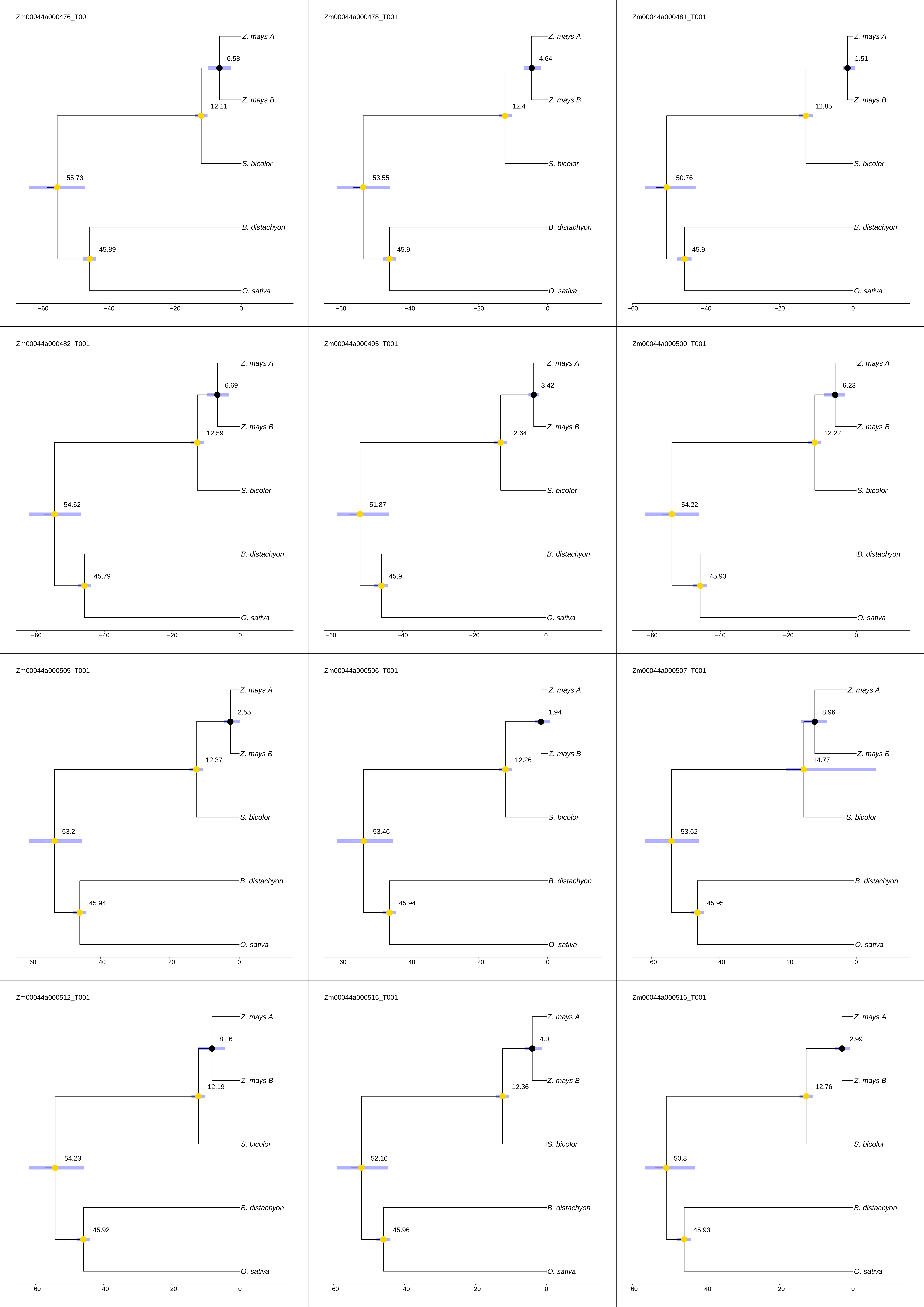
Zm00044a000476_T001
6.58
12.11
55.73
45.89
Zm00044a000478_T001
4.64
12.4
53.55
45.9
Zm00044a000481_T001
1.51
12.85
50.76
45.9
Zm00044a000482_T001
6.69
12.59
54.62
45.79
Zm00044a000495_T001
3.42
12.64
51.87
45.9
Zm00044a000500_T001
6.23
12.22
54.22
45.93
Zm00044a000505_T001
2.55
12.37
53.2
45.94
Zm00044a000506_T001
1.94
12.26
53.46
45.94
Zm00044a000507_T001
8.96
14.77
53.62
45.95
Zm00044a000512_T001
8.16
12.19
54.23
45.92
Zm00044a000515_T001
4.01
12.36
52.16
45.96
Zm00044a000516_T001
2.99
12.76
50.8
45.93
Z. mays A
Z. mays B
S. bicolor
B. distachyon
O. sativa
−60
−40
−20
0

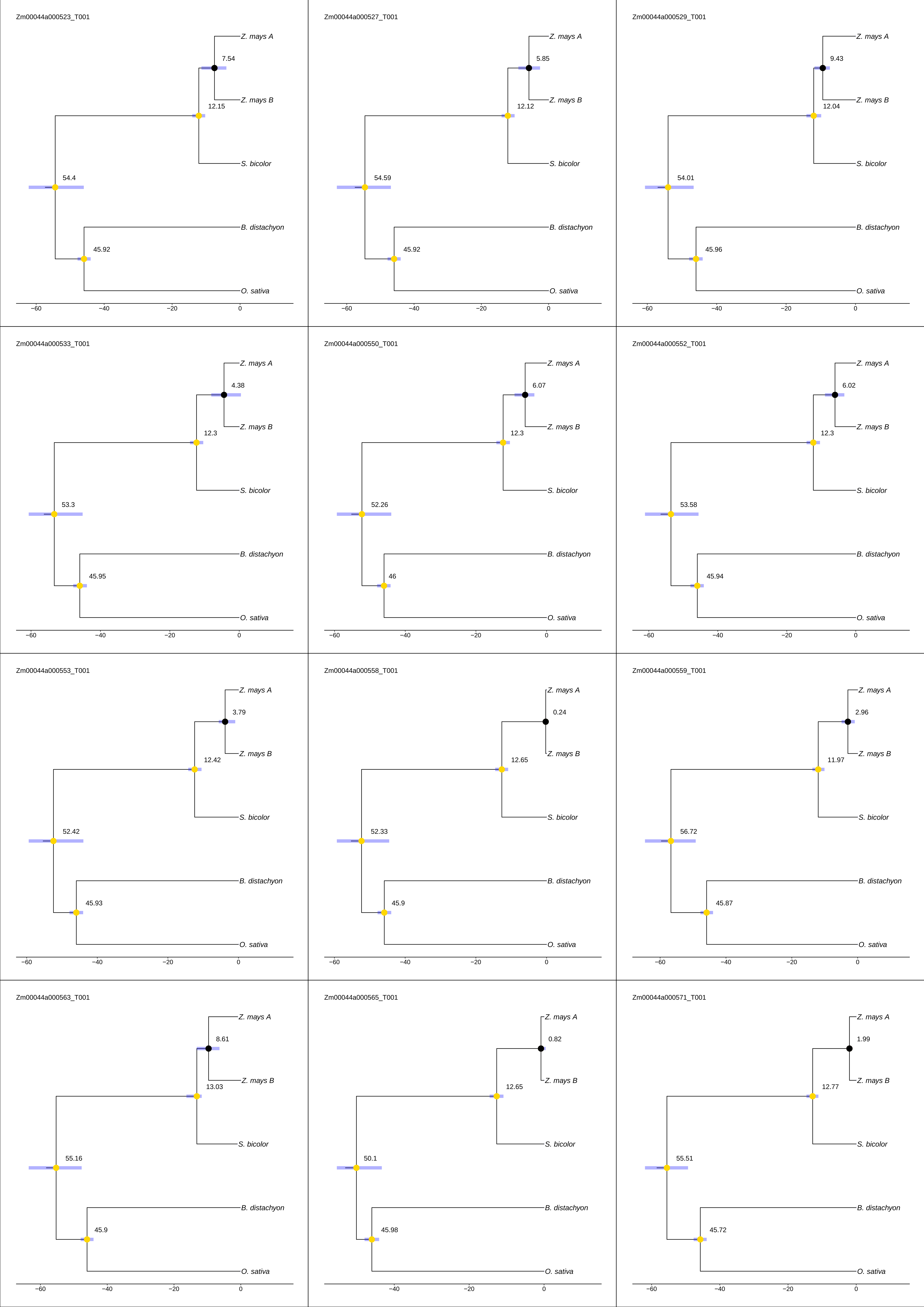

Zm00044a000523_T001
Z. mays A
7.54
Z. mays B
12.15
S. bicolor
54.4
B. distachyon
45.92
O. sativa
−60 −40 −20 0
Zm00044a000527_T001
Z. mays A
5.85
Z. mays B
12.12
S. bicolor
54.59
B. distachyon
45.92
O. sativa
−60 −40 −20 0
Zm00044a000529_T001
Z. mays A
9.43
Z. mays B
12.04
S. bicolor
54.01
B. distachyon
45.96
O. sativa
−60 −40 −20 0
Zm00044a000533_T001
Z. mays A
4.38
Z. mays B
12.3
S. bicolor
53.3
B. distachyon
45.95
O. sativa
−60 −40 −20 0
Zm00044a000550_T001
Z. mays A
6.07
Z. mays B
12.3
S. bicolor
52.26
B. distachyon
46
O. sativa
−60 −40 −20 0
Zm00044a000552_T001
Z. mays A
6.02
Z. mays B
12.3
S. bicolor
53.58
B. distachyon
45.94
O. sativa
−60 −40 −20 0
Zm00044a000553_T001
Z. mays A
3.79
Z. mays B
12.42
S. bicolor
52.42
B. distachyon
45.93
O. sativa
−60 −40 −20 0
Zm00044a000558_T001
Z. mays A
0.24
Z. mays B
12.65
S. bicolor
52.33
B. distachyon
45.9
O. sativa
−60 −40 −20 0
Zm00044a000559_T001
Z. mays A
2.96
Z. mays B
11.97
S. bicolor
56.72
B. distachyon
45.87
O. sativa
−60 −40 −20 0
Zm00044a000563_T001
Z. mays A
8.61
Z. mays B
13.03
S. bicolor
55.16
B. distachyon
45.9
O. sativa
−60 −40 −20 0
Zm00044a000565_T001
Z. mays A
0.82
Z. mays B
12.65
S. bicolor
50.1
B. distachyon
45.98
O. sativa
−40 −20 0
Zm00044a000571_T001
Z. mays A
1.99
Z. mays B
12.77
S. bicolor
55.51
B. distachyon
45.72
O. sativa
−60 −40 −20 0

Zm00044a000576_T001

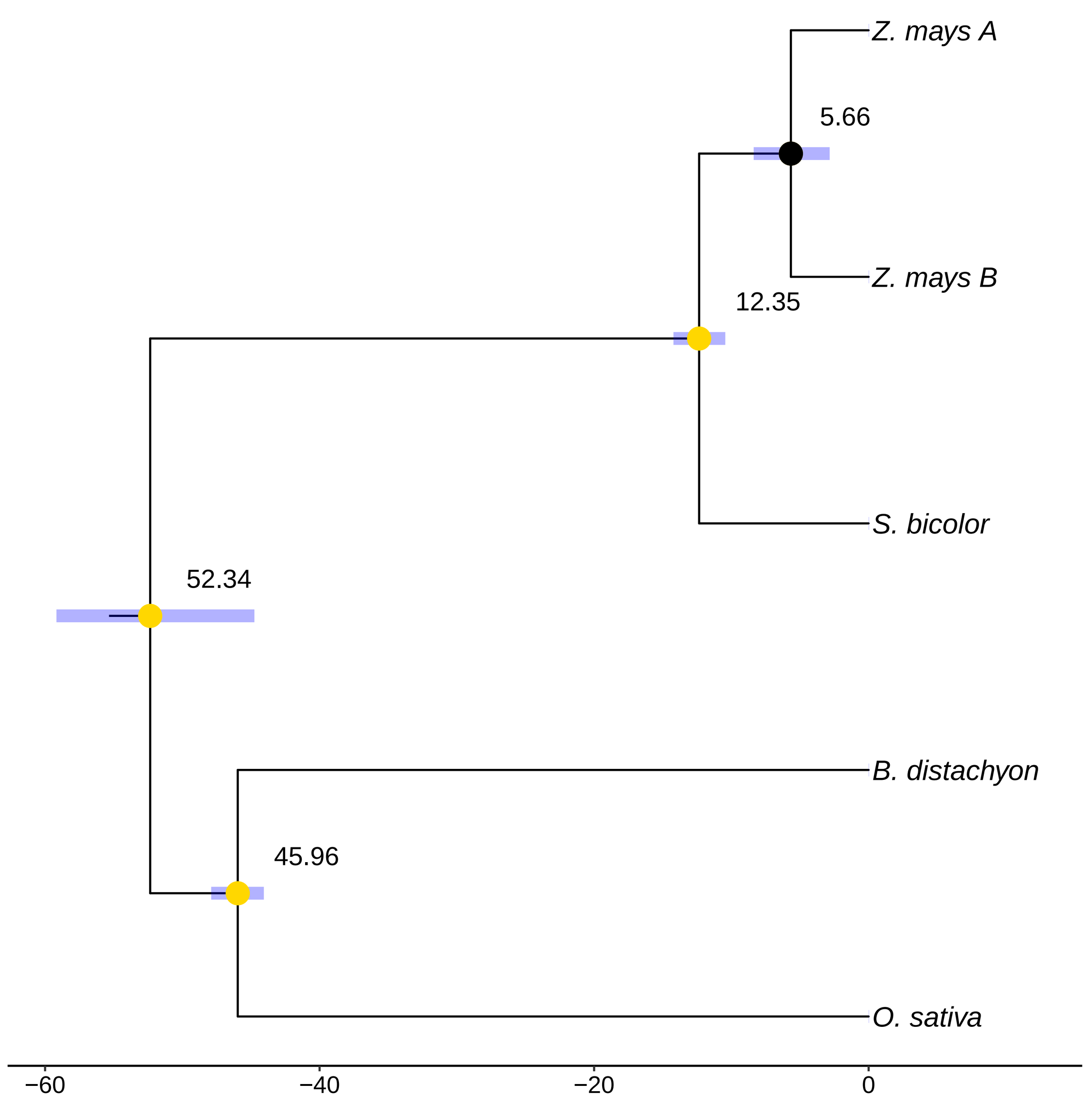

Zm00044a000577_T001

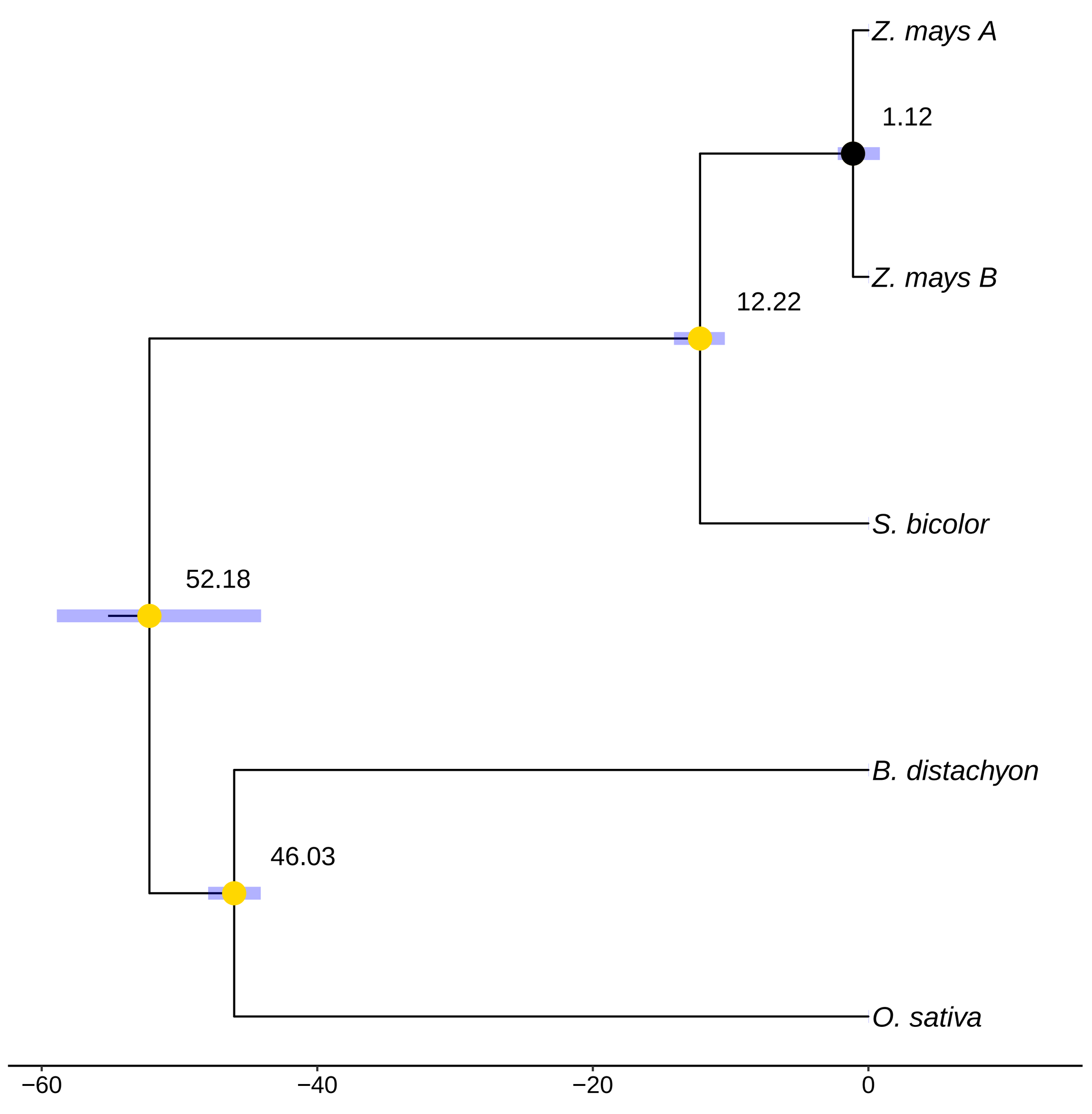

Zm00044a000578_T001

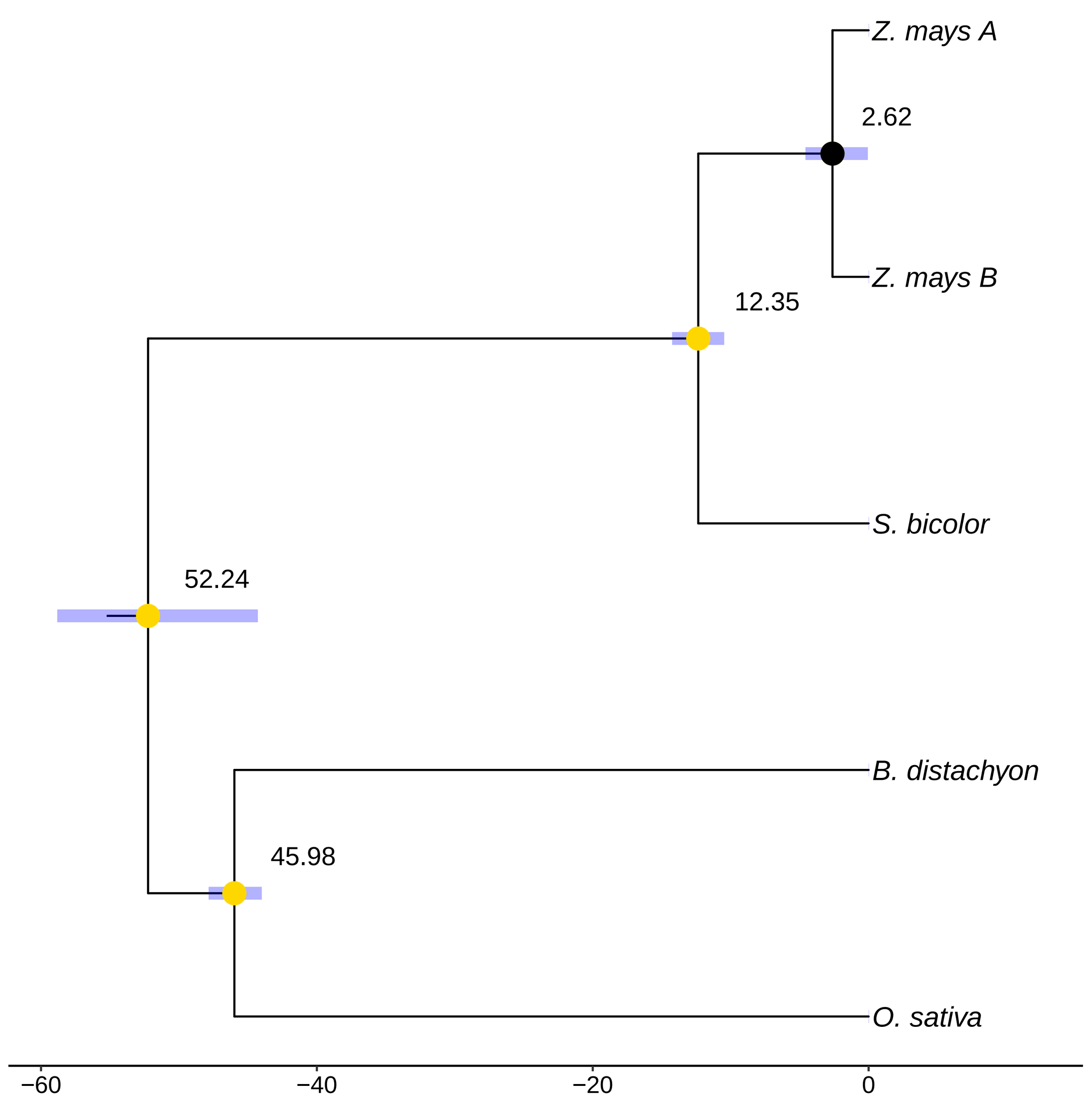

Zm00044a000583_T001

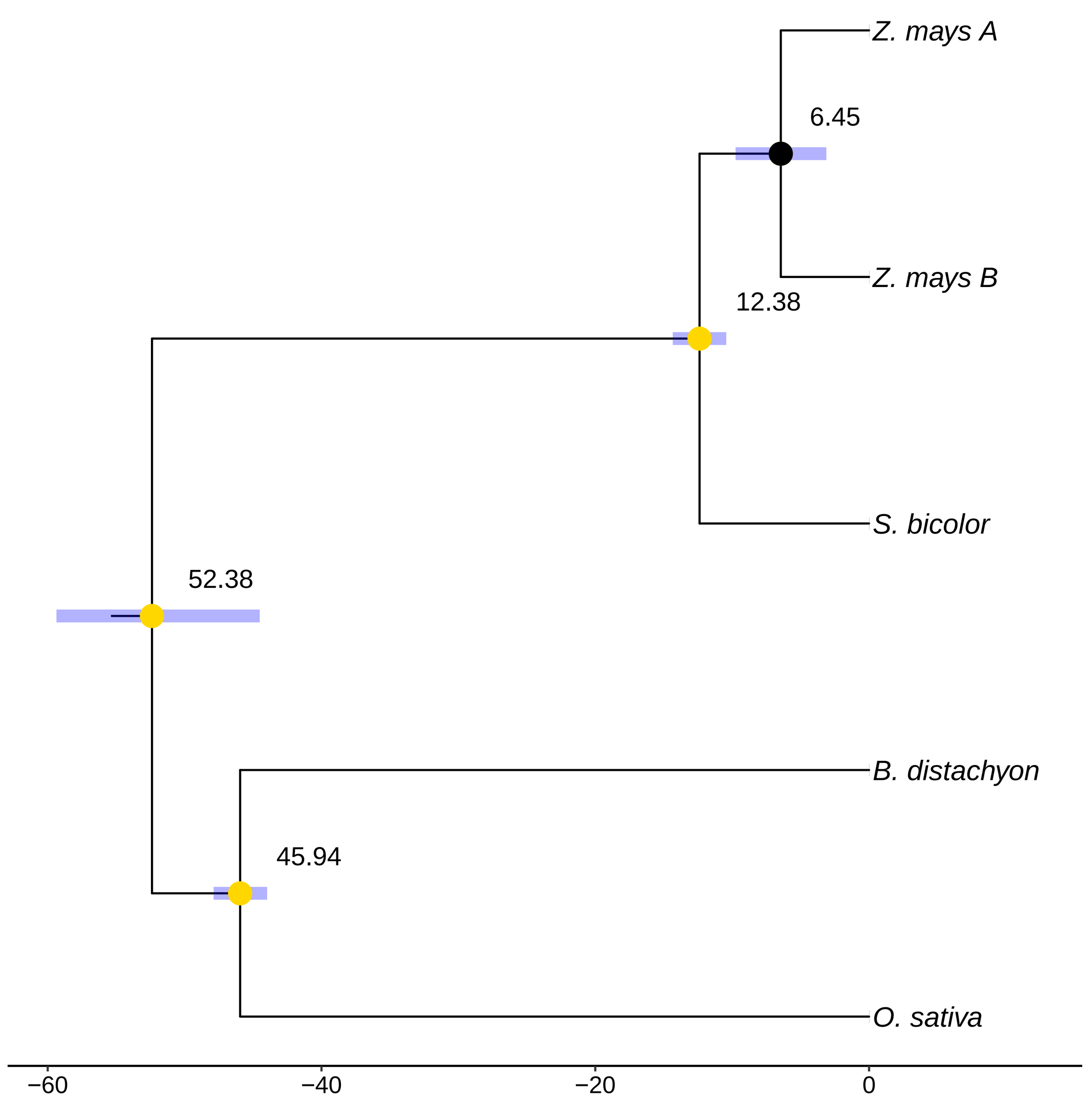

Zm00044a000586_T001

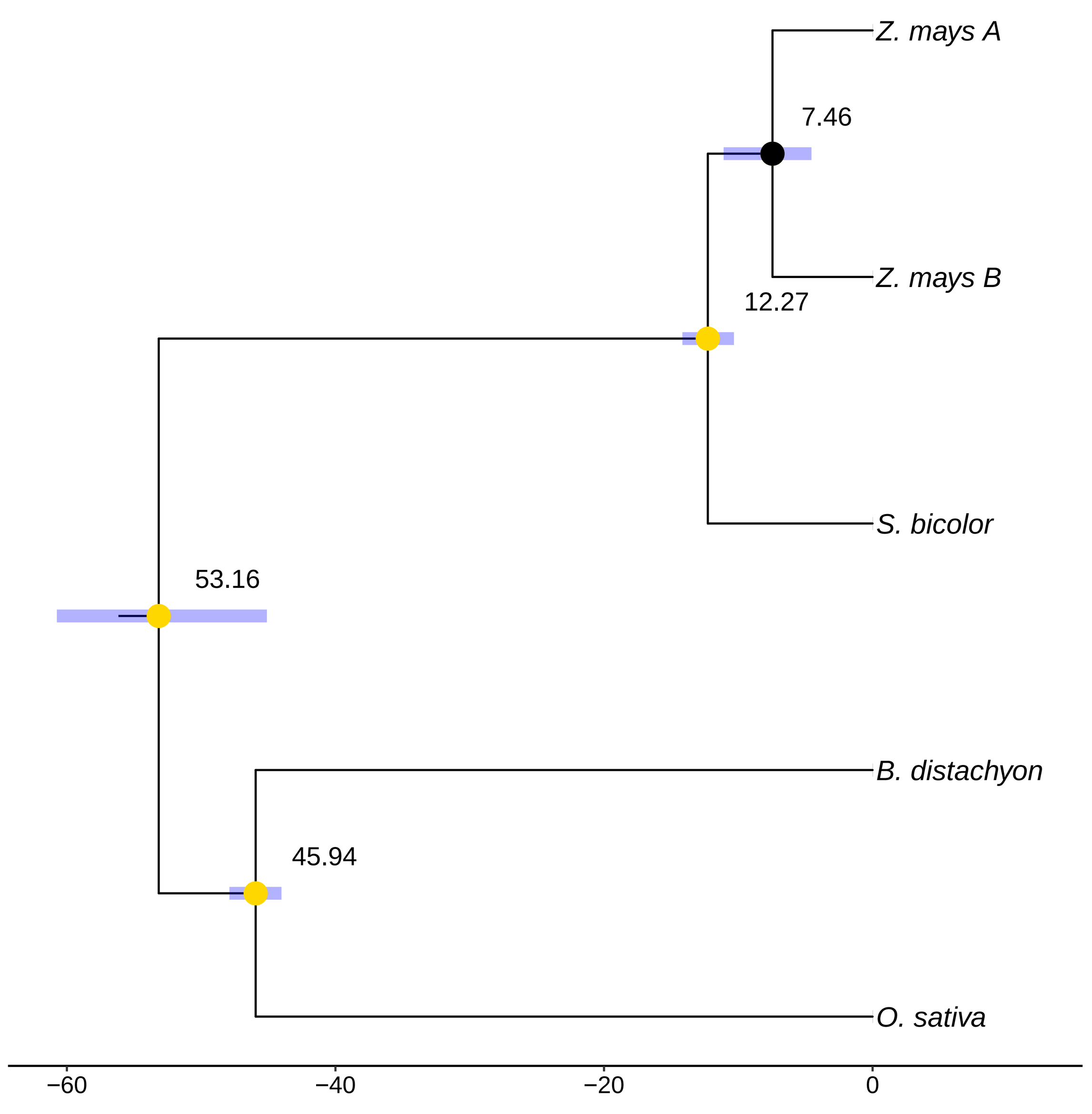

Zm00044a000588_T001

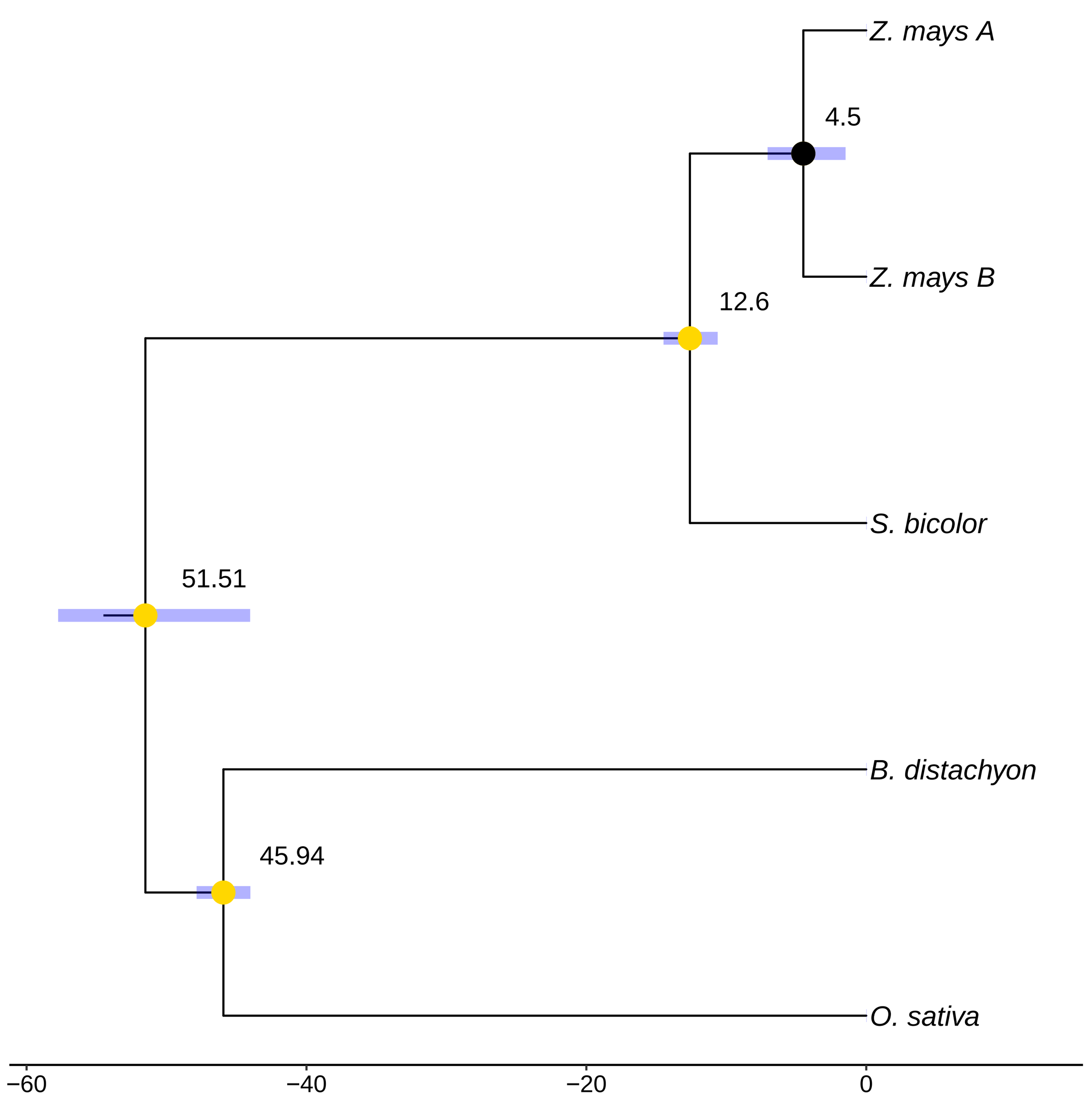

Zm00044a000593_T001

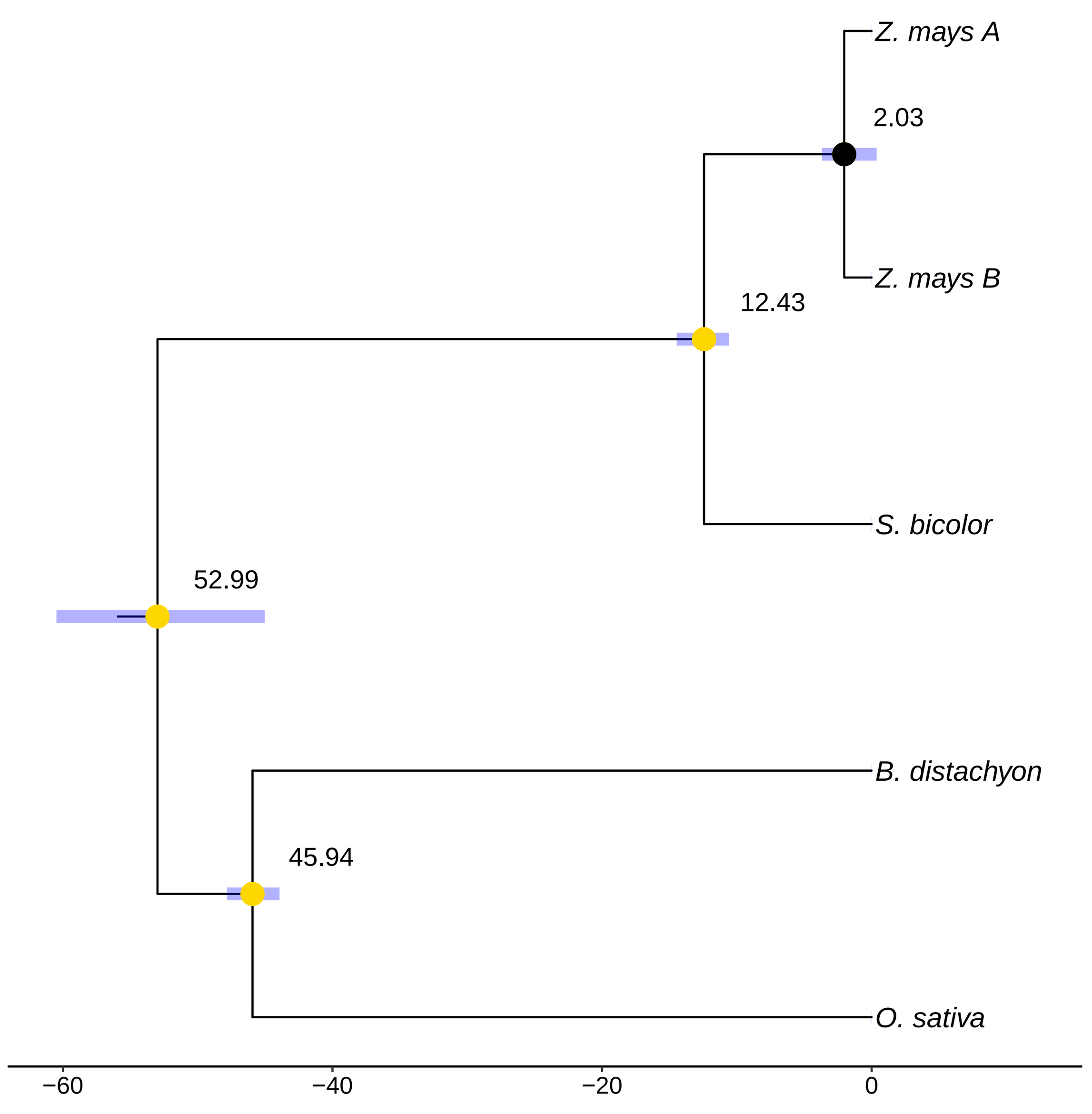

Zm00044a000597_T001

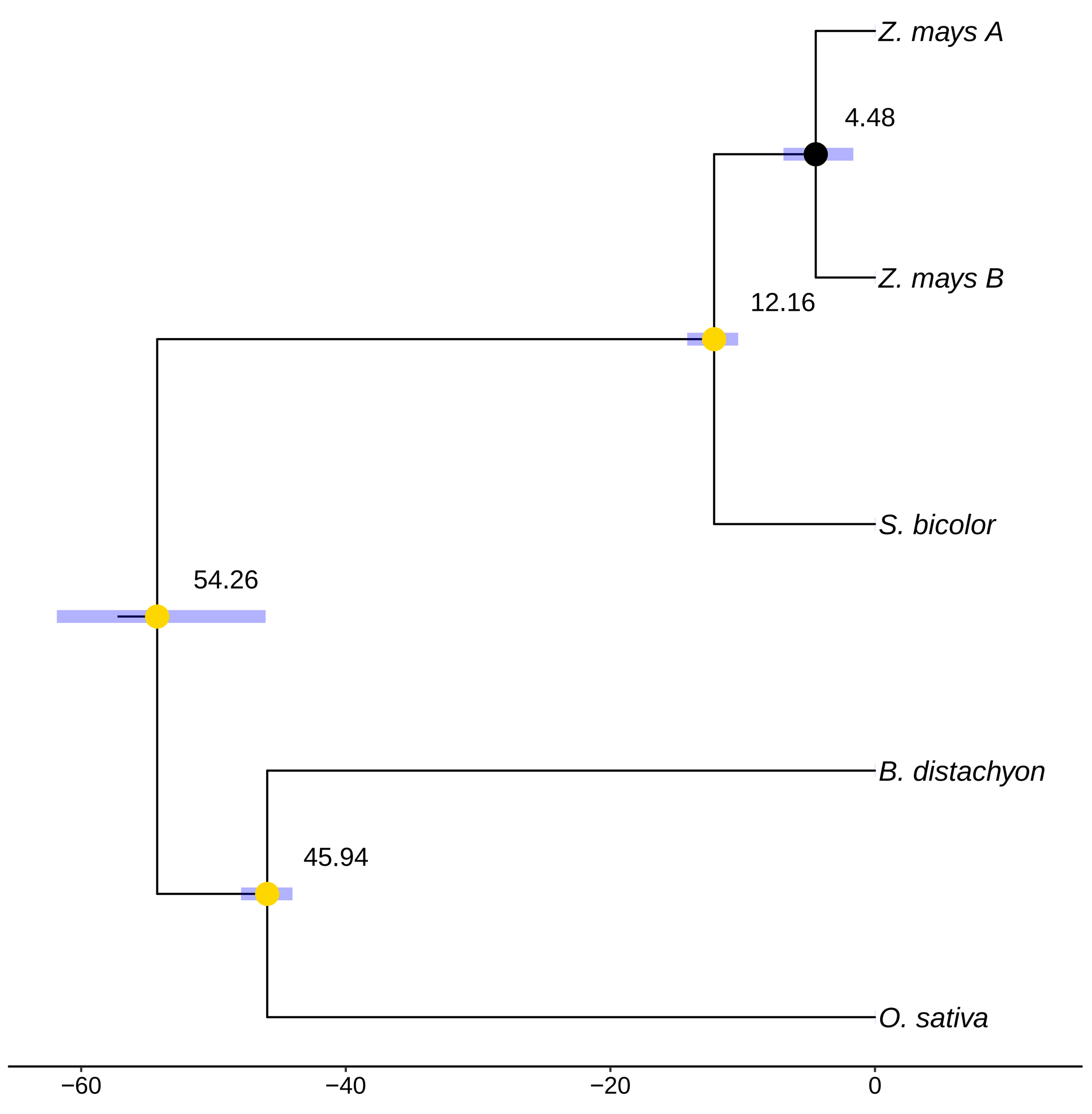

Zm00044a000601_T001

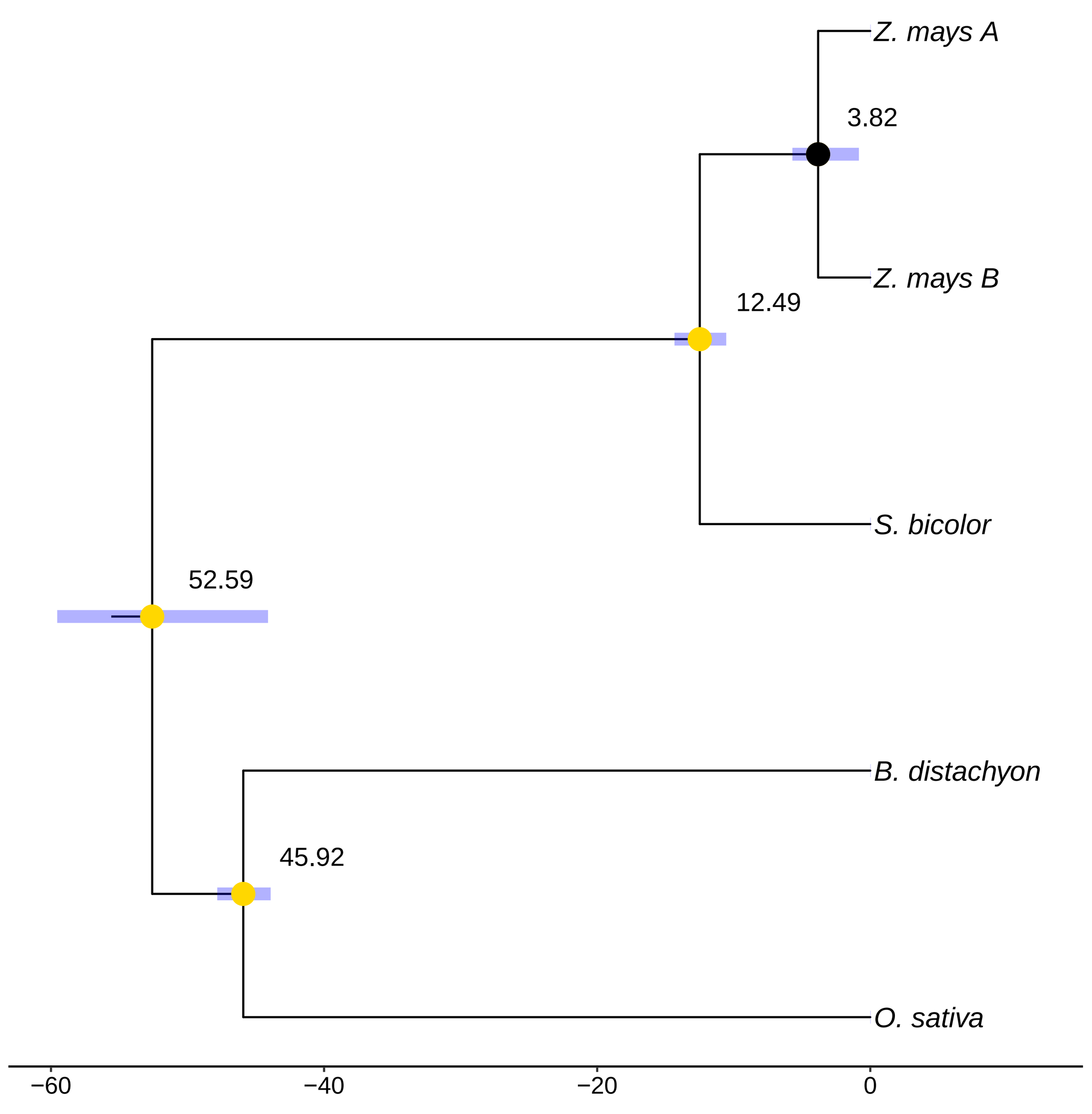

Zm00044a000605_T001

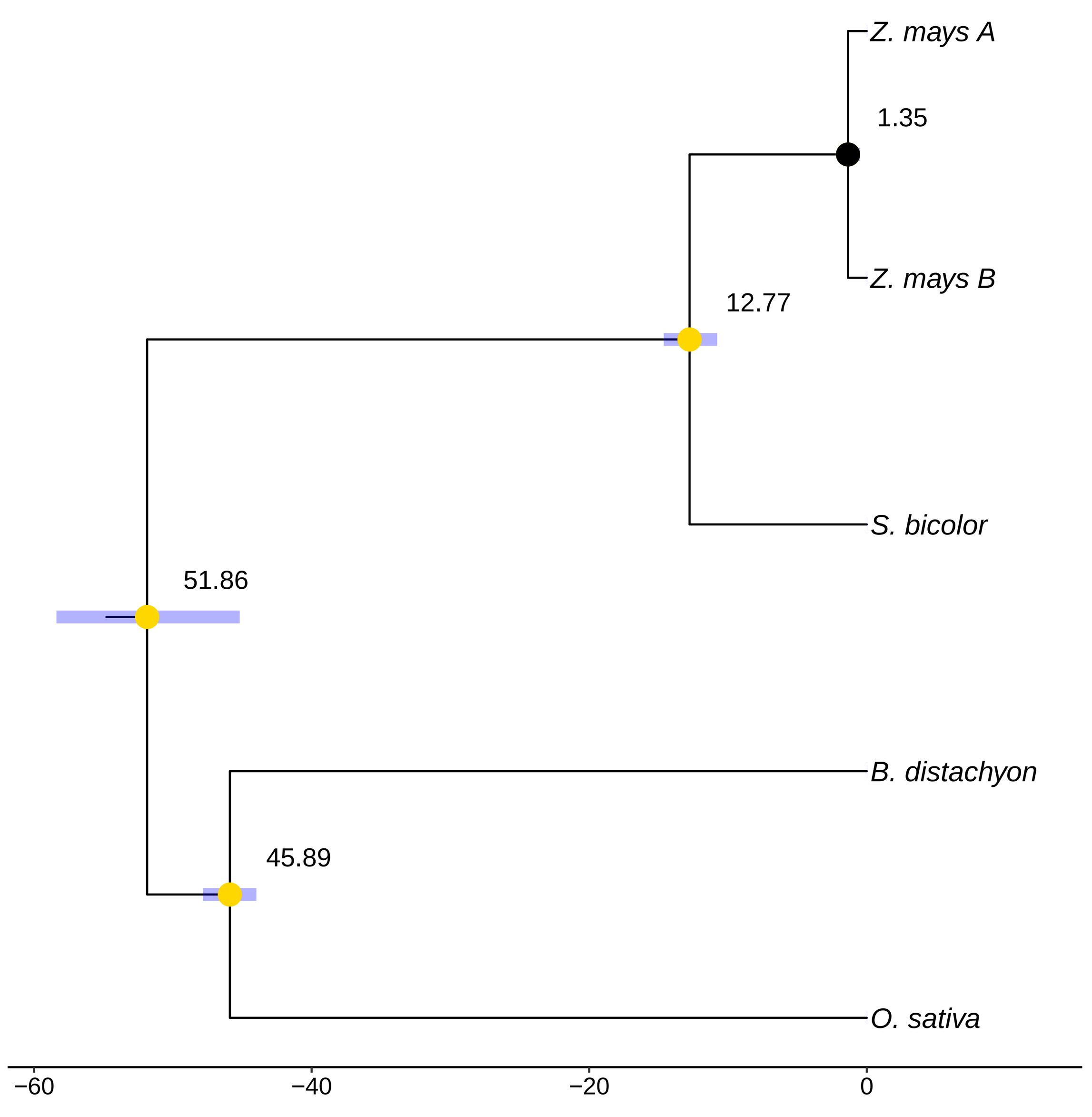

Zm00044a000609_T001

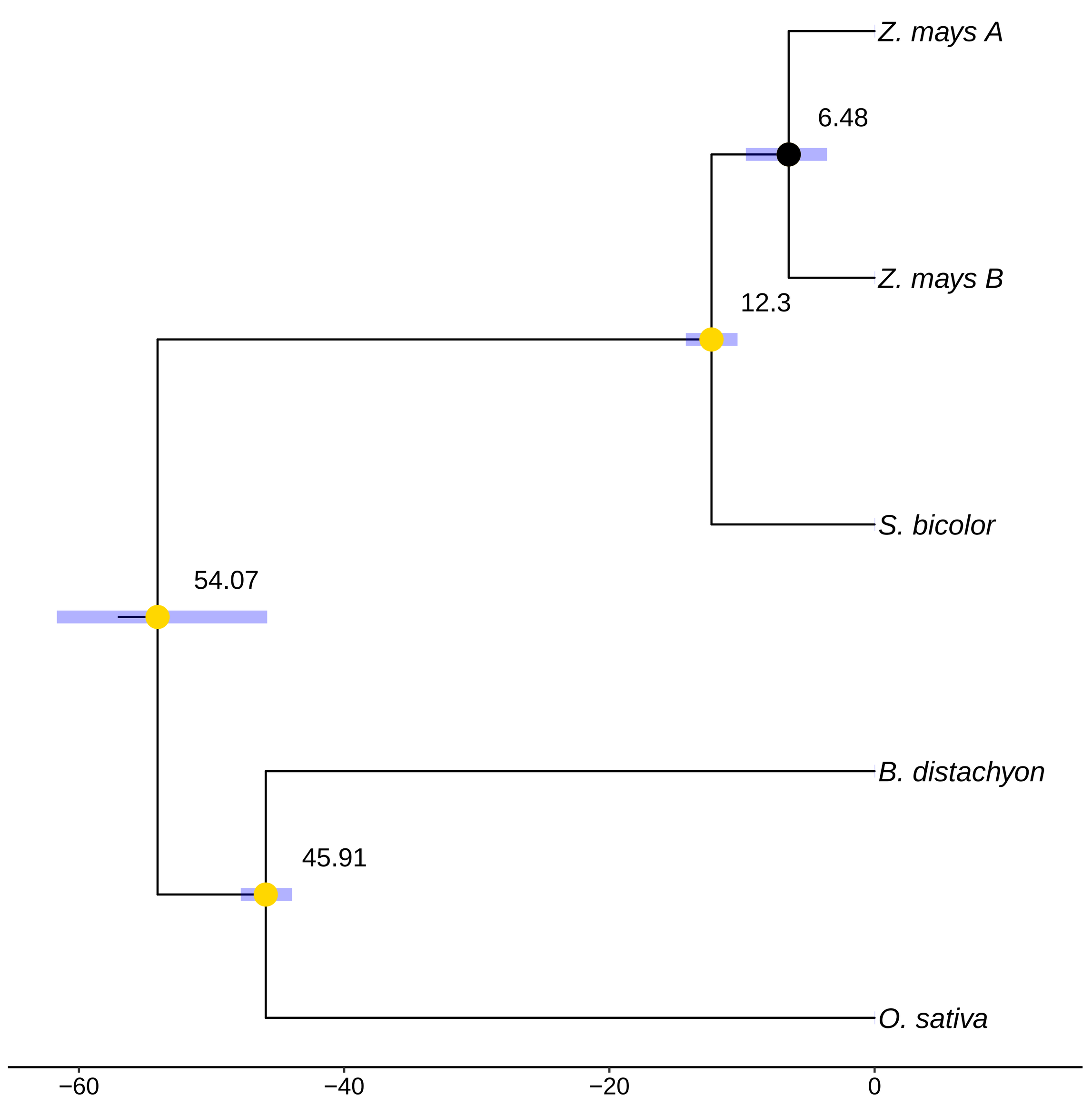

Zm00044a000619_T001

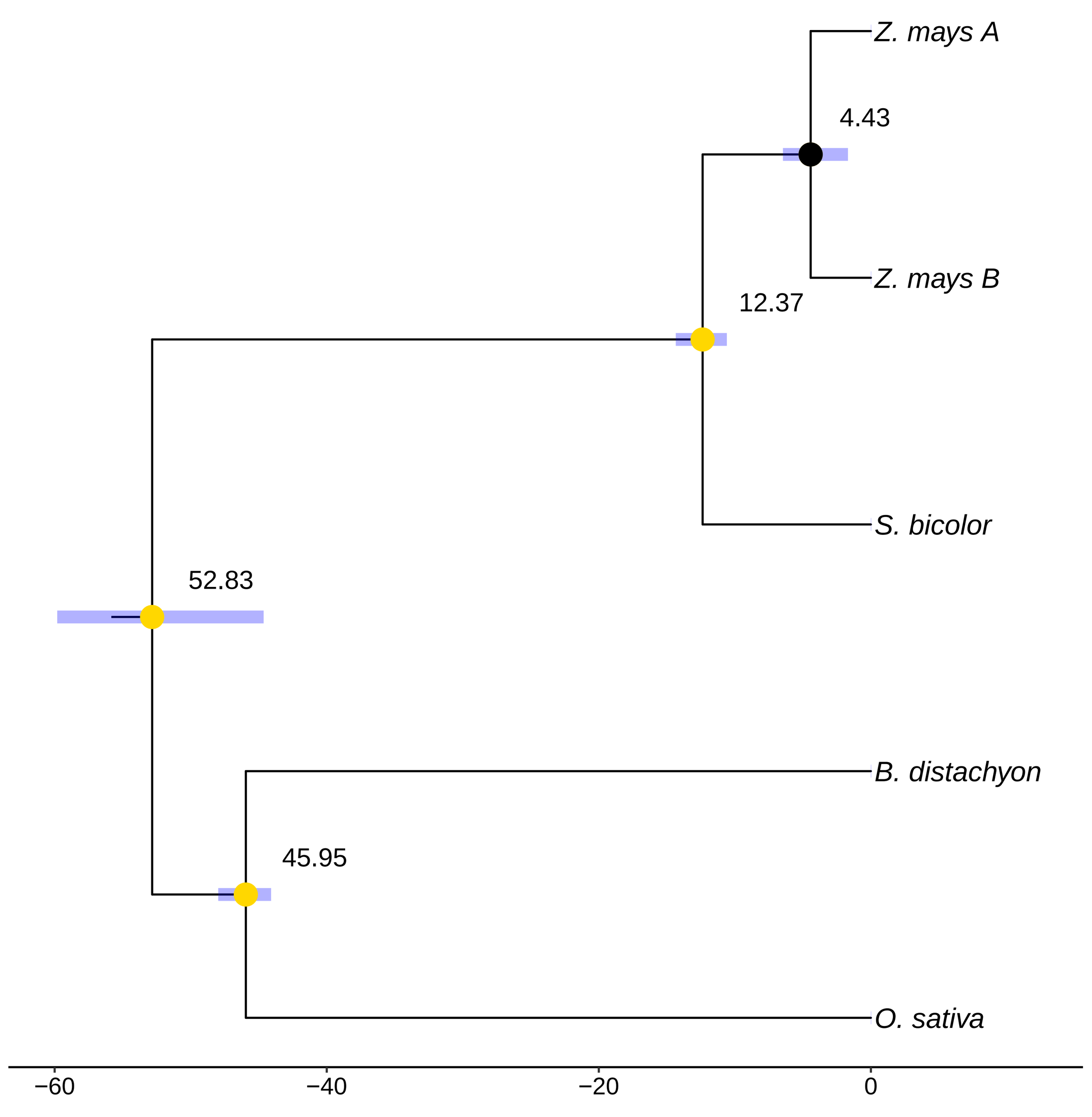

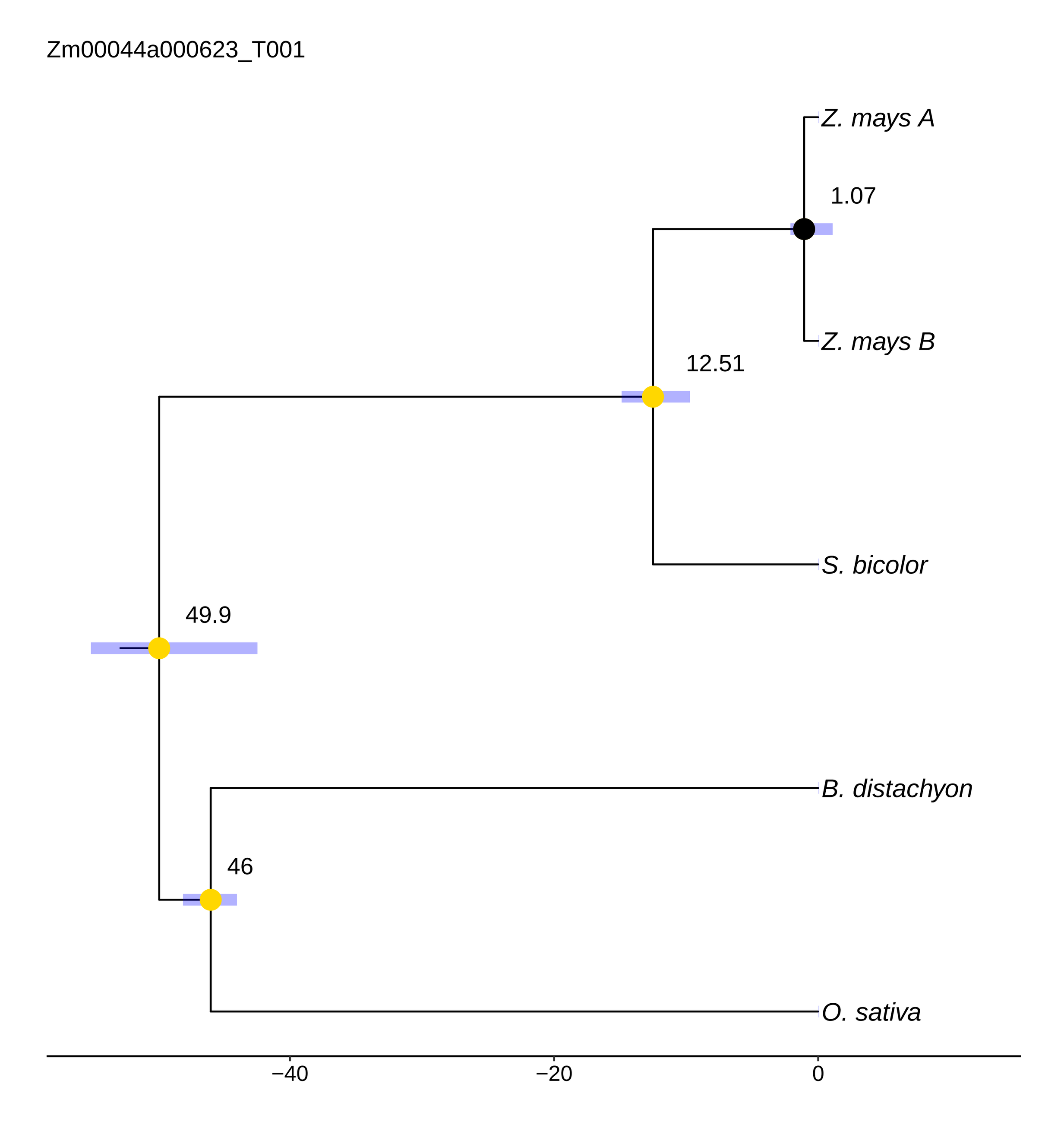
Zm00044a000623_T001
Z. mays A
1.07
Z. mays B
12.51
S. bicolor
49.9
B. distachyon
46
O. sativa
−40
−20
0

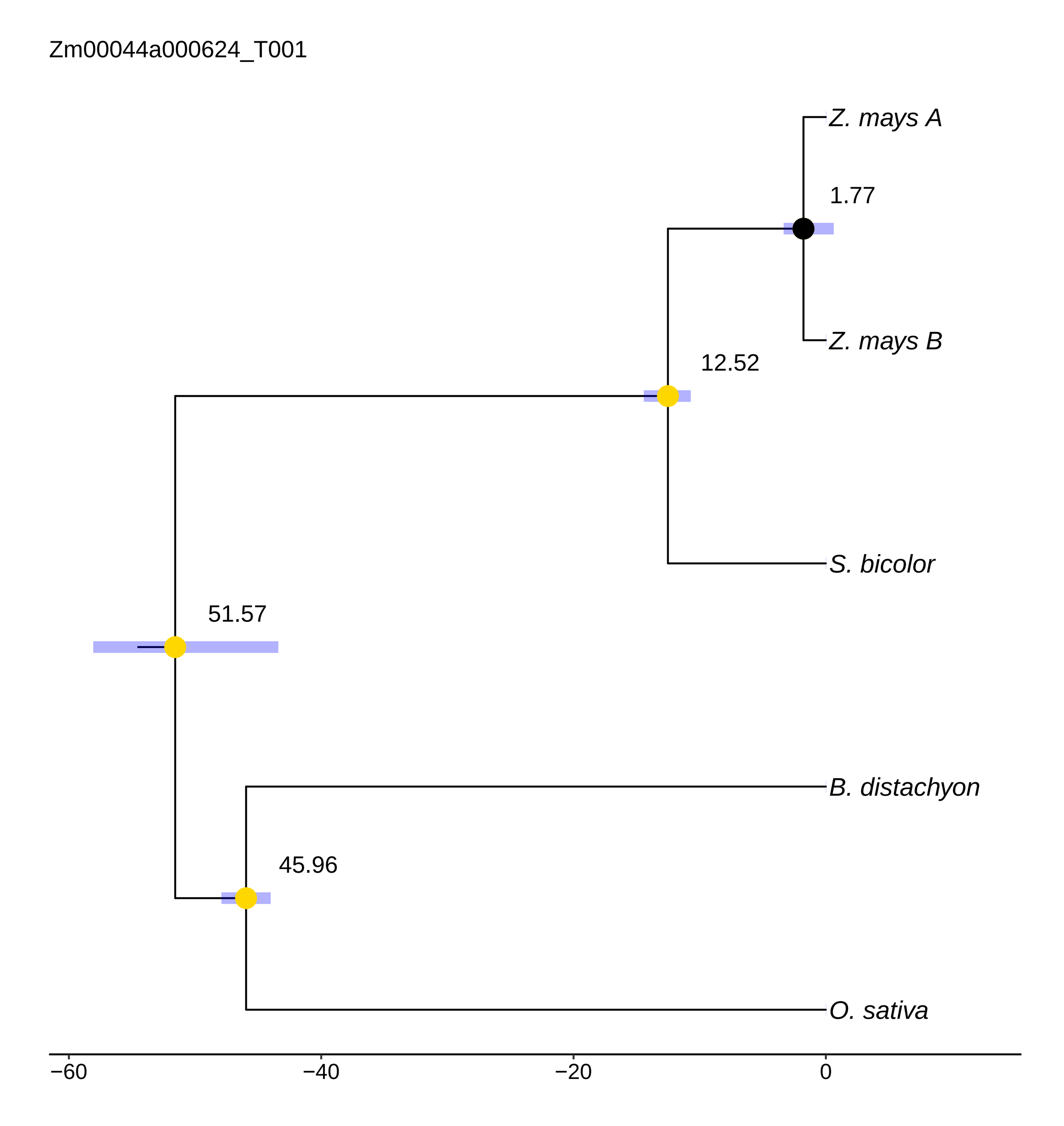
Zm00044a000624_T001
Z. mays A
1.77
Z. mays B
12.52
S. bicolor
51.57
B. distachyon
45.96
O. sativa
−60
−40
−20
0

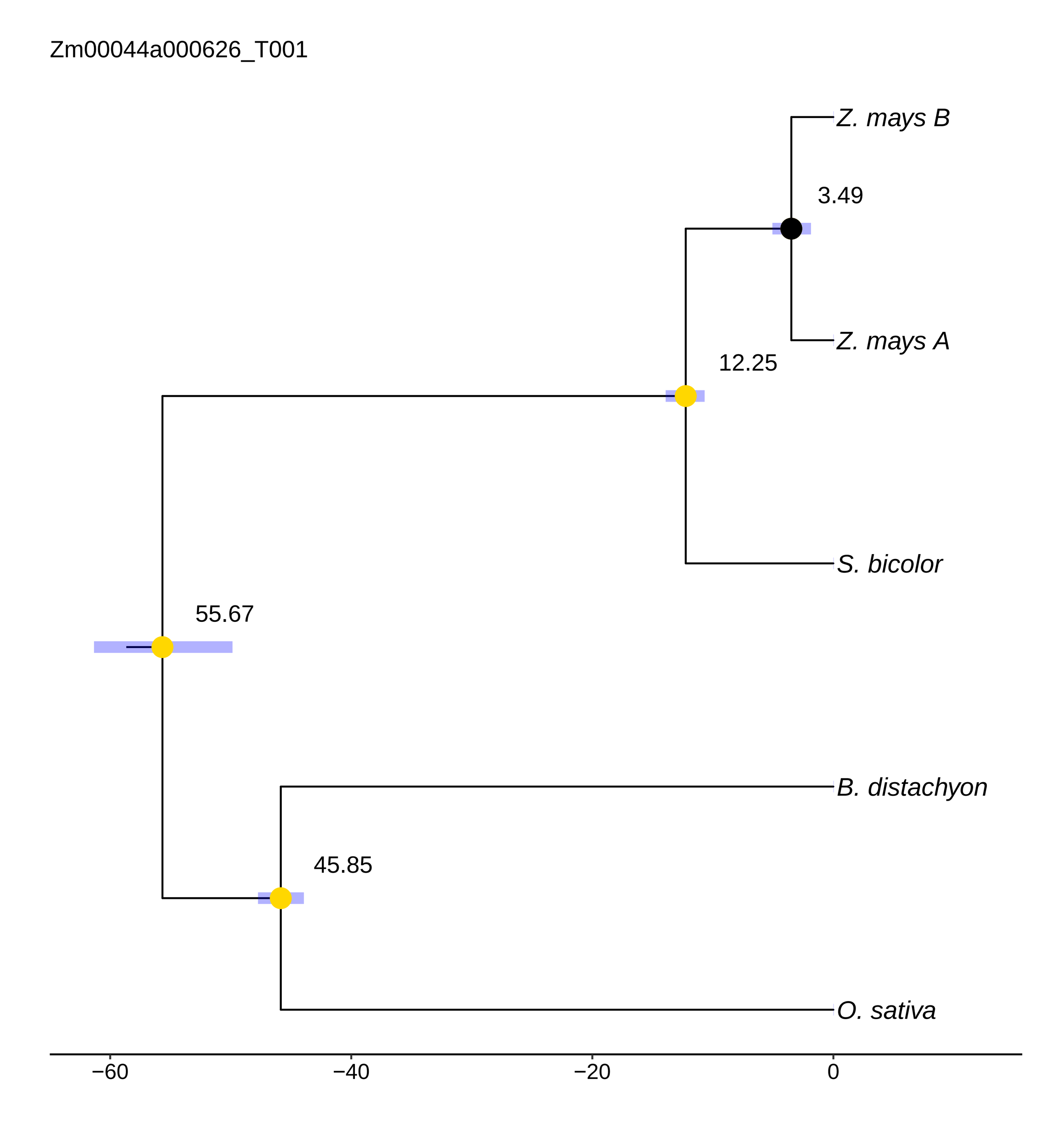
Zm00044a000626_T001
Z. mays B
3.49
Z. mays A
12.25
S. bicolor
55.67
B. distachyon
45.85
O. sativa
−60
−40
−20
0

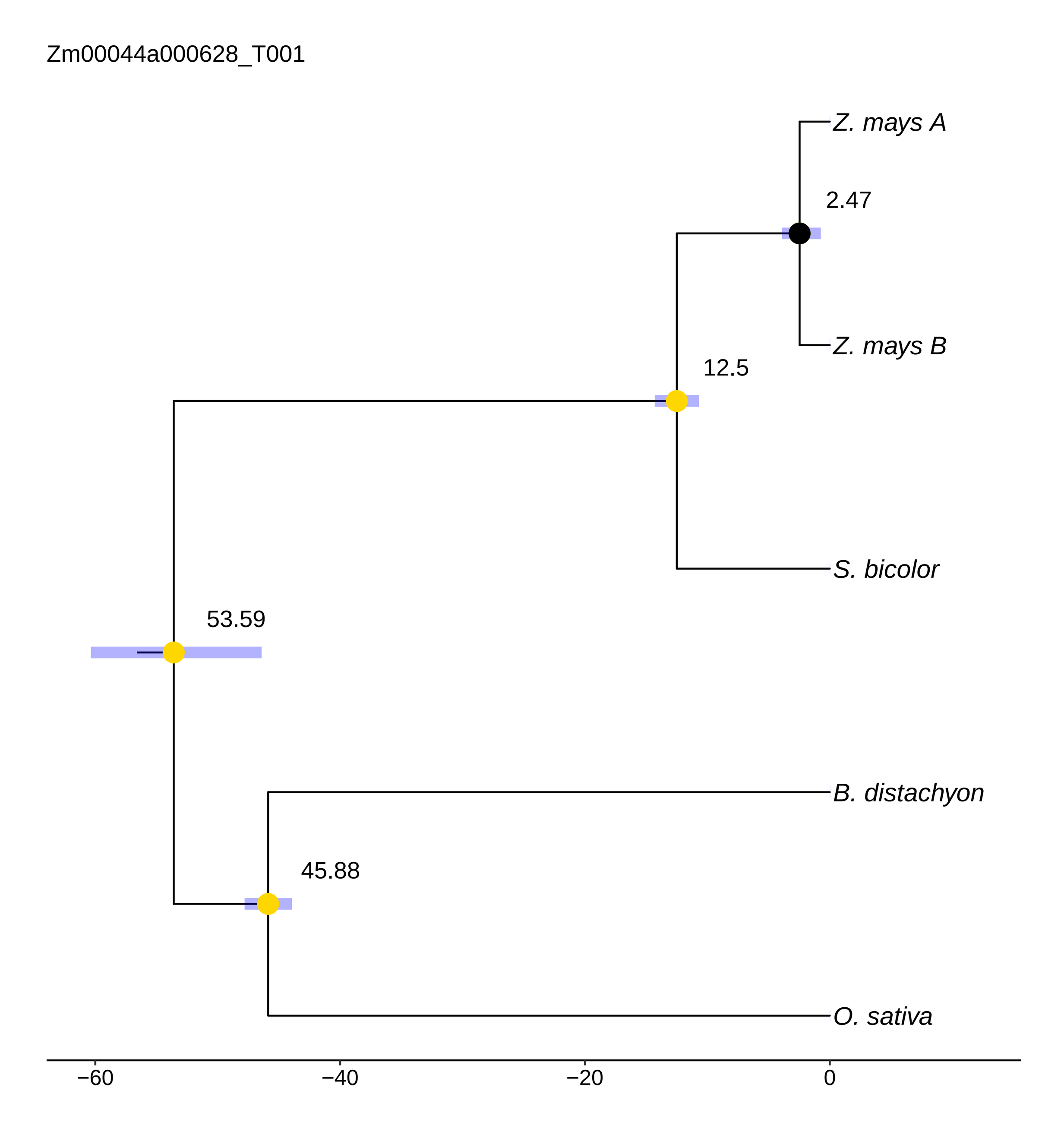
Zm00044a000628_T001
Z. mays A
2.47
Z. mays B
12.5
S. bicolor
53.59
B. distachyon
45.88
O. sativa
−60
−40
−20
0

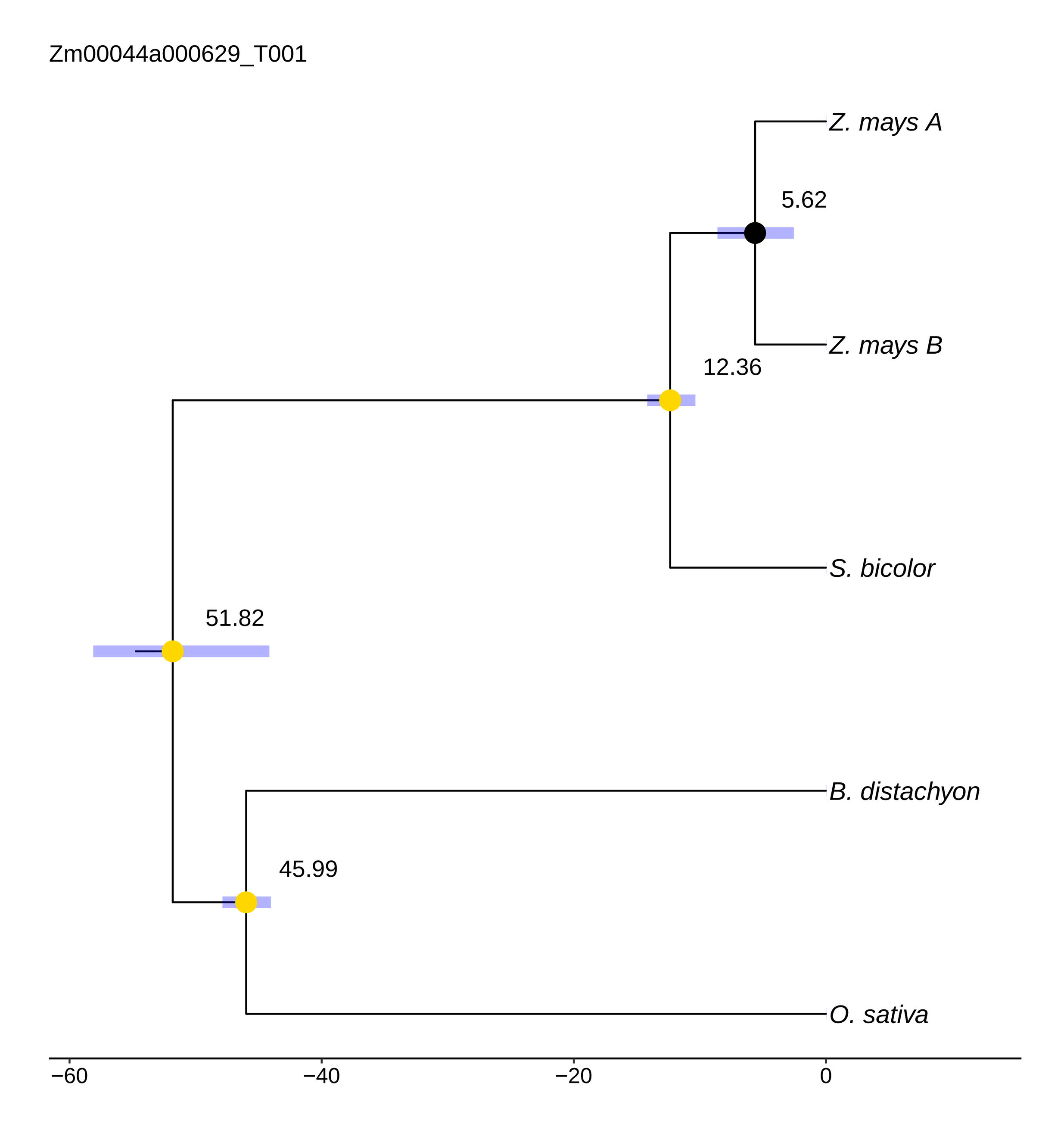
Zm00044a000629_T001
Z. mays A
5.62
Z. mays B
12.36
S. bicolor
51.82
B. distachyon
45.99
O. sativa
−60
−40
−20
0

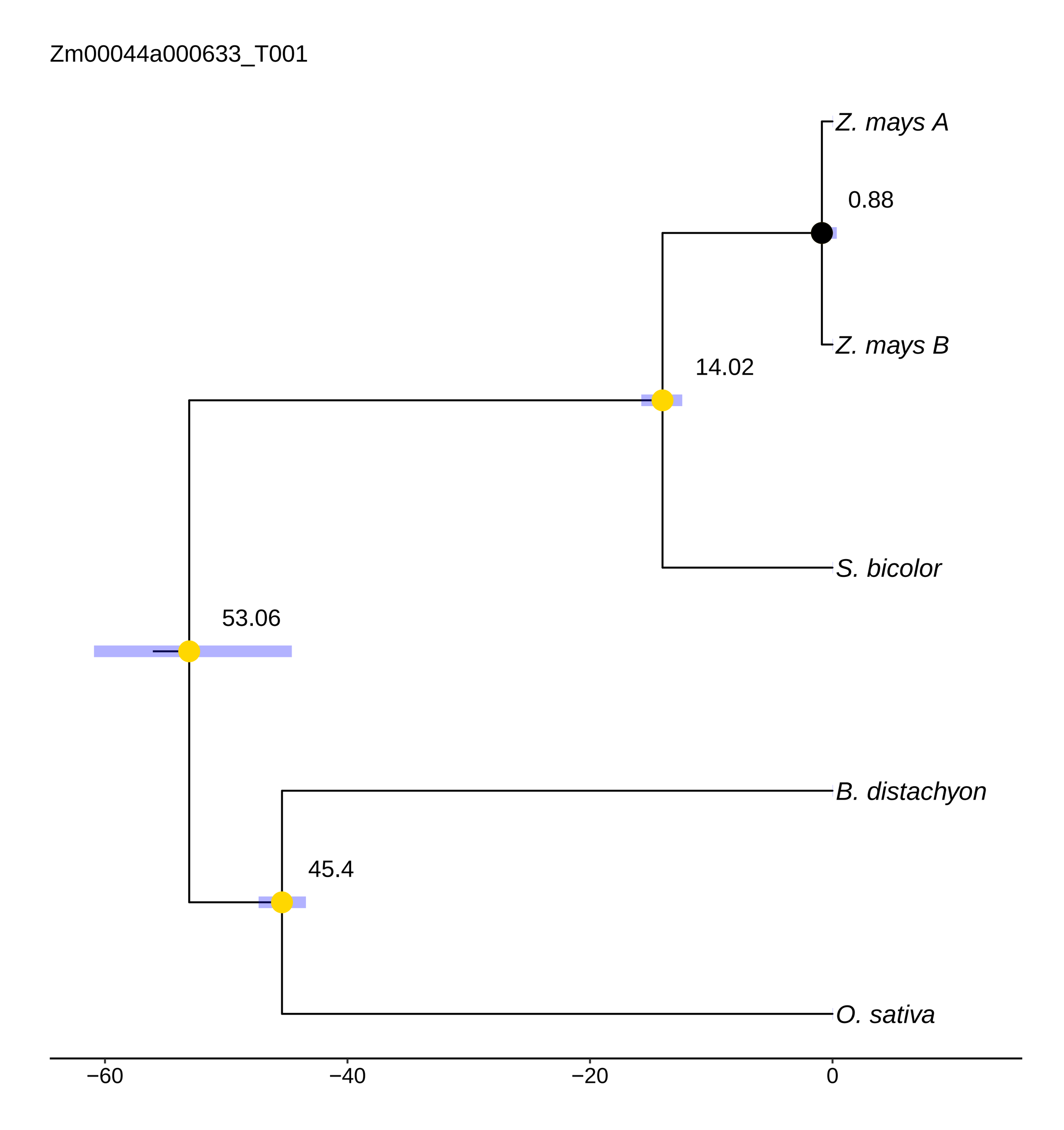
Zm00044a000633_T001
Z. mays A
0.88
Z. mays B
14.02
S. bicolor
53.06
B. distachyon
45.4
O. sativa
−60
−40
−20
0

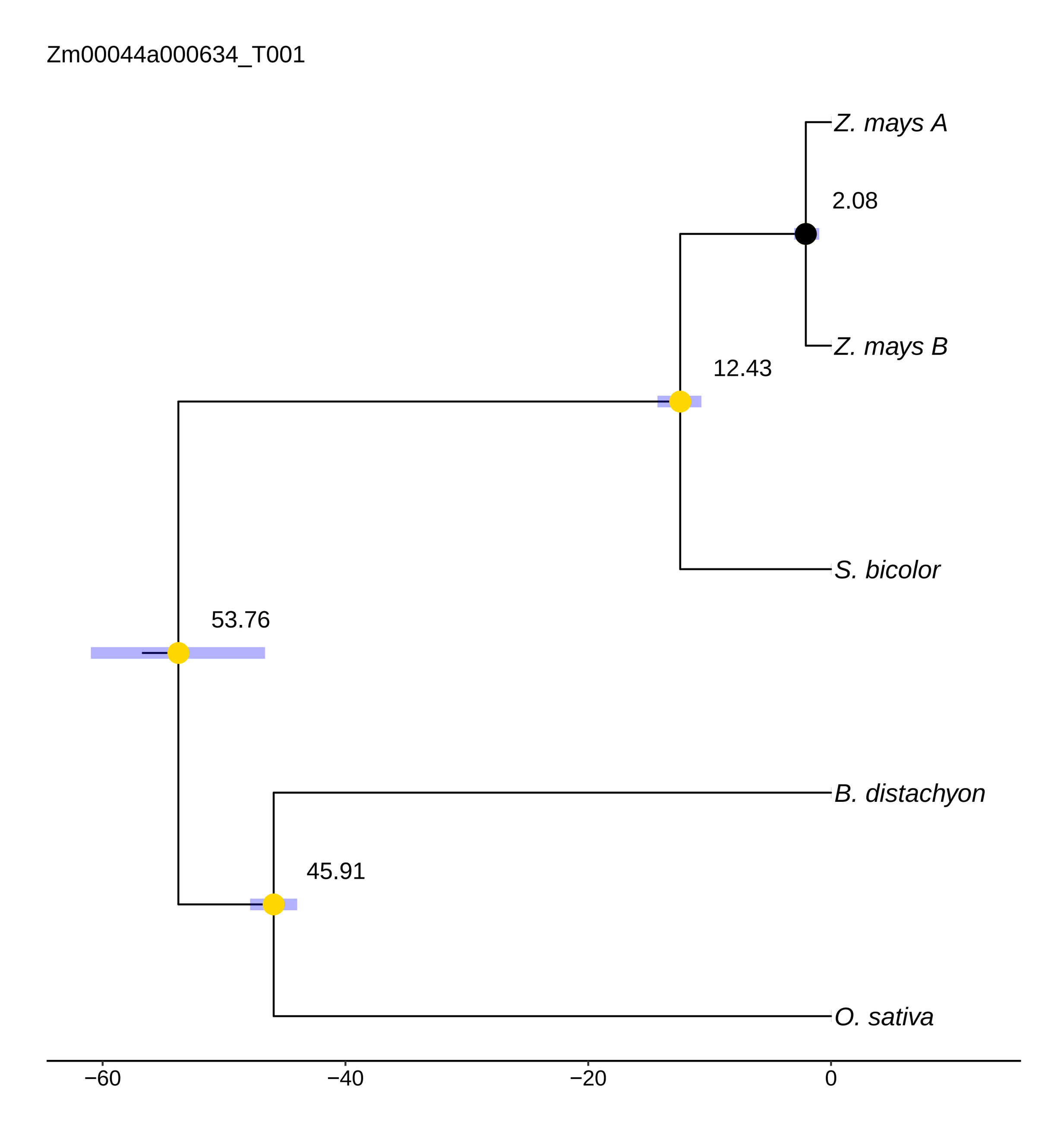
Zm00044a000634_T001
Z. mays A
2.08
Z. mays B
12.43
S. bicolor
53.76
B. distachyon
45.91
O. sativa
−60
−40
−20
0

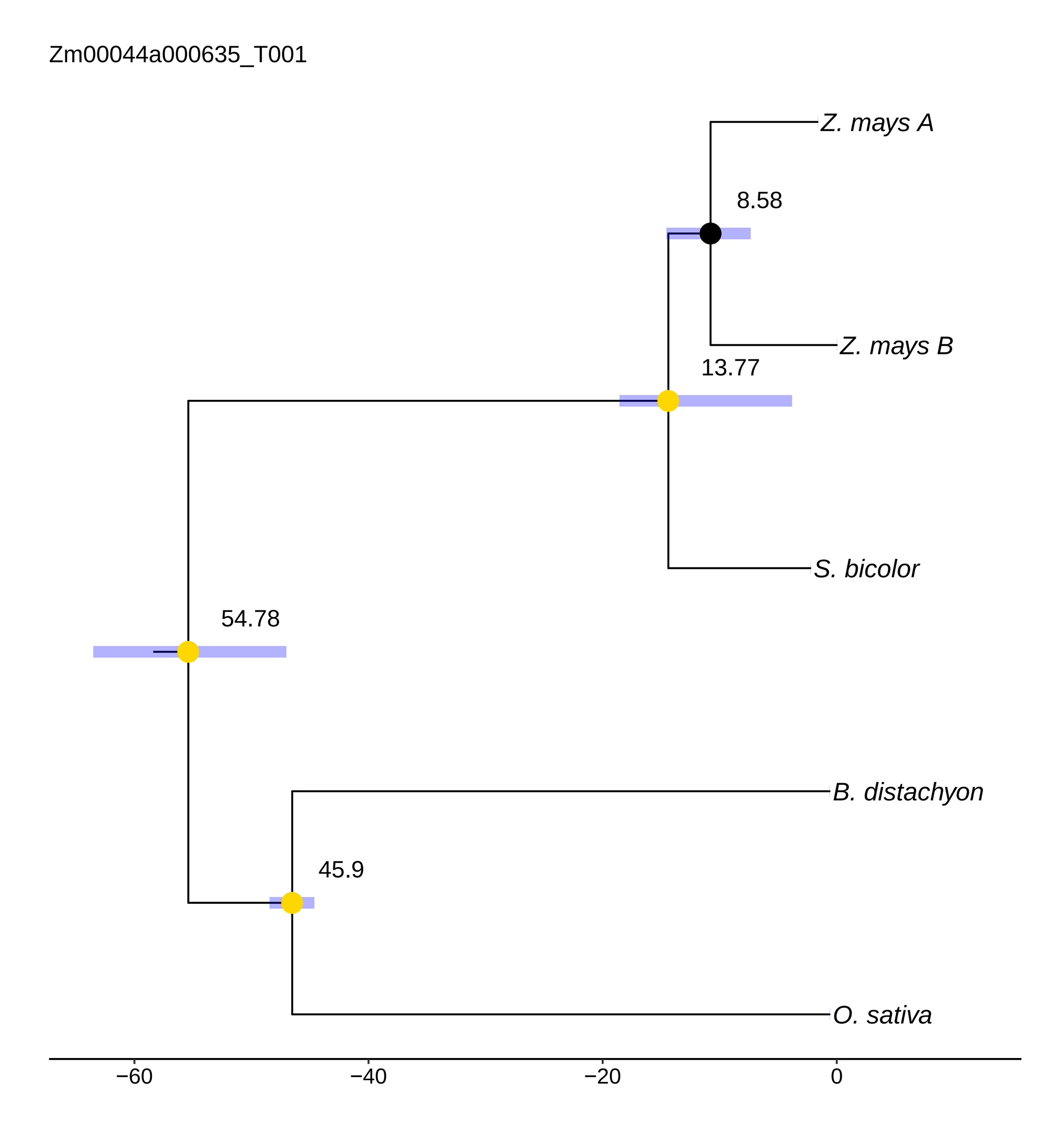
Zm00044a000635_T001
Z. mays A
8.58
Z. mays B
13.77
S. bicolor
54.78
B. distachyon
45.9
O. sativa
−60
−40
−20
0

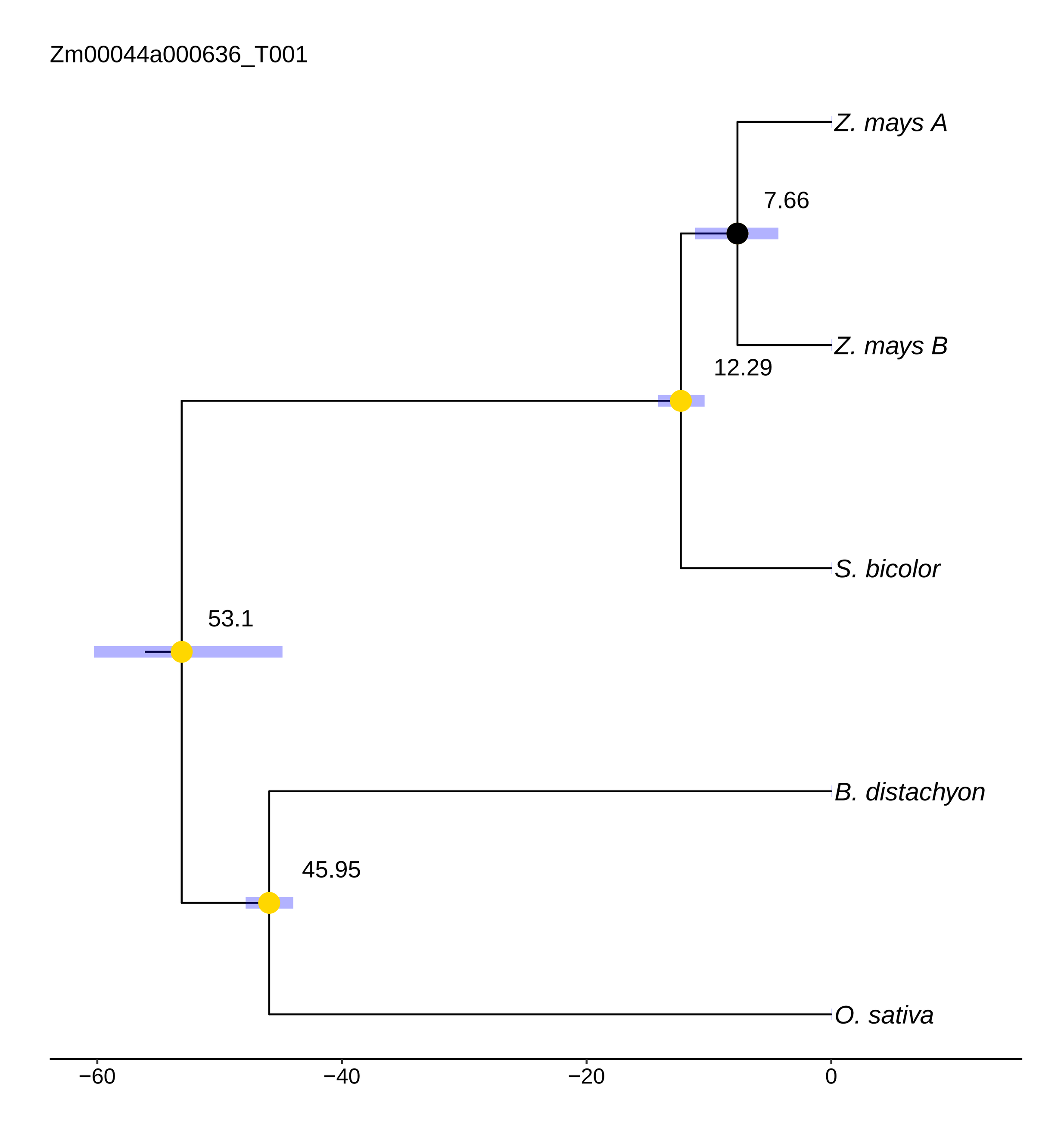
Zm00044a000636_T001
Z. mays A
7.66
Z. mays B
12.29
S. bicolor
53.1
B. distachyon
45.95
O. sativa
−60
−40
−20
0

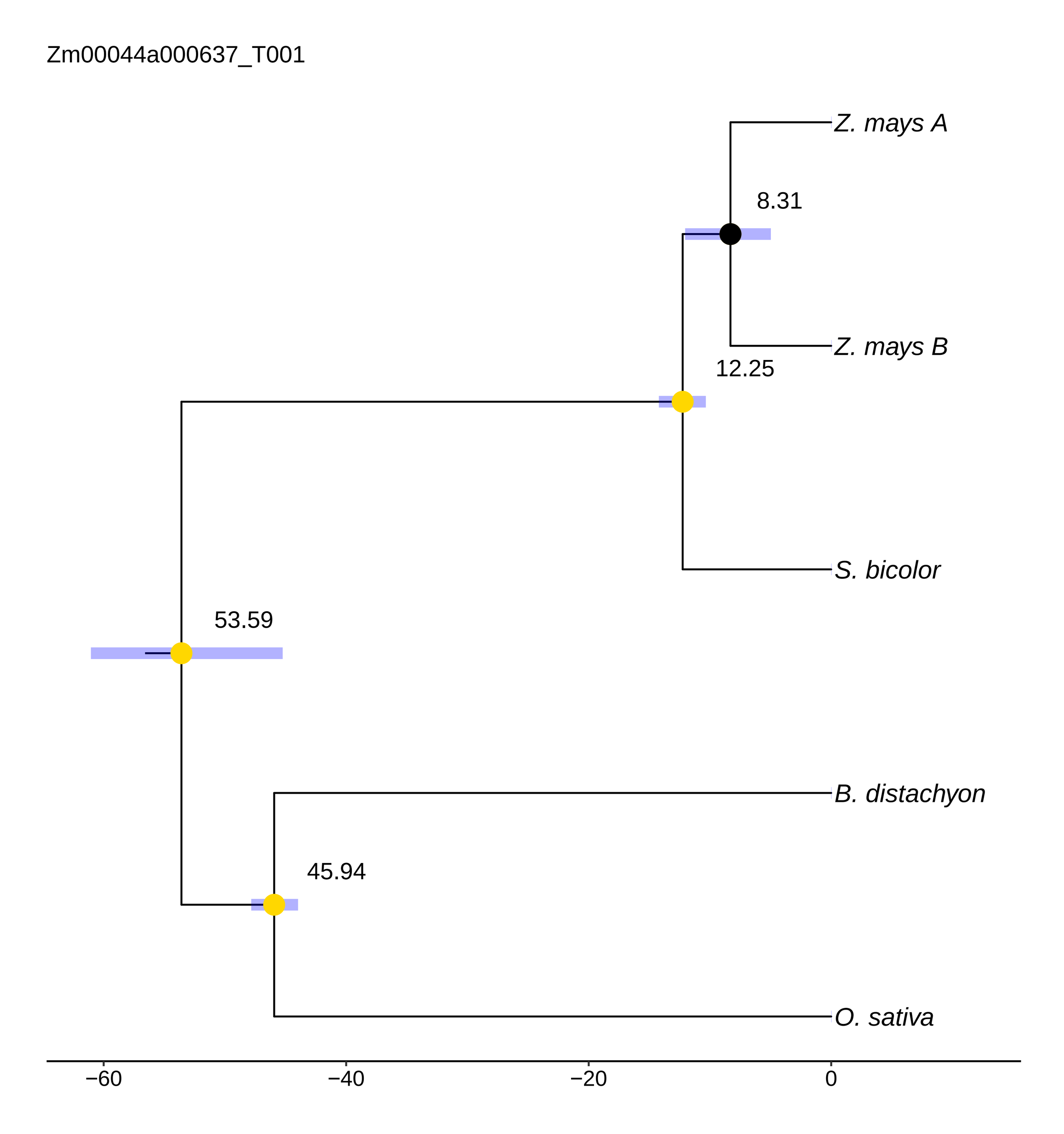
Zm00044a000637_T001
Z. mays A
8.31
Z. mays B
12.25
S. bicolor
53.59
B. distachyon
45.94
O. sativa
−60
−40
−20
0

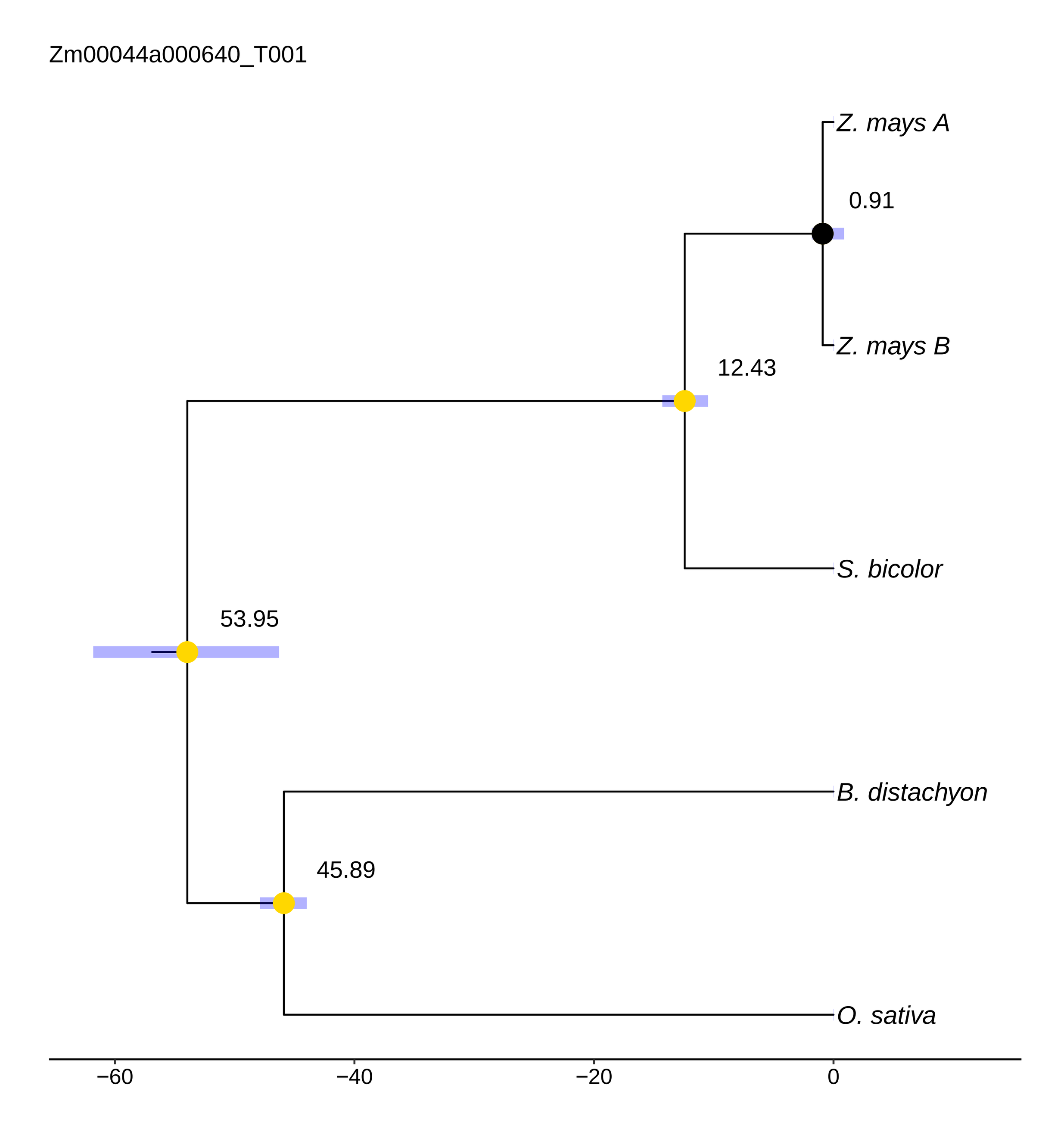
Zm00044a000640_T001
Z. mays A
0.91
Z. mays B
12.43
S. bicolor
53.95
B. distachyon
45.89
O. sativa
−60
−40
−20
0

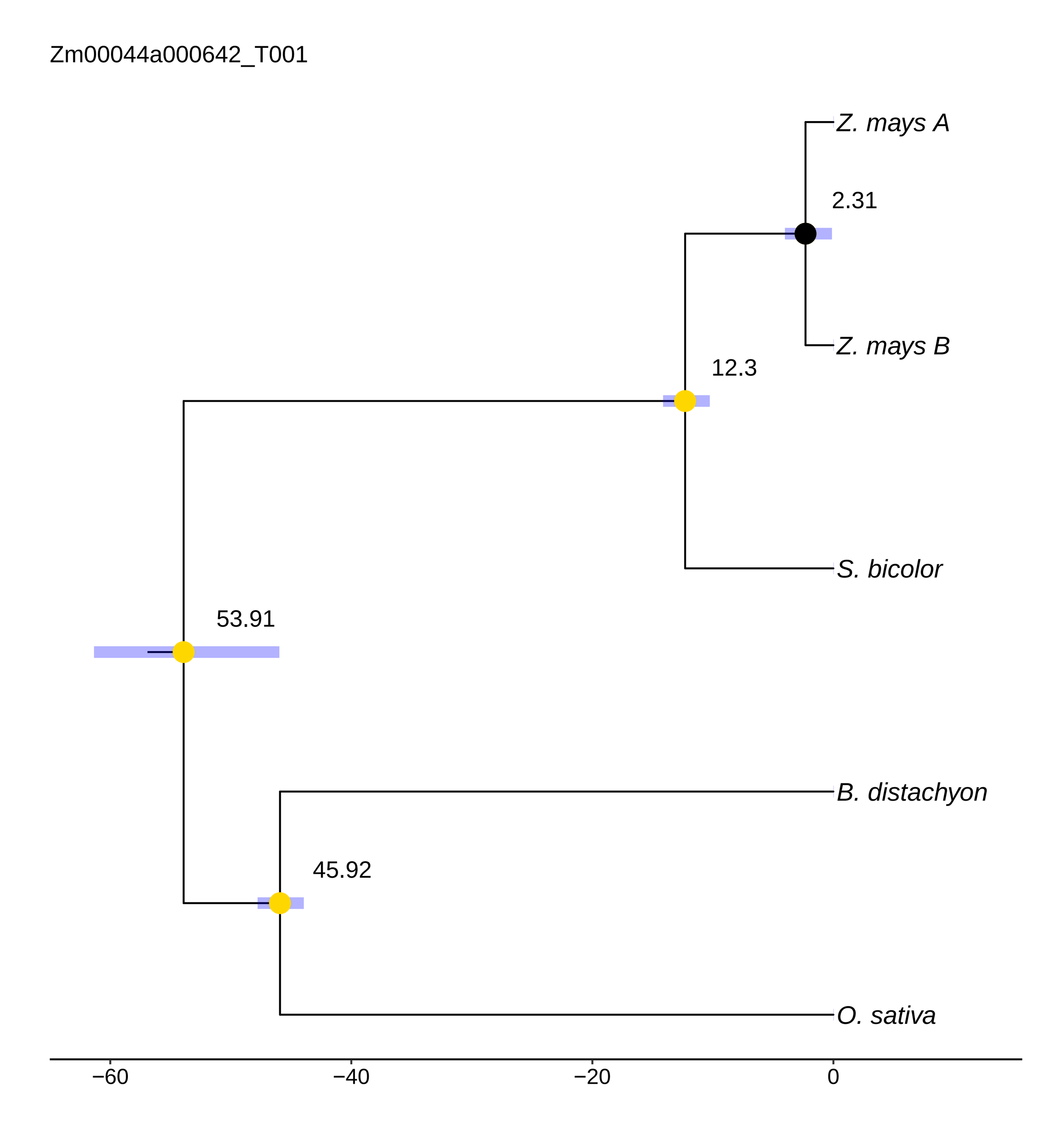
Zm00044a000642_T001
Z. mays A
2.31
Z. mays B
12.3
S. bicolor
53.91
B. distachyon
45.92
O. sativa
−60
−40
−20
0

Zm00044a000646_T001

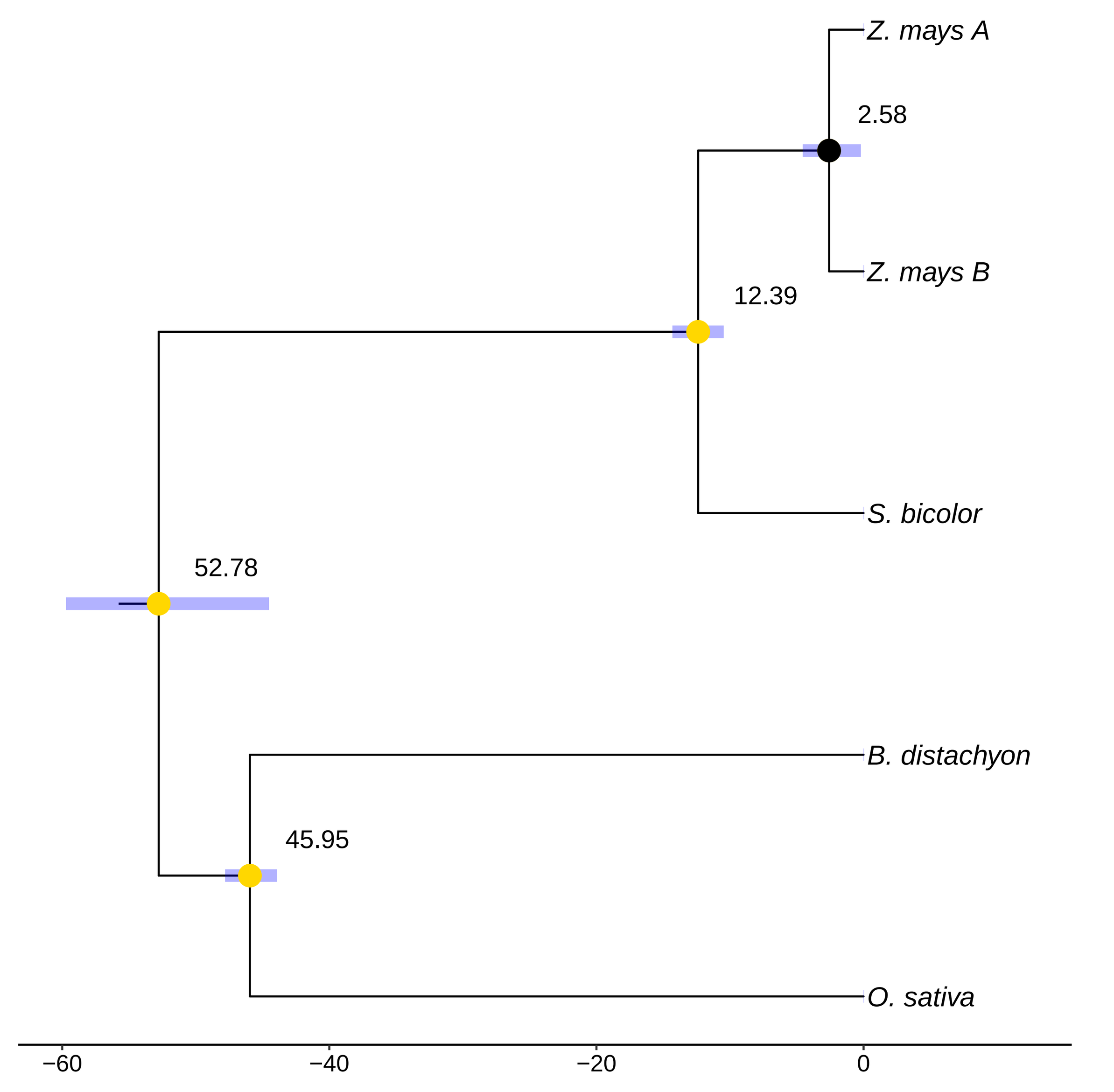

Zm00044a000648_T001

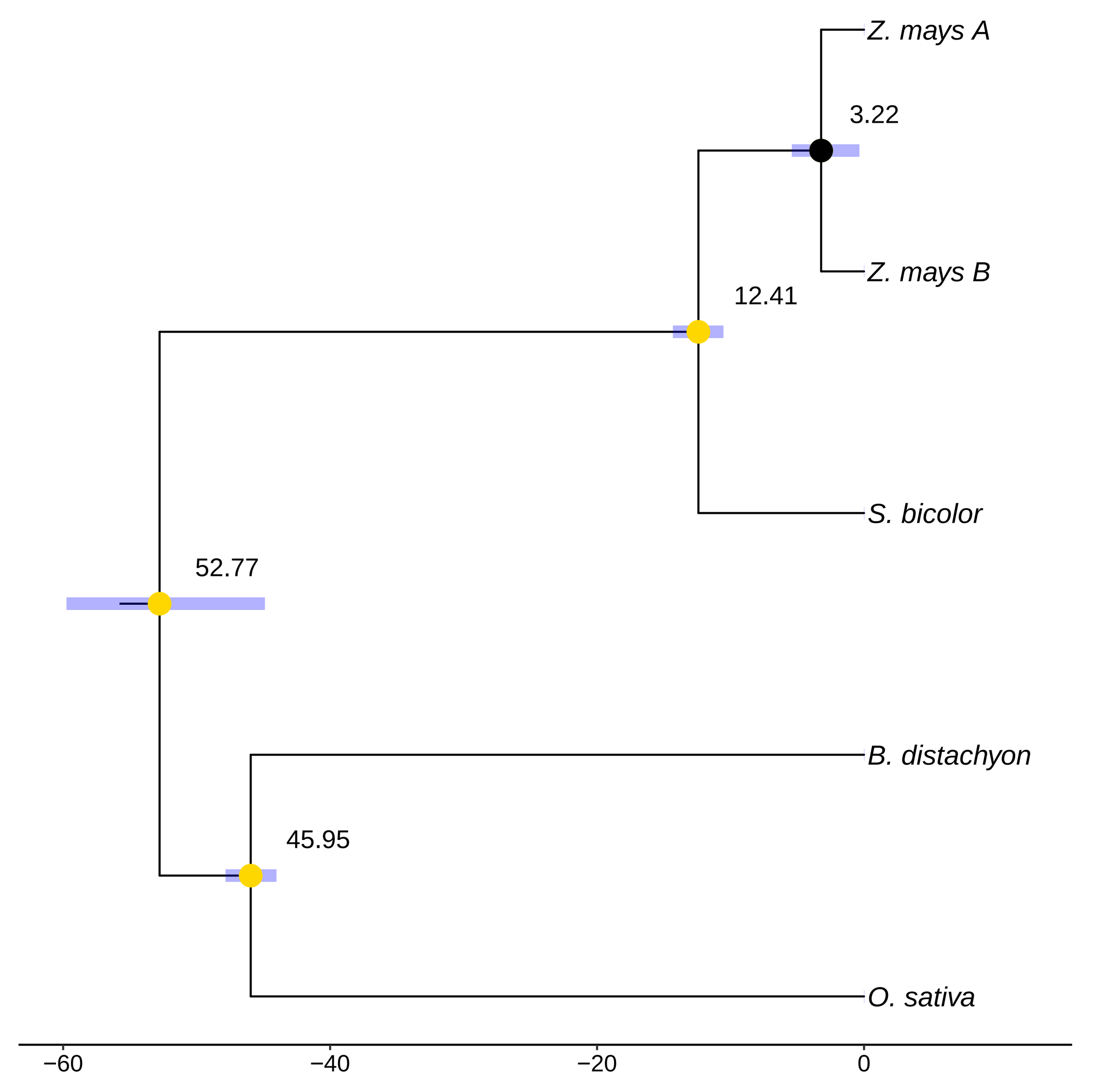

Zm00044a000650_T001

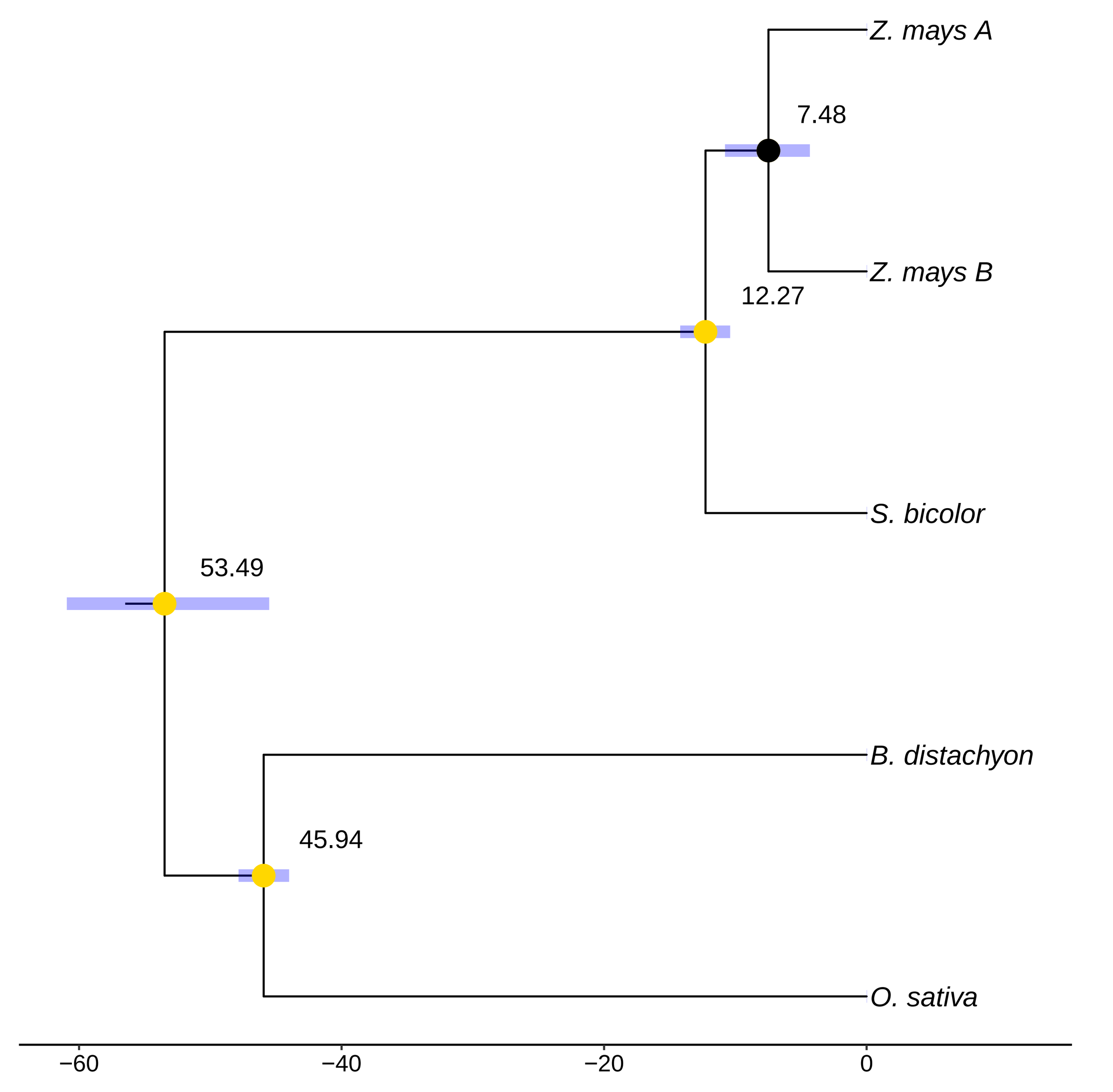

Zm00044a000652_T001

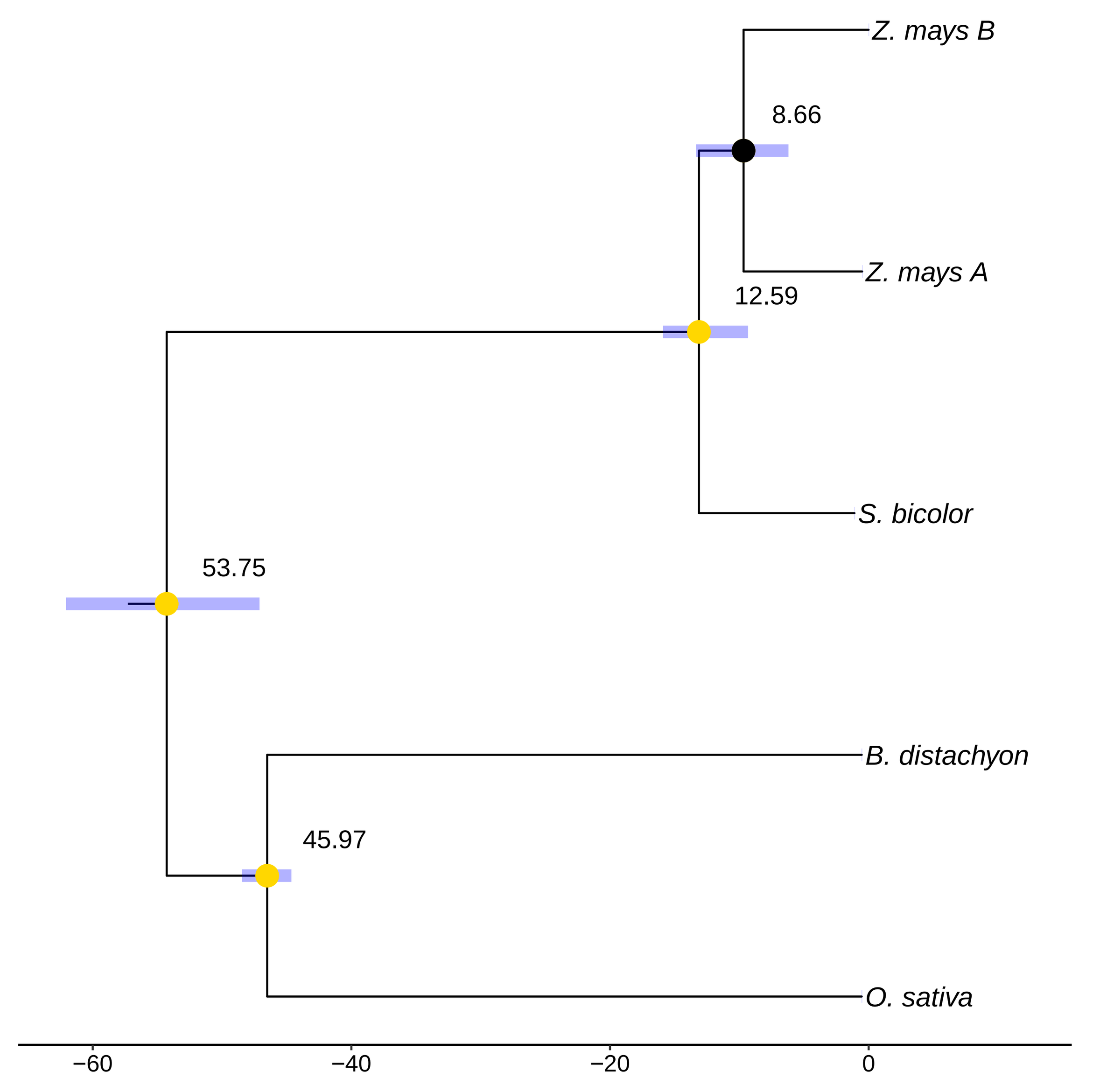

Zm00044a000657_T001

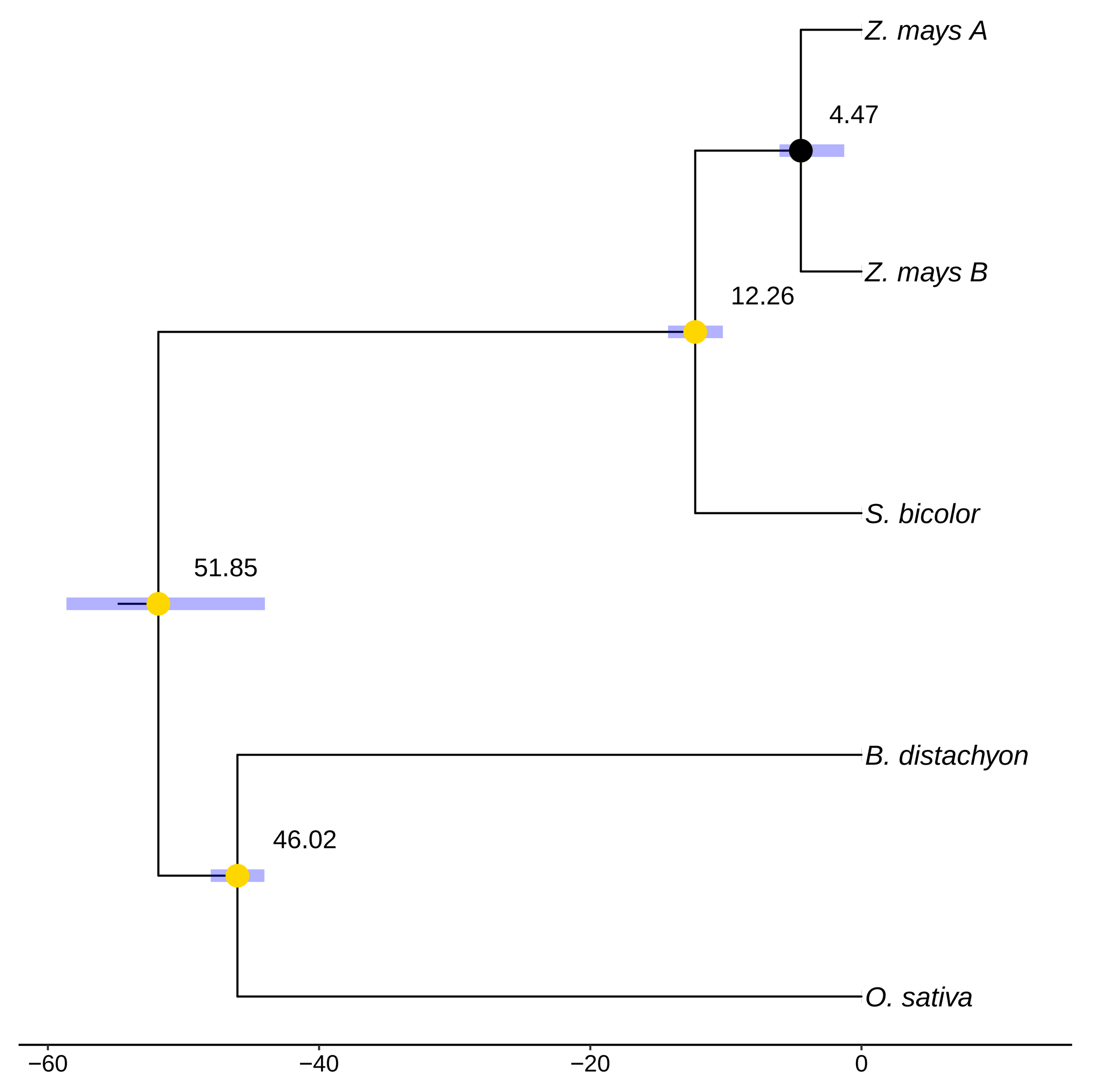

Zm00044a000658_T001

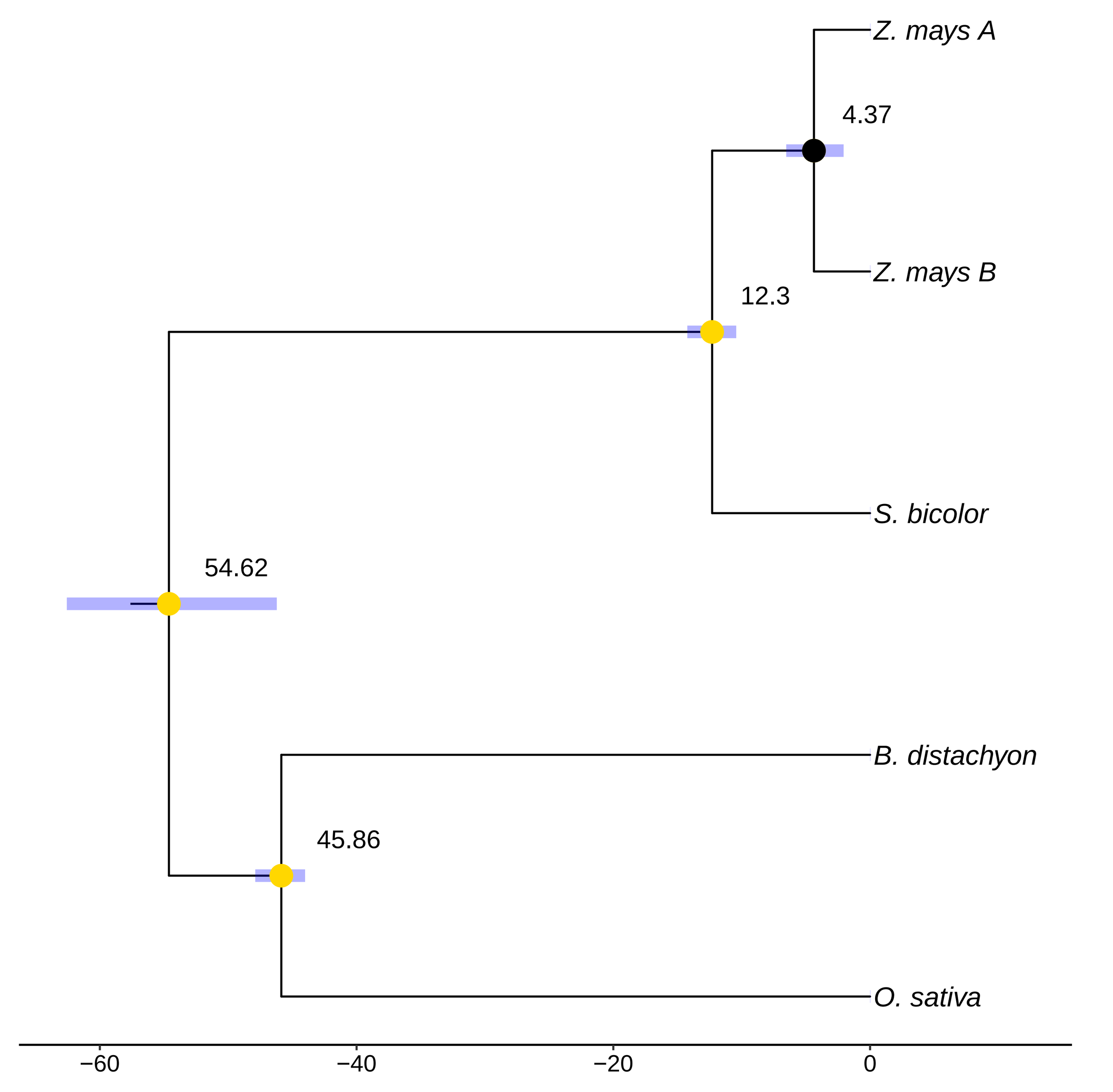

Zm00044a000659_T001

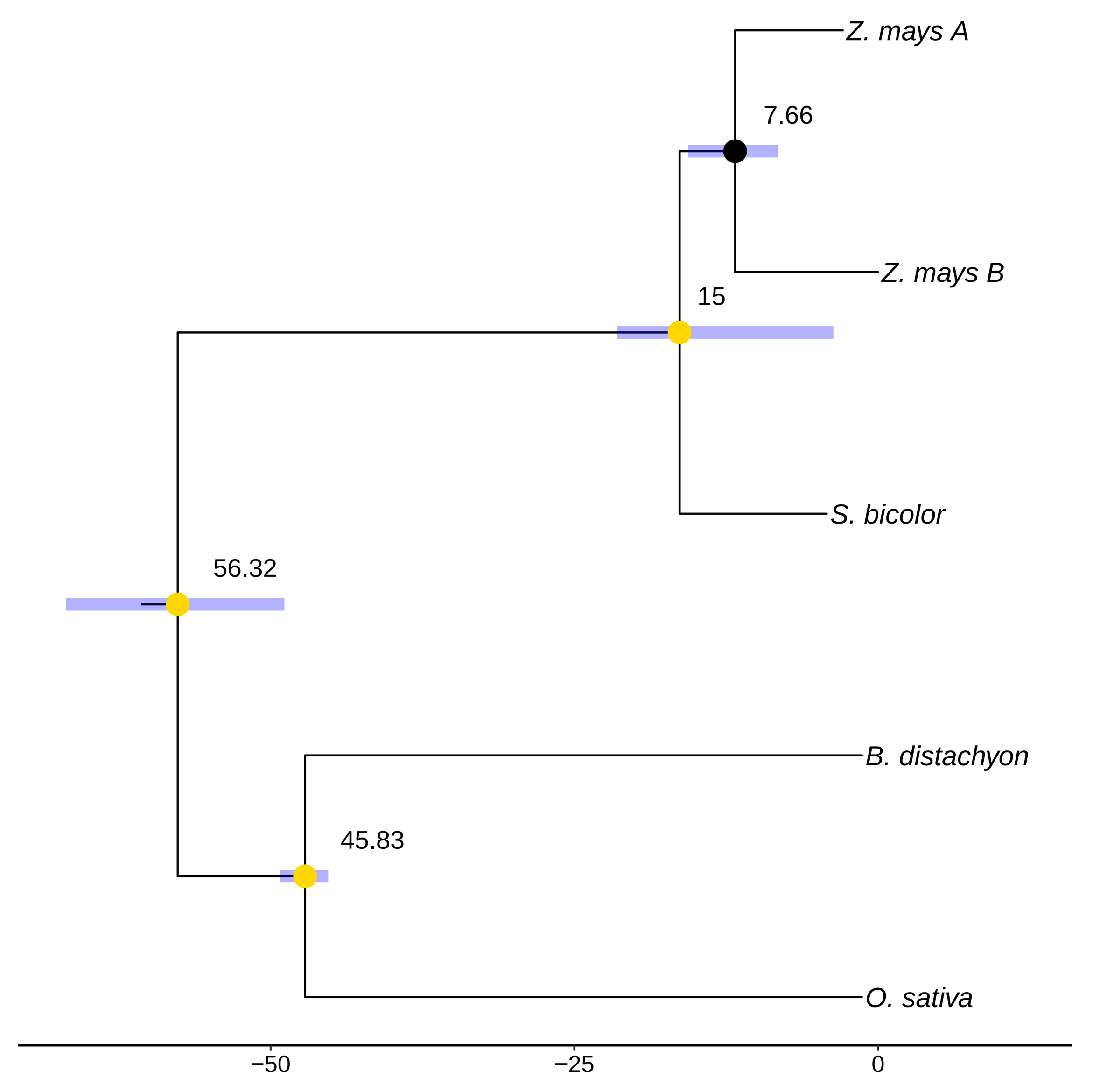

Zm00044a000674_T001

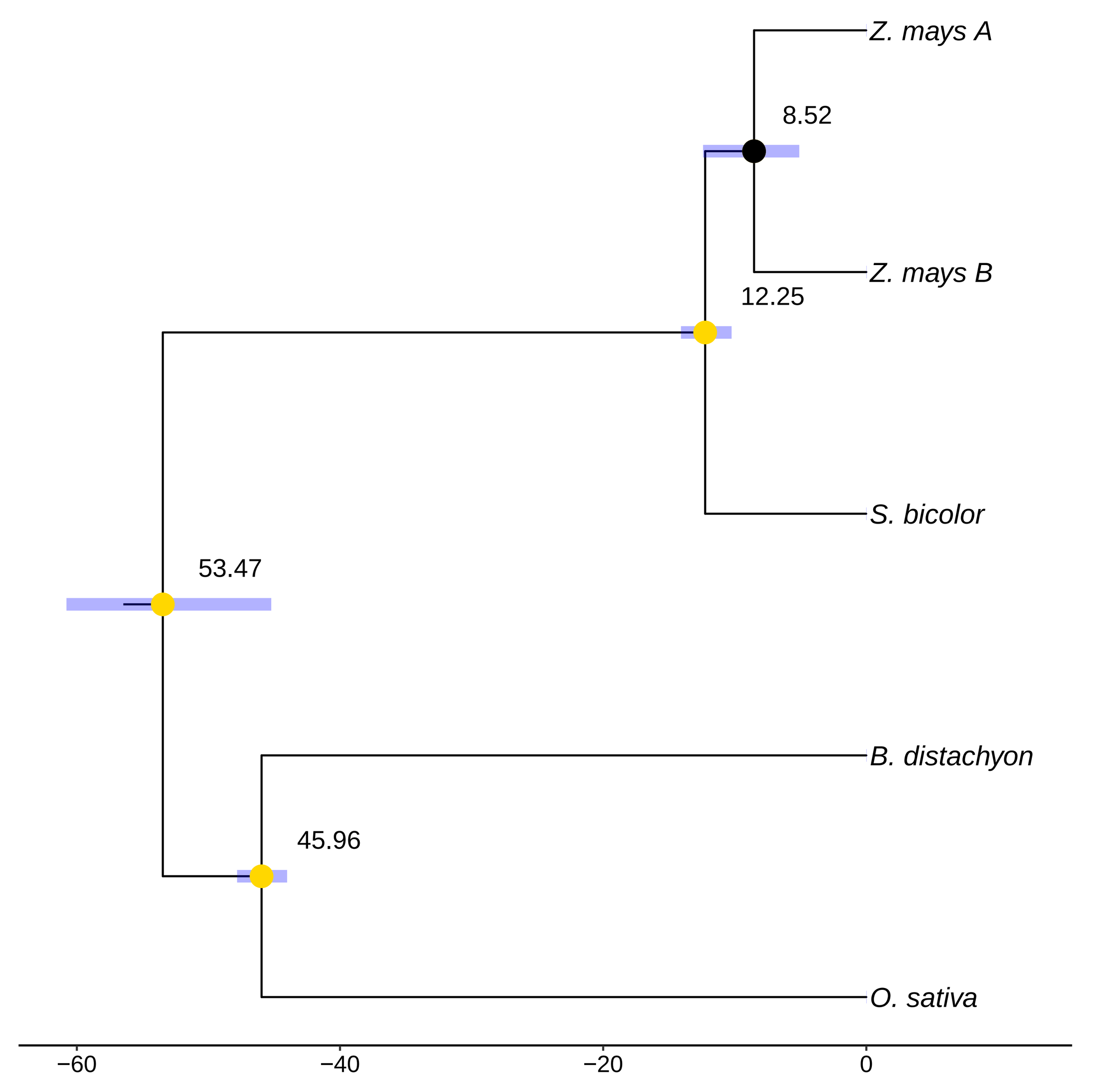

Zm00044a000679_T001

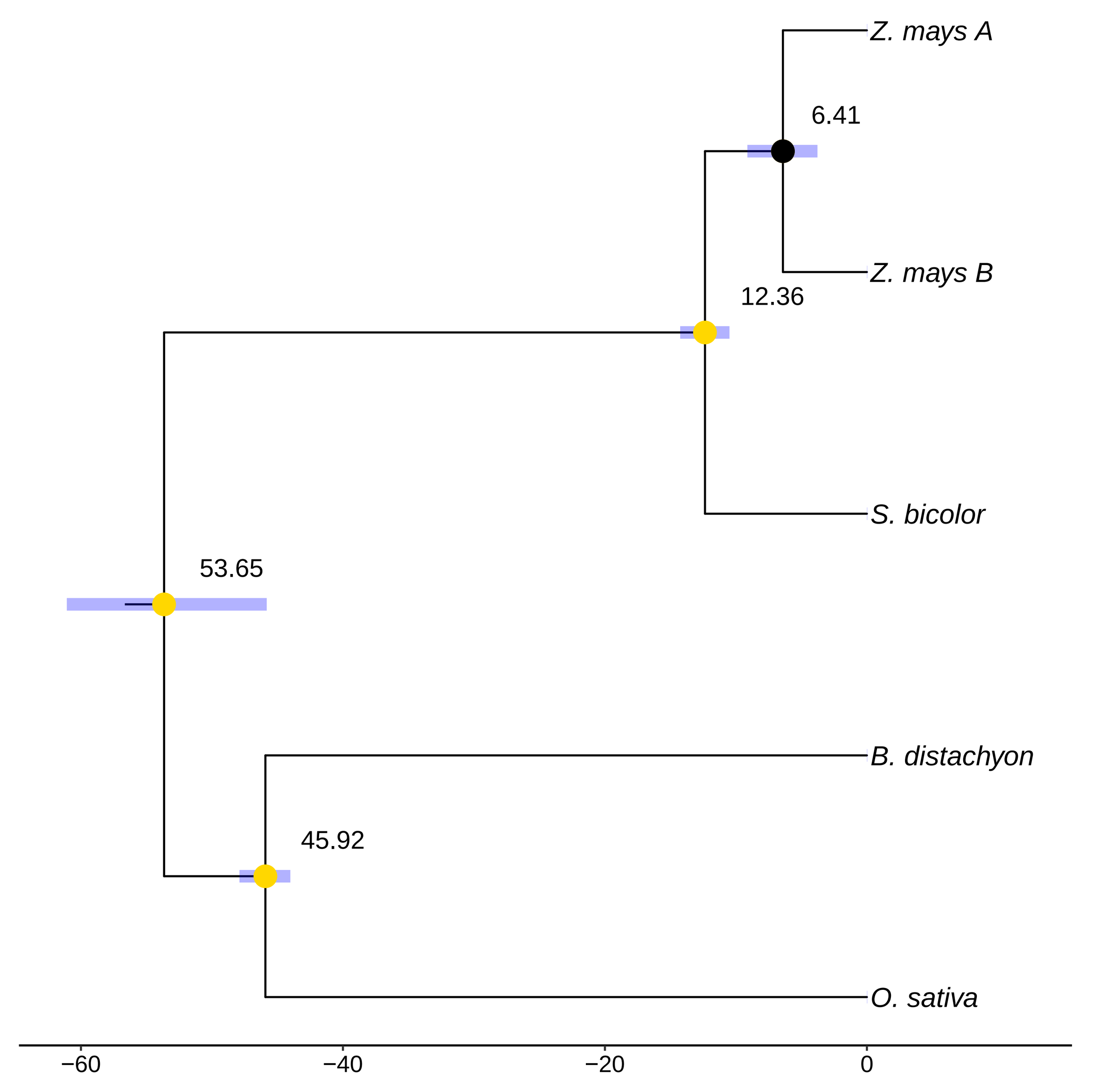

Zm00044a000680_T001

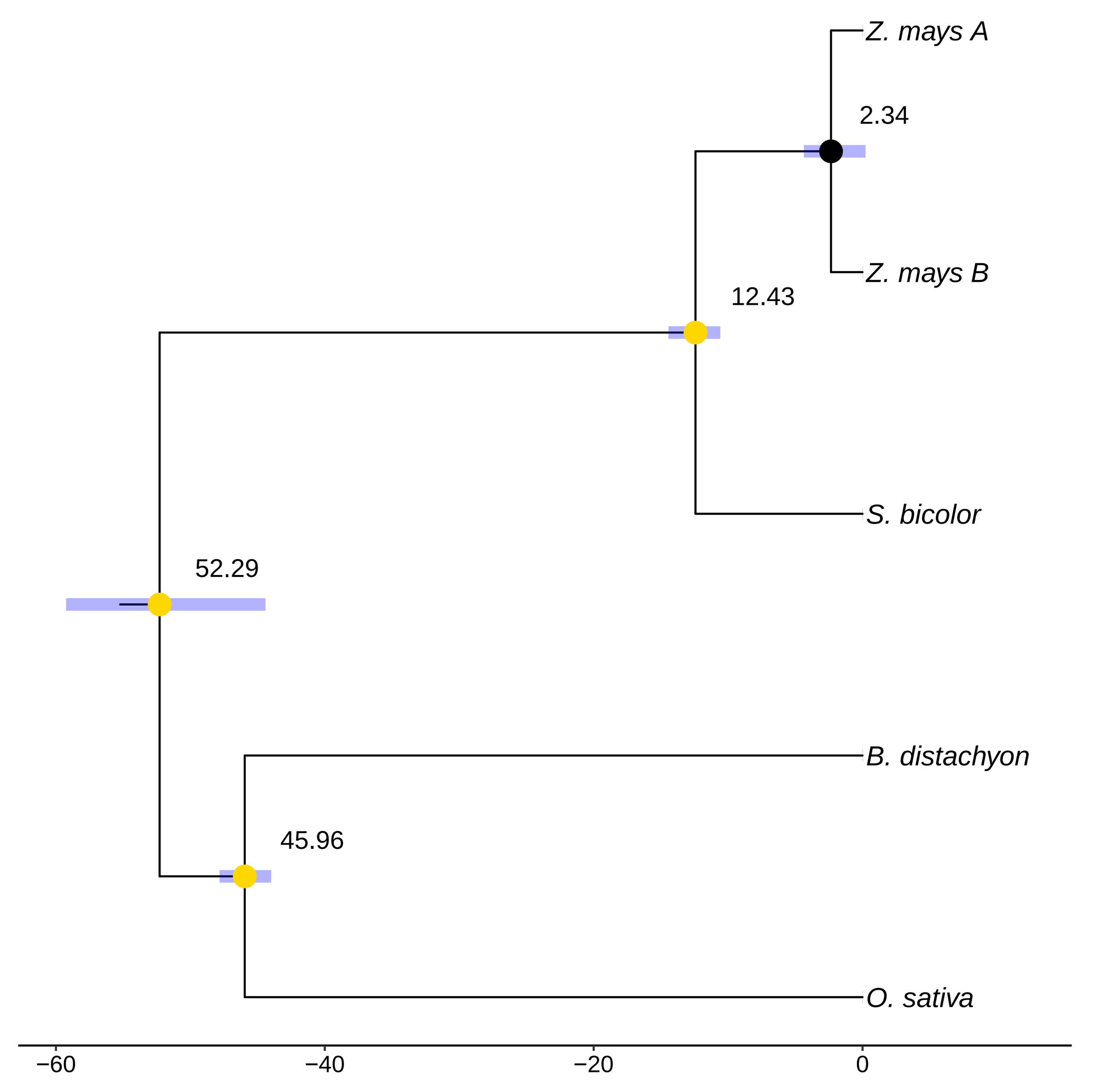

Zm00044a000681_T001

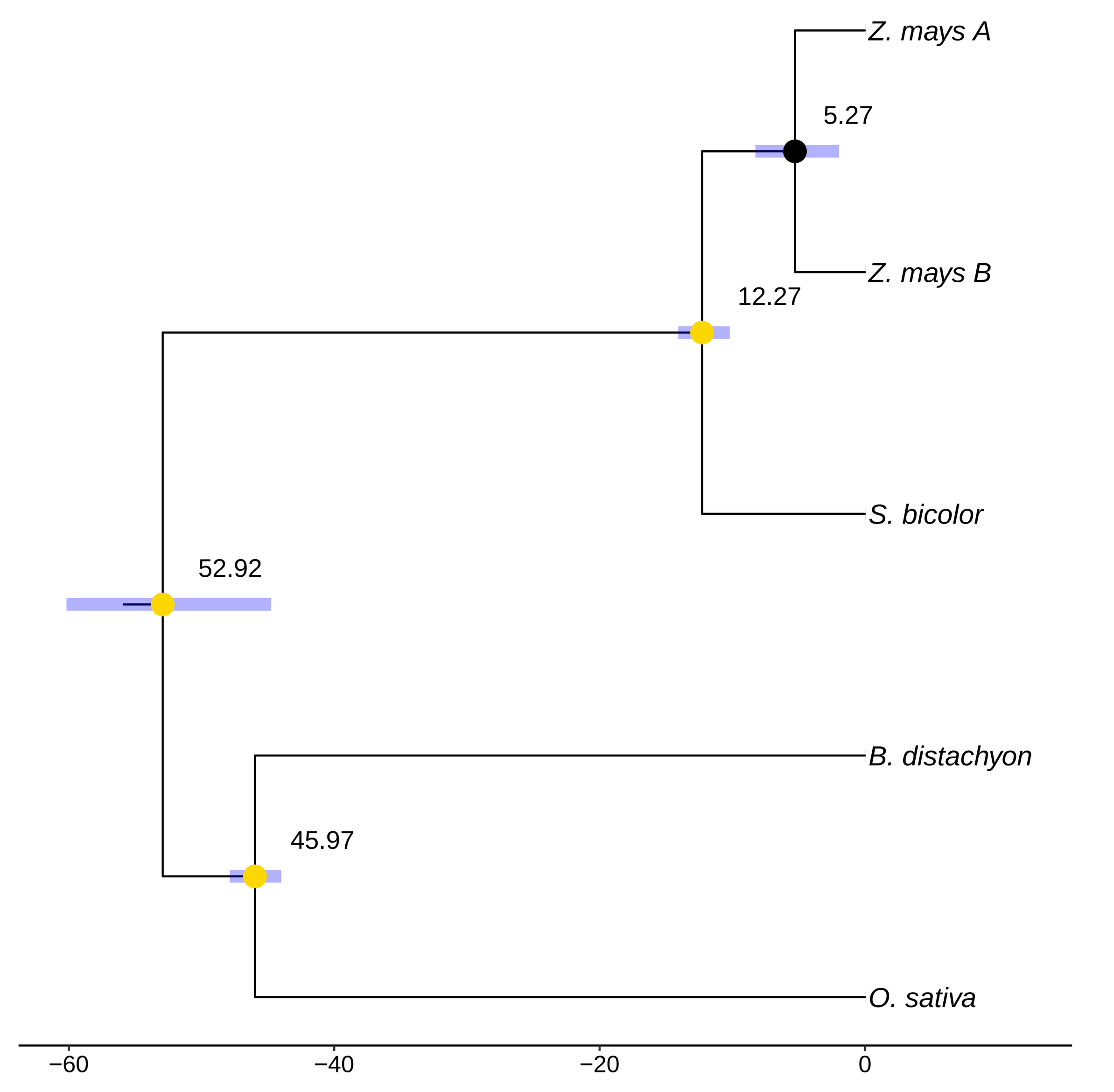

Zm00044a000682_T001

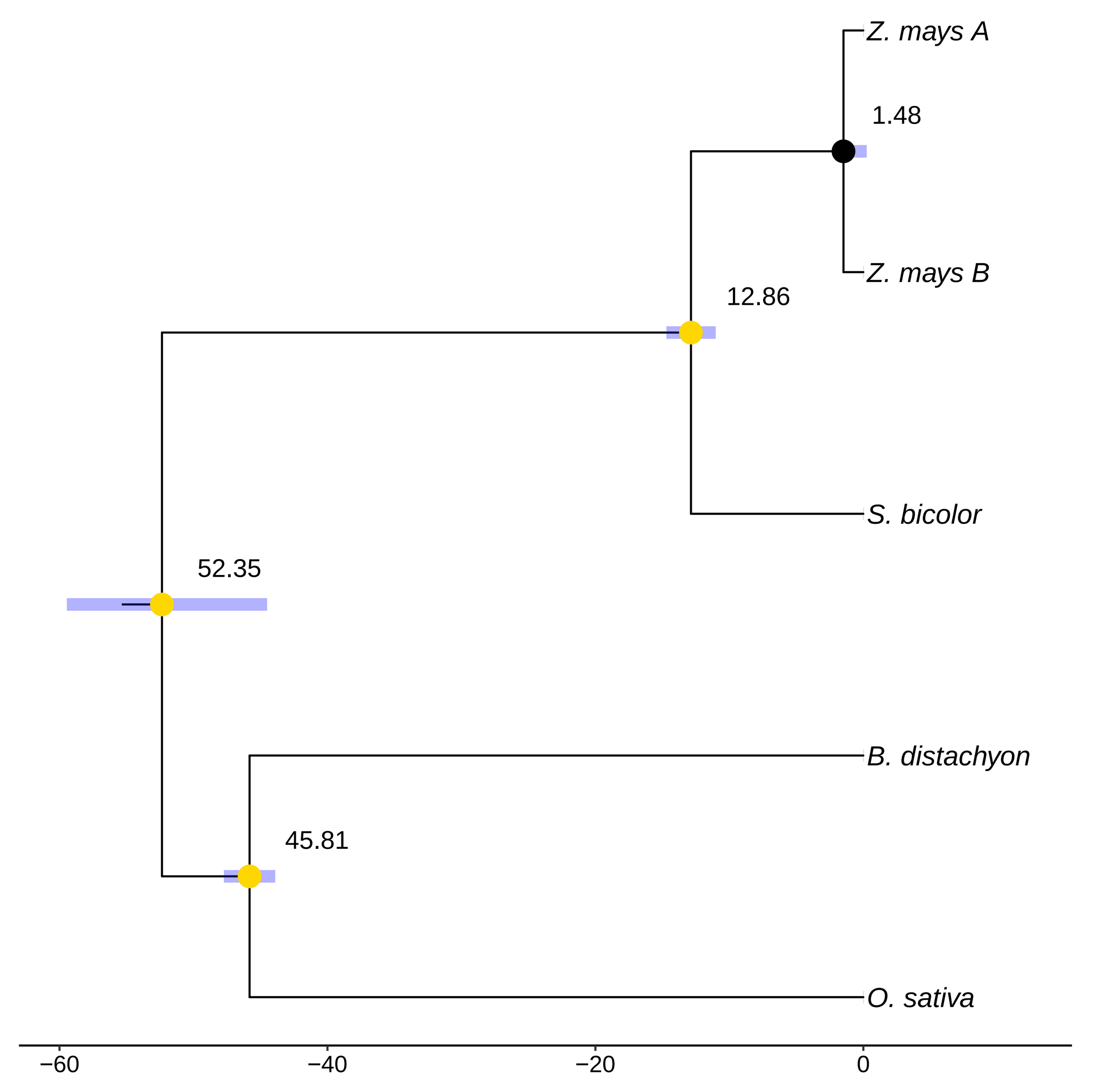

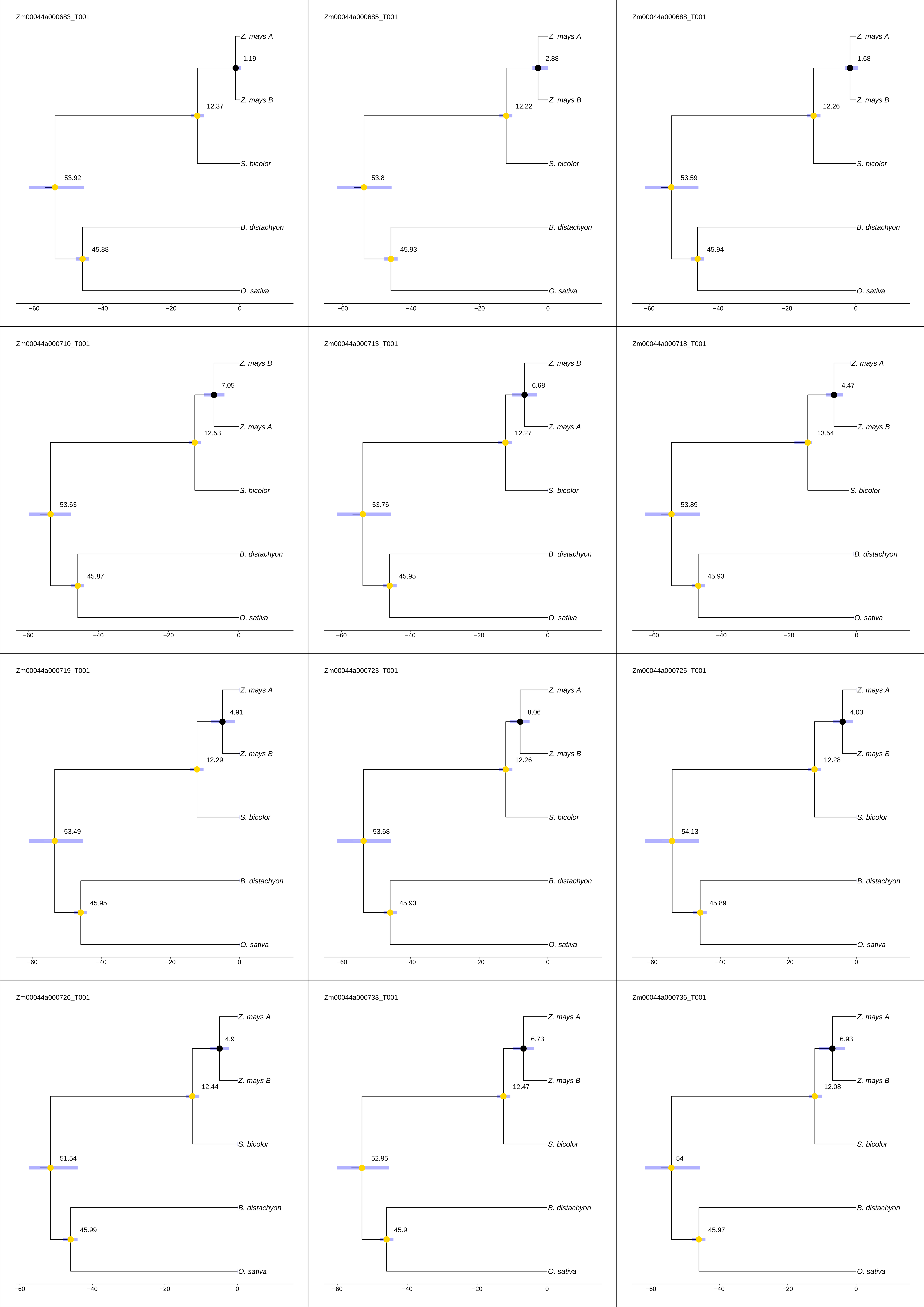
Zm00044a000683_T001
Z. mays A
1.19
Z. mays B
12.37
S. bicolor
53.92
B. distachyon
45.88
O. sativa
−60
−40
−20
0
Zm00044a000685_T001
Z. mays A
2.88
Z. mays B
12.22
S. bicolor
53.8
B. distachyon
45.93
O. sativa
−60
−40
−20
0
Zm00044a000688_T001
Z. mays A
1.68
Z. mays B
12.26
S. bicolor
53.59
B. distachyon
45.94
O. sativa
−60
−40
−20
0
Zm00044a000710_T001
Z. mays B
7.05
Z. mays A
12.53
S. bicolor
53.63
B. distachyon
45.87
O. sativa
−60
−40
−20
0
Zm00044a000713_T001
Z. mays B
6.68
Z. mays A
12.27
S. bicolor
53.76
B. distachyon
45.95
O. sativa
−60
−40
−20
0
Zm00044a000718_T001
Z. mays A
4.47
Z. mays B
13.54
S. bicolor
53.89
B. distachyon
45.93
O. sativa
−60
−40
−20
0
Zm00044a000719_T001
Z. mays A
4.91
Z. mays B
12.29
S. bicolor
53.49
B. distachyon
45.95
O. sativa
−60
−40
−20
0
Zm00044a000723_T001
Z. mays A
8.06
Z. mays B
12.26
S. bicolor
53.68
B. distachyon
45.93
O. sativa
−60
−40
−20
0
Zm00044a000725_T001
Z. mays A
4.03
Z. mays B
12.28
S. bicolor
54.13
B. distachyon
45.89
O. sativa
−60
−40
−20
0
Zm00044a000726_T001
Z. mays A
4.9
Z. mays B
12.44
S. bicolor
51.54
B. distachyon
45.99
O. sativa
−60
−40
−20
0
Zm00044a000733_T001
Z. mays A
6.73
Z. mays B
12.47
S. bicolor
52.95
B. distachyon
45.9
O. sativa
−60
−40
−20
0
Zm00044a000736_T001
Z. mays A
6.93
Z. mays B
12.08
S. bicolor
54
B. distachyon
45.97
O. sativa
−60
−40
−20
0

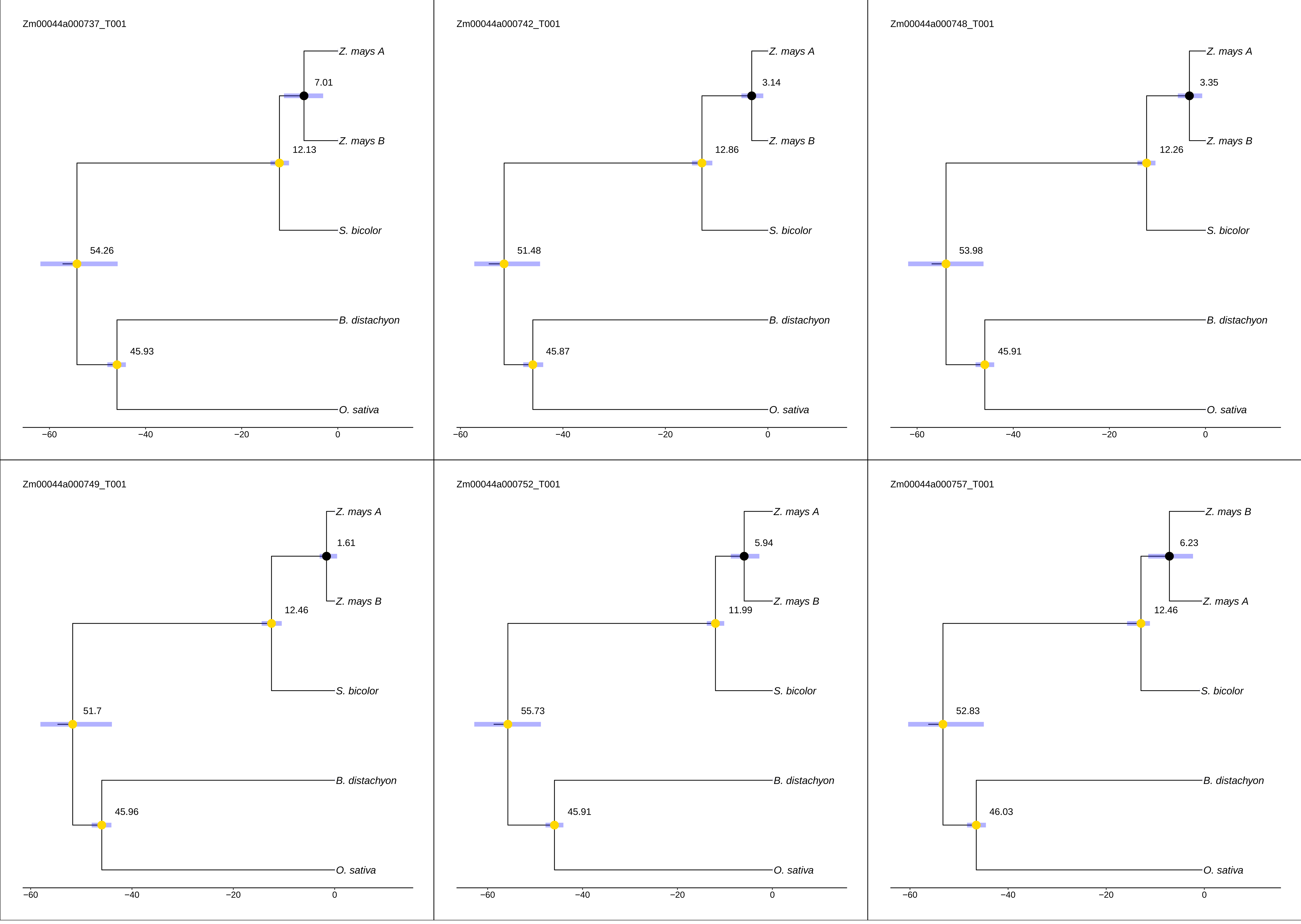

Data S8. Phylogenetic trees of 198 gene sets comprising homologs in all analysed sequences; i.e. genomes of *Brachypodium dystachion*, *Oryza sativa*, *Sorghum bicolor* and *Zea mays* and maize B chromosome sequence.

Tree topologies have been calculated from set of five orthologous genes (copies in the sequence of the maize B, *B. dystachion*, *O. sativa*, *S. bicolor* and *Z. mays*) which were multiple-aligned using MUSCLE. PartitionFinder was used to define the best evolution model for each gene set and BEAST was used to compute the phylogenetic trees using alignments and models previously generated. A consensus tree was produced using TreeAnnotator after burning the hundred first trees. On each tree, the numbers at the nodes correspond to the ages (in MY) estimated by BEAST, and the blue bars give the 95% highest posterior density interval. Yellow dots represent the calibration points for Poaceae, BED clade and Andropogoneae which age priors have been set as normal distribution with the respective value: 55 ± 5 MYA, 46 ± 1 MYA and 12 ± 1 MYA. The black dots indicate the divergence between the B chromosome and the A genome.
